# Inhibition of platelet-surface-bound proteins during coagulation under flow II: Antithrombin and heparin

**DOI:** 10.1016/j.bpj.2022.10.038

**Published:** 2022-11-02

**Authors:** Kenji Miyazawa, Aaron L. Fogelson, Karin Leiderman

**Affiliations:** 1 Quantitative Biosciences and Engineering, Colorado School of Mines, Golden, Colorado; 2 Department of Mathematics, University of Utah, Salt Lake City, Utah; 3 Department of Biomedical Engineering, University of Utah, Salt Lake City, Utah; 4 Mathematics Department, University of North Carolina at Chapel Hill, Chapel Hill, North Carolina; 5 Computational Medicine Program, University of North Carolina at Chapel Hill, Chapel Hill, North Carolina

## Abstract

Blood coagulation is a self-repair process regulated by activated platelet surfaces, clotting factors, and inhibitors. Antithrombin (AT) is one such inhibitor that impedes coagulation by targeting and inactivating several key coagulation enzymes. The effect of AT is greatly enhanced in the presence of heparin, a common anticoagulant drug. When heparin binds to AT, it either bridges with the target enzyme or induces allosteric changes in AT leading to more favorable binding with the target enzyme. AT inhibition of fluid-phase enzymes caused little suppression of thrombin generation in our previous mathematical models of blood coagulation under flow. This is because in that model, flow itself was a greater inhibitor of the fluid-phase enzymes than AT. From clinical observations, it is clear that AT and heparin should have strong inhibitory effects on thrombin generation, and thus we hypothesized that AT could be inhibiting enzymes bound to activated platelet surfaces that are not subject to being washed away by flow. We extended our mathematical model to include the relevant reactions of AT inhibition at the activated platelet surfaces as well as those for unfractionated heparin and a low molecular weight heparin. Our results show that AT alone is only an effective inhibitor at low tissue factor densities, but in the presence of heparin, it can greatly alter, and in some cases shut down, thrombin generation. Additionally, we studied each target enzyme separately and found that inactivation of no single enzyme could substantially suppress thrombin generation.

## Significance

We have developed a novel mathematical model of flow-mediated coagulation that is sensitive to changes in antithrombin levels, especially at low tissue factor densities, and to antithrombin and heparin for all tissue factor densities examined. The sensitivity of the system is entirely due to inhibition reactions that occur on activated platelet surfaces. The model we present here can serve as tool to explore other combinations of target enzymes for optimal inhibition and anticoagulation strategies.

## Introduction

Antithrombin (AT) is an inhibitor of blood coagulation that belongs to the serine protease inhibitor (serpin) superfamily (1) and is found in plasma (2,3). It is continually generated in the liver to maintain a normal level in plasma of approximately 2.4 *μ*M (4). The anticoagulant activity of AT involves the irreversible inactivation (5) of four key serine proteases generated during coagulation: FIXa, FXa, FXIa, and thrombin. Inactivation of FIXa reduces tenase formation (6); inactivation of FXa reduces prothrombinase formation (4); inactivation of FXIa weakens its positive feedback (7); and inactivation of thrombin reduces positive feedback, platelet activation, and overall clot formation (4). A deficiency in AT can lead to excessive thrombin generation (8), which is associated with venous thromboembolism (9).

Although AT is thought to be an important inhibitor by itself, the range of its inactivation rates for coagulation enzymes is quite large, from 10,000 to 14,000/(M⋅s), which covers measurements at both 25°C and 37°C (10,11). Its anticoagulant activity is significantly enhanced in the presence of heparin, which is the oldest anticoagulant drug used in clinical medicine, discovered first by Mclean in 1916 (12,13). Heparin is a naturally occurring glycosaminoglycan that exerts its anticoagulant properties by forming a complex with AT and facilitating an enhanced inhibitory effect of AT on activated coagulation factors (14). Unfractionated heparin (UFH) is a minimally processed form of the natural heparin and thus was the first to be used in medicine. The structure and length of UFH were found to be quite heterogeneous, and this led to unwanted and unpredictable side effects and the need for continuous monitoring during its use as an anticoagulant drug. Further processing to shorten and standardize lengths was desirable to achieve more predictable outcomes (12). These low molecular weight heparins (LMWHs) are derived from UFH using different but controlled manufacturing processes that lead to mean molecular weights that are less than half that of UFH (15,16). Compared with UFH, LMWHs have longer circulating half-lives and higher bioavailability, more predictable outcomes, and less monitoring and are thus largely favored for clinical use (17,18,19,20).

The anticoagulant activity of heparin is its ability to accelerate the inactivation of activated coagulation factors via AT. This activity and the molecular weight of heparin have an interesting and complex relationship. It is now understood that there are two main mechanisms for heparin’s anticoagulant activity. There is allosteric activation of AT by heparin, which alters the structure of AT and enhances its recognition by the various coagulation factors (21,22), and there is also the fact that heparin provides a template to which both AT and coagulation factors can bind and form a ternary bridging complex (23,24). It is thought that the longer the heparin, the more significant the bridging effect can be (11). The allosteric activation mechanism works to inactivate FIXa and FXa but not thrombin or FXIa (21,22). The bridging mechanism can potentially affect all four species, to varying degrees, but thrombin inhibition is solely dependent on this mechanism; thrombin inhibition is significantly enhanced by UFH when there is sufficient room on it for thrombin to bind but much less so by LMWHs (23,24). Enhanced inhibition of FXIa occurs mainly by the bridging mechanism but with a slight variation: FXIa has two binding sites for heparin, one noncatalytic site through which the bridging with AT can occur, and a catalytic site through which heparin can trigger allosteric modulation of FXIa functional activity (in contrast to the allosteric effects on AT described above) (7,25). To summarize, both LMWH and UFH can affect all four enzymes, but they do so to varying degrees, depending on their lengths. In this study, we used a mathematical model of flow-mediated coagulation to explore the effects of LMWH and UFH on inhibition of each individual enzyme and all of them together. We chose to use Nadroparin as the LMWH since there are literature values for its kinetic rate constants along with rates for UFH within the same experimental study (11).

In our companion study focused on tissue factor pathway inhibitor (TFPI), we showed that platelet surfaces played an important yet indirect role in the inhibitory mechanisms of TFPI (26). Platelet-surface-bound enzymes are necessary in coagulation since they help localize coagulation to the site of injury and are many orders of magnitude more efficient than their fluid-phase analogs. However, they are limited by the number of activated platelets and the corresponding binding sites on those platelets’ surfaces. We hypothesized that direct binding of these platelet-bound enzymes by fluid-phase inhibitors would have a stronger overall inhibitory effect on coagulation compared with their binding of fluid-phase enzymes since the fluid-phase complexes are subject to being washed away by flow. Our simulation results confirmed our hypothesis in the case of TFPI, and this led us to hypothesize that AT (with and without heparin) may be working in a similar manner.

To investigate this, we extended the model presented in our companion paper (26) to include surface-dependent inactivation by AT. In our previous model, AT could directly bind and inactivate fluid-phase enzymes only, FIXa, FXa, and thrombin, but these inhibitory reactions showed little to no effect on thrombin generation (27) except under the near-stasis conditions of venous thrombosis (28). Here, we have included the inactivation of FXa, FIXa, and thrombin bound to the platelet surface and, additionally, the inactivation of FXIa in both fluid phase and bound to the platelet surface. Heparin (UFH and LMWH) was explicitly introduced into the model to examine its effects on anticoagulant activity via AT. Our results demonstrated that the inclusion of surface-dependent inactivation magnified the effects of AT and did so to a greater extent when heparin was present. AT in the new version of the model altered thrombin generation at a low, but not high, TF and almost entirely through its effect on surface-bound enzymes. In the presence of heparin, thrombin generation could be significantly delayed and reduced, and these behaviors, too, were completely dependent on direct inhibition of the surface-bound enzymes. In summary, we tested two targets for inhibition of thrombin generation under flow: fluid phase and platelet-bound enzymes. We identified direct binding to platelet-surface-bound enzymes by fluid-phase inhibitors to be the primary mechanism for effective overall inhibition of thrombin generation under flow.

## Materials and methods

### Mathematical model review

Here, we give a brief review of our previously developed mathematical model of flow-mediated coagulation (29,30,31) and the details of the extensions we have made to it. More details about this model and its sensitivity to parameters can be found elsewhere (27). The model simulates the coagulation reactions occurring in a small reaction zone (RZ) above an injury where TF in the subendothelium (SE) is exposed (see model schematic in Fig. S1). Clotting factors and platelets are transported into and out of the RZ by a combination of flow and diffusion using a mass transfer coefficient whose value is a function of vessel and injury size, the flow’s shear rate, and the species’ diffusivity. Clotting factor concentrations in the RZ change due to their involvement in the coagulation reactions and by transport in and out of the zone. Similarly, platelet concentrations change as platelets adhere to the injured wall and become activated and as other platelets are transported in and out of the zone. As platelets build up in the RZ, the height and volume of the RZ increase, with the volume of plasma and concentration of platelets in it changing accordingly. The concentration of each species in the RZ is tracked with an ordinary differential equation; this choice relies on the assumption that each species is uniformly distributed (well mixed) within the RZ. An additional well-mixed endothelial zone (EZ) is located adjacent to the RZ in the direction perpendicular to the flow, with height equal to that of the RZ and the width dependent on the flow shear rate and protein diffusion coefficients. The EZ is where active protein C is produced by a complex formed by thrombomodulin in that zone and thrombin, which has diffused to the EZ from the RZ. This active protein C either diffuses into the RZ or is carried away by the flow.

There are three forms of platelets in the model: unactivated platelets that exist in the plasma phase, activated platelets that are directly attached to the SE, and activated platelets in the thrombus that are not directly attached to the SE. Activation of platelets is achieved by contact with the SE, by interaction with thrombin, or by exposure to already activated platelets (this is an indirect way to model release of agonists from platelet stores). Activated platelets provide the membrane surface necessary for coagulation factors to bind and react. Each activated platelet expresses specified types and numbers of binding sites to which coagulation proteins can selectively bind.

In our companion paper (26), we extended the model to include novel TFPI-mediated inhibition reactions that allow TFPI to act directly on activated platelet-surface-bound species. These reactions enabled enhanced inhibitory effects compared with our previous version(s) of the model. Here, we included an additional extension to incorporate the inactivation of coagulation enzymes by AT and the AT-heparin complex, specifically on the platelet surface.

### Extension in the new model

The new AT/heparin-mediated inactivation reactions include the inactivation of FXa, FIXa, and thrombin bound to the platelet surface and inactivation of FXIa both in the fluid phase and bound to the platelet surface. We previously assumed that the AT-mediated inactivation occurred with a pseudo first-order reaction rate due to the high concentration of AT in the plasma. To track intermediate species more carefully in this study, we have explicitly introduced both AT and heparin as new species. We allow the interaction between AT and heparin to include the formation of complexes, which then have an accelerated inactivation rate that depends on the heparin type (UFH or LMWH). The standard concentration of AT is set to 2.4 *μ*M, and the concentration of heparin depends on the type of heparin (refer to Section S3). The kinetic rates we used are based on the experimentally observed data (11). Additionally, we allow AT to diffuse into the endothelial region and further inhibit the FXa, FIXa, and thrombin that reside there.

**Figure 1:**
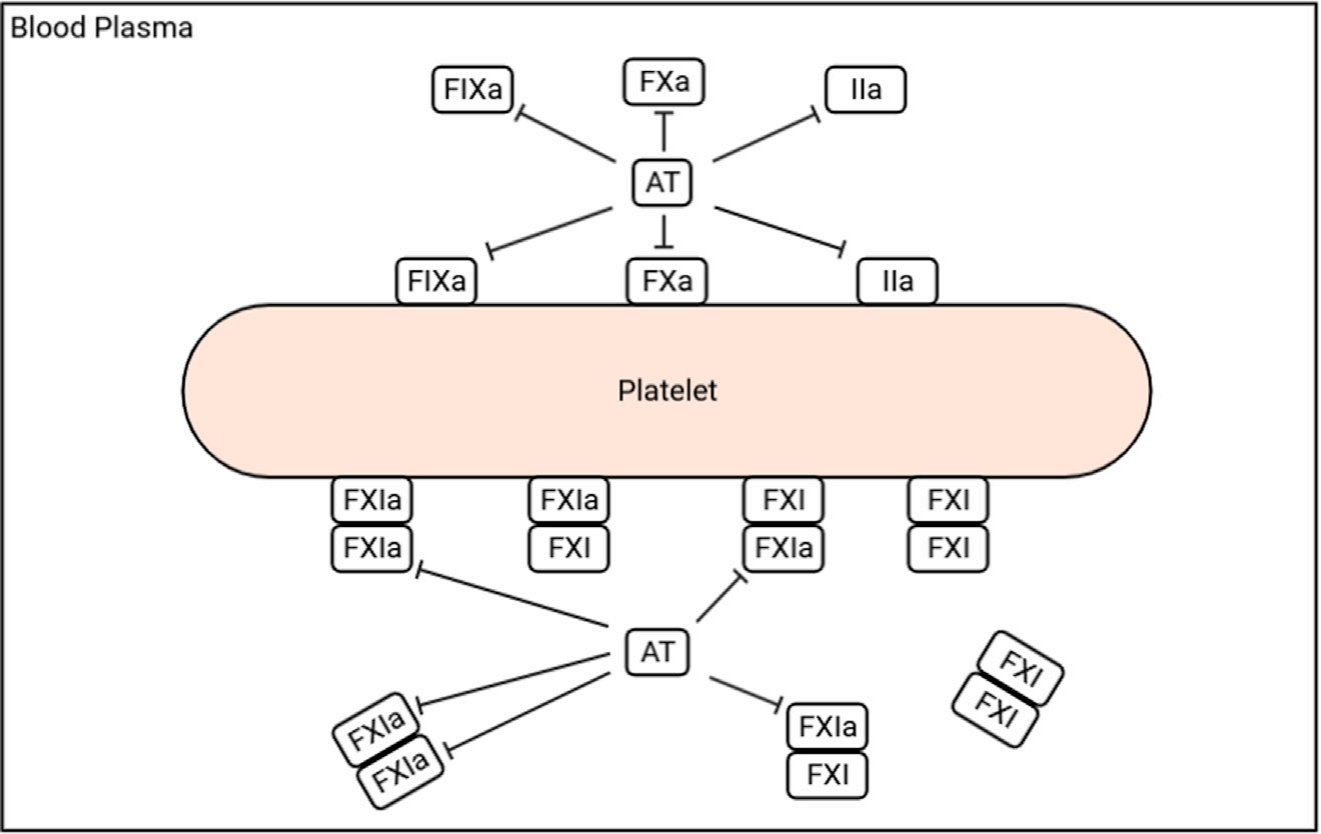
Newly added reactions involving AT, FIXa, FXa, thrombin (IIa), and FXIa. Inactivation reactions are indicated with T-shaped arrows. FXI/FXIa exists as a dimer form, and only the exposed end of FXIa can be inactivated by AT. To see this figure in color, go online.

All of the new reactions involving AT and heparin are sketched in Fig. 1, and the corresponding reactions are listed in Tables 1 and 2. In Eq. 1, we show the evolution equation for the concentration of one of the new species as an example of the nature of the model’s equations. A full listing of the model equations and parameter values is given in Section S2. Note that the ordinary differential equations labeled 1–104 comprise the model from our companion paper (26), and the remaining equations represent the new species in this paper.

**Table 1:** List of reactions added on top of the extension with newly added TFPI-meditaed reactions, and their kinetic rate constants, with literature references

Reaction list							
Reaction no.	Reactants	Products	*k*^+^ (M^−1^s^−1^)	*k*^−^ (s^−1^)	$k_{LMWH}^{+}$ (M^−1^s^−1^)	$k_{UFH}^{+}$ (M^−1^s^−1^)	Note
1	AT+ ${\text{E}}_{9}^{m}$	AT: ${\text{E}}_{9}^{m}$	4.8 ∗ 10^2^	–	–	–	a
2	AT+ ${\text{E}}_{9}^{m*}$	AT: ${\text{E}}_{9}^{m*}$	4.8 ∗ 10^2^	–	–	–	b
3	AT+ ${\text{E}}_{10}^{m}$	AT: ${\text{E}}_{10}^{m}$	3.5 ∗ 10^3^	–	–	–	a
4	AT+ ${\text{E}}_{2}^{m}$	AT: ${\text{E}}_{2}^{m}$	1.4 ∗ 10^4^	–	–	–	a
5	AT + E_11_:Z_11_	AT:E_11_:Z_11_	2.4 ∗ 10^2^	–	–	–	a
6	AT + E_11_:E_11_	AT:E_11_:E_11_	2.4 ∗ 10^2^	–	–	–	a
7	AT + AT:E_11_:E_11_	AT:E_11_:E_11_:AT	2.4 ∗ 10^2^	–	–	–	a
8	AT + E_11_: ${\text{Z}}_{11}^{m}$	AT:E_11_: ${\text{Z}}_{11}^{m}$	2.4 ∗ 10^2^	–	–	–	a
9	AT + E_11_: ${\text{E}}_{11}^{m}$	AT:E_11_: ${\text{E}}_{11}^{m}$	2.4 ∗ 10^2^	–	–	–	a
10	AT + Hep	ATH	1	2.77 ∗ 10^7^	–	–	c
11	ATH+ ${\text{E}}_{9}^{m}$	ATH: ${\text{E}}_{9}^{m}$	–	–	5 ∗ 10^5^	6.2 ∗ 10^6^	d
12	ATH+ ${\text{E}}_{9}^{m*}$	ATH: ${\text{E}}_{9}^{m*}$	–	–	5 ∗ 10^5^	6.2 ∗ 10^6^	b
13	ATH+ ${\text{E}}_{10}^{m}$	ATH: ${\text{E}}_{10}^{m}$	–	–	1.3 ∗ 10^6^	6.6 ∗ 10^6^	d
14	ATH+ ${\text{E}}_{2}^{m}$	ATH: ${\text{E}}_{2}^{m}$	–	–	5.3 ∗ 10^6^	4.7 ∗ 10^7^	d
15	ATH + E_11_:Z_11_	ATH:E_11_:Z_11_	–	–	1 ∗ 10^4^	1.8 ∗ 10^5^	d
16	ATH + E_11_:E_11_	ATH:E_11_:E_11_	–	–	1 ∗ 10^4^	1.8 ∗ 10^5^	d
17	ATH + ATH:E_11_:E_11_	ATH:E_11_:E_11_:AT	–	–	1 ∗ 10^4^	1.8 ∗ 10^5^	d
18	ATH + E_11_: ${\text{Z}}_{11}^{m}$	ATH:E_11_: ${\text{Z}}_{11}^{m}$	–	–	1 ∗ 10^4^	1.8 ∗ 10^5^	d
19	ATH + E_11_: ${\text{E}}_{11}^{m}$	ATH:E_11_: ${\text{E}}_{11}^{m}$	–	–	1 ∗ 10^4^	1.8 ∗ 10^5^	d

k^+^ shows forward reaction rate, and k^−^ shows backward reaction rate. Subscript LMWH and UFH indicates reaction rates when AT is bound to either LWMH or UFH, respectively.

a For inhibition of FIXa by AT, *k*^+^ = 4.8 ∗ 10^2^. For inhibition of FXa by AT, *k*^+^ = 3.5 ∗ 10^3^. For inhibition of thrombin by AT, *k*^+^ = 1.4 ∗ 10^4^. And for inhibition of FXIa by AT, *k*^+^ = 2.4 ∗ 10^2^, from Olson et al. (10).

b We assume the inhibition rate of FIXa and binding rate to platelet on specific binding site are the same as the normal binding site.

c Binding of heparin to antithrombin, K_D_ = 36 nM for LWMH, and K_D_ = 9.7 nM for UFH, from Olson et al. (21).

d Accelerated inhibition of FIXa by AT:UFH complex, *k*^+^ = 6.2 ∗ 10^6^, and AT:LMWH complex, *k*^+^ = 5 ∗ 10^5^. For FXa by AT:UFH complex, *k*^+^ = 6.6 ∗ 10^6^, and AT:LMWH complex, *k*^+^ = 1.3 ∗ 10^6^. For thrombin by AT:UFH complex, *k*^+^ = 4.7 ∗ 10^7^, and AT:LMWH complex, *k*^+^ = 5.3 ∗ 10^6^. And for FXIa by AT:UFH complex, *k*^+^ = 1.8 ∗ 10^5^, and AT:LMWH complex, *k*^+^ = 1 ∗ 10^4^, from Olson et al. (11).

**Table 2:** List of platelet-binding reactions added on top of the extension with newly added TFPI-mediated reactions, and their kinetic rate constants, with literature references

Reaction list					
Reaction no.	Reactants	Products	*k*^*on*^ (M^−1^s^−1^)	*k*^*off*^ (s^−1^)	Note
20	AT:E_9_+P_9_	AT: ${\text{E}}_{9}^{m}$	1 ∗ 10^7^	2.5 ∗ 10^−2^	a
21	AT:E_9_+ ${\text{P}}_{9}^{*}$	AT: ${\text{E}}_{9}^{m*}$	1 ∗ 10^7^	2.5 ∗ 10^−2^	b
22	AT:E_10_ + P_10_	AT: ${\text{E}}_{10}^{m}$	1 ∗ 10^7^	2.5 ∗ 10^−2^	c
23	AT:E_2_+ ${\text{P}}_{2}^{*}$	AT: ${\text{E}}_{2}^{m}$	1 ∗ 10^7^	5.9	d
24	AT:E_11_:Z_11_ + P_11_	AT:E_11_: ${\text{Z}}_{11}^{m}$	1 ∗ 10^7^	0.1	e
25	AT:E_11_:E_11_+ ${\text{P}}_{11}^{*}$	AT:E_11_: ${\text{E}}_{11}^{m}$	1 ∗ 10^7^	0.017	f
26	ATH:E_9_+P_9_	ATH: ${\text{E}}_{9}^{m}$	1 ∗ 10^7^	2.5 ∗ 10^−2^	a
27	ATH:E_9_+ ${\text{P}}_{9}^{*}$	ATH: ${\text{E}}_{9}^{m*}$	1 ∗ 10^7^	2.5 ∗ 10^−2^	b
28	ATH:E_10_ + P_10_	ATH: ${\text{E}}_{10}^{m}$	1 ∗ 10^7^	2.5 ∗ 10^−2^	c
29	ATH:E_2_+ ${\text{P}}_{2}^{*}$	ATH: ${\text{E}}_{2}^{m}$	1 ∗ 10^7^	5.9	d
30	ATH:E_11_:Z_11_ + P_11_	ATH:E_11_: ${\text{Z}}_{11}^{m}$	1 ∗ 10^7^	0.1	e
31	ATH:E_11_:E_11_+ ${\text{P}}_{11}^{*}$	ATH:E_11_: ${\text{E}}_{11}^{m}$	1 ∗ 10^7^	0.017	f

k^*on*^ shows binding rate and k^*off*^ shows unbinding reactions rate.

a Binding of FIXa to platelet surface, K_D_ = 2.5 ∗ 10^−9^, from Ahmad et al. (32).

b We assume the inhibition rate of FIXa and binding rate to platelet on specific binding site are the same as the normal binding site.

c Binding of FXa to platelet surface, K_D_ = 2.5 ∗ 10^−9^, from Walsh et al. (33).

d Binding of thrombin to platelet surface, K_D_ = 5.9 ∗ 10^−7^, from Mann et al. (31).

e Binding of FIX to platelet surface, K_D_ = 1 ∗ 10^−7^, from Greengard et al. (34).

f Binding of FIXa to platelet surface, K_D_ = 1.7 ∗ 10^−7^, from Miller et al. (35).

The model uses the following notation: *Z*_*i*_ and *E*_*i*_ refer to a specific zymogen or procofactor species and the corresponding enzyme or cofactor species when they are in the plasma, $Z_{i}^{m}$ and $E_{i}^{m}$ refer to the surface-bound versions of these proteins (e.g., $E_{7}^{m}$ refers to the TF:VIIa complex on the SE), and *E*_8_ and $E_{8}^{m}$ refer to factor VIIIa in the plasma and bound to a platelet surface, respectively. The concentrations of the proteins are denoted similarly but with lower case *z* and *e*. So, $e_{8}^{m}$ is the concentration of platelet-bound factor VIIIa. The symbols *TF*, *P*_2_, *P*_5_, *P*_8_, *P*_9_, *P*_10_, and *P*_11_ are used to denote TF and the platelet binding sites for prothrombin, FV/FVa, FVIII/FVIIIa, FIX/FIXa, FX/FXa, and FXI, respectively. For the platelet binding sites specific to thrombin, factor IXa, and factor XIa, we use the symbols $P_{2}^{*}$, $P_{9}^{*}$, and $P_{11}^{*}$. The concentrations of binding sites are indicated similarly but with lower case *p*. We denote the complex of *Z*_*i*_ and *E*_*j*_ by *Z*_*i*_:*E*_*j*_ and its concentration by [*Z*_*i*_:*E*_*j*_]; so, for example, $AT:E_{9}^{m}$ denotes AT bound to platelet-bound Factor IXa, and $\left[AT:E_{9}^{m}\right]$ refers to its concentration.

### Inhibition of FXa/FIXa/thrombin by AT

In our previous models (29,30,31), AT inactivated FXa, FIXa, and thrombin in the fluid phase only. Now, AT can additionally inactivate all of these enzymes when they are bound to the platelet surface (reactions 1–4 in Table 1). We assume the inactivation rates to be the same for both fluid-phase and platelet-bound enzymes and allow the inactivated AT-enzyme complex to bind/unbind from the platelet surface with kinetic rates that match those of the enzyme itself. We also assume that platelet-bound enzymes, which are also bound to AT, occupy their corresponding binding site. For example, one AT:FXa complex on the membrane, $AT:E_{10}^{m}$, takes up one FX/FXa binding site.

### Inactivation of FXIa by AT

In our new model, we introduce the inactivation of FXIa by AT in both fluid- and platelet-bound phases (reactions 5–9 in Table 1). Since FXI and FXIa are dimers, we have included inactivation of only the FXIa part of the several dimeric forms that involve FXI and FXIa: FXI:FXI, FXI:FXIa, and FXIa:FXIa in the fluid. Each of these can also be bound to the platelet surface. We assume that when FXIa is bound directly to a platelet binding site, it does not participate in any reactions and that AT can only inhibit the free FXIa end of the dimer that is not directly bound. For example, AT can bind and inhibit FXIa in the FXIa:FXIa complex in the fluid phase on either or both ends (reaction 6 in Table 1) but can only inhibit the FXIa that is exposed to the fluid when the other end is bound to the platelet (reactions 8 and 9 in Table 1, respectively). The detailed FXI/FXIa complexes and available inhibition sites are also shown in the reaction schematic in Fig. 1.

### Introduction of heparin

Heparin is introduced as a new species with a fixed upstream concentration set to 253 and 759 nM for UFH and LMWH, respectively, based on the recommended therapeutic range 0.3–0.7 U/mL (36). The conversion of heparin potency to molar concentration can be found in Section S3. We also varied this concentration to understand how this affects heparin’s impact.

### Inactivation by AT:Heparin complex

We assume that AT can bind to heparin and form the complex we call *ATH* (reaction 10 in Table 1). The AT:Heparin complex inactivates FXa, FIXa, FXIa, and thrombin in either the fluid- or platelet-bound phase by direct binding (reactions 11–19 in Table 1). When AT is bound to LMWH, we simply change the kinetic rate constants to represent Nadroparin, which greatly accelerates the inactivation of FIXa (by 3 orders of magnitude), accelerates inactivation of FXa and thrombin to a lesser extent (by a factor of 370), and accelerates FXIa inactivation by a yet smaller amount (by a factor of 40). When AT is bound to UFH, we use the reaction kinetic reported from the same experiment, which indicates that it accelerates inactivation of all of the targeted enzymes (750–13,000 times increase) more potently than does LMWH. Platelet-bound enzymes that are inactivated by AT:heparin complexes can also bind/unbind from the platelet surface and, while bound, occupy the corresponding enzyme binding site on the platelet (reactions 20–31 in Table 2).

### Example equation

Newly added reactions listed in Table 1 were expressed mathematically using the law of mass action. For example, the following equation describes the rate of change of the platelet-bound concentration of FXa bound to AT. It accounts for the binding and unbinding of the complex FXa:AT with the platelet surface and the direct binding of AT from the fluid to platelet-bound FXa:

(1) $$\frac{d\left[E_{10}^{m}:AT\right]}{dt}=\underset{\text{FXa:AT binding to platelet}}{\underset{︸}{k_{10}^{\text{on}}\left[E_{10}:AT\right]p_{10}^{\text{avail}}}}\;-\underset{\text{FXa:AT unbinding from platelet}}{\underset{︸}{k_{10}^{\text{off}}\left[E_{10}^{m}:AT\right]}}+\;\underset{︸}{k_{e_{10}^{m}:\text{AT}}^{+}\cdot e_{10}^{m}\cdot AT}._{\text{Inactivation of platelet}\text{-}\text{bound FXa by AT}}.$$

Here, $p_{10}^{\text{avail}}$ is the concentration of FX/FXa binding sites on activated platelets that are not occupied by an FX- or FXa-containing species.

## Results

### TF and shear rate dependence

**Figure 2:**
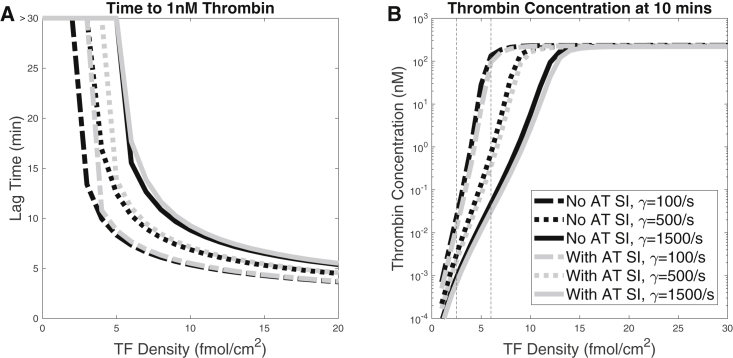
(*A*) Lag time and (*B*) thrombin concentration after 10 min of activity, with variations in TF density and shear rate. TF density was varied from 0 to 30 fmol/cm^2^, and shear rate was either 100, 500, or 1,500/s. “SI” in the legend represents surface-dependent inactivation. Black lines represent results with AT-mediated inactivation of fluid-phase enzymes and noinactivation of surface-bound enzymes, and gray lines represent the results with inactivation of both fluid-phase and surface-bound enzymes by AT. Vertical dashed lines indicate TF densities of interest. Flat curves at >30 min indicate a lag time larger than 30 min in (*A*).

Here, we examined how the variation in TF density and shear rate affected thrombin production with the new AT inhibitory mechanisms at the platelet surface and compared the outcomes with those without that surface inhibition. For various TF densities in the range 0–30 fmol/cm^2^ and for shear rates 100, 500, and 1,500/s, we performed simulations with and without surface inhibition (denoted “SI” in the figure). We looked at two output metrics, the lag time, which we define as the time point at the thrombin concentration first reaches 1 nM, and the thrombin concentration at 10 min. Fig. 2 *A* shows the lag times, and Fig. 2 *B* shows the thrombin concentrations at 10 min.

With and without the surface inhibition, the lag time decreased as the TF density increased and/or the shear rate decreased. These behaviors occur because a higher TF density provides a larger initial stimulus and decreasing the shear rate slows the loss of essential enzymes from the RZ. Also, the thrombin concentrations at 10 min increased with the TF density, sharply at low TF densities and more gradually at high ones. In fact, the results indicated a threshold dependence on TF density in all cases examined. (We refer to curves of thrombin at 10 min versus TF density as threshold curves.) The thrombin concentration at 10 min, however, was only very slightly affected by the shear rate (Fig. 2 *B*). In summary, the effects of TF density and shear rate remain qualitatively the same in the presence of the new AT reactions without heparin-mediated acceleration.

### Influence of AT level on thrombin generation

**Figure 3:**
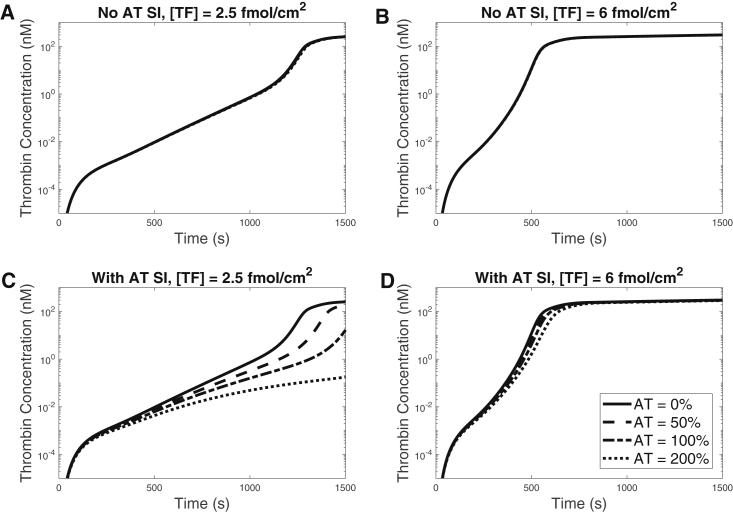
Thrombin time courses for various AT levels, with and without surface-dependent inactivation reactions. SI denotes surface-mediated inactivation. Shown in the top row of the figure are TF = (*A*) 2.5 and (*B*) 6 fmol/cm^2^, each without SI. Shown in the bottom row of the figure are TF = (*C*) 2.5 and (*D*) 6 fmol/cm^2^, each with SI. AT level was 0%, 50%, 100%, or 200% of 2.4 *μ*M. Shear rate was set to 100/s for all simulations.

Next, we compared how thrombin generation was affected by various AT levels at low versus high TF density. We varied the AT level from 0% to 200% of its physiological concentration (2.4 *μ*M) and used 4 and 15 fmol/cm^2^ for the low and high TF densities, respectively. Fig. 3 shows plots of thrombin generation with (Fig. 3, *C* and *D*) and without (Fig. 3, *A* and *B*) surface inhibition by AT for low TF (Fig. 3, *A* and *C*) and high TF (Fig. 3, *B* and *D*). In the absence of the surface AT reactions, there is no to little change in the thrombin generation for all levels of AT (Fig. 3, *A* versus *B*). When the AT surface reactions are present, thrombin generation is significantly delayed at the low, but not the high, TF density (Fig. 3, *C* and *D*). Another observation is that the concentration of thrombin after 20 min of activity remains almost the same in all cases; similar to our TFPI study, the AT affects mainly the timing of the thrombin burst. In summary, surface-dependent inactivation by AT affects thrombin generation, but this is more prominent at a low TF density. Since measurements of plasma levels of thrombin-AT (TAT) are used clinically, we calculated the instantaneous rates of generation of TAT complexes (in the plasma and on the platelet surfaces) and their removal by flow for various levels of AT. We found that more TAT was generated than carried away by flow, with the majority staying bound to platelet surfaces, as shown in Fig. S8.

### Influence of heparin level on thrombin generation

**Figure 4:**
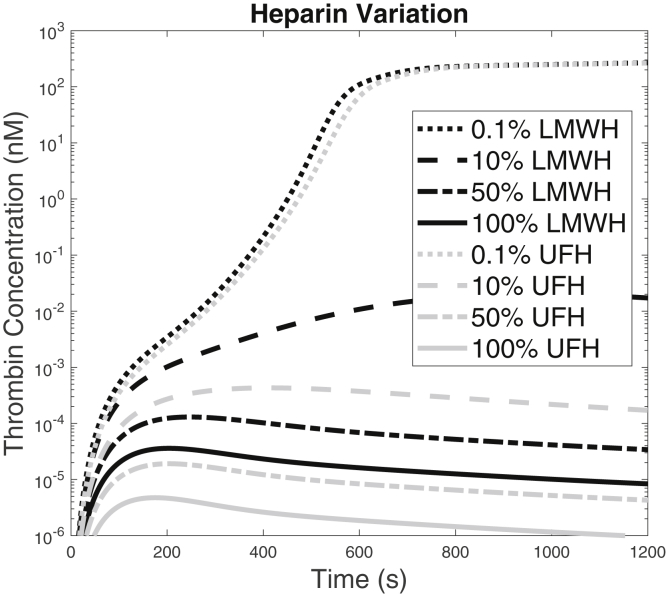
Thrombin generation with TF = 6 fmol/cm^2^ and varied levels of LWMH and UFH. Thrombin generation in the presence of LMWH and UFH are shown in black and gray lines, respectively. The heparin levels were varied as 0.1%, 10%, 50%, or 100% of a standard therapeutic concentration.

The effect of different heparin concentrations was also examined. The thrombin time courses shown in Fig. 4 were generated for different heparin concentrations (0.1%, 10%, 50%, and 100%) for both LWMH (where 100% = 253 nM) and UFH (for which 100% = 759 nM). The values for 100% were based on recommended dosages, and the conversions and references are in the supporting material. As the level of heparin was increased, the delay in thrombin generation was increased, and at the therapeutic concentration, both types of heparins prevented the thrombin concentration from reaching 1 nM. As expected, UFH had a higher overall inhibitory effect. Our new model exhibits the distinct and clinically observed effects on anticoagulant activity of LMWH and UFH.

### Effects of surface-dependent AT inactivation on thrombin, FIXa, FXa, and FXIa

**Figure 5:**
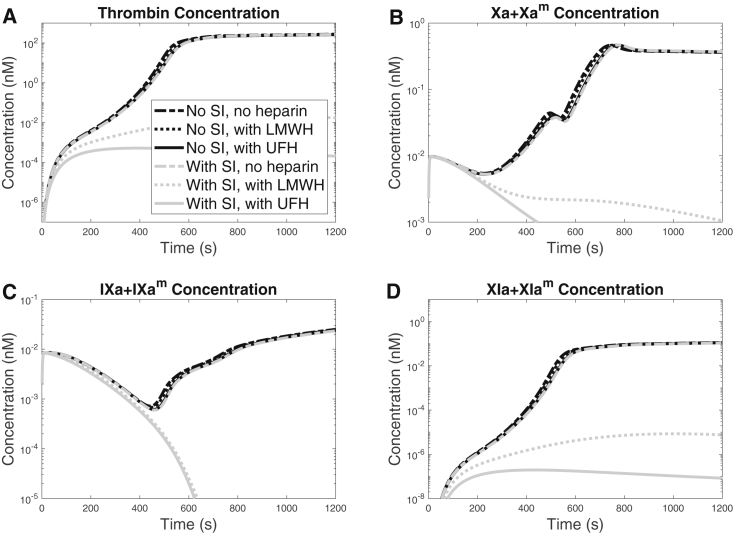
Concentration time courses of (*A*) thrombin, (*B*) FXa, fluid phase and surface bound, (*C*) FIXa, fluid phase and surface bound, and (*D*) FXIa, fluid phase and surface bound, each with a TF density of 15 fmol/cm^2^ and a shear rate of 100/s. Simulations were performed with or without surface-dependent AT inactivation reactions (SI, surface-mediated inactivation), which are shown as gray and black curves, respectively, and for no heparin, LMWH, or UFH, shown by varying line styles.

To study the individual enzymes and the effect of inactivation on their concentrations, we tracked the time courses of thrombin, FXa, FIXa, and FXIa. Fig. 5 shows plots of the sum of both the fluid-phase and platelet-bound species. We examined these concentrations in the absence and presence of the surface-dependent inactivation by AT, and under the condition of no heparin, with LWMH or with UFH at 25% of their therapeutic concentrations. For all species, the cases without surface-dependent inactivation by AT or ATH (antithrombin bound to heparin) showed almost indistinguishable curves, meaning that AT and ATH were not effective at reducing enzyme concentrations when inactivating fluid-phase enzymes only. In the presence of surface-dependent inactivation by AT, but in the absence of heparin, the concentrations of all species look similar to the case with no surface-dependent inactivation. With surface-dependent inactivation, both the LMWH and UFH substantially reduced the concentrations of all enzymes, with the strongest effects from UFH. These results collectively show that the surface-dependent inhibition reactions are critical and necessary to induce observable effects of heparin on its target enzymes.

### Examination of the major inactivation reactions

**Figure 6:**
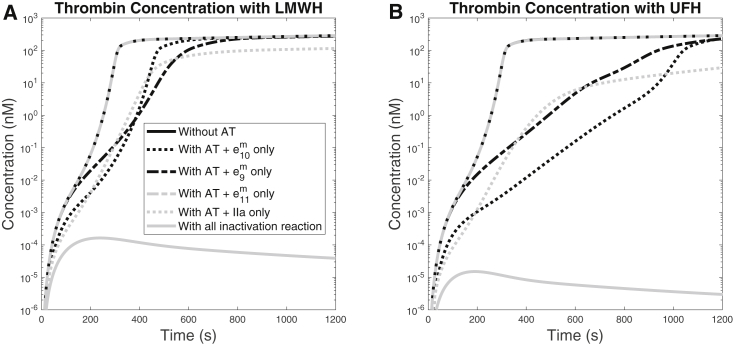
Thrombin generation in the presence of (*A*) LMWH or (*B*) UFH. The time course is obtained from simulations in which we turn off all AT-mediated inactivation reactions and then allow inhibition of FXa, FIXa, FXIa, and thrombin, individually and one by one. Each curve thus shows thrombin generation when there is either no or only one inactivation reaction that exists in the system. TF density was set to 15 fmol/cm^2^, and shear rate was set to 100/s. Heparin concentration is fixed to 100% of the standard therapeutic concentration.

To understand the effects of inactivating each enzyme alone, we separately kept individual inactivation reactions “turned on” while “turning off” the remaining reactions; turning off a reaction here means that we set the corresponding association rates to zero. For example, to focus on the effects of inactivating FIXa alone, we keep AT inactivation of FIXa turned on while setting AT association rates for FXa, FXIa, and thrombin to zero. For these studies, we used a TF density of 15 fmol/cm^2^ and a shear rate of 100/s. Fig. 6 shows the resulting thrombin time courses under the influence of either LMWH (Fig. 6 *A*) or UFH (Fig. 6 *B*). Simulations with LMWH and UFH showed similar trends. In both cases, inactivating FXIa had a negligible effect. Inactivating either FIXa, FXa, or thrombin increased the lag time; with LMWH, the lag times were increased by about 200 s, and with UFH, they were increased by about 400 s for FIXa and thrombin inactivation and by about 800 s for FXa inactivation. The thrombin concentrations after 20 min were about the same for individual inactivation of FIXa, FXa, and FXIa, around 150 nM. For inactivation of thrombin, the thrombin concentration after 20 min is reduced to about 100 nM with LMWH and about 20 nM with UFH; this is due to a reduction in both positive feedback and the direct inactivation itself. When LMWH and UFH are working to their full potential, i.e., they are inactivating all four enzymes to their respective degrees, the thrombin generation is essentially shut off, with concentrations that never even reach 1 pM. In summary, our model shows that inactivation of any of the single target enzymes alone is not enough to prevent robust thrombin generation; inhibiting multiple targets together enables accumulative inhibition to completely extinguish thrombin generation.

## Discussion

In our previous mathematical models of flow-mediated coagulation (29,30,31), AT inactivated FIXa, FXa, and thrombin in the fluid phase only, and this had little effect on thrombin production since the fluid-phase species, whether inactivated or not, were subject to flow and could easily be washed away. Our extended model, which includes direct binding of fluid-phase AT to platelet-bound species, shows a new sensitivity of the model coagulation system to AT. In particular, we find that AT can dramatically increase the lag time of thrombin generation at low TF density through this surface-dependent inhibition mechanism. However, at high TF, even when AT was increased to 200% of its normal concentration, the changes in thrombin generation are slight. Addition of these new reactions does not significantly alter the TF density threshold or shear rate dependence. We found only small increases in the lag time and slight changes in thrombin concentration after 10 min of clotting activity, when compared with simulations run in the absence of the AT-mediated inactivation reactions, over a wide range of TF densities. As observed in our previous studies (29,30,31), increasing the flow shear rate increases the lag time and reduces the thrombin concentration after 10 min of clotting activity.

The presence of heparin greatly magnifies the sensitivity of the system to AT, and this sensitivity is due entirely to the direct binding of platelet-bound species by fluid-phase ATH. When the AT-mediated inactivation of platelet-surface-bound enzymes is turned off, neither type of heparin has any noticeable effect on the system. This highlights the critical role that the platelet surface plays in this process. We find that UFH at 10% of a therapeutic level or LMWH at 100% of a therapeutic level can completely shut down thrombin generation, even at high TF densities. These results are generally in line with observed responses to heparin therapies in the sense that UFH is a stronger inhibitor than LMWH; this is because UFH affects the inactivation of FIXa, FXa, FXIa, and thrombin, whereas LMWH affects mainly the inactivation of FIXa and FXa and has less influence on the inactivation of thrombin and FXIa.

In our model we do not include direct effects of heparin on the rate of platelet deposition in the reactions. The only way that platelet deposition can be affected by heparin in this model is indirectly through its effect on thrombin generation and thrombin’s activation of platelets. In particular, less thrombin from the heparin will reduce positive feedback and will also reduce the number of platelets activated by thrombin. Some plots of the platelet dynamics with varying types and concentrations of heparin are shown in the Fig. S6; heparin indeed reduces the platelet deposition.

It has been shown that the addition of heparin can also accelerate the inhibitory effect of TFPI, as shown in prothrombinase activity assays initiated with FXa, FV, prothrombin, and lipids, but such an effect was greatly diminished when FXa was preincubated with partially activated FV (37). These data are in line with several observations showing procoagulant effects of heparin when AT is not present in the system (37,38). Wood et al. further explored these ideas and showed that the negatively charged heparin molecule can block the interaction between TFPI*α* and partially activated FV (FV-h) (39). Therefore, addition of heparin can reduce the inhibitory effect of TFPI*α* toward prothrombinase that is made with FV-h but has no effect on prothrombinase made with fully activated FVa. Our model does not reflect the reduced binding interaction between TFPI*α* and FV-h by heparin treatment, but our model and results can still give some insight about what might happen under these conditions. The results in our companion TFPI study (26) showed that the coagulation response in the absence of binding between TFPI*α* and FV-h was enhanced. That scenario resembles the situation where heparin would block the binding of TFPI*α* to FV-h. We note that AT-mediated inactivation in that version of the model had no effect because it only acted on fluid-phase enzymes, thus we can consider it to be a case in which there is essentially no AT, isolating the procoagulant effects of heparin to be through TFPI*α*/FV-h interactions. Nevertheless, further exploration of procoagulant effects of heparin and the extension of heparin-TFPI*α* would be an interesting topic for future work.

The model allowed us to do simulations in which we could isolate the effect of inhibiting one coagulation enzyme at a time by setting the rate constants for other enzymes to zero. In doing this systematically, we found that no one single enzyme inactivation was enough to prevent substantial thrombin generation under flow for the TF densities examined. Inactivating FXIa alone had almost no effect. Inactivation of each FIXa, FXa, or thrombin individually by either LMWH or UFH led to increased lag time. The lag times were increased more for UFH than LMWH. Substantial thrombin was still produced by 20 min in these cases. The strength of heparin inhibition seems to be the simultaneous enzyme targets at multiple steps in the coagulation system.

There are other types of LMWH heparins and derivatives of UFH that have been developed for use as anticoagulant drugs (11,12,15) that we did not study in this work. There is a large body of clinical research to understand which heparins and their derivatives work best for various indications, for example treatment of deep vein thrombosis versus thromboprophylaxis after surgery. Complete details are beyond the scope of this work, but we point interested readers to a few published reviews (40,41). Researchers are still trying to identify individual risk factors associated with bleeding when using heparin as an anticoagulant treatment (42,43). Even though newer types of anticoagulants are being developed, advanced, and frequently used in place of heparins (44), both LMWH and UFH were effective in managing clotting complications of COVID-19 (45,46).

## Conclusion

In this study, we explored the effects of surface-dependent inactivation by AT in a model coagulation system under flow. As we concluded in our companion (26) TFPI study, targeting the enzymes bound to activated platelet surfaces was required for efficient inhibition of thrombin generation. We showed that AT alone can delay thrombin generation when TF density is low but not when TF density is high. This inhibition was completely dependent on the platelet surface reactions. Our results show that AT in the presence of heparin can drastically inhibit thrombin generation. We developed a new version of our mathematical model that is sensitive to AT and heparin in ways that have been observed clinically, i.e., the magnitude of the effects of LMWH versus UFH. We found that inactivating single enzymes only was ineffective at suppressing thrombin generation. Combinations of two or three targets could be examined to aid in the design of new anticoagulant drugs. The model we developed in this study and in our companion TFPI study (26) will serve as a powerful tool for such use in future studies.

## Author contributions

K.M. carried out all simulations. K.M., A.L.F., and K.L. designed the research, analyzed the data, and wrote the article.

## Supporting material

Document S1. Figures S1–S8 and Tables S1–S14

Document S2. Article plus supporting material
