# Supplementary material for: Inhibition of platelet-surface-bound proteins during coagulation under flow II: Antithrombin and heparin
Source: Biophys J. 2022 Nov 2;122(1):230–40. doi: 10.1016/j.bpj.2022.10.038 (PMC9822793; doi:10.1016/j.bpj.2022.10.038)
Supplement: Document S1. Figures S1–S8 and Tables S1–S14 [file mmc1.pdf]

Biophysical Journal, Volume *122*

Supplemental information

Inhibition of platelet-surface-bound proteins during coagulation under flow II: Antithrombin and heparin

Kenji Miyazawa, Aaron L. Fogelson, and Karin Leiderman

Manuscript submitted to **Biophysical** *Journal*

Article

Supplementary Information for: Inhibition of platelet-surface-bound proteins during coagulation under flow

Kenji Miyazawa, Aaron L. Fogelson, Karin Leiderman

S1 MODEL SCHEMATIC

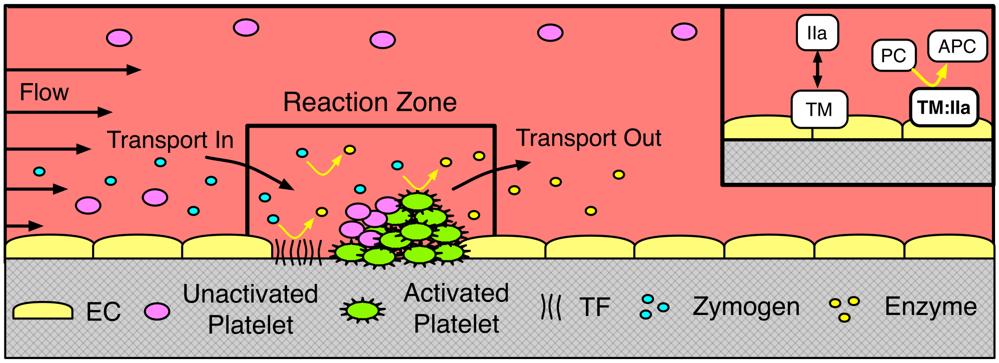

Figure S1: Schematic of the model reaction zone (main figure) and endothelial zone (inset).

S2 MODEL EQUATIONS

Below we have listed the full model equations for all species. The model detailed here includes extensions of our previous work (1–3). In total, the model consists of 130 species (and their corresponding ordinary differential equations) and 239 parameters including kinetic rates and initial/upstream concentrations. The solution of the model equations was carried out with our in-house fortran code that uses DLSODE for the numerical solution of the differential equations; each run of the model that simulates 40 minutes of clotting activity takes less than 10 seconds on a linux-based laptop. Simulations of this model (in the absence of heparin) can be performed with our online coagulation simulator: ClotSims. Below, blue text indicates the additional terms we added for the TFPI-mediated reactions, and purple text indicates the additional terms we added for the AT-mediated reactions. Strikeouts shows the terms we have removed from our previous version of the model.

$$
\begin{aligned}
\frac{d}{dt}z_7 &= -k_7^{on} z_7 [TF]^{avail} + k_7^{off} z_7^m \\
&\quad -k^{+}_{z_7:e_2} z_7 e_2 + k^{-}_{z_7:e_2}[Z_7 : E_2] \\
&\quad -k^{+}_{z_7:e_{10}} z_7 e_{10} + k^{-}_{z_7:e_{10}}[Z_7 : E_{10}] \\
&\quad +k_{flow}(z_7^{up} - z_7) \\
&\quad -k^{+}_{z_7:e_9} z_7 e_9 + k^{-}_{z_7:e_9}[Z_7 : E_9]
\end{aligned} \tag{1}
$$

$$
\begin{aligned}
\frac{d}{dt}e_7 &= -k_7^{on} e_7 [TF]^{avail} + k_7^{off} e_7^m \\
&\quad +k^{cat}_{z_7:e_2}[Z_7 : E_2] + k^{cat}_{z_7:e_{10}}[Z_7 : E_{10}] \\
&\quad k_{flow}(e_7^{up} - e_7) + k^{cat}_{z_7:e_9}[Z_7 : E_9]
\end{aligned} \tag{2}
$$

$$
\begin{aligned}
\frac{d}{dt}z_{10} &= -k_{10}^{on} z_{10} p_{10}^{avail} + k_{10}^{off} z_{10}^m \\
&\quad -k^{+}_{z_{10}:e_7^m} e_7^m + k^{-}_{z_{10}:e_7^m}[Z_{10} : E_7^m] \\
&\quad k_{flow}(z_{10}^{up} - z_{10})
\end{aligned} \tag{3}
$$

$$
\begin{aligned}
\frac{d}{dt}e_{10} &= -k_{10}^{on} e_{10} p_{10}^{avail} + k_{10}^{off} e_{10}^m \\
&\quad +k^{cat}_{z_{10}:e_7^m}[Z_{10} : E_7^m] \\
&\quad +(k^{cat}_{z_7:e_{10}} + k^{-}_{z_7:e_{10}})[Z_7 : E_{10}] - k^{+}_{z_7:e_{10}} e_{10} z_7 \\
&\quad +(k^{cat}_{z_7^m:e_{10}} + k^{-}_{z_7^m:e_{10}})[Z_7^m : E_{10}] - k^{+}_{z_7^m:e_{10}} e_{10} z_7^m \\
&\quad -k^{+}_{TFPI:e_{10}} e_{10}[TFPI] + k^{-}_{TFPI:e_{10}^m}[TFPI : E_{10}] \\
&\quad k_{flow}(e_{10}^{up} - e_{10}) \\
&\quad \cancel{-k^{in}_{AT:e_{10}} e_{10}} \\
&\quad -k_{diff}(e_{10} - e_{10}^{ec}) \\
&\quad -k^{+}_{TFPI:e_5^h:e_{10}}[TFPI : E_5^h]e_{10} \\
&\quad -k^{-}_{TFPI:e_5^h:e_{10}}[E_{10} : TFPI : E_5^h] \\
&\quad -k^{+}_{TFPI:e_5^{hm}:e_{10}}[TFPI : E_5^{hm}]e_{10} \\
&\quad +k^{-}_{TFPI:e_5^{hm}:e_{10}}[E_{10} : TFPI : E_5^{hm}] \\
&\quad -k^{AT}_{e_{10}} e_{10}[AT] \\
&\quad +k^{ATH}_{e_{10}} e_{10}[AT : Hep]
\end{aligned} \tag{4}
$$

$$
\begin{aligned}
\frac{d}{dt}z_{10}^{m} &= k_{10}^{on}z_{10}p_{10}^{avail} - k_{10}^{off}z_{10}^{m} \\
&\quad k_{z_{10}^{m}:TEN}^{+}z_{10}^{m}[TEN] + k_{z_{10}^{m}:TEN}^{-}[Z_{10}^{m}:TEN] \\
&\quad -k_{z_{10}^{m}:TEN}^{+}z_{10}^{m}[TEN^{*}] + k_{z_{10}^{m}:TEN}^{-}[Z_{10}^{m}:TEN^{*}]
\end{aligned} \tag{5}
$$

$$
\begin{aligned}
\frac{d}{dt}e_{10}^{m} &= k_{10}^{on}e_{10}p_{10}^{avail} - k_{10}^{off}e_{10}^{m} \\
&\quad +k_{z_{10}^{m}:TEN}^{cat}[Z_{10}^{m}:TEN] + (k_{z_{5}^{m}:e_{10}^{m}}^{cat} + k_{z_{5}^{m}:e_{10}^{m}}^{-})[Z_{5}^{m}:E_{10}^{m}] \\
&\quad -k_{z_{5}^{m}:e_{10}^{m}}^{+}e_{10}^{m}z_{5}^{m} + (k_{z_{8}^{m}e_{10}^{m}}^{cat} + k_{z_{8}^{m}e_{10}^{m}}^{-})[Z_{8}^{m}:E_{10^{m}}] \\
&\quad -k_{z_{8}^{m}:e_{10}^{m}}^{+}e_{10}^{m}z_{8}^{m} \\
&\quad +k_{e_{5}^{m}:e_{10}^{m}}^{-}[PRO] - k_{e_{5}^{m}:e_{10}^{m}}^{p}e_{10}^{m}e_{5}^{m} \\
&\quad +k_{z_{10}^{m}:TEN}^{cat}[Z_{10}^{m}:TEN^{*}] \\
&\quad -k_{e_{5}^{hm}:e_{10}^{m}}^{+}e_{10}^{m}e_{5}^{hm} + k_{e_{5}^{hm}:e_{10}^{m}}^{-}PRO^{h} \\
&\quad -k_{TFPI:e_{10}^{m}}^{+}e_{10}^{m}TFPI + k_{TFPI:e_{10}^{m}}^{-}[TFPI:E_{10}^{m}] \\
&\quad -k_{TFPI:e_{5}^{hm}:e_{10}^{m}}^{+}[TFPI:E_{5}^{hm}]e_{10}^{m} \\
&\quad +k_{TFPI:e_{5}^{hm}:e_{10}^{m}}^{-}[E_{10}^{m}:TFPI:E_{5}^{hm}] \\
&\quad -k_{TFPI:e_{10}:e_{5}^{hm}}^{+}[TFPI:E_{5}^{hm}]e_{10}^{m} \\
&\quad +k_{TFPI:e_{10}:e_{5}^{hm}}^{-}[TFPI:PRO^{h}]^{v5} \\
&\quad -k_{e_{10}^{m}}^{AT}e_{10}^{m}[AT] \\
&\quad -k_{e_{10}^{m}}^{ATH}e_{10}[AT:Hep]
\end{aligned} \tag{6}
$$

$$
\begin{aligned}
\frac{d}{dt}z_{5} &= -k_{5}^{on}z_{5}p_{5}^{avail} + k_{5}^{off}z_{5}^{m} \\
&\quad -k_{z_5:e_2}^{+}z_{5}e_{2} + k_{z_5:e_2}^{-}[Z_{5}:E_{2}] \\
&\quad k_{flow}(z_{5}^{up} - z_{5}) \\
&\quad +n_{5}(k_{adh}^{+}p_{PLAS}^{avail} + k_{plt}^{act}([PL_{a}^{v}] + [PL_{a}^{s}]) + k_{e2}^{act}\frac{e_{2}}{e_{2}+0.001})[PL]
\end{aligned} \tag{7}
$$

$$
\begin{aligned}
\frac{d}{dt}e_{5} &= -k_{5}^{on}e_{5}p_{5}^{avail} + k_{5}^{off}e_{5}^{m} \\
&\quad +k_{z_5:e_2}^{cat}[Z_{5}:E_{2}] \\
&\quad +k_{flow}(e_{5}^{up} - e_{5}) \\
&\quad k_{e_5:APC}^{-}[APC:E_{5}] - k_{e_5:APC}^{+}e_{5}[APC] \\
&\quad +k_{e_{5}^{h}:e_2}^{cat}[E_{5}^{h}:E_{2}]
\end{aligned} \tag{8}
$$

$$
\begin{aligned}
\frac{d}{dt}z_{5}^{m} &= k_{5}^{on}z_{5}p_{5}^{avail} - k_{5}^{off}z_{5}^{m} \\
&\quad -k_{z_{5}^{m}e_{10}^{m}}^{+}z_{5}^{m}e_{10}^{m} + k_{z_{5}^{m}:e_{10}^{m}}^{-}[Z_{5}^{m}:E_{10}^{m}] \\
&\quad -k_{z_{5}^{m}:e_{2}^{m}}^{+}z_{5}^{m}e_{2}^{m} + k_{z_{5}^{m}e_{2}^{m}}^{-}[Z_{5}^{m}:E_{2}^{m}]
\end{aligned} \tag{9}
$$

$$\frac{d}{dt}e_5^m = k_5^{on}e_5p_5^{avail} - k_5^{off}e_5^m$$
$$+k^{cat}_{z_5^m:e_2^m}[Z_5^m:E_2^m]$$
$$+k^{-}_{e_5^m:APC}[APC:E_5^m]$$
$$-k^{+}_{e_5^m:APC}e_5^m[APC]$$
$$-k^{+}_{e_5^m:e_{10}^m}e_5^me_{10}^m + k^{-}_{e_5^m:e_{10}^m}[PRO]$$
$$+k^{cat}_{e_5^{hm}:e_2^m}[E_5^{hm}:E_2^m] \quad (10)$$

$$\frac{d}{dt}z_8 = -k_8^{on}z_8p_8^{avail} + k_8^{off}z_8^m$$
$$+k_{flow}(z_8^{up} - z_8)$$
$$-k^{+}_{z_8:e_2}z_8e_2 + k^{-}_{z_8:e_2}[Z_8:E_2] \quad (11)$$

$$\frac{d}{dt}e_8 = -k_8^{on}e_8p_8^{avail} + k_8^{off}e_8^m$$
$$k_{flow}(e_8^{up} - e_8)$$
$$+k^{cat}_{z_8:e_2} - 0.005e_8$$
$$+k^{-}_{e_8:APC}[APC:E_8] - k^{+}_{e_8:APC}e_8[APC] \quad (12)$$

$$\frac{d}{dt}z_8^m = k_8^{on}z_8p_8^{avail} - k_8^{off}z_8^m$$
$$-k^{+}_{z_8^m:e_{10}^m}z_8^me_{10}^m + k^{-}_{z_8^m:e_{10}^m}[Z_8^m:E_{10}^m]$$
$$-k^{+}_{z_8^m:e_2^m}z_8^me_2^m$$
$$+k^{-}_{z_8^m:e_2^m}[Z_8^m:E_2^m] \quad (13)$$

$$\frac{d}{dt}e_8^m = k_8^{on}e_8p_8^{avail} - k_8^{off}e_8^m$$
$$k^{cat}_{z_8^m:e_{10}^m}[Z_8^m:E_{10}^m] + k^{cat}_{z_8^m:e_2^m}[Z_8^m:E_2^m]$$
$$-k^{+}_{e_8^m:APC}e_8^m[APC] + k^{-}_{e_8^m:APC}[APC:E_8^m]$$
$$-k^{+}_{e_8^m:e_9^m}e_9^me_8^m + k^{-}_{e_8^m:e_9^m}[TEN] - 0.005e_8^m$$
$$-k^{+}_{e_8^m:e_9^m}e_8^me_9^m + k^{-}_{e_8^m:e_9^m}[TEN] \quad (14)$$

$$\frac{d}{dt}z_9 = k_{flow}(z_9^{up} - z_9) - k_9^{on}p_9^{avail}z_9 + k_9^{off}z_9^m$$
$$-k^{+}_{z_9:e_7^m}z_9e_7^m + k^{-}_{z_9:e_7^m}[Z_9:E_7^m]$$
$$-k^{+}_{z_9:e_{11}^h}e_{11}^h + k^{-}_{z_9:e_{11}^h}[Z_9:E_{11}^h]$$
$$-k^{+}_{z_9:e_{11}1}z_9e_{11} + k^{-}_{z_9:e_{11}}[Z_9:E_{11}] \quad (15)$$

$$
\begin{aligned}
\frac{d}{dt}e_9 &= f_{flow}(e_9^{up} - e_9) && (16)\\
&-k_9^{on} p_9^{avail} e_9 + k_9^{off} e_9^m \\
&k^{cat}_{z_9:e_7^m}[Z_9 : E_7^m] \\
&\cancel{-k^{in}_{AT:e_9} e_9} - k^+_{z_7:e_9} z_7 e_9 \\
&+(k^{cat}_{z_7:e_9} + k^-_{z_7:e_9})[Z_7 : E_9] \\
&+(k^{cat}_{z_7^m:e_9} + k^-_{z_7^m:e_9})[Z_7^m : E_9] - k^+_{z_7^m:e_9} z_7^m e_9 \\
&-k_9^{on} p_9^{*,avail} e_9 + k_9^{off} e_9^{m*} \\
&-k_{diff}(e_9 - e_9^{ec}) \\
&+k^{cat}_{z_9:e_{11}^h}[Z_9 : E_{11}^h] + k^{cat}_{z_9:e_{11}}[Z_9 : E_{11}] \\
&-k^{AT}_{e_9} e_9[AT] \\
&-k^{ATH}_{e_9} e_9[AT : Hep]
\end{aligned}
$$

$$
\begin{aligned}
\frac{d}{dt}z_9^m &= k_9^{on} p_9^{avail} z_9 - k_9^{off} z_9^m && (17)\\
&-k^+_{z_9^m:e_{11}^{h,m}} z_9^m e_{11}^{h,m} + k^-_{z_9^m e_{11}^{h,m}}[Z_9^m : E_{11}^{h,m}] \\
&-k^+_{z_9^m:e_{11}^{m*}} z_9^m e_{11}^{m*} + k^-_{z_9^m e_{11}^{m*}}[Z_9^m : E_{11}^{m*}]
\end{aligned}
$$

$$
\begin{aligned}
\frac{d}{dt}e_9^m &= k_9^{on} p_9^{avail} e_9 - k_9^{off} e_9^m && (18)\\
&-k^+_{e_8^m:e_9^m} e_8^m e_9^m + k^-_{e_8^m:e_9^m}[TEN] \\
&+k^{cat}_{z_9^m:e_{11}^{h,m}}[Z_9^m : E_{11}^{h,m}] \\
&+k^{cat}_{z_9^m:e_{11}^{m*}}[Z_9^m : E_{11}^{m*}] \\
&-k^{AT}_{e_9^m} e_9^m[AT] \\
&-k^{ATH}_{e_9^m} e_9^m[AT : Hep]
\end{aligned}
$$

$$
\frac{d}{dt}z_2 = -k_2^{on} p_2^{avail} z_2 + k_2^{off} z_2^m + k_{flow}(z_2^{up} - z_2) \qquad (19)
$$

$$
\begin{aligned}
\frac{d}{dt}e_2 &= k_{flow}(e_2^{up} - e_2) && (20)\\
&-k_{2*}^{on} p_2^{*,avail} e_2 + k_{2*}^{off} e_2^m + k^{cat}_{z_2^m:PRO}[Z_2^m : PRO] \\
&\cancel{-k^{in}_{AT:e_2} e_2} - k_{z_5:e_2^p} z_5 e_2 + (k^{cat}_{z_5:e_2} + k^-_{z_5:e_2})[Z_5 : E_2] \\
&-k^+_{z_8:e_2} z_8 e_2 + (k^{cat}_{z_8:e_2} + k^-_{z_8:e_2})[Z_8 : E_2] \\
&-k^+_{z_7:e_2} z_7 e_2 + (k^{cat}_{z_7^m:e_2} + k^-_{z_7:e_2})[Z_7 : E_2] \\
&-k^+_{z_7^m e_2} z_7^m e_2 + (k^{cat}_{z_7^m:e_2} + k^-_{z_7^m:e_2})[Z_7^m : E_2] \\
&-k_{diff}(e_2 - e_2^{ec}) \\
&-k^+_{z_{11}:e_2} z_{11} + (k^-_{z_{11}:e_2} + k^{cat}_{z_{11}:e_2})[Z_{11} : E_2] \\
&-k^+_{e_{11}^h:e_2} e_{11}^h e_2 + (k^-_{e_{11}^h:e_2} + k_{e_{11}^h} : e_2)^{cat}[E_{11}^h : E_2] \\
&+k^{cat}_{z_2^m:PRO^h}[Z_2^m : PRO^h] - k^+_{e_5^h:e_2} e_2 e_5^h \\
&+k^-_{e_5^h:e_2}[E_5^h : E_2] + k^{cat}_{e_5^h:e_2}[E_5^h : E_2] \\
&-k^{AT}_{e_2} e_2[AT] \\
&-k^{ATH}_{e_2} e_2[AT : Hep]
\end{aligned}
$$

$$
\begin{aligned}
\frac{d}{dt} z_2^m &= k_2^{on} p_2^{avail} z_2 - k_2^{off} z_2^m \\
&\quad - k^+_{z_2^m:PRO} z_2^m [PRO] + k^-_{z_2^m:PRO} [Z_2^m : PRO] \\
&\quad - k^+_{z_2^m:PRO^h} z_2^m PRO^h + k^-_{z_2^m:PRO^h} [Z_2^m : PROh]
\end{aligned} \tag{21}
$$

$$
\begin{aligned}
\frac{d}{dt} e_2^m &= k_{2*}^{on} p_2^{avail} e_2 - k_{2*}^{off} e_2^m \\
&\quad + (k^{cat}_{z_5^m:e_2^m} + k^-_{z_5^m:e_2^m}) [Z_5^m : E_2^m] \\
&\quad - k^+_{z_5^m:e_2^m} z_5^m e_2^m \\
&\quad + (k^{cat}_{z_8^m:e_2^m} + k^-_{z_8^m:e_2^m}) [Z_8^m : E_2^m] - k^+_{z_8^m:E_2^m} z_8^m e_2^m \\
&\quad - k^+_{z_{11}^m:e_2^m} z_{11}^m e_2^m \\
&\quad + (k^-_{z_{11}^m:e_2^m} + k^{cat}_{z_{11}^m:e_2^m}) [Z_{11}^m : E_2^m] \\
&\quad - k^+_{e_{11}^{h,m*} e_2^m} e_{11}^{h,m*} e_2^m \\
&\quad + (k^-_{e_{11}^{h,m*} e_2^m} + k^{cat}_{e_{11}^{h,m*}:e_2^m}) [E_{11}^{hms} : E_2^m] \\
&\quad - k^+_{e_5^{hm}:e_2^m} e_2^m e_5^{hm} + k^-_{e_5^{hm}:e_2^m} [E_5^{hm} : E_2^m] \\
&\quad + k^{cat}_{e_5^{hm}:e_2^m} [E_5^{hm} : E_2^m] \\
&\quad - k^+_{PRO^h:e_2^m} PRO^h e_2^m + k^-_{PRO^h:e_2^m} [PRO^h : E_2^m] \\
&\quad + k^{cat}_{PRO^h:e_2^m} [PRO^h : E_2^m] \\
&\quad - k^{AT}_{e_2^m} e_2^m [AT] \\
&\quad - k^{ATH}_{e_2^m} e_2^m [AT : Hep]
\end{aligned} \tag{22}
$$

$$
\begin{aligned}
\frac{d}{dt} [TEN] &= -k^-_{e_8^m:e_9^m} [TEN] + k_{e_8^m:e_9^m} e_8^m e_9^m \\
&\quad + (k^{cat}_{z_{10}^m:TEN} + k^-_{z_{10}^m:TEN}) [Z_{10}^m : TEN] - k^+_{z_{10}^m:TEN} z_{10}^m [TEN]
\end{aligned} \tag{23}
$$

$$
\begin{aligned}
\frac{d}{dt} [PRO] &= -k^-_{e_5^m:e_{10}^m} [PRO] + k^+_{e_5^m:e_{10}^m} e_{10}^m e_5^m \\
&\quad + (k^{cat}_{z_2^m:PRO} + k^-_{z_2^m:PRO}) [Z_2^m : PRO] - k^+_{z_2^m:PRO} z_2^m [PRO] \\
&\quad + k^{cat}_{PRO^h:e_2^m} [PRO^h : E_2^m]
\end{aligned} \tag{24}
$$

$$
\begin{aligned}
\frac{d}{dt} [PL_a^s] &= k^+_{adh} p_{PLAS}^{avail} [PL] - k^-_{adh} [PL_a^s] \\
&\quad + k^+_{adh} [PL_a^v] * p_{PLAS}^{avail}
\end{aligned} \tag{25}
$$

$$
\begin{aligned}
\frac{d}{dt} [PL] &= k^p_{flow} ([PL]^{up} - [PL]) \\
&\quad - k^+_{adh} p_{PLAS}^{avail} + (k^{act}_{plt} ([PL_a^v] + [PL_a^s]) + k^{act}_{e2} \frac{e_2}{e_2 + 0.001}) [PL]
\end{aligned} \tag{26}
$$

$$
\begin{aligned}
\frac{d}{dt} [PL_a^v] &= k^-_{adh} [PL_a^s] - k^+_{adh} [PL_a^v] p_{PLAS}^{avail} \\
&\quad + (k^{act}_{plt} ([PL_a^v] + [PL_a^s]) + k^{act}_{e2} \frac{e_2}{e_2 + 0.001}) [PL]
\end{aligned} \tag{27}
$$

$$
\begin{aligned}
\frac{d}{dt} z_7^m &= k_7^{on} z_7 [TF]^{avail} - k_7^{off} z_7^m \\
&\quad - k^+_{z_7^m:e_{10}} z_7^m e_{10} - k^+_{z_7^m:e_2} z_7^m e_2 \\
&\quad + k^-_{z_7^m:e_{10}} [Z_7^m : E_{10}] + k^-_{z_7^m:e_2} [Z_7^m : E_2] \\
&\quad - k^+_{z_7^m:e_9} z_7^m e_9 + k^-_{z_7^m:e_9} [Z_7^m : E_9] \\
&\quad - z_7^m \frac{d}{dt} [PL_a^s] \frac{1}{p_{PLAS}^{avail}}
\end{aligned} \tag{28}
$$

$$\frac{d}{dt}e_7^m = k_7^{on}e_7[TF]^{avail} - k_7^{off}e_7^m \tag{29}$$
$$k^+_{TFPI:e_{10}:e_7^m}e_7^m[TFPI:E_{10}] + k^-_{TFPI:e_{10}:e_7^m}[TFPI:E_7^m]$$
$$+k^{cat}_{z_7^m:e_{10}}[Z_7^m:E_{10}] + k^{cat}_{z_7^m:e_2}[Z_7^m:E_2]$$
$$+(k^{cat}_{z_{10}:e_7^m} + k^-_{z_{10}:e_7^m})[Z_{10}:E_7^m]$$
$$-k^+_{z_{10}:e_7^m}e_7^m z_{10} - k^+_{z_9:e_7^m}e_7^m z_9$$
$$+(k^{cat}_{z_9:e_7^m} + k^-_{z_9:e_7^m})[Z_9:E_7^m]$$
$$+k^{cat}_{z_7^m:e_9}[Z_7^m:E_9] - e_7^m\frac{d}{dt}[PL_a^s]\frac{1}{p^{avail}_{PLAS}}$$

$$\frac{d}{dt}[TFPI] = -k^+_{TFPI:e_{10}}e_{10}[TFPI] + k^-_{TFPI:e_{10}}[TFPI:E_{10}] \tag{30}$$
$$k_{flow}([TFPI]^{up} - [TFPI])$$
$$-k^+_{TFPI:e_5^{hm}}e_5^{hm}[TFPI] + k^-_{TFPI:e_5^{hm}}[TFPI:E_5^{hm}]$$
$$-k^+_{TFPI:e_5^h}e_5^h[TFPI] + k^-_{TFPI:e_5^h}[TFPI:E_5^h]$$
$$-k^+_{TFPI:e_{10}^m}e_{10}^m[TFPI] + k^-_{TFPI:e_{10}^m}[TFPI:E_{10}^m]$$
$$-k^+_{TFPI:PRO^h:v_{10}}PRO^h[TFPI]$$
$$+k^-_{TFPI:PRO^h:v_{10}}[TFPI:PRO^h]^{v10}$$
$$-k^+_{TFPI:PRO^h:v_5}PRO^h[TFPI]$$
$$+k^-_{TFPI:PRO^h:v_5}[TFPI:PRO^h]^{v5}$$

$$\frac{d}{dt}[TFPI:E_{10}] = k^+_{TFPI:e_{10}}e_{10}[TFPI] - k^-_{TFPI:e_{10}}[TFPI:E_{10}] \tag{31}$$
$$+k^-_{TFPI:e_{10}:e_7^m}[TFPI:E_{10}:E_7^m]$$
$$-k^+_{TFPI:e_{10}:e_7^m}e_7^m[TFPI:E_{10}] + k_{flow}([TFPI:E_{10}]^{up} - [TFPI:E_{10}])$$
$$-k^+_{TFPI:e_{10}:e_5^h}[TFPI:E_{10}]e_5^h$$
$$+k^-_{TFPI:e_{10}:e_5^h}[E_{10}:TFPI:E_5^h]$$
$$-k_{10}^{on}[TFPI:E_{10}]p_{10}^{avail} + k_{10}^{off}[TFPI:E_{10}^m]$$

$$\frac{d}{dt}[TFPI:E_{10}:E_7^m] = -k^-_{TFPI:e_{10}:e_7^m}[TFPI:E_{10}:E_7^m] + k^+_{TFPI:e_{10}:e_7^m}e_7^m[TFPI:E_{10}] \tag{32}$$
$$-[TFPI:E_{10}:E_7^m]\frac{d}{dt}[PL_a^s]\frac{1}{p^{avail}_{PLAS}}$$

$$\frac{d}{dt}[APC] = (k^{cat}_{e_5^m:APC} + k^-_{e_5^m:APC})[APC:E_5^m] - k^{cat}_{e_5^m:APC}e_5^m \tag{33}$$
$$+(k^{cat}_{e_8^m:APC} + k^-_{e_8^m:APC})[APC:E_8^m] - k^+_{e_8^m:APC}e_8^m[APC]$$
$$+k_{flow}([APC]^{up} - [APC]) - k_{diff}([APC] - [APC^{ec}])$$
$$+(k^{cat}_{e_5:APC} + k^-_{e_5:APC})[APC:E_5] - k^+_{e_5:APC}e_5[APC]$$
$$+(k^{cat}_{e_8:APC} + k^-_{e_8:APC})[APC:E_8] - k^+_{e_8:APC}e_8[APC]$$
$$-k^+_{e_5^{hm}:APC}e_5^{hm}APC + k^-_{e_5^{hm}:APC}[APC:E_5^{hm}]$$
$$+k^{cat}_{e_5^{hm}:APC}[APC:E_5^{hm}] - k^+_{e_5^h:APC}e_5^h APC$$
$$+k^-_{e_5^h:APC}[APC:E_5^h] + k^{cat}_{e_5^h:APC}[APC:E_5^h]$$

$$\frac{d}{dt}[Z_7:E_2] = k_{flow}([Z_7:E_2]^{up} - [Z_7:E_2]) + k^{+}_{z_7:e_2} e_2 z_7 \tag{34}$$
$$-(k^{cat}_{z_7:e_2} + k^{-}_{z_7:e_2})[Z_7:E_2]$$

$$\frac{d}{dt}[Z_7:E_{10}] = k^{+}_{z_7:e_{10}} e_{10} z_7 - (k^{cat}_{z_7:e_{10}} + k^{-}_{z_7:e_{10}})[Z_7:E_{10}] \tag{35}$$
$$+k_{flow}([Z_7:E_{10}]^{up} - [Z_7:E_{10}])$$

$$\frac{d}{dt}[Z_7^m:E_{10}] = k^{+}_{z_7^m:e_{10}} e_{10} z_7^m \tag{36}$$
$$-(k^{cat}_{z_7^m:e_{10}} + k^{-}_{z_7^m:e_{10}})[Z_7^m:E_{10}] - [Z_7^m:E_{10}]\frac{d}{dt}[PL_a^s]\frac{1}{p^{avail}_{PLAS}}$$

$$\frac{d}{dt}[Z_7^m:E_2] = k^{+}_{z_7^m:e_2} e_2 z_7^m - (k^{cat}_{z_7^m:e_2} + k^{-}_{z_7^m:e_2})[Z_7^m:E_2] \tag{37}$$
$$-[Z_7^m:E_2]\frac{d}{dt}[PL_a^s]\frac{1}{p^{avail}_{PLAS}}$$

$$\frac{d}{dt}[Z_{10}:E_7^m] = k^{+}_{z_{10}:e_7^m} e_7^m z_{10} - (k^{cat}_{z_{10}:e_7^m} + k^{-}_{z_{10}:e_7^m})[Z_{10}:E_7^m] \tag{38}$$
$$-[Z_{10}:E_7^m]\frac{d}{dt}[PL_a^s]\frac{1}{p^{avail}_{PLAS}}$$

$$\frac{d}{dt}[Z_{10}^m:TEN] = k^{+}_{z_{10}^m:TEN} z_{10}^m[TEN] - (k^{cat}_{z_{10}^m:TEN} + k^{-}_{z_{10}^m:TEN})[Z_{10^m}:TEN] \tag{39}$$

$$\frac{d}{dt}[Z_5:E_2] = k^{+}_{z_5:e_2} e_2 z_5 - (k^{cat}_{z_5:e_2} + k^{-}_{z_5:e_2})[Z_5:E_2] \tag{40}$$
$$+k_{flow}([Z_5:E_2]^{up} - [Z_5:E_2])$$

$$\frac{d}{dt}[Z_5^m:e_{10}^m] = k^{+}_{z_5^m:e_{10}^m} e_{10}^m z_5^m - (k^{cat}_{z_5^m:e_{10}} + k^{-}_{z_5^m:e_{10}^m})[Z_5^m:E_{10}^m] \tag{41}$$

$$\frac{d}{dt}[Z_5^m:E_2^m] = k^{+}_{z_5^m:e_2^m} e_2^m z_5^m - (k^{cat}_{z_5^m:e_2^m} + k^{-}_{z_5^m:e_2^m})[Z_5^m:E_2^m] \tag{42}$$

$$\frac{d}{dt}[Z_8^m:E_{10}^m] = k^{+}_{z_8^m:e_{10}^m} e_{10}^m z_8^m - (k^{cat}_{z_8^m:e_{10}^m} + k^{-}_{z_8^m:e_{10}^m})[Z_8^m:E_{10}^m] \tag{43}$$

$$\frac{d}{dt}[Z_8^m:E_2^m] = k^{+}_{z_8^m:e_2^m} e_2^m z_8^m - (k^{cat}_{z_8^m:e_2^m} + k^{-}_{z_8^m:e_2^m})[Z_8^m:E_2^m] \tag{44}$$

$$\frac{d}{dt}[Z_8:E_2] = k^{+}_{z_8:e_2} e_2 z_8 - (k^{cat}_{z_8:e_2} + k^{-}_{z_8:e_2})[Z_8:E_2] \tag{45}$$
$$k_{flow}([Z_8:E_2]^{up} - [Z_8:E_2])$$

$$\frac{d}{dt}[APC:E_8^m] = k^{+}_{e_8^m:APC} e_8^m[APC] - (k^{cat}_{e_8^m:APC} + k^{-}_{e_8^m:APC})[APC:E_8^m] \tag{46}$$

$$\frac{d}{dt}[Z_9:E_7^m] = k^{+}_{z_9:e_7^m} e_7^m z_9 - (k^{cat}_{z_9:e_7^m} + k^{-}_{z_9:e_7^m})[Z_9:E_7^m] \tag{47}$$
$$-[Z_9:E_7^m]\frac{d}{dt}[PL_a^s]\frac{1}{p^{avail}_{PLAS}}$$

$$\frac{d}{dt}[Z_2^m:PRO] = k^{+}_{z_2^m:PRO} z_2^m[PRO] - (k^{cat}_{z_2^m:PRO} + k^{-}_{z_2^m:PRO})[Z_2^m:PRO] \tag{48}$$

$$\frac{d}{dt}[APC:E_5^m] = k^{+}_{e_5^m:APC} e_5^m[APC] - (k^{cat}_{e_5^m:APC} + k^{-}_{e_5^m:APC})[APC:E_5^m] \tag{49}$$

$$\frac{d}{dt}[TF] = -[TF]\frac{d}{dt}[PL_a^s]\frac{1}{p^{avail}_{PLAS}} \tag{50}$$

$$\frac{d}{dt}[Z_7:E_9] = k^{+}_{z_7:e_9} e_9 z_7 - (k^{cat}_{z_7:e_9} + k^{-}_{z_7:e_9})[Z_7:E_9] \tag{51}$$

$$\frac{d}{dt}[Z_7^m:E_9] = k^{+}_{z_7^m:e_9} e_9 z_7^m - (k^{cat}_{z_7^m:e_9} + k^{-}_{z_7^m:e_9})[Z_7^m:E_9] \tag{52}$$
$$-[Z_7^m:E_9]\frac{d}{dt}[PL_a^s]\frac{1}{p^{avail}_{PLAS}}$$

$$\begin{aligned}\frac{d}{dt}e_9^{m*} = {} & k_9^{on} p_9^{*,avail} e_9 - k_9^{off} e_9^{m*} + k^-_{e_8^m:e_9^m}[TEN^*] \\ & -k^+_{e_8^m:e_9^m} e_8^m e_9^{m*} \\ & -k^{AT}_{e_9^m} e_9^{m*}[AT] \\ & -k^{ATH}_{E_9^m} e_9^{m*}[AT:Hep]\end{aligned} \tag{53}$$

$$\begin{aligned}\frac{d}{dt}[TEN^*] = {} & -k^-_{e_8^m:e_9^m}[TEN^*] + k^+_{e_8^m:e_9^m} e_8^m e_9^{m*} \\ & +(k^{cat}_{z_{10}^m:TEN} + k^-_{z_{10}^m:TEN})[Z_{10}^m : TEN^*] \\ & k^+_{z_{10}^m:TEN}[TEN^*] z_{10}^m\end{aligned} \tag{54}$$

$$\frac{d}{dt}[Z_{10}^m : TEN^*] = k^+_{z_{10}^m:TEN}[TEN^*] z_{10}^m - (k^{cat}_{z_{10}^m:TEN} + k_{z_{10}^m} : TEN)^-[Z_{10}^m : TEN^*] \tag{55}$$

$$\begin{aligned}\frac{d}{dt}e_2^{ec} = {} & k_{diff}(e_2 - e_2^{ec}) + k_{flow}(e_2^{ec,up} - e_2^{ec}) \\ & \cancel{-k^{in}_{AT:e_2} e_2^{ec}} \\ & -k^{on}_{TM} e_2^{ec}[TM]^{avail} + k^{off}_{TM}[TM : E_2^{ec}] - k^{AT}_{e_2^{ec}} e_2^{ec}[AT]\end{aligned} \tag{56}$$

$$\begin{aligned}\frac{d}{dt}[APC^{ec}] = {} & k_{flow}([APC]^{up} - [APC^{ec}]) + k_{diff}([APC] - [APC^{ec}]) \\ & +k^{cat}_{PC:TM:e_2}[TM : E_2^{ec} : APC]\end{aligned} \tag{57}$$

$$\begin{aligned}\frac{d}{dt}[TM : E_2^{ec}] = {} & k^+_{TM}[E_2^{ec}](1 - [TM : E_2^{ec}] - [TM : E_2^{ec} : APC]) \\ & -k^-_{TM}[TM : E_2^{ec}] - k^+_{PC:TM:e_2}[TM : E_2^{ec}] \\ & +(k^-_{PC:TM:e_2} + k^{cat}_{PC:TM:e_2})[TM : E_2^{ec} : APC]\end{aligned} \tag{58}$$

$$\begin{aligned}\frac{d}{dt}[TM : E_2^{ec} : APC] = {} & k^+_{PC:TM:e_2}[TM : E_2^{ec}] \\ & -(k^-_{PC:TM:e_2} + k^{cat}_{PC:TM:e_2})[TM : E_2^{ec} : APC]\end{aligned} \tag{59}$$

$$\begin{aligned}\frac{d}{dt}e_9^{ec} = {} & k_{diff}(e_9 - e_9^{ec}) + k_{flow}(e_9^{up} - e_9^{ec}) \\ & \cancel{-k^{in}_{AT:e_9} e_9^{ec}} - k^{AT}_{e_9^{ec}} e_9^{ec}[AT]\end{aligned} \tag{60}$$

$$\begin{aligned}\frac{d}{dt}e_{10}^{ec} = {} & k_{diff}(e_{10} - e_{10}^{ec}) + k_{flow}(e_{10}^{up} - e_{10}^{ec}) \\ & \cancel{-k^{in}_{AT:e_{10}} e_{10}^{ec}} - k^{AT}_{e_{10}^{ec}} e_{10}^{ec}[AT]\end{aligned} \tag{61}$$

$$\frac{d}{dt}[APC : E_5] = -(k^{cat}_{e_5:APC} + k^-_{e_5:APC})[APC : E_5] + k^+_{e_5:APC} e_5 [APC] \tag{62}$$

$$\frac{d}{dt}[APC : E_8] = -(k^{cat}_{e_8:APC} + k^-_{e_8:APC})[APC : E_8] + k^+_{e_8:APC} e_8 [APC] \tag{63}$$

$$\begin{aligned}\frac{d}{dt}z_{11} = {} & k_{flow}(z_{11}^{up} - z11) - k^{on}_{z_{11}} z_{11} p_{11}^{avail} \\ & k^{off}_{z_{11}} z_{11}^m - k^+_{z_{11}:e_{11}^h} z_{11} e_{11}^h \\ & k^-_{z_{11}:e_{11}^h}[Z_{11} : E_{11}^h] - k^+_{z_{11}:e_{11}} z_{11} e_{11} \\ & k^-_{z_{11}:e_{11}}[Z_{11} : E_{11}] - k^+_{z_{11}:e_2} z_{11} e_2 \\ & k^-_{z_{11}:e_2}[Z_{11} : E_2]\end{aligned} \tag{64}$$

$$\frac{d}{dt}e_{11} = k_{flow}(e_{11}^{up} - e_{11}) \tag{65}$$
$$-k_{e_{11}^{on,s}}e_{11}p_{111}^{avail} + k_{e_{11}^{off,s}}e_{11}^{m*}$$
$$-k_{z_9:e_{11}}^{+}z_9e_{11} + (k_{z_9:e_{11}}^{-} + k_{z_9:e_{11}}^{cat})[Z_9 : E_{11}]$$
$$-k_{z_{11}:e_{11}}^{+}z_{11}e_{11} + (k_{z_{11}:e_{11}}^{-} + k_{z_{11}:e_{11}}^{cat})[Z_{11} : E_{11}]$$
$$+k_{e_{11}^h:e_{11}^h}^{cat}[E_{11}^h : E_{11}^h] - k_{e_{11}^h:e_{11}}^{+}e_{11}^h e_{11}$$
$$+(k_{e_{11}^h:e_{11}}^{-} + 2 * k_{e_{11}^h:e_{11}}^{cat})[E_{11}^h : E_{11}]$$
$$+k_{e_{11}^h:e_2}^{cat}[E_{11}^h : E_2]$$
$$-k_{e_{11}}^{AT}e_{11}[AT]$$
$$-k_{e_{11}}^{ATH}e_{11}[AT : Hep]$$
$$\frac{d}{dt}z_{11}^m = k_{z_{11}}^{on}z_{11}p_{11}^{avail} - k_{z_{11}}^{off}z_{11}^m \tag{66}$$
$$-k_{z_{11}^m:e_{11}^{h,m}}^{+}z_{11}^m e_{11}^{h,m} + k_{z_{11}^m:e_{11}^{h,m}}^{-}[Z_{11}^m : E_{11\,hm}]$$
$$-k_{z_{11}^m:e_{11}^{m*}}^{+}z_{11}^m e_{11}^{m*} + k_{z_{11}^m:e_{11}^{m*}}^{-}[Z_{11}^m : E_{11}^{m*}]$$
$$-k_{z_{11}^m:E_2^m}^{+}z_{11}^m e_2^m + k_{z_{11}^m:e_2^m}^{-}[Z_{11}^m : E_2^m]$$
$$\frac{d}{dt}e_{11}^{m*} = k_{e_{11}}^{on*}e_{11}p_{111}^{avail} - k_{e_{11}}^{off*}e_{11}^{m*} \tag{67}$$
$$-k_{z_9^m:e_{11}^{m*}}^{+}e_{11}^{m*}z_9^m$$
$$+(k_{z_9^m:e_{11}^{m*}}^{-} + k_{z_9^m:e_{11}^{m*}}^{cat})[Z_9^m : E_{11}^{m*}]$$
$$-k_{z_{11}^m:e_{11}^{m*}}^{+}z_{11}^m e_{11}^{m*}$$
$$+(k_{z_{11}^m:e_{11}^{m*}}^{-} + k_{z_{11}^m:e_{11}^{m*}}^{cat})[Z_{11}^m : E_{11}^{m*}]$$
$$+k_{e_{11}^{h,m*}:e_{11}^{m*}}^{+}e_{11}^{h,m*}e_{11}^{m*}$$
$$+(k_{e_{11}^{h,m*}:e_{11}^{m*}}^{-} + 2 * k_{e_{11}^{h,m*}:e_{11}^{m*}}^{cat})[E_{11}^{hms} : E_{11}^{h,m}]$$
$$+k_{e_{11}^{h,m*}:e_2^m}^{cat}[E_{11}^{hms} : E_2^m]$$
$$+k_{e_{11}}^{AT}e_{11}^{m*}[AT]$$
$$-k_{e_{11}}^{ATH}e_{11}^{m*}[AT : Hep]$$
$$\frac{d}{dt}[Z_{11}^m : E_2^m] = k_{z_{11}^m:e_2^m}^{+}z_{11}^m e_2^m - (k_{z_{11}^m:e_2^m}^{-} + k_{z_{11}^m:e_2^m}^{cat})[Z_{11}^m : E_2^m] \tag{68}$$
$$\frac{d}{dt}[Z_9^m : E_{11}^{m*}] = k_{z_9^m:e_{11}^{m*}}^{+}z_9^m e_{11}^m s \tag{69}$$
$$-(k_{z_9^m:e_{11}^{m*}}^{-} + k_{z_9^m:e_{11}^{m*}}^{cat})[Z_9^m : E_{11}^{m*}]$$
$$\frac{d}{dt}[Z_{11} : E_2] = k_{flow}([Z_{11} : E_2]^{up} - [Z_{11} : E_2]) + k_{z_{11}:e_2}^{+}z_{11}e_2 \tag{70}$$
$$-(k_{z_{11}:e_2}^{-} + k_{z_{11}:e_2}^{cat})[Z_{11} : E_2]$$
$$\frac{d}{dt}[Z_9 : E_{11}] = k_{flow}([Z_9 : E_{11}]^{up} - [Z_9 : E_{11}]) + k_{z_9:e_{11}}^{+}z_9e_{11} \tag{71}$$
$$-(k_{z_9:E_{11}}^{-} + k_{z_9:e_{11}}^{cat})[Z_9 : E_{11}]$$
$$\frac{d}{dt}[Z_{11} : E_{11}] = k_{flow}([Z_{11} : E_{11}]^{up} - [Z_{11} : E_{11}]) + k_{z_{11}:e_{11}}^{+}z_{11}e_{11} \tag{72}$$
$$-(k_{z_{11}:e_{11}}^{-} + k_{z_{11}:e_{11}}^{cat})[Z_{11} : E_{11}]$$

$$
\begin{aligned}
\frac{d}{dt}e^{h}_{11} &= k^{on*}_{e^h_{11}} e^h_{11} p^{avail}_{111} + k^{off*}_{e^h_{11}} e^{h,m*}_{11} \\
&\quad -k^{on}_{e^h_{11}} e^h_{11} p^{avail}_{11} + k^{off}_{e^h_{11}} e^{h,m}_{11} \\
&\quad -k^{+}_{z_9:e^h_{11}} z_9 e^h_{11} + (k^{-}_{z_9:e^{h,m}_{11}} + k^{cat}_{z_9:e^h_{11}})[Z_9 : E^h_{11}] \\
&\quad -k_{z_{11}:e^{hp}_{11}} z_{11} e^h_{11} + (k^{-}_{z_{11}:e^h_{11}m} + 2 * k^{cat}_{z_{11}:e^h_{11}})[Z_{11:E^h_{11}}] \\
&\quad +k^{cat}_{z_{11}:e_{11}}[Z_{11} : E_{11}] + k^{cat}_{z_{11}:e_2}[Z_{11} : E_2] \\
&\quad -2 * k^{+}_{e^h_{11}:e^h_{11}} e^h_{11} e^h_{11} \\
&\quad +(2 * k^{-}_{e^h_{11}:e^h_{11}} + k^{cat}_{e^g_{11}:e^h_{11}})[E^h_{11} : E^h_{11}] \\
&\quad -k^{+}_{e^h_{11}:e_{11}} e^h_{11} e_{11} + k^{-}_{e^h_{11}:e_{11}}[E^h_{11} : E_{11}] \\
&\quad -k^{+}_{e^h_{11}:e_2} e^h_{11} e_2 + k^{-}_{e^h_{11}:e_2}[E^h_{11} : E_2] \\
&\quad +k_{flow}(e^{h,up}_{11} - e^h_{11}) \\
&\quad -k^{AT}_{e^h_{11}} e^h_{11}[AT] \\
&\quad -k^{ATH}_{e_{11}} e^h_{11}[AT : Hep]
\end{aligned} \tag{73}
$$

$$
\begin{aligned}
\frac{d}{dt}e^{h,m}_{11} &= k^{on}_{e^h_{11}} e^h_{11} p^{avail}_{11} - k^{off}_{e^h_{11}} e^{h,m}_{11} \\
&\quad -k^{+}_{z^m_9:e^{h,m}_{11}} z^m_9 e^{h,m}_{11} \\
&\quad +(k^{-}_{z^m_9:e^{h,m}_{11}} + k^{cat}_{z^m_9:e^{h,m}_{11}})[Z^m_9 : E^{h,m}_{11}] \\
&\quad -k^{+}_{z^m_{11}:e^{h,m}_{11}} z^m_{11} e^{h,m}_{11} \\
&\quad +(k^{-}_{z^m_{11}:e^{h,m}_{11}} + 2 * k^{cat}_{z^m_{11}:e^{h,m}_{11}})[Z^m_{11} : E^{h,m}_{11}] \\
&\quad +k^{cat}_{z^m_{11}:e^{m*}_{11}}[Z^m_{11} : E^{m*}_{11}] + k^{cat}_{z^m_{11}:e^m_2}[Z^m_{11} : E^m_2] \\
&\quad -k^{+}_{e^{h,m*}_{11}:e^{h,m}_{11}} e^{h,m*}_{11} e^{h,m}_{11} \\
&\quad +(k^{-}_{e^{h,m*}_{11}:e^{h,m}_{11}} + k^{cat}_{e^{h,m*}_{11}:e^{h,m}_{11}})[E^{hms}_{11} : E^{h,m}_{11}] \\
&\quad -k^{AT}_{e_{11}} e^{hm}_{11}[AT] \\
&\quad -k^{ATH}_{e_{11}} e^{hm}_{11}[AT : Hep]
\end{aligned} \tag{74}
$$

$$
\begin{aligned}
\frac{d}{dt}e^{h,m*}_{11} &= k^{on*}_{e^h_{11}} e^h_{11} p^{avail}_{111} - k^{off*}_{e^h_{11}} e^{h,m*}_{11} \\
&\quad -k^{+}_{e^{h,m*}_{11}:e^{h,m}_{11}} e^{h,m*}_{11} e^{h,m}_{11} + k^{-}_{e^{h,m*}_{11}:e^{h,m}_{11}}[E^{hms}_{11} : E^{h,m}_{11}] \\
&\quad -k^{+}_{e^{h,m*}_{11}:e^{m*}_{11}} e^{h,m*}_{11} e^{m*}_{11} + k^{-}_{e^{h,m*}_{11}:e^{m}_{11}s}[E^{hms}_{11} : E^{m*}_{11}] \\
&\quad -k^{+}_{e^{h,m*}_{11}:e^m_2} e^{h,m*}_{11} e^m_2 + k^{-}_{e^{h,m*}_{11}:e^m_2}[E^{hms}_{11} : E^m_2]
\end{aligned} \tag{75}
$$

$$
\begin{aligned}
\frac{d}{dt}[Z_9 : E^h_{11}] &= k_{flow}([Z_9 : E^h_{11}]^{up} - [Z_9 : E^h_{11}]) \\
&\quad +k^{+}_{z_9:e^h_{11}} z_9 e^h_{11} - (k^{-}_{z_9:e^h_{11}} + k^{cat}_{z_9:e^h_{11}})[Z_9 : E^h_{11}]
\end{aligned} \tag{76}
$$

$$
\begin{aligned}
\frac{d}{dt}[Z^m_9 : E^{h,m}_{11}] &= k^{+}_{z^m_9:e^{h,m}_{11}} z^m_9 e^{h,m}_{11} \\
&\quad -(k^{-}_{z^m_9:e^h_{11}m} + k^{cat}_{z^m_9:e^h_{11}m})[Z^m_9 : E^{h,m}_{11}]
\end{aligned} \tag{77}
$$

$$\frac{d}{dt}[Z_{11}:E^h_{11}] = k_{flow}([Z_{11}:E^h_{11}]^{up} - [Z_{11}:E^h_{11}]) + k_{z_{11}:e^{hp}_{11}} z_{11} e^h_{11} - (k^-_{z_{11}:e^h_{11}} + k^{cat}_{z_{11}:e^h_{11}})[Z_{11}:E^h_{11}] \tag{78}$$

$$\frac{d}{dt}[E^h_{11}:E^h_{11}] = k_{flow}([E^h_{11}:E^h_{11}]^{up} - [E^h_{11}:E^h_{11}]) + k^+_{e^h_{11}:e^h_{11}} e^h_{11} e^h_{11} - (k^-_{e^h_{11}:e^h_{11}} + k^{cat}_{e^h_{11}:e^h_{11}})[E^h_{11}:E^h_{11}] \tag{79}$$

$$\frac{d}{dt}[E^h_{11}:E_{11}] = k_{flow}([E^h_{11}:E_{11}]^{up} - [E^h_{11}:E_{11}]) + k^+_{e^h_{11}:e_{11}} e^h_{11} - (k^-_{e^h_{11}:e_{11}} + k^{cat}_{e^h_{11}:e_{11}})[E^h_{11}:E_{11}] \tag{80}$$

$$\frac{d}{dt}[E^h_{11}:E_2] = k_{flow}([E^h_{11}:E_2]^{up} - [E^h_{11}:E_2]) + k^+_{e^h_{11}:e_2} e^h_{11} e_2 - (k^-_{e^h_{11}:e_2} + k^{cat}_{e^h_{11}:e_2})[E^h_{11}:E_2] \tag{81}$$

$$\frac{d}{dt}[Z^m_{11}:E^{h,m}_{11}] = k^+_{z^m_{11}:e^{h,m}_{11}} z^m_{11} e^{h,m}_{11} - (k^-_{z^m_{11}:e^{h,m}_{11}} + k^{cat}_{z^m_{11}:e^{h,m}_{11}})[Z^m_{11}:E^{h,m}_{11}] \tag{82}$$

$$\frac{d}{dt}[Z^m_{11}:E^{m*}_{11}] = k^+_{z^m_{11}:e^{m*}_{11}} z^m_{11} e^{m*}_{11} - (k^-_{z^m_{11}:e^{m*}_{11}} + k^{cat}_{z^m_{11}:e^m_{11}s})[Z^m_{11}:E^{m*}_{11}] \tag{83}$$

$$\frac{d}{dt}[E^{hms}_{11}:E^{h,m}_{11}] = k^+_{e^{h,m*}_{11}:e^{h,m}_{11}} e^{h,m}_{11} - (k^-_{e^{h,m*}_{11}:e^{h,m}_{11}} + k^{cat}_{e^{h,m*}_{11}:e^{h,m}_{11}})[E^{hms}_{11}:E^{h,m}_{11}] \tag{84}$$

$$\frac{d}{dt}[E^{hms}_{11}:E^{m*}_{11}] = k_{e^{h,m*}_{11}:e^{h,m*}_{11}} e^{m*}_{11} - (k^-_{e^{h,m*}_{11}:e^{m*}_{11}} + k^{cat}_{e^{h,m*}_{11}:e^{m*}_{11}})[E^{hms}_{11}:E^{m*}_{11}] \tag{85}$$

$$\frac{d}{dt}[E^{hms}_{11}:E^m_2] = k^+_{e^{h,m*}_{11}:e^m_2} e^{h,m*}_{11} e^m_2 - (k^-_{e^{hms}_{11}:e^m_2} + k^{cat}_{e^{h,m*}_{11}:e^m_2}[E^{hms}_{11}:E^m_2]) \tag{86}$$

$$\frac{d}{dt}e^{hm}_5 = +k^{cat}_{z^m_5:e^m_{10}}[Z^m_5:E^m_{10}] + k^{on}_5 e^h_5 p^{avail}_5 - k^{off}_5 e^{hm}_5 - k^+_{e^{hm}_5:e^m_{10}} e^m_{10} e^{hm}_5 + k^-_{e^{hm}_5:e^m_{10}} PRO^h - k^+_{e^{hm}_5:e^m_2} e^m_2 e^{hm}_5 + k^-_{e^{hm}_5:e^m_2}[E^{hm}_5:E^m_2] - k^+_{TFPI:e^{hm}_5} e^{hm}_5 TFPI + k^-_{TFPI:e^{hm}_5}[TFPI:E^{hm}_5] - k^+_{e^{hm}_5:APC} e^{hm}_5 APC + k^-_{e^{hm}_5:APC}[APC:E^{hm}_5] - k^+_{TFPI:e_{10}:e^{hm}_5}[TFPI:E^m_{10}]e^{hm}_h + k^-_{TFPI:e_{10}:e^{hm}_5}[E^m_{10}:TFPI:E^{hm}_5] - k^+_{TFPI:e_{10}:e^{hm}_5:e^m_{10}}[TFPI:E^m_{10}]e^{hm}_5 + k^-_{TFPI:e_{10}:e^{hm}_5:e^m_{10}}[TFPI:PRO^h_{v10}] \tag{87}$$

$$
\begin{aligned}
\frac{d}{dt}e_5^h &= -k_{5on}e_5^h p_5^{avail} + k_5^{off}e_5^{hm} \\
&+k_{flow}(e_5^{up} - e_5^h) + (1 - f_5)N_5 dpl \cdot p \\
&-k^+_{e_5^h:e_2}e_5^h + k^-_{e_5^h:e_2}[E_5^h : E_2] \\
&-k^+_{e_5^h:APC}APC \cdot e_5^h + k^-_{e_5^h:APC}[APC : E_5^h] \\
&-k^+_{TFPI:e_5^h}e_5^h TFPI \\
&+k^-_{TFPI:e_5^h}[TFPI : E_5^h] \\
&-k^+_{TFPI:e_{10}:e_5^h}[TFPI : E_{10}]e_5^h \\
&+k^-_{TFPI:e_{10}:e_5^h}[E_{10} : TFPI : E_5^h] \\
&-k^+_{TFPI:e_{10}^m:e_5^h}[TFPI : E_{10}^m]e_5^h \\
&+k^-_{TFPI:e_{10}^m:e_5^h}[E_{10}^m : TFPI : E_5^h]
\end{aligned} \tag{88}
$$

$$
\begin{aligned}
\frac{d}{dt}PRO^h &= +k^+_{e_5^{hm}:e_{10}^m}e_{10}^m e_5^{hm} - k^-_{e_5^{hm}:e_{10}^m}PRO^h \\
&-k^+_{z_2^m:PRO^h}PRO^h z_2^m + k^-_{z_2^m:PRO^h}[Z_2^m : PRO^h] \\
&+k^{cat}_{z_2^m:PRO^h}[Z_2^m : PRO^h] \\
&-k^+_{TFPI:PRO^h:v_{10}}PRO^h TFPI + k^-_{TFPI:PRO^h:v_{10}}[TFPI : PRO^h_{v10}] \\
&-k^+_{TFPI:PRO^h:v_5}PRO^h TFPI + k^-_{TFPI:PRO^h:v_5}[TFPI : PRO^h_{v5}] \\
&-k^+_{PRO^h:e_2^m}PRO^h e_2^m + k^-_{PRO^h:e_2^m}[PRO^h : E_2^m]
\end{aligned} \tag{89}
$$

$$
\begin{aligned}
\frac{d}{dt}[Z_2^m : PRO^h] &= +k^+_{z_2^m:PRO^h}PRO^h z_2^m - k^-_{z_2^m:PRO^h}[Z_2^m : PRO^h] \\
&-k^{cat}_{z_2^m:PRO^h}[Z_2^m PRO^h]
\end{aligned} \tag{90}
$$

$$
\begin{aligned}
\frac{d}{dt}[E_5^{hm} : E_2^m] &= +k^+_{e_5^{hm}:e_2^m}e_2^m e_5^{hm} - k^-_{e_5^{hm}:e_2^m}[E_5^{hm} : E_2^m] \\
&-k^{cat}_{e_5^{hm}:e_2^m}[E_5^{hm} : E_2^m]
\end{aligned} \tag{91}
$$

$$
\begin{aligned}
\frac{d}{dt}[E_5^h : E_2] &= +k^+_{e_5^h:e_2}e_2 e_5^h - k^-_{TFPI:e_5^h}[E_5^h : E_2] \\
&-k^{cat}_{e_5^h:e_2}[E_5^h : E_2] + k_{flow}([E_5^h : E_2]^{up} - [E_5^h : E_2])
\end{aligned} \tag{92}
$$

$$
\begin{aligned}
\frac{d}{dt}[TFPI : E_5^{hm}] &= +k^+_{TFPI:e_5^{hm}}e_5^{hm}TFPI - k^-_{TFPI:e_5^{hm}}[TFPI : E_5^{hm}] \\
&-k^+_{TFPI:e_5^{hm}:e_{10}m}[TFPI : E_5^{hm}]e_{10}^m \\
&+k^-_{TFPI:e_5^{hm}:e_{10}m}[E_{10}^m : TFPI : E_5^{hm}] \\
&+k_5^{on}[TFPI : E_5^h]p_5^{avail} - k_5^{off}[TFPI : E_5^{hm}] \\
&-k^+_{TFPI:e_{10}:e_5^{hm}}[TFPI : E_5^{hm}]e_{10}^m \\
&+k^-_{TFPI:e_{10}:e_5^{hm}}[TFPI : PRO^h_{v5}] \\
&-k^+_{TFPI:e_5^{hm}:e_{10}}[TFPI : E_5^{hm}]e_{10} \\
&+k^-_{TFPI:e_5^{hm}:e_{10}}[E_{10} : TFPI : E_5^{hm}]
\end{aligned} \tag{93}
$$

$$
\begin{aligned}
\frac{d}{dt}[APC : E_5^{hm}] &= +k^+_{e_5^{hm}:APC}E_5^{hm}APC - k^-_{e_5^{hm}:APC}[APC : E_5^{hm}] \\
&+k^{cat}_{e_5^{hm}:APC}[APC : E_5 hm]
\end{aligned} \tag{94}
$$

$$\frac{d}{dt}[APC:E_5^h] = +k^+_{e_5^h:APC} e_5^h APC - k^-_{e_5^{hm}:APC}[APC:E_5^h] - k^{cat}_{e_5^h:APC}[APC:E_5^h] + k_{flow}([APC:E_5^h]^{up} - [APC:E_5^h]) \quad (95)$$

$$\frac{d}{dt}[TFPI:E_5^h] = +k_{TFPI:e_5^h up} e_5^h TFPI - k^-_{kTFPI:e_5^h}[TFPI:E_5^h] + k_{flow}([TFPI:E_5^h]^{up} - [TFPI:E_5^h]) - k^+_{TFPI:e_5^h:e_{10}}[TFPI:E_5^h]e_{10} + k^-_{TFPI:e_5^h:e_{10}}[E_{10}:TFPI:E_5^h] + k_5^{on}[TFPI:E_5^h]p_5^{avail} + k_5^{off}[TFPI:E_5^{hm}] \quad (96)$$

$$\frac{d}{dt}[TFPI:E_{10}^m] = +k^+_{TFPI:e_{10}^m} e_{10}^m TFPI - k^-_{TFPI:e_{10}^m}[TFPI:E_{10}^m] - k^+_{TFPI:e_{10}:e_5^h m}[TFPI:E_{10}^m]e_5^{hm} + k^-_{TFPI:e_{10}:e_5^{hm}}[E_{10}^m:TFPI:E_5^{hm}] + k_{10}^{on}[TFPI:E_{10}]p_{10}^{avail} - k_{10}^{off}[TFPI:E_{10}^m] - k^+_{TFPI:e_{10}^m:e_5^{hm}}[TFPI:E_{10}^m]e_5^{hm} + k^-_{TFPI:e_{10}^m:e_5^{hm}}[TFPI:PRO^h]^{v10} - k^+_{TFPI:e_{10}^m:e_5^h}[TFPI:E_{10}^m]e_5^h + k^-_{TFPI:e_{10}^m:e_5^h}[E_{10}^m:TFPI:E_5^h] \quad (97)$$

$$\frac{d}{dt}[TFPI:PRO^h]^{v10} = +k^+_{TFPI:PRO^h:v_{10}} PRO^h TFPI - k^-_{TFPI:PRO^h:v_{10}}[TFPI:PRO^h]^{v10} + k^+_{TFPI:e_{10}^m:e_5^{hm}}[TFPI:E_{10}^p][TFPI:E_{10}^m]e_5^{hm} - k^-_{TFPI:e_{10}^m:e_5^{hm}}[TFPI:PRO^h]^{v10} \quad (98)$$

$$\frac{d}{dt}[TFPI:PRO^h]^{v5} = +k^+_{TFPI:PRO^h:v_5} PRO^h TFPI - k^-_{TFPI:PRO^h:v_5}[TFPI:PRO^h]^{v5} + k^+_{TFPI:e_{10}:e_5^{hm}}[TFPI:E_5^{hm}]e_{10}^m - k^-_{TFPI:e_{10}:e_5^{hm}}[TFPI:PRO^h]^{v5} \quad (99)$$

$$\frac{d}{dt}[E_{10}^m:TFPI:E_5^{hm}] = +k^+_{TFPI:e_{10}:e_5^h m}[TFPI:E_{10}^m]e_5^{hm} - k^-_{TFPI:e_{10}:e_5^h m}[E_{10}^m:TFPI:E_5^{hm}] + k^+_{TFPI:e_5^{hm}:e_{10} m}[TFPI:E_5^{hm}]e_{10}^m - k^-_{TFPI:e_5^{hm}:e_{10} m}[E_{10}^m:TFPI:E_5^{hm}] + k_{10}^{ont} p_{10}^{avail} - k_{10}^{offt}[E_{10}^m:TFPI:E_5^{hm}] + k_5^{ont}[E_{10}^m:TFPI:E_5^h]p_5^{avail} - k_5^{offt}[E_{10}^m:TFPI:E_5^{hm}] \quad (100)$$

$$\frac{d}{dt}[E_{10}:TFPI:E_5^h] = +k_{flow}([E_{10}:TFPI:E_5^h]^{up} - [E_{10}:TFPI:E_5^h]) \tag{101}$$
$$+k^+_{TFPI:e_{10}:e_5^h}[TFPI:E_{10}]e_5^h$$
$$-k^-_{TFPI:e_{10}:e_5^h}[E_{10}:TFPI:E_5^h]$$
$$+k^+_{TFPI:e_5^h:e_{10}}[TFPI:E_5^h]e_{10}$$
$$-k^-_{TFPI:e_5^h:e_{10}}[E_{10}:TFPI:E_5^h]$$
$$-k_5^{ont}[E_{10}:TFPI:E_5^h]p_5^{avail}$$
$$+k_5^{offt}[E_{10}:TFPI:E_5^{hm}]$$
$$-k_{10}^{ont}[E_{10}:TFPI:E_5^h]p_{10}^{avail}$$
$$+K_{10}^{offt}[E_{10}^m:TFPI:E_5^h]$$

$$\frac{d}{dt}[E_{10}:TFPI:E_5^{hm}] = +k_5^{ont}[E_{10}:TFPI:E_5^h]p_5^{avail} \tag{102}$$
$$-k_5^{offt}[E_{10}:TFPI:E_5^{hm}]$$
$$-k_{10}^{ont}[E_{10}:TFPI:E_5^{[}hm]]p_{10}^{avail}$$
$$+k_{10}^{offt}[E_{10}^m:TFPI:E_5^{hm}]$$
$$+k^+_{TFPI:e_5^{hm}:e_{10}}[TFPI:E_5^{hm}]e_{10}$$
$$-k^-_{TFPI:e_5^{hm}:e_{10}}[E_{10}:TFPI:E_5^{hm}]$$

$$\frac{d}{dt}[E_{10}^m:TFPI:E_5^h] = +k_{10}^{ont}[E_{10}:TFPI:E_5^h]p_{10}^{avail} \tag{103}$$
$$-k_{10}^{offt}[E_{10}^m:TFPI:E_5^h]$$
$$-k_5^{ont}[E_{10}^m:TFPI:E_5^h]p_5^{avail}$$
$$+k_5^{offt}[E_{10}^m:TFPI:E_5^{hm}]$$
$$+k^+_{TFPI:e_{10}^m:e_5^h}[TFPI:E_{10}^m]e_5^h$$
$$-k^-_{TFPI:e_{10}^m:e_5^h}[E_{10}^m:TFPI:E_5^h]$$

$$\frac{d}{dt}[PRO^h:E_2^m] = +k^+_{PRO^h:e_2^m}PRO^hE_2^m - k^-_{PRO^h:e_2^m}[PRO^h:E_2^m] \tag{104}$$
$$-k^{cat}_{PRO^h:e_2^m}[PRO^h:E_2^m]$$

$$\frac{d}{dt}[E_9:AT] = -k_9^{on}p_9^{avail}[E_9:AT] \tag{105}$$
$$+k_9^{off}[E_9^m:AT] + k_{e_9}^{AT}e_9[AT]$$
$$+k_{flow}([E_9:AT]_{up} - [E_9:AT])$$
$$-k_9^{on}p_{91}^{avail}[E_9:AT] + k_9^{off}[E_9^{m*}:AT]$$

$$\frac{d}{dt}[E_9^m:AT] = +k_{e_9^m}^{AT}e_9^m[AT] \tag{106}$$
$$-k_9^{off}[E_9^m:AT] + k_9^{on}p_9^{avail}[E_9:AT]$$

$$\frac{d}{dt}[E_9^{m*}:AT] = +k_{e_9^m}^{AT}e_9^{m*}[AT] \tag{107}$$
$$-k_9^{off}[E_9^{m*}:AT] + k_9^{on}p_{91}^{avail}[E_9:AT]$$

$$\frac{d}{dt}[E_{10}:AT] = +k_{e_{10}}^{AT}e_{10}[AT] \tag{108}$$
$$+k_{flow}([E_{10}:AT]_{up} - [E_{10}:AT])$$
$$+k_{10}^{off}[E_{10}^m:AT] - k_{10}^{on}p_{10}^{avail}[E_{10}:AT]$$

$$\frac{d}{dt}[E_{10}^m : AT] = +k_{e_{10}^m}^{AT} e_{10}^m [AT] \quad (109)$$
$$-k_{10}^{off}[E_{10}^m : AT] + k_{10}^{on} p_{10}^{avail} [E_{10} : AT]$$

$$\frac{d}{dt}[E_2 : AT] = +k_{e_2}^{off}[E_2^m : AT] - k_{e_2}^{on} p_2^{avail}[E_2 : AT] \quad (110)$$
$$+k_{e_2}^{AT} e_2 + k_{flow}([E_2 : AT]_{up} - [E_2 : AT])$$

$$\frac{d}{dt}[E_2^m : AT] = +k_{e_2^m}^{AT} e_2^m [AT] \quad (111)$$
$$-k_{e_2}^{off}[E_2^m : AT] + k_{e_2}^{on} p_2^{avail}[E_2 : AT]$$

$$\frac{d}{dt}[E_{11} : AT] = +k_{e_{11}}^{AT} e_{11}[AT] \quad (112)$$
$$-k_{e_{11}}^{AT}[E_{11} : AT][AT]$$
$$+k_{11}^{off}[E_{11}^{m*} : AT] - k_{11}^{on} p_{111}^{avail}[E_{11} : AT]$$
$$+k_{e_{11}}^{ATH} e_{11}[AT : Hep] - k_{e_{11}}^{ATH}[E_{11} : ATH][AT : Hep]$$
$$+k_{flow}([E_{11} : ATH]_{up} - [E_{11} : ATH])$$
$$-k_{e_{11}}^{on} p_{111}^{avail}[E_{11} : ATH] + k_{e_{11}}^{off}[E_{11}^{m*} : ATH]$$

$$\frac{d}{dt}[AT : E_{11} : AT] = +k_{e_{11}}^{AT}[E_{11} : AT][AT] \quad (113)$$

$$\frac{d}{dt}[E_{11}^{m*} : AT] = +k_{e_{11}}^{AT} e_{11}^{m*}[AT] \quad (114)$$
$$-k_{11}^{off}[E_{11}^{m*} : AT] + k_{11}^{on} p_{111}^{avail}[E_{11} : AT]$$

$$\frac{d}{dt}[E_{11}^h : AT] = +k_{e_{11}}^{AT} e_{11}^h [AT] \quad (115)$$
$$+k_{11}^{off}[E_{11}^{hm} : AT] - k_{11}^{on} p_{11}^{avail}[E_{11}^h : AT]$$

$$\frac{d}{dt}[E_{11}^{hm} : AT] = +k_{e_{11}}^{AT} e_{11}^{hm}[AT] \quad (116)$$
$$-k_{11}^{off}[E_{11}^{hm} : AT] + k_{11}^{on} p_{11}^{avail}[E_{11}^h : AT]$$

$$\frac{d}{dt}[AT] = -k_{e_9}^{AT} e_9[AT] - k_{e_9^m}^{AT} e_9^m [AT] \quad (117)$$
$$+k_{e_9^m}^{AT} e_9^{m*}[AT]$$
$$-k_{e_{10}}^{AT} e_{10}[AT] + ke_{10}^{m\,AT} e_{10}^m [AT]$$
$$-k_{e_2}^{AT} e_2[AT] - k_{e_2^m}^{AT} e_2^m [AT]$$
$$-k_{e_{11}}^{AT} e_{11}[AT] - k_{e_{11}}^{AT}[E_{11} : AT][AT]$$
$$-k_{e_{11}}^{AT} e_{11}^{m*}[AT] - k_{e_{11}}^{AT} e_{11}^h [AT]$$
$$-k_{e_{11}}^{AT} e_{11}^h [AT]$$
$$+k_{flow}([AT]_{up} - [AT])$$
$$-k_{[AT:Hep]}^{+}[Hep][AT] + k_{[AT:Hep]}^{-}[AT : Hep]$$

$$\frac{d}{dt}[Hep] = -k_{[AT:Hep]}^{+}[Hep][AT] + k_{[AT:Hep]}^{-}[AT : Hep] \quad (118)$$

$$\frac{d}{dt}[AT:Hep] = +k^{+}_{[AT:Hep]}[Hep][AT] - k^{-}_{[AT:Hep]}[AT:Hep] \tag{119}$$
$$-k^{ATH}_{e_{10}} e_{10}[AT:Hep] - k^{ATH}_{e^m_{10}} e^m_{10}[AT:Hep]$$
$$-k^{ATH}_{e_2} e_2[AT:Hep] - k^{ATH}_{e^m_2} e^m_2[AT:Hep]$$
$$-k^{ATH}_{e_9} e_9[AT:Hep] - k^{ATH}_{e^m_9} e^m_9[AT:Hep]$$
$$-k^{ATH}_{e^m_9} e^{m*}_9[AT:Hep]$$
$$-k^{ATH}_{e_{11}} e_{11}[AT:Hep] - k^{ATH}_{e_{11}}[E_{11}:ATH][AT:Hep]$$
$$-k^{ATH}_{e_{11}} e^{m*}_{11}[AT:Hep] - k^{ATH}_{e_{11}} e^h_{11}[AT:Hep]$$
$$-k^{ATH}_{e_{11}} e^{hm}_{11}[AT:Hep]$$

$$\frac{d}{dt}[E_{10}:ATH] = +k^{ATH}_{e_{10}} e_{10}[AT:Hep] + k_{flow}([E_{10}:ATH]_{up} - [E_{10}:ATH]) \tag{120}$$
$$-k^{on}_{10} p^{avail}_{10}[E_{10}:ATH] + k^{off}_{10}[E^m_{10}:ATH]$$

$$\frac{d}{dt}[E^m_{10}:ATH] = +k^{ATH}_{e^m_{10}} e^m_{10}[AT:Hep] \tag{121}$$
$$+k^{on}_{10} p^{avail}_{10}[E_{10}:ATH] - k^{off}_{10}[E^m_{10}:ATH]$$

$$\frac{d}{dt}[E_2:ATH] = +k^{ATH}_{e_2} e_2[AT:Hep] + k_{flow}([E_2:ATH]_{up} - [E_2:ATH]) \tag{122}$$
$$-k^{on}_{e_2} p^{avail}_2[E_2:ATH] + k^{off}_{e_2}[E^m_2:ATH]$$

$$\frac{d}{dt}[E^m_2:ATH] = +k^{ATH}_{e^m_2} e^m_2[AT:Hep] \tag{123}$$
$$+k^{on}_2 p^{avail}_2[E_2:ATH] - k^{off}_{e_2}[E^m_2:ATH]$$

$$\frac{d}{dt}[E_9:ATH] = +k^{ATH}_{e_9} e_9[AT:Hep] + k_{flow}([E_9:ATH]_{up} - [E_9:ATH]) \tag{124}$$
$$-k^{on}_{e_9} p^{avail}_9[E_9:ATH] + k^{off}_{e_9}[E^m_9:ATH]$$
$$-k^{on}_{e_9} p^{avail}_{91}[E_9:ATH] + k^{off}_9[E^{m*}_9:ATH]$$

$$\frac{d}{dt}[E^{m*}_9:ATH] = +k^{ATH}_{e^m_9} e^m_9[AT:Hep] \tag{125}$$
$$+k^{on}_{e_9} p^{avail}_9[E_9:ATH] - k^{off}_{e_9}[E^m_9:ATH]$$

$$\frac{d}{dt}[E^{m*}_9:ATH] = +k_{e^m_9:ATH} e^{m*}_9[AT:Hep] \tag{126}$$
$$+k^{on}_{e_9} p^{avail}_{91}[E_9:ATH] - k^{off}_{e_9}[E^{m*}_9:ATH]$$

$$\frac{d}{dt}[ATH:E_{11}:ATH] = +k^{ATH}_{e_{11}}[AT:Hep][E_{11}:ATH] \tag{127}$$
$$+k_{flow}([ATH:E_{11}:ATH]_{up} - [ATH:E_{11}:ATH])$$

$$\frac{d}{dt}[E^{m*}_{11}:ATH] = +k^{ATH}_{e_{11}} e^{m*}_{11}[AT:Hep] + k^{on}_{e_{11}} p^{avail}_{111}[E_{11}:ATH] \tag{128}$$
$$-k^{off}_{e_{11}}[E^{m*}_{11}:ATH]$$

$$\frac{d}{dt}[E^h_{11}:ATH] = +k^{ATH}_{e_{11}} e^h_{11}[AT:Hep] \tag{129}$$
$$+k_{flow}([E^h_{11}:ATH]_{up} - [E^h_{11}:ATH])$$
$$+k^{off}_{e_{11}}[E^{hm}_{11}:ATH] - k^{on}_{e_{11}} p^{avail}_{111}[E^h_{11}:ATH]$$

$$\frac{d}{dt}[E^{hm}_{11}:ATH] = +k^{ATH}_{e_{11}} e^{hm}_{11}[AT:Hep] \tag{130}$$
$$-k^{off}_{11}[E^{hm}_{11}:ATH]$$
$$+k^{on}_{11} p^{avail}_{111}[E^h_{11}:ATH]$$

S1 Table. INITIAL PLASMA LEVELS. Descriptions, notation and labels for each parameter associated with initial plasma levels are listed. The value of each parameter is found in the corresponding table listed above.

Description	Notation	Label	Table
Prothrombin	z_2	Z_2	S8
Factor V	z_5	Z_5	S8
Factor VII	z_7	Z_7	S8
Factor VIII	z_8	Z_8	S8
Factor IX	z_9	Z_9	S8
Factor X	z_{10}	Z_{10}	S8
Factor XI	z_{11}	Z_{11}	S8
TFPI	[TFPI]	TFPI	S8
AT	[AT]	AT	S8
Hep	[Hep]	Hep	S8

S2 Table. PLATELET CHARACTERISTICS. Descriptions, notation and labels for each parameter associated with platelet characteristics are listed. The value of each parameter is found in the corresponding table listed above.

Description	Notation	Label	Table
Platelet count	PL^{up}	PLup	S8
Binding site number for II	N_2	N2	S8
Binding site number for IIa	N_2^*	N2*	S8
Binding site number for V/Vh/Va	N_5	N5	S8
Binding site number for VIII/VIIIa	N_8	N8	S8
Binding site number for IX	N_9	N9	S8
Binding site number for IXa	N_9^*	N9*	S8
Binding site number for X/Xa	N_{10}	N10	S8
Binding site number for XI	N_{11}	N11	S8
Binding site number for XIa	N_{11}^*	N11*	S8
Rate of unactivated platelets adhering to SE	k_{adh}^{+}	kadh	S14
Rate of activated platelets adhering to SE	$k_{adh}^{+,*}$	kadh1	S14
Rate of platelet activation by platelet in solution	k_{plt}^{act}	kact$_{plt}$	S14
Rate of platelet activation on SE	$k_{plt}^{act,*}$	kact*$_{plt}$	S14
Rate of platelet activation by thrombin	$k_{e_2}^{act}$	kact$_{e2}$	S14

S3 Table. KINETIC RATE CONSTANTS. Descriptions, notation and labels for each parameter associated with kinetic rate constants are listed. The value of each parameter is found in the corresponding table listed above.

Description	Notation	Label	Table
Rates of activation of TF:VII by fX	K_M	KZ7mE10M	S9
	$k^{cat}_{z_7^m:e_{10}}$	KZ7mE10CAT	S9
	$k^{-}_{z_7^m:e_{10}}$	KZ7mE10MI	S9
Rates of activation of fX by TF:VIIa	K_M	KZ10E7mM	S9
	$k^{cat}_{z_{10}:e_7^m}$	KZ10E7mCAT	S9
	$k^{-}_{z_{10}:e_7^m}$	KZ10E7mMI	S9
Rates of activation of fIX by TF:VIIa	K_M	KZ9E7mM	S9
	$k^{cat}_{z_9:e_7^m}$	KZ9E7mCAT	S9
	$k^{-}_{z_9:e_7^m}$	KZ9E7mMI	S9
Rates of binding of fVII/fVIIa to TF	k_7^{on}	K7ON	S9
	k_7^{off}	K7OFF	S10
Rates of activation of TF:VII by fXa	K_M	KZ7E10M	S10
	$k^{cat}_{z_7:e_{10}^m}$	KZ7E10CAT	S10
	$k^{-}_{z_7:e_{10}^m}$	KZ7E10MI	S10
Rates of activation of TF:VII by fIIa	K_M	KZ7E2M	S10
	$k^{cat}_{z_7:e_2}$	KZ7E2CAT	S10
	$k^{-}_{z_7:e_2}$	KZ7E2MI	S10
Rates of activation of TF:VII by fIXa	K_M	KZ7E9M	S10
	$k^{cat}_{z_7:e_9}$	KZ7E9CAT	S10
	$k^{-}_{z_7:e_9}$	KZ7E9MI	S10
Rates of activation of fV by fIIa	K_M	KZ5E2M	S10
	$k^{cat}_{z_5:e_2}$	KZ5E2CAT	S10
	$k^{-}_{z_5:e_2}$	KZ5E2MI	S10
Rates of activation of fVIII by fIIa	K_M	KZ8E2M	S10
	$k^{cat}_{z_8:e_2}$	KZ8E2CAT	S10
	$k^{-}_{z_8:e_2}$	KZ8E2MI	S10
Rates of activation of fIX by fXIa-fXIa	$k^{+}_{z_9:e_{11}}$	KZ9E11P	S10
	$k^{cat}_{z_9:e_{11}}$	KZ9E11CAT	S10
	$k^{-}_{z_9:e_{11}}$	KZ9E11MI	S10
Rates of activation of fIX by fXIa-fXI	$k^{+}_{z_9:e_{11}^h}$	KZ9E11P	S10
	$k^{cat}_{z_9:e_{11}^h}$	KZ9E11CAT	S10
	$k^{-}_{z_9:e_{11}^h}$	KZ9E11MI	S10
Rates of activation of fXI by fIIa	$k^{+}_{z_{11}:e_2}$	KZ11E2P	S10
	$k^{cat}_{z_{11}:e_2}$	KZ11E2CAT	S10
	$k^{-}_{z_{11}:e_2}$	KZ11E2MI	S11
Rates of binding of fX/fXa to plt. surface	k_{10}^{on}	K10ON	S11
	k_{10}^{off}	K10OFF	S11
Rates of binding of fV/fVa to plt. surface	k_5^{on}	K5ON	S11
	k_5^{off}	K5OFF	S1
Rates of binding of fVIII/fVIIIa to plt. surface	k_8^{on}	K8ON	S1
	k_8^{off}	K8OFF	S1
Rates of binding of fIX/fIXa to plt. surface	k_9^{on}	K9ON	S1
	k_9^{off}	K9OFF	S11
Rates of binding of fII/fIIa to plt. surface	k_2^{on}, $k_2^{on,*}$	K2ON, K2SON	S1
	k_2^{off}, $k_2^{off,*}$	K2OFF, K2SOFF	S11
Rates of binding of fXI/fXIa to plt. surface	k_{11}^{on}, $k_{11}^{on,*}$	K11ON, K11SON	S11
	k_{11}^{off}, $k_{11}^{off,*}$	K11OFF, K11SOFF	S12

S4 Table. KINETIC RATE CONSTANTS. Descriptions, notation and labels for each parameter associated with kinetic rate constants are listed. The value of each parameter is found in the corresponding table listed above.

Description	Notation	Label	Table
Rates of activation of fV by fXa on plt. surface	K_M	KZ5mE10mM	S12
	$k^{cat}_{z_5^m:e_{10}^m}$	KZ5mE10mCAT	S12
	$k^{-}_{z_5^m:e_{10}^m}$	KZ5mE10mMI	S12
Rates of activation of fV by fIIa on plt. surface	K_M	KZ5mE2mM	S12
	$k^{cat}_{z_5:e_2^m}$	KZ5mE2mCAT	S12
	$k^{-}_{z_5:e_2^m}$	KZ5mE2mMI	S12
Rates of activation of fVIII by fXa on plt. surface	K_M	KZ8ME10MM	S12
	$k^{cat}_{z_8:e_{10}^m}$	KZ8ME10MCAT	S12
	$k^{-}_{z_8:e_{10}^m}$	KZ8ME10MMI	S12
Rates of activation of fVIII by fIIa on plt. surface	K_M	KZ8ME2MM	S12
	$k^{cat}_{z_8:e_2^m}$	KZ8mE2mCAT	S12
	$k^{-}_{z_8:e_2^m}$	KZ8mE2mMI	S12
Rates of activation of fX by TEN on plt. surface	K_M	KZ10mTENM	S12
	$k^{cat}_{z_{10}^m:TEN}$	KZ10mTENCAT	S12
Rates of activation of fII by PRO on plt. surface	K_M	KZ2mPROM	S12
	$k^{cat}_{z_2^m:PRO}$	KZ2mPROCAT	S12
Rates of activation of fXI by fIIa on plt. surfaces	$k^{+}_{z_{11}^m:e_2^m}$	KZ11mE2mP	S12
	$k^{cat}_{z_{11}^m:e_2^m}$	KZ11mE2mCAT	S12
	$k^{-}_{z_{11}^m:e_2^m}$	KZ11mE2mMI	S12
Rates of activation of fIX by fXIa-fXIa on plt. surface	K_M	KZ9mE11mP	S12
	$k^{cat}_{z_9^m:e_{11}^m}$	KZ9mE11mCAT	S12
	$k^{-}_{z_9^m:e_2^m}$	KZ9mE11mMI	S12
Rates of formation of TEN on plt. surface	$k^{+}_{e_8^m:e_9^m}$	KE8mE9mP	S12
	$k^{-}_{e_8^m:e_9^m}$	KE8mE9mMI	S12
Rates of formation of PRO on plt. surface	$k^{+}_{e_5^m:e_{10}^m}$	KE5mE10mP	S12
	$k^{-}_{e_5^m:e_{10}^m}$	KE5mE10mMI	S12
Rates of inhibition of fXa by TFPI	$k^{+}_{tfpia:e_{10}}$	KTFPI_E10_P	S13
	$k^{-}_{tfpia:e_{10}}$	KTFPI_E10_M	S13
Rates of inhibition of TF:VIIa by TFPIa	$k^{+}_{tfpia:e_7^m}$	KTFPIa_E7m_P	S13
	$k^{-}_{tfpia:e_7^m}$	KTFPIa_E7m_M	S13
Rates of inhibition of fVa by APC on plt. surface	K_M	KE5mAPCM	S13
	$k^{cat}_{e_5^m:APC}$	KE5mAPCCAT	S13
	$k^{-}_{e_5^m:APC}$	KE5mAPCMI	S13
Rates of inhibition of fVIIIa by APC on plt. surface	K_M	KE8mAPCM	S13
	$k^{cat}_{e_8^m:APC}$	KE8mAPCCAT	S13
	$k^{-}_{e_8^m:APC}$	KE8mAPCMI	S13
Rates of inhibition of fIIa by TM on plt. surface	k^{on}_{TM}	KTMP	S13
	k^{off}_{TM}	KTMM	S14

S5 Table. NEW KINETIC RATE CONSTANTS ADDED IN TFPI EXTENSION. Descriptions, notation and labels for each parameter associated with kinetic rate constants are listed. The value of each parameter is found in the corresponding table listed above.

Description	Notation	Label	Table
Rates of binding of fV-h by fXa on plt. surface	$k^{+}_{e5hme10m}$	KE5HME10MP	S10
	$k^{-}_{e_5^m:e_{10}^m}$	KE5HME10MMI	S12
Rates of activation of II by PROh on plt. surface	K_M	KZ2MPROHM	S10
	$k^{cat}_{z_2^m:PRO^h}$	KZ2MPROHCAT	S12
	$k^{-}_{z_2^m:PRO^h}$	KZ2MPROHMI	S12
Rates of activation of fV-h by IIa on plt. surface	K_M	KE5HME2MM	S10
	$k^{cat}_{e_5^{hm}:e_2^m}$	KE5HME2MCAT	S12
	$k^{-}_{e_5^{hm}:e_2^m}$	KE5HME2MMI	S12
Rates of activation of fV-h by IIa in fluid	K_M	KE5HE2M	S10
	$k^{cat}_{e_5^h:e_2}$	KE5HE2CAT	S12
	$k^{-}_{e_5^h:e_2}$	KE5HE2MI	S12
Rates of binding of fV-h by TFPI on plt. surface	$k^{+}_{TFPI:e_5^{hm}}$	KTFPIE5HMP	S10
	$k^{-}_{TFPI:e_5^{hm}}$	KTFPIE5HMMI	S13
Rates of binding of fV-h by TFPI in fluid	$k^{+}_{TFPI:e_5^h}$	KTFPIE5HP	S10
	$k^{-}_{TFPI:e_5^h}$	KTFPIE5HMI	S13
Rates of binding of fXa by TFPI on plt. surface	$k^{+}_{TFPI:e_{10}^m}$	KTFPI_E10M_P	S10
	$k^{-}_{TFPI:e_{10}^m}$	KTFPI_E10M_MI	S13
Rates of binding of PROh by TFPI on plt. surface by binding fXa	$k^{+}_{TFPI:PRO^h:v_{10}}$	KTFPIPROHV10P	S10
	$k^{-}_{TFPI:PRO^h:v_{10}}$	KTFPIPROHV10MI	S13
Rates of binding of PROh by TFPI on plt. surface by binding fV-h	$k^{+}_{TFPI:PRO^h:v_5}$	KTFPIPROHV5P	S10
	$k^{-}_{TFPI:PRO^h:v_5}$	KTFPIPROHV5MI	S13
Rates of inactivation of fV-h by APC on plt. surface	K_M	KE5HMAPCM	S10
	$k^{cat}_{e_5^{hm}}$	KE5HMAPCCAT	S13
	$k^{-}_{e_5^{hm}:APC}$	KE5HMAPCMI	S13
Rates of inactivation of fV-h by APC in fluid	K_M	KE5HAPCM	S10
	$k^{cat}_{e_5^h}$	KE5HAPCCAT	S13
	$k^{-}_{e_5^h:APC}$	KE5HAPCMI	S13

S6 Table. NEW KINETIC RATE CONSTANTS ADDED IN AT EXTENSION. Descriptions, notation and labels for each parameter associated with kinetic rate constants are listed. The value of each parameter is found in the corresponding table listed above.

Description	Notation	Label	Table
Rates of inactivation of fIXa by AT on plt. surface	$k_{e_9^m}^{AT}$	KE9MATIII	S13
Rates of inactivation of fXa by AT on plt. surface	$k_{e_{10}^m}^{AT}$	KE10MATIII	S13
Rates of inactivation of IIa by AT on plt. surface	$k_{e_2^m}^{AT}$	KE2MATIII	S13
Rates of inactivation of fXIa by AT on plt. surface	$k_{e_{11}^m}^{AT}$	KE11MATIII	S13
Rates of inactivation of fIXa by AT in fluid	$k_{e_9}^{AT}$	KE9ATIII	S13
Rates of inactivation of fXa by AT in fluid	$k_{e_{10}}^{AT}$	KE10ATIII	S13
Rates of inactivation of IIa by AT in fluid	$k_{e_2 AT}$	KE2ATIII	S13
Rates of inactivation of fXIa by AT in fluid	$k_{e_{11}}^{AT}$	KE11ATIII	S13
Rates of binding of AT by Heparin on plt. surface	$k_{AT:Hep}^{+}$	KATBHEPMI	S13
	$k_{AT:Hep}^{-}$	KATBHEPMI	S13
Rates of inactivation of fIXa by ATH on plt. surface	$k_{e_9^m}^{ATH}$	KE9MATH	S13
Rates of inactivation of fXa by ATH on plt. surface	$k_{e_{10}^m}^{ATH}$	KE10MATH	S13
Rates of inactivation of IIa by ATH on plt. surface	$k_{e_2^m}^{ATH}$	KE2MATH	S13
Rates of inactivation of fXIa by AT on plt. surface	$k_{e_{11}^m}^{ATH}$	KE11MATH	S13
Rates of inactivation of fIXa by ATH in fluid	$k_{e_9}^{ATH}$	KE9ATH	S13
Rates of inactivation of fXa by ATH in fluid	$k_{e_{10}}^{ATH}$	KE10ATH	S13
Rates of inactivation of IIa by ATH in fluid	$k_{e_2}^{ATH}$	KE2ATH	S13
Rates of inactivation of fXIa by AT in fluid	$k_{e_{11}}^{ATH}$	KE11ATH	S13

Kinetic and Physical Parameter Values:

S7 Table. DIFFUSION COEFFICIENTS FOR PLATELETS AND FLUID-PHASE CHEMICAL SPECIES (a) From (4). (b) From (5).

Platelets	2.5×10^{-7} cm^2/s	a
Proteins	5×10^{-7} cm^2/s	b

S8 Table. NORMAL CONCENTRATIONS AND SURFACE BINDING SITE NUMBERS (a) From (6). (b) From (7). (c) (8) suggests that normal plasma concentration of fVIIa is about 1% of the normal fVII concentration. (d) From (9). (e) (f) From (10). (g) Estimated as described in the text of the Supplementary Information. (h) From (11). (i) From (12). (j) From (13). (k) From (14). (l) From (15, 16). (m) Number of fV molecules released per activated platelet (17). (n) Maximum concentration of platelets in a 2 μm high reaction zone assuming that 20 platelets can cover a 10μm-by-10μm injured surface (18). (o) From (19). (p) Refer to heparin dosage calculation in later section of supplemental material.

Prothrombin	1.4 μM	a
Factor V	0.01 μM	b
Factor VII	0.01 μM	a
Factor VIIa	0.1 nM	c
Factor VIII	1.0 nM	a
Factor IX	0.09 μM	a
Factor X	0.17 μM	a
Factor XI	30.0 nM	a
TFPI	0.5 nM	d
Protein C	65 nM	e
Platelet count	$2.5(10)^5/\mu$l	f
N_2	1000/plt	g
N_2^*	1000/plt	g
N_5	3000/plt	h
N_8	450/plt	i
N_9	250/plt	j
N_9^*	250/plt	j
N_{10}	2700/plt	k
N_{11}	1500/plt	l
N_{11}^*	250/plt	l
n_5	3000/plt	m
p_{PLAS}	0.167 nM	n
AT	2.4 nM	o
$LMWH$	253 nM	p
UFH	759 nM	p

S9 Table. REACTIONS ON SUBENDOTHELIUM (a) $k^{cat}_{z_7^m:e_{10}} = 5.0$ sec^{-1} and $K_M = 1.2 \cdot 10^{-6}$ M (20). (b) $k^{cat}_{z_7^m:e_2} = 6.1 \cdot 10^{-2}$ sec^{-1} and $K_M = 2.7 \cdot 10^{-6}$ M (20). (d) $k^{cat}_{z_{10}:e_7^m} = 1.15$ sec^{-1} and $K_M = 4.5 \cdot 10^{-7}$ M (6). (d) $k^{cat}_{z_9:e_7^m} = 1.15$ sec^{-1} and $K_M = 2.4 \cdot 10^{-7}$ M (21). (e) $K_d = 1.0 \cdot 10^{-10}$ M (22).

Activation (of -, by -)							
(TF:VII,fXa)	E_{10}, Z_7^m	$Z_7^m : E_{10}$	E_7^m	$k^+_{z_7^m:e_{10}}$=5.0 · 10^6	$k^-_{z_7^m:e_{10}}$=1.0	$k^{cat}_{z_7^m:e_{10}}$=5.0	a
(TF:VII, fIIa)	E_2, Z_7^m	$Z_7^m : E_2$	E_7^m	$k^+_{z_7^m:e_2}$=3.92 · 10^5	$k^-_{z_7^m:e_2}$=1.0	$k^{cat}_{z_7^m:e_2}$=6.1 · 10^{-2}	b
(fX, TF:VIIa)	E_7^m, Z_{10}	$Z_{10} : E_7^m$	E_{10}	$k^+_{z_{10}:e_7^m}$=5.0 · 10^6	$k^-_{z_{10}:e_7^m}$=1.0	$k^{cat}_{z_{10}:e_7^m}$=1.15	c
(fIX, TF:VIIa)	E_7^m, Z_9	$Z_9 : E_7^m$	E_9	$k^+_{z_9:e_7^m}$=9.4 · 10^6	$k^-_{z_9:e_7^m}$=1.0	$k^{cat}_{z_9:e_7^m}$=1.15	d
Binding (of -, with -)							
(fVII, TF)	Z_7, TF		Z_7^m	k_7^{on}=5.0 · 10^7	k_7^{off}=5.0 · 10^{-3}		e
(fVIIa, TF)	E_7, TF		E_7^m	k_7^{on}=5.0 · 10^7	k_7^{off}=5.0 · 10^{-3}		e

S10 Table. REACTIONS IN THE PLASMA (a) $k^{cat}_{z_7:e_{10}} = 5.0\,\text{sec}^{-1}$ and $K_M = 1.2 \cdot 10^{-6}$ M (20). (b) $k^{cat}_{z_7:e_2} = 6.1 \cdot 10^{-2}\,\text{sec}^{-1}$ and $K_M = 2.7 \cdot 10^{-6}$ M (20) (c) $k^{cat}_{z_5:e_2} = 0.23\,\text{sec}^{-1}$ and $K_M = 7.17 \cdot 10^{-8}$ M (23). (d) $k^{cat}_{z_8:e_2} = 0.9\,\text{sec}^{-1}$ (24) and $K_M = 2 \cdot 10^{-7}$ M (25). (e) $k^{cat}_{z_{11}:e_2} = 1.3 \cdot 10^{-4}$, $K_M = 50$nM (26). Rate constants apply also for thrombin-activation of XIa-XI. (f) $k^{cat}_{z_9:e^h_{11}} = 0.21$, $K_M = 0.2\mu$M (27, 28). Rate constants apply also for activation of IX by XIa-XIa.

Reaction	Reactants	Complex	Product	$M^{-1}sec^{-1}$	sec^{-1}	sec^{-1}	Note
Activation (of -, by -)							
(fVII, fXa)	Z_7, E_{10}	$Z_7 : E_{10}$	E_7	$k^+_{z_7:e_{10}}=5 \cdot 10^6$	$k^-_{z_7:e_{10}}=1.0$	$k^{cat}_{z_7:e_{10}}=5.0$	a
(fVII, fIIa)	Z_7, E_2	$Z_7 : E_2$	E_7	$k^+_{z_7:e_2}=3.92 \cdot 10^5$	$k^-_{z_7:e_2}=1.0$	$k^{cat}_{z_7:e_2}=6.1 \cdot 10^{-2}$	b
(fV, fIIa)	Z_5, E_2	$Z_5 : E_2$	E_5	$k^+_{z_5:e_2}= 1.73 \cdot 10^7$	$k^-_{z_5:e_2}=1.0$	$k^{cat}_{z_5:e_2}=0.23$	c
(fVIII, fIIa)	Z_8, E_2	$Z_8 : E_2$	E_8	$k^+_{z_8:e_2}=2.64 \cdot 10^7$	$k^-_{z_8:e_2}=1.0$	$k^{cat}_{z_8:e_2}=0.9$	d
(fXI-fXI, fIIa)	Z_{11}, E_2	$Z_{11} : E_2$	E^h_{11}	$k^+_{z_{11}:e_2} = 2.0 \cdot 10^7$	$k^-_{z_{11}:e_2} = 1.0$	$k^{cat}_{z_{11}:e_2} = 1.3 \cdot 10^{-4}$	e
(fIX, fXIa)	Z_9, E^h_{11}	$Z_9 : E^h_{11}$	E_9	$k^+_{z_9:e^h_{11}} = 0.6 \cdot (10)^7$	$k^-_{z_9:e^h_{11}} = 1.0$	$k^{cat}_{z_9:e^h_{11}} = 0.21$	f

S11 Table. BINDING TO PLATELET SURFACES (a) For fIX binding to platelets, $K_d = 2.5 \cdot 10^{-9}$ M (13), and for fX binding to platelets, K_d has approximately the same value (11). For fX binding to PCPS vesicles, the on-rate is about 10^7 M^{-1}sec^{-1} and the off-rate is about 1.0 sec^{-1} (29) giving a dissociation constant of about 10^{-7} M. To estimate on- and off-rates for the higher-affinity binding of fX to platelets, we keep the on-rate the same as for vesicles and adjust the off-rate to give the correct dissociation constant. The rates for fIX binding with platelets are taken to be the same as for fX binding. (b) We assume binding constants for fIXa binding to the specific fIXa binding sites are the same as for shared sites. (c) fV binds with high-affinity to phospholipids (PCPS) (29) and we use the same rate constants reported there to describe fV binding to platelets. (d) The K_d for fVIII binding with platelets is taken from (12). We set the off-rate k_8^{off} for fVIII binding to platelets equal to that for fV binding to platelets, and calculate the on-rate k_8^{on}. (e) For prothrombin interactions with platelets, K_d is reported to be $5.9 \cdot 10^{-7}$ M (30). We choose k_2^{off} and set $k_2^{\text{on}} = k_2^{\text{off}}/K_d$. (f) Estimated as described in the text of the Supplementary Information. (g) $K_d = 10$ nM (31). (h) $K_d = 1.7$ nM (16).

Reaction	Reactants	Products	M^{-1}sec^{-1}	sec^{-1}	Note
Factor IX	Z_9, P_9	Z_9^m	k_9^{on}=$1.0 \cdot 10^7$	k_9^{off}=$2.5 \cdot 10^{-2}$	a
Factor IXa	E_9, P_9	E_9^m	k_9^{on}=$1.0 \cdot 10^7$	k_9^{off}=$2.5 \cdot 10^{-2}$	a
Factor IXa	E_9, P_9^*	$E_9^{m,*}$	k_9^{on}=$1.0 \cdot 10^7$	k_9^{off}=$2.5 \cdot 10^{-2}$	b
Factor X	Z_{10}, P_{10}	Z_{10}^m	k_{10}^{on}=$1.0 \cdot 10^7$	k_{10}^{off}=$2.5 \cdot 10^{-2}$	a
Factor Xa	E_{10}, P_{10}	E_{10}^m	k_{10}^{on}=$1.0 \cdot 10^7$	k_{10}^{off}=$2.5 \cdot 10^{-2}$	a
Factor V	Z_5, P_5	Z_5^m	k_5^{on}=$5.7 \cdot 10^7$	k_5^{off}=0.17	c
Factor Vh	E_5^h, P_5	E_5^{hm}	k_5^{on}=$5.7 \cdot 10^7$	k_5^{off}=0.17	c
Factor Va	E_5, P_5	E_5^m	$k_5^{\text{on}} = 5.7 \cdot 10^7$	k_5^{off}=0.17	c
Factor VIII	Z_8, P_8	Z_8^m	$k_8^{\text{on}} = 5.0 \cdot 10^7$	k_8^{off}=0.17	d
Factor VIIIa	E_8, P_8	E_8^m	$k_8^{\text{on}} = 5.0 \cdot 10^7$	k_8^{off}=0.17	d
Factor II	Z_2, P_2	Z_2^m	$k_2^{\text{on}} = 1.0 \cdot 10^7$	k_2^{off}=5.9	e
Factor IIa	E_2, P_2	E_2^m	$k_2^{*,\text{on}} = 1.0 \cdot 10^7$	$k_2^{*,\text{off}} = 0.2$	f
Factor XI	Z_{11}, P_{11}	Z_{11}^m	$k_{z_{11}}^{\text{on}} = 1.0 \cdot 10^7$	$k_{z_{11}}^{\text{off}}$=0.1	g
Factor XIa	E_{11}, P_{11}^*	E_{11}^m	$k_{e_{11}}^{\text{on}} = 1.0 \cdot 10^7$	$k_{e_{11}}^{\text{off}}$=0.017	h

S12 Table. REACTIONS ON PLATELET SURFACES (a) $k^{\text{cat}}_{z_5^m:e_{10}^m} = 0.046$ sec^{-1} and $K_M = 10.4 \cdot 10^{-9}$ M (32). (b) The rate constants for thrombin activation of fV on platelets are assumed to be the same as in plasma. (c) $k^{\text{cat}}_{z_8^m:e_{10}^m} = 0.023$ sec^{-1} and $K_M = 2.0 \cdot 10^{-8}$ M (25). (d) The rate constants for thrombin activation of fVIII on platelets are assumed to be the same as in plasma. (e) The formation of the tenase and prothrombinase complexes is assumed to be very fast with $K_d = 1.0 \cdot 10^{-10}$ M (33). (f) $k^{\text{cat}}_{z_{10}^m:ten} = 20$ sec^{-1} and $K_M = 1.6 \cdot 10^{-7}$ M (34). (g) $k^{\text{cat}}_{z_2^m:pro} = 30$ sec^{-1} and $K_M = 3.0 \cdot 10^{-7}$ M (35). (h) $k^{\text{cat}}_{z_{11}^m:e_2^m} = 1.3 \cdot 10^{-4}$, $K_M = 50$ nM (26). Rate constants apply also for thrombin-activation of Plt-XIa-XI. (i) $k^{\text{cat}}_{z_9^m:e_{11}^{h,m}} = 0.21$, $K_M = 0.2\mu$M (27, 28). Rate constants apply also for activation of platelet-bound IX by Plt-XIa-XIa.

Reaction	Reactants	Complex	Product	M^{-1}sec^{-1}	sec^{-1}	sec^{-1}	Note
**Activation (of -, by -)**							
(V, Xa)	Z_5^m, E_{10}^m	$Z_5^m : E_{10}^m$	E_5^{hm}	$k^+_{z_5^m:e_{10}^m}$=1.0 · 10^8	$k^-_{z_5^m:e_{10}^m}$=1.0	$k^{\text{cat}}_{z_5^m:e_{10}^m}$=4.6 · 10^{-2}	a
(V, IIa)	Z_5^m, E_2^m	$Z_5^m : E_2^m$	E_5^m	$k^+_{z_5^m:e_2^m}$=1.73 · 10^7	$k^-_{z_5^m:e_2^m}$=1.0	$k^{\text{cat}}_{z_5^m:e_2^m}$= 0.23	b
(Vh, IIa)	E_5^{hm}, E_2^m	$E_5^{hm} : E_2^m$	E_5^m	$k^+_{z_5^m:e_2^m}$=1.73 · 10^7	$k^-_{z_5^m:e_2^m}$=1.0	$k^{\text{cat}}_{z_5^m:e_2^m}$= 0.23	b
(VIII, Xa)	Z_8^m, E_{10}^m	$Z_8^m : E_{10}^m$	E_8^m	$k^+_{z_8^m:e_{10}^m}$=5.1 · 10^7	$k^-_{z_8^m:e_{10}^m}$=1.0	$k^{\text{cat}}_{z_8^m:e_{10}^m}$=2.3 · 10^{-2}	c
(VIII, IIa)	Z_8^m, E_2^m	$Z_8^m : E_2^m$	E_8^m	$k^+_{z_8^m:e_2^m}$=2.64 · 10^7	$k^-_{z_8^m:e_2^m}$=1.0	$k^{\text{cat}}_{z_8^m:e_2^m}$= 0.9	d
(X, VIIIa:IXa)	Z_{10}^m, TEN	$Z_{10}^m : TEN$	E_{10}^m	$k^+_{z_{10}^m:ten}$=1.31 · 10^8	$k^-_{z_{10}^m:ten}$=1.0	$k^{\text{cat}}_{z_{10}^m:ten}$=20.0	f
(X, VIIIa:IXa*)	Z_{10}^m, TEN^*	$Z_{10}^m : TEN^*$	E_{10}^m	$k^+_{z_{10}^m:ten}$=1.31 · 10^8	$k^-_{z_{10}^m:ten}$=1.0	$k^{\text{cat}}_{z_{10}^m:ten}$=20.0	f
(II, Vh:Xa)	$Z_2^m, PROh$	$Z_2^m : PROh$	E_2^m	$k^+_{z_2^m:pro}$= 1.03 · 10^8	$k^-_{z_2^m:pro}$=1.0	$k^{\text{cat}}_{z_2^m:pro}$=30.0	g
(II, Va:Xa)	Z_2^m, PRO	$Z_2^m : PRO$	E_2^m	$k^+_{z_2^m:pro}$= 1.03 · 10^8	$k^-_{z_2^m:pro}$=1.0	$k^{\text{cat}}_{z_2^m:pro}$=30.0	g
(XI-XI, IIa)	Z_{11}^m, E_2^m	$Z_{11}^m : E_2^m$	E_{11}^{hm}	$k^+_{z_{11}^m:e_2^m}$ = 2.0 · 10^7	$k^-_{z_{11}^m:e_2^m}$ = 1.0	$k^{\text{cat}}_{z_{11}^m:e_2^m}$ = 1.3 · 10^{-4}	h
(IX, XIa)	Z_9^m, E_{11}^{hm}	$Z_9^m : E_{11}^{hm}$	E_9	$k^+_{z_9^m:e_{11}^m}$ = 0.6 · 10^7	$k^-_{z_9^m:e_{11}^m}$ = 1.0	$k^{\text{cat}}_{z_9^m:e_{11}^m}$ = 0.21	i
**Binding (of -, with -)**							
(VIIIa, IXa)	E_8^m, E_9^m		TEN	k^+_{ten}=1.0 · 10^8	k^-_{ten}=0.01		e
(VIIIa, IXa*)	$E_8^m, E_9^{m,*}$		TEN^*	k^+_{ten}=1.0 · 10^8	k^-_{ten}=0.01		e
(Vh, Xa)	E_5^{hm}, E_{10}^m		$PROh$	k^+_{pro}=1.0 · 10^8	k^-_{pro}=0.01		e
(Va, Xa)	E_5^m, E_{10}^m		PRO	k^+_{pro}=1.0 · 10^8	k^-_{pro}=0.01		e

S13 Table. INHIBITION REACTIONS (a) From (19). (b) From (36). (c) For inhibition of fVa by APC, $k^{\text{cat}}_{e_5^m:APC} = 0.5$ sec^{-1} and $K_M = 12.5 \cdot 10^{-9}$ (37). We assume the same reaction rates for the inhibition of fVIIIa by APC. (d) From (38). (e)From (39). (f) From (40). (g) $K_d = 0.5$ nM and $[PC] = 65$ nM (41). (h)From (42). (i) $k_{PC:TM:e_2^{ec}} = 0.167$ sec^{-1} , $K_M = 0.7 \cdot 10^{-6}$ M (43).

Reaction	Reactants	Product	$M^{-1}sec^{-1}$	sec^{-1}	sec^{-1}	Note
Inactivation (of -, by -)						
(IXa, AT-III)	E_9, AT	$E_9 : AT$		$k_{e_9}^{AT}$=4.8 · 10^2		a
(Xa, AT-III)	E_{10}, AT	$E_{10} : AT$		$k_{e_{10}}^{AT}$=3.5 · 10^3		a
(IIa, AT-III)	E_2, AT	$E_2 : AT$		$k_{e_2}^{AT}$=1.4 · 10^4		a
(XIa, AT-III)	E_{11}, AT	$E_{11} : AT$		$k_{e_{11}}^{AT}$=2.4 · 10^2		a
(XIa:AT, AT-III)	$E_{11} : AT$, AT	$AT : E_{11} : AT$		$k_{e_{11}}^{AT}$=2.4 · 10^2		a
(IXa, ATH)	E_9, ATH	$E_9 : ATH$		$k_{e_9}^{AT}$=5 · 10^5		b
(Xa, ATH)	E_{10}, ATH	$E_{10} : ATH$		$k_{e_{10}}^{AT}$=1.3 · 10^6		b
(IIa, ATH)	E_2, ATH	$E_2 : ATH$		$k_{e_2}^{AT}$=5.3 · 10^6		b
(XIa, ATH)	E_{11}, ATH	$E_{11} : ATH$		$k_{e_{11}}^{AT}$=1 · 10^4		b
(XIa:AT, ATH)	$E_{11} : AT$, ATH	$ATH : E_{11} : ATH$		$k_{AT:e_{11}}^{in}$=1 · 10^4		b
(APC, Va)	$APC,\ E_5^m$	none	$k_{e_5^m:APC}^{+}$ = 1.2 · 10^8	$k_{e_5^m:APC}^{-}$ = 1.0	$k_{e_5^m:APC}^{cat}$ = 0.5	c
(APC, VIIIa)	$APC,\ E_8^m$	none	$k_{e_8^m:APC}^{+}$ = 1.2 · 10^8	$k_{e_8^m:APC}^{-}$ = 1.0	$k_{e_8^m:APC}^{cat}$ = 0.5	c
Binding (of -, with -)						
(TFPI, Xa)	$TFPI,E_{10}$	$TFPIa$	$k_{tfpia:e_{10}}^{+}$=1.6 · 10^7	$k_{tfpia:e_{10}}^{-}$=3.3 · 10^{-4}		d
(TFPI, Vh)	$TFPI,\ E_5^h$	$TFPI : E_5^h$	$k_{tfpi:e5h}^{+}$ = 0.05 · 10^9	$k_{tfpi:e5h}^{-}$ = 0.0045		e
(TFPI:Xa, Vh)	$TFPIa,\ E_5^h$	$E_5^h : TFPI : E_{10}$	$k_{tfpi:e5h}^{+}$ = 0.05 · 10^9	$k_{tfpi:e5h}^{-}$ = 0.0045		e
(TFPI:Vh, Xa)	$TFPI : E_5^h, E_{10}$	$E_5^h : TFPI : E_{10}$	$k_{tfpia:e_{10}}^{+}$=1.6 · 10^7	$k_{tfpia:e_{10}}^{-}$=3.3 · 10^{-4}		d
(Xa:Vh, TFPI)	$E_{10} : E_5^h, TFPIa$	$E_{10} : E_5^h : TFPI$	$k_{tfpibprohv5}^{+}$ = 0.05 · 10^9	$k_{tfpibprohv5}^{-}$ = 0.0045		e
(Xa:Vh, TFPI)	$E_{10} : E_5^h, TFPIa$	$TFPI : E_{10} : E_5^h$	$k_{tfpibprohv10}^{+}$ = 1.6 · 10^7	$k_{tfpibprohv10}^{-}$ = 3.3 · $10^{-}4$		d
(TFPIa, TF:VIIa)	$TFPIa,E_7^m$	$TFPIa : E_7^m$	$k_{tfpia:e_{10}}^{+}$=1.6 · 10^7	$k_{tfpia:e_{10}}^{-}$=3.3 · 10^{-4}		f
(TM, Thrombin)	$TM,\ E_2^{ec}$	$TM : E_2^{ec}$	k_{TM}^{on} = 1.0 · 10^8	k_{TM}^{off} = 5.0 · 10^{-2}		g
(AT-III, Heparin)	AT, Hep	ATH	k_{ATH}^{+} = 1.0	k_{ATH}^{-} = 2.77 · 10^7		h
Activation (of -, by -)						
(PC, TM:E_2^{ec})	$TM : E_2^{ec}$	APC	$k_{PC:TM:e_2^{ec}}^{+}$ = 1.7 · 10^6	$k_{PC:TM:e_2^{ec}}^{-}$ = 1.0	$k_{PC:TM:e_2^{ec}}^{cat}$ = 0.16	i

S14 Table. PLATELET TRANSITIONS (a) Estimated from data in (44, 45) as described in (46). (b) Estimated from data in (47) as described in (46). SE=subendothelium.

Reactants	Reactants	Products	$M^{-1}sec^{-1}$	sec^{-1}	Note
Unactivated platelet adhering to SE	PL, SE	PL_a^s	k_{adh}^{+}=2 $\cdot$ 10^{10}	k_{adh}^{-}=0	a
Activated platelet adhering to SE	PL_a^v, SE	PL_a^v	k_{adh}^{+}=2 $\cdot$ 10^{10}	k_{adh}^{-}=0	a
Platelet activation by platelet in solution	PL, PL_a^v	$2PL_a^v$	$k_{plt}^{\text{act}} = 3 \cdot 10^8$		b
Platelet activation on SE	PL, PL_a^s	PL_a^v, PL_a^s	$k_{plt}^{\text{act}} = 3 \cdot 10^8$		b
Platelet activation by thrombin	PL, E_2	PL_a^v		$k_{e_2}^{\text{act}}$=0.50	b

S3 CONVERSION OF HEPARIN POTENCY TO MOLAR CONCENTRATION

Based on the recommended dosage of heparin treatment (0.3-0.7 U/ml) (48), we use the value of 0.5 U/ml. Based on the information from second international standard for heparin by WHO, the conversion factor will be 130 U/mg (49). By using the mean molecular weight of heparin as 15kDa (50), we can get:
0.5 U/ml * 1/130 mg/U = 0.0038 mg/ml
$0.0038 g/L * 1/15000 mol/g = 2.53 * 10^-7 M = 253 nM$
For the LMWH, since the molecular weight of LMWH varies based on different product (51), we use 5kDa as its molecular weight. Therefore, the concentration of heparin at 100% is determined by:
$0.0038 g/L * 1/5000 mol/g = 2.53 * 10^-7 M = 759 nM$
Both concentrations were set as 100% baseline dosage concentration. For example, when we use 50% concentration of UFH and LMWH, we are using 126.5 nM and 379.5 nM respectively.

S4 LOCAL SENSITIVITY ANALYSIS - METHOD

As in our previous work (52), we again focus on the sensitivity of three special thrombin metrics:

1. Lag time: A measure of how fast the system is turned on, defined as the amount of time required for thrombin to reach 1 nM.
2. Maximum relative rate: A measure of how fast thrombin is produced once the system is turned on, defined as

$$\max_{t>t_{1nM}} \left(\frac{d[\text{thrombin}]}{dt}/[\text{thrombin}]\right).$$

3. Final concentration: The thrombin concentration after 20 minutes of clotting activity.

We examine the sensitivity of these metrics to two types of parameter variations: (i) the plasma levels of seven zymogens and two inhibitors, and (ii) the values of 24 new kinetic parameters that are related to TFPI reactions. We used a derivative-based approach to quantify the sensitivity of each metric with respect to centered difference in the parameters in a range of values (50%, 75%, 100%, 125% and 150% for the plasma level parameters, and 90%, 95%, 100%, 105% and 110% for kinetic parameters). The standard values for each plasma level parameters were set to the initial conditions. Similar to our previous SA results (52), we found that each of the metrics behaved monotonically with respect to varying each plasma level from 50% to 150% of the standard values, as shown in Fig. (S2A-C) and the kinetic parameters from 90% to 110% as shown in Fig. (S3A-C). The min/max values of these monotonic curves shows the change in the thrombin metric due to the factor change. Clotting factor variations had a significant effect on all three thrombin metrics but the largest change in the thrombin metrics due to variations in the kinetic parameters was less than 0.2% and therefore, we did not characterize the sensitivity of these parameters further. For the clotting factors and inhibitors, we quantified their sensitivity by the absolute difference they produced in each metric when considering their extremal values (i.e., 50% and 150%). For each metric, we ranked the parameters by considering their relative absolute difference. We define $x = (x_1, x_2, ..., x_p)$ to be the standard model parameter values and $m_i(x_{j,y\%})$ to be the values of the i-th metric when parameter j is chosen to be y% of its standard value and all other parameters are chosen to be at their standard value. The local sensitivity of the i-th metric to the j-th parameter is then:

$$LS_j^i = \frac{|m_i(x_{j,150\%} - m_i(x_{j,50\%}))|}{\max_k(|m_i(x_{k,150\%} - m_i(x_{k,50\%}))|)}$$

Each sensitivity score, LS, is then a number between 0 and 1 and we use these values to rank the input sensitivities. In our results, we denote LS scores higher than 0.75 with solid black triangles, LS scores from 0.25 to 0.75 as gray triangles, and for LS scores lower than 0.25 we use open triangles. In addition, because the response of the system outputs was monotonic throughout the entire range, we show separately the change in each metric for the 50% increase with the triangle upward and a 50% decrease with a triangle facing downward. Then the y-value of the triangle corresponds to its result on the output.

The local SA results in Fig. (S2D-I) reveal the most influential clotting factors and inhibitors, when perturbed one at a time for each of the three thrombin metrics. Fig. (S2D) shows that FVIII and FX have the greatest effect on the lag time, where an increase in either FVIII or FX levels by 50% leads to an approximately 10% decrease in the lag time from baseline. This is seen with the solid black (LS scores above 0.75), upward-facing (increase in factor level) triangles, with y-value near -10% showing the decrease in lag time. Comparing with sesitivity results from our old model((52)), we see an increased sensitivity to TFPI, where a decrease/increase by 50% leads to about a 8% decrease/5% increase in the lag time from baseline, respectively, although the TFPI LS score still does not reach 0.75. Fig. (S2E) shows that variations in FVIII, FIX, and FX have the largest effect on the maximum relative rate of thrombin generation, and this metric still has low sensitivity to TFPI. These findings are the same as in our previous results and make sense since these factors influence the rate of formation of the tenase complex on platelets, which affects the amplification stage of coagulation, and the inhibitory effect by TFPI does not alter such amplification process. It also indicates that new TFPI inhibitory reactions does not have significant influence towards the rate at which thrombin is being made. Fig. (S2F) shows that the final concentration metric is sensitive only to prothrombin (FII) as was found previously((52)). The corresponding LS scores are shown in Fig. (S2H-I).

Fig.(S3) demonstrates the local SA results for each of kinetic parameters that are related to TFPI reactions. Forward and reverse rate for each of the reaction are varied by 10% and change in lag time, maximum relative rate and final concentrations were observed. The results indicate that slight perturbation in reaction kinetics has minimal effect towards these three thrombin metrics, where none of the kinetic parameter caused more than 1% change from baseline in each cases. Such insensitiveness of the kinetic parameters, however, indicates the tolerance of the model towards the possible error in the kinetic parameters retrieved from experimental design.

S5 OTHER FIGURES

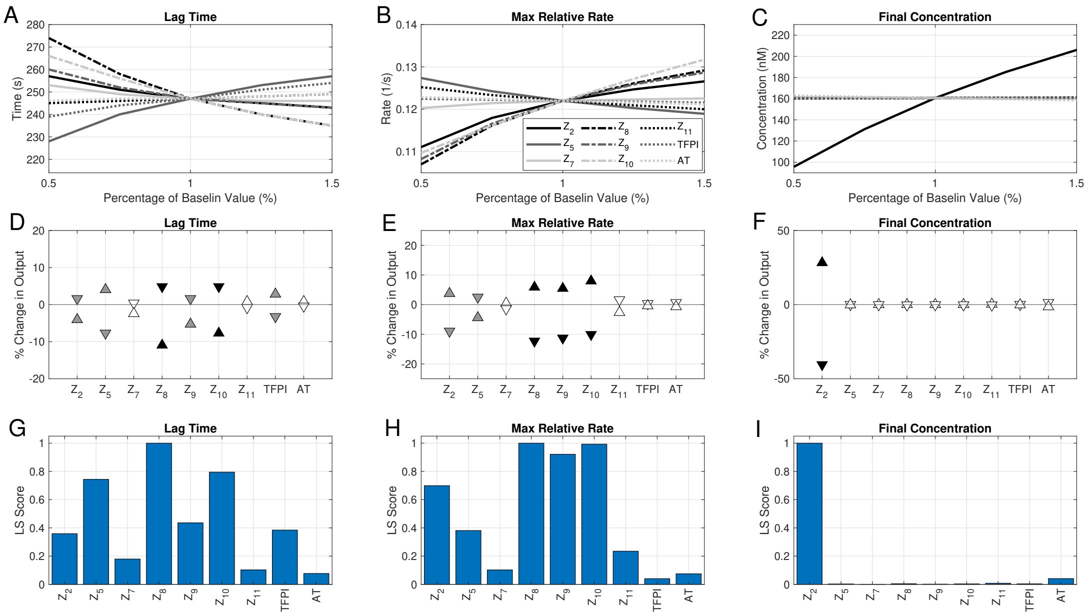

Figure S2: Local sensitivity analysis of clotting factor levels on thrombin metrics. The initial conditions of clotting factor and inhibitor levels were varied between 50% and 150% of their baseline values. Shown are (A,B,C) the amplitude change in lag time, maximum relative rate, and final thrombin concentration, (D,E,F) the percentage change in each of the metrics, and (G,H,I) the LS scores for each metric and for each species. Solid black triangles represent the species with LS score higher than 0.75, gray triangles for LS scores from 0.25 to 0.75, and open triangles for LS lower than 0.25. The arrow direction indicates if the variable was increased or decreased.

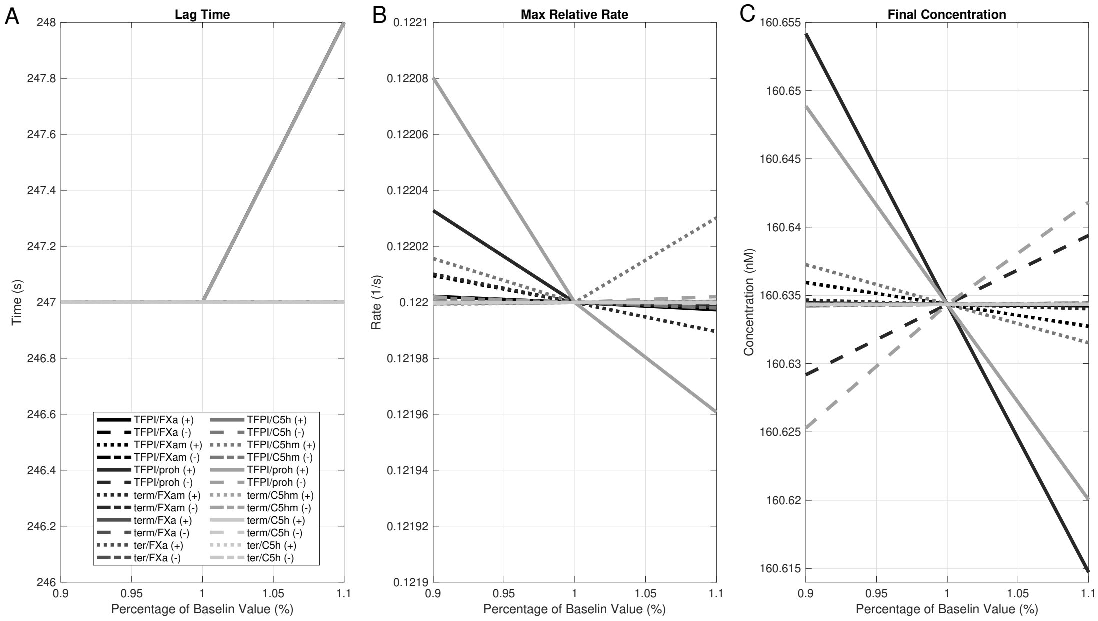

Figure S3: Local sensitivity analysis of TFPI-related kinetic rates on thrombin metrics. The new kinetic parameters were varied between 90% and 110% of their baseline values. Shown are (A,B,C) the amplitude of the changes in the lag time, maximum relative rate and final thrombin concentration due to the kinetic parameter variations. The plus/minus sign indicates the association/dissociation rate, respectively. Lower case *m* represents the components that are bound to platelet surface. The forward slash shows which two components are interacting each other, while the "ter" and "term" indicates interactions that involve a ternary complex and whether the species is in plasma or bound to the platelet surface, respectively. For example: term/FXa (+) indicates the rate of association between the platelet-bound TFPI:FV-h complex and the fluid phase FXa to form the ternary complex FXa:TFPI:FV-h.

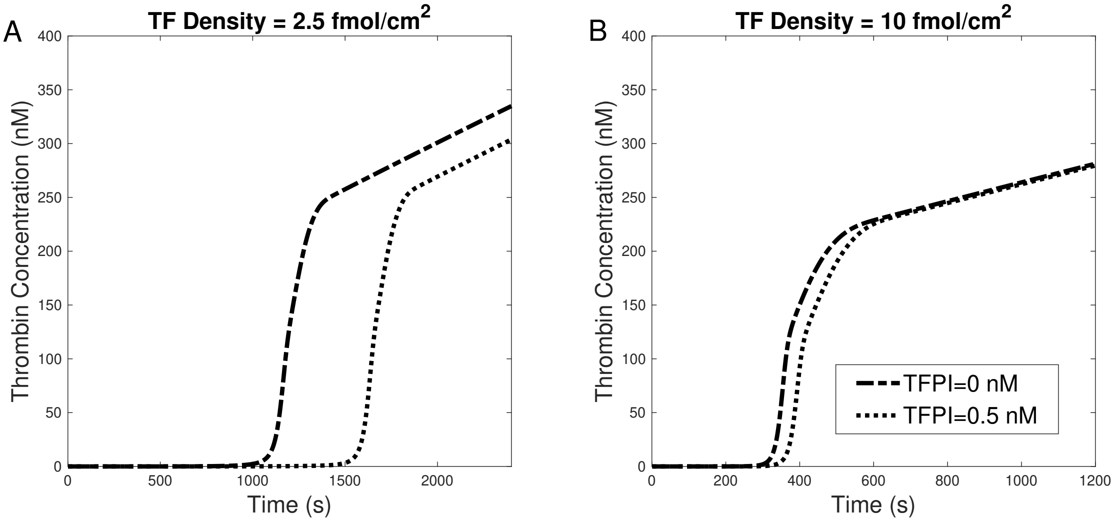

Figure S4: Thrombin generation time courses under different TFPI levels (0 nM and 0.5 nM) plotted in linear scale. TF level is varied by 2.5 fmol/cm^2 (A) and 10 fmol/cm^2 (B). Shear rate is fixed at 100/s.

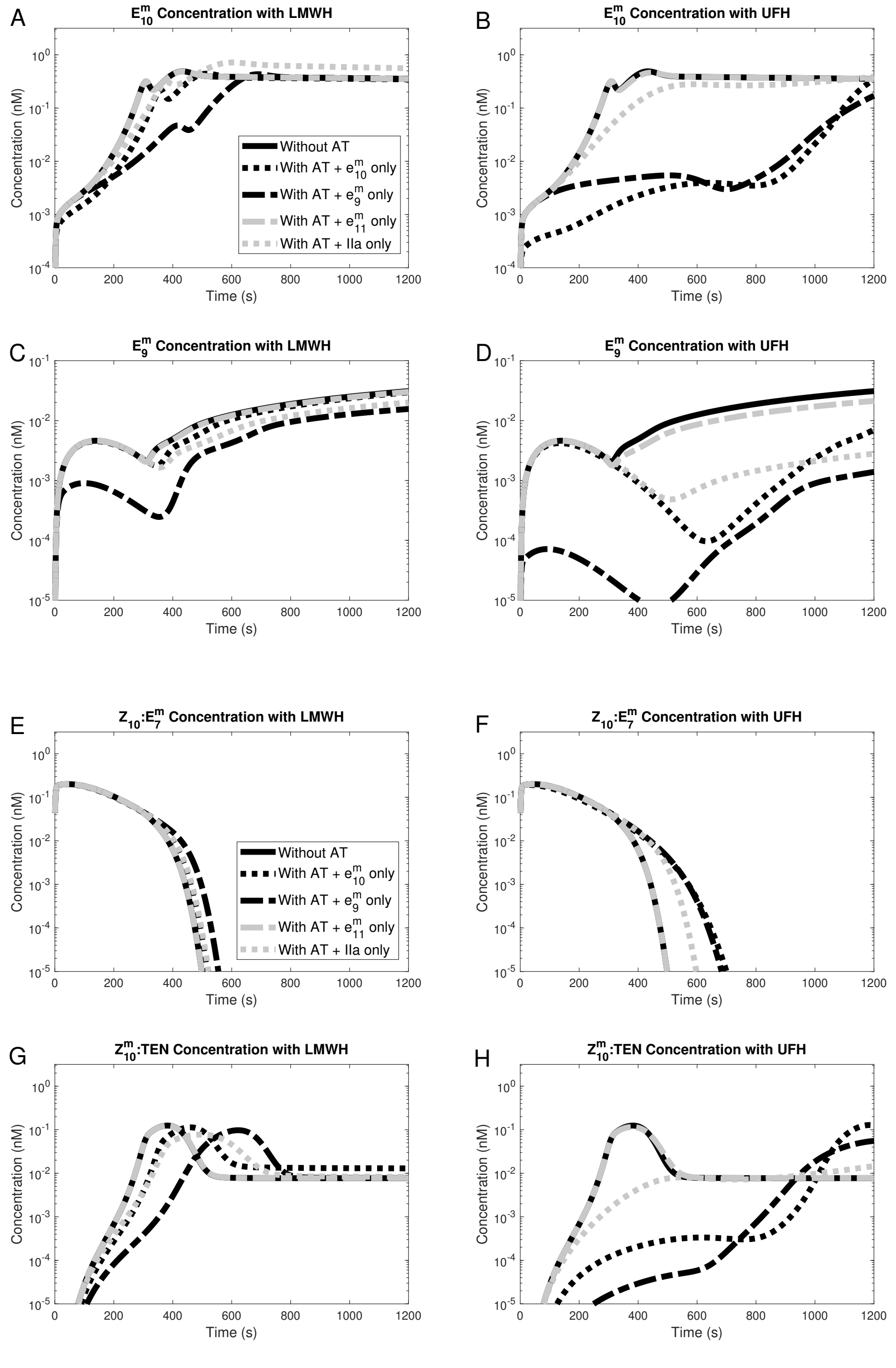

Figure S5: FXa concentration in the presence of LMWH (A) or UFH (B), FIXa concentration in the presence of LMWH (C) or UFH (D), FX:TF:VIIa concentration in the presence of LMWH (E) or UFH (F), and FX:tenase concentration in the presence of LMWH (G) or UFH (H). The time course is obtained from simulations in which we turn off all the AT-mediated inactivation reactions and then allow inhibition of FXa, FIXa, FXIa and thrombin, individually and one by one. Each curve thus shows thrombin/tenase generation when there is either no or only one inactivation reaction that exists in the system. TF density was set to 15 fmol/cm^2 and shear rate was set to 100/s. Heparin concentration is fixed to 100% of the standard therapeutic concentration.

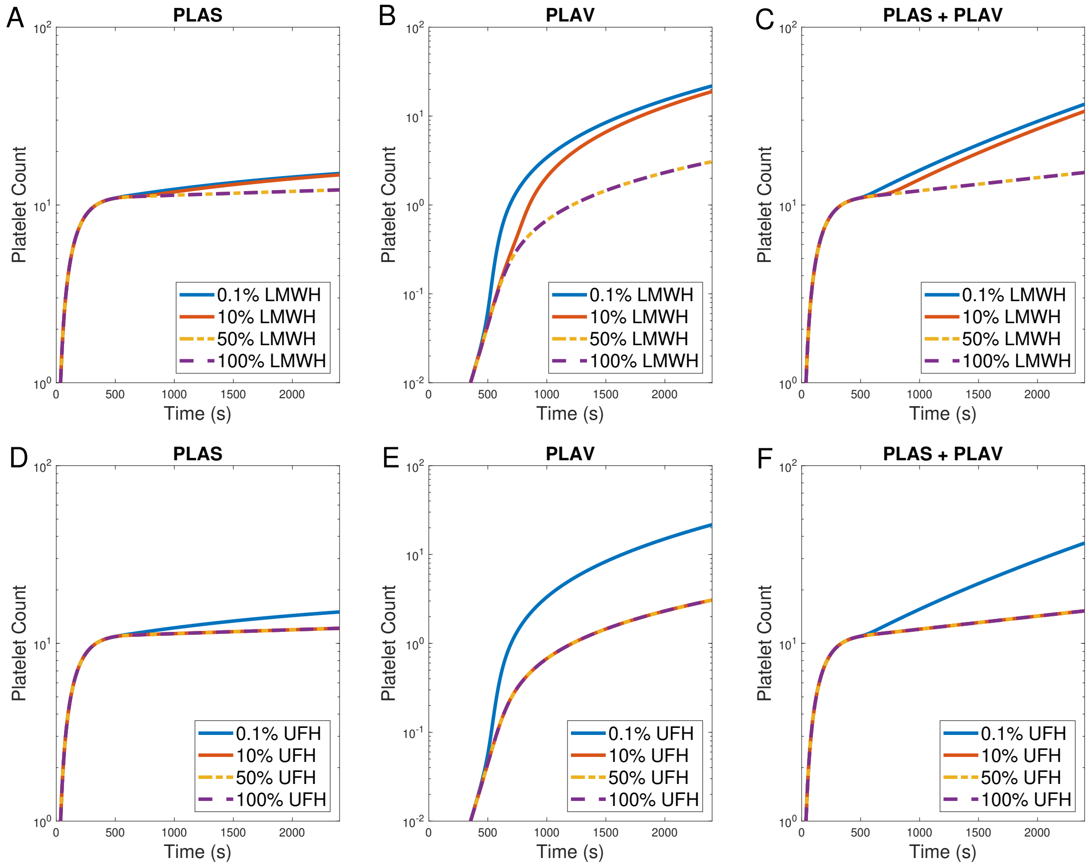

Figure S6: Subendothelium-attacehed platelet count (PLAS) and paltelet-attached-activated platelet count (PLAV) time course with varied LMWH treatmemnt (A-C) or UFH treatment (D-F). TF level is fixed to 6 $fmol/cm^2$. We examined how heparin in the system might affect platelet deposition. We specifically looked at two types of platelets: those that are activated and bound to subendothelium (PLAS), and those that are activated and bound to deposited platelets (PLAV), and their sum. The platelets accumulate on the subendothelium (SE) and PLAV eventually plateaus due to the limited space at the SE, whereas platelets above the injury site will continue to grow. Increasing the heparin concentrations led to decreases in both platelet species through time.This is because by increasing amount of heparin, it can greatly reduce the thrombin in the reaction zone, which leads to reduced amount of platelet to be activated by thrombin. Such a reduction can cause a shift from platelet-bound platelet to subendothelium-bound platelet. The increase in subendothelium-bound platelet will physically cover up the surface, which can negatively influence the initiation phase of coagulation.

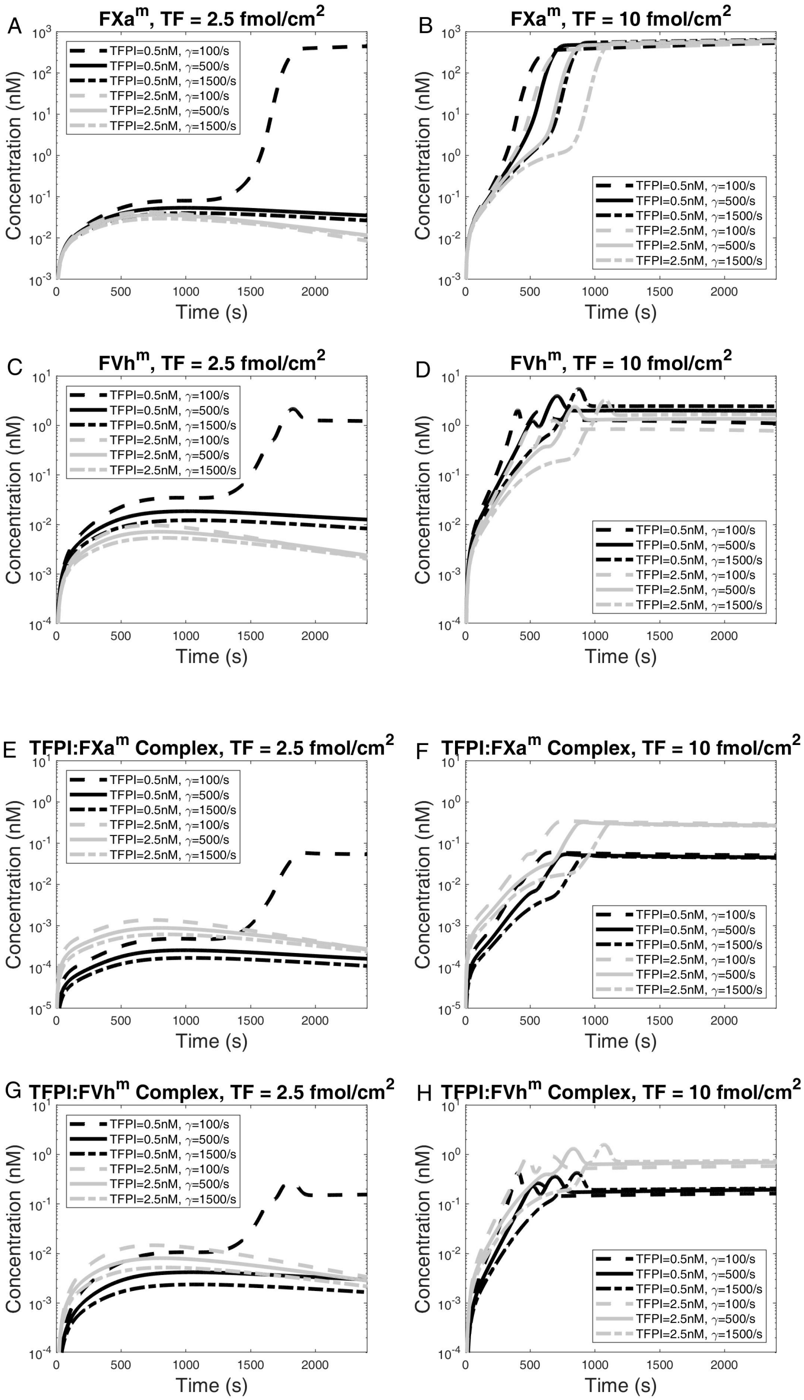

Figure S7: Concentration time course of platelet surface bound FXa, FV-h, and their complexes with TFPI. TF level is varied by 2.5 fmolcm% (A,C,E,G) and 10 fmol/cm^2 (B,D,F,H). Under each TF level, TFPI level is varied by 0.5 nM and 2.5 nM, and shear rate is varied by 100/s, 500/s and 1500/s.

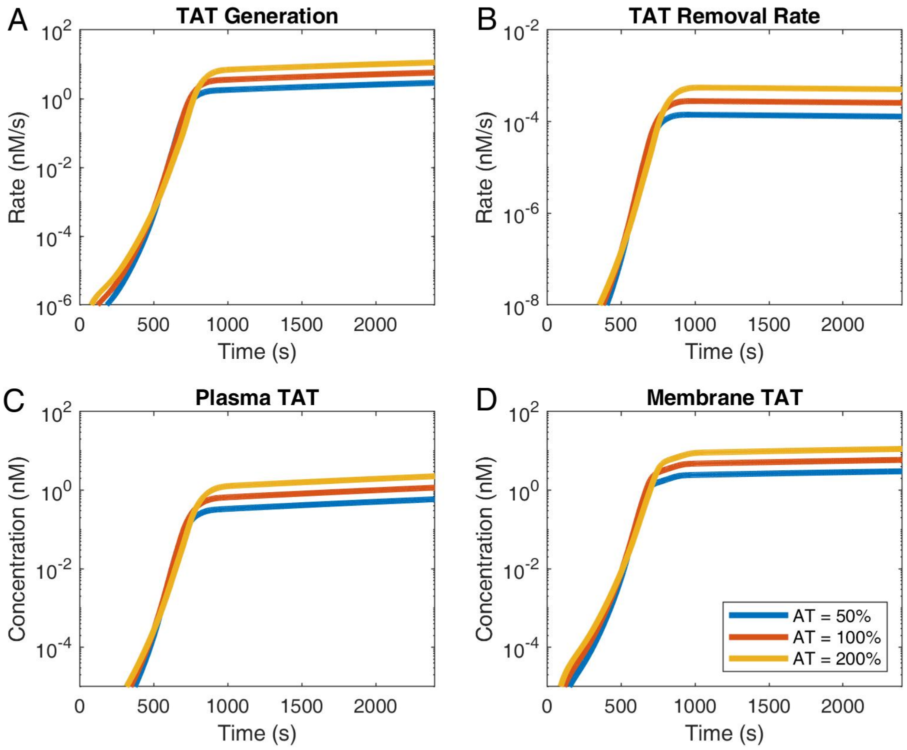

Figure S8: Instantaneous generation and removal of TAT (A,B), and accumulative concentration of TAT in plasma (C) and on the platelet membrane (D). TF level is fixed to 5 fmol/cm^2, and shear rate is fixed to 100/s.

REFERENCES

1. Kuharsky, A. L., and A. L. Fogelson, 2001. Surface-Mediated Control of Blood Coagulation: The Role of Binding Site Densities and Platelet Deposition. *Biophysical Journal* 80:1050–1074. https://linkinghub.elsevier.com/retrieve/pii/S0006349501760857.

2. Fogelson, A. L., and N. Tania, 2005. Coagulation under Flow: The Influence of Flow-Mediated Transport on the Initiation and Inhibition of Coagulation. *Pathophysiol Haemos Thromb* 34:91–108. https://www.karger.com/Article/FullText/89930.

3. Fogelson, A., Y. Hussain, and K. Leiderman, 2012. Blood Clot Formation under Flow: The Importance of Factor XI Depends Strongly on Platelet Count. *Biophysical Journal* 102:10–18. https://linkinghub.elsevier.com/retrieve/pii/S0006349511013142.

4. Turitto, V. T., and E. F. Leonard, 1972. Platelet adhesion to a spinning surface. *Trans. Amer. Soc. Artif. Int. Organs* 18:348–54.

5. Young, M., P. Carroad, and R. Bell, 1980. Estimation of diffusion coefficients of proteins. *Biotech and Bioeng* 22:947–955.

6. Mann, K., M. Nesheim, W. Church, P. Haley, and S. Krishnaswamy, 1990. Surface-dependent Reactions of the Vitamin K-dependent enzyme complexes. *Blood* 76:1–16.

7. Mann, K., E. Bovill, and S. Krishnaswamy, 1991. Surface-dependent reactions in the propagation phase of blood coagulation. *Ann. N. Y. Acad. Sci.* 614:63–75.

8. Morrissey, J. H., 1995. Tissue Factor Modulation of Factor VIIa Activity: Use in Measuring Trace Levels of Factor VIIa in Plasma,. *Thromb. Haemost.* 74:185–188.

9. Novotny, W., S. Brown, J. Miletich, D. Rader, and G. Broze, 1991. Plasma antigen levels of the lipoprotein-associated coagulation inhibitor in patient samples. *Blood* 78:387–93.

10. Weiss, H. J., 1975. Platelet Physiology and Abnormalities of Platelet Function (Part 1). *New Engl. J. Med.* 293:531–541.

11. Walsh, P. N., 1994. Platelet-Coagulant Protein Interactions. *In* R. W. Colman, J. Hirsh, V. J. Marder, and E. W. Salzman, editors, Hemostasis and Thrombosis: Basic Principles and Clinical Practice, J.B. Lippincott Company, Philadelphia, PA, 629–651. 3d edition.

12. Nesheim, M. E., D. D. Pittman, J. H. Wang, D. Slonosky, A. R. Giles, and R. J. Kaufman, 1988. The binding of S-labeled recombinant Factor VIII to activated and unactivated human platelets. *J Biol Chem* 263:16467.

13. Ahmad, S., R. Rawala-Sheikh, and P. Walsh, 1989. Comparative interactions of Factor IX and Factor IXa with human platelets. *Journal of Biological Chemistry* 264:3244–3251.

14. Mann, K., S. Krishnaswamy, and J. Lawson, 1992. Surface-dependent Hemostasis. *Semin Hematol* 29:213–26.

15. Baglia, F., B. Jameson, and P. Walsh, 1995. Identification and Characterization of a Binding Site for Platelets in the Apple 3 domain of coagulation Factor XI. *J Biol Chem* 270:6734–40.

16. Miller, T., D. Sinha, T. Baird, and P. Walsh, 2007. A Catalytic Domain Exosite (Cys^{527}-Cys^{542}) in Factor XIa mediates binding to a site on activated platelets. *Biochemistry* 46:14450–60.

17. Tracy, P., L. L. Eide, E. J. Bowie, and K. G. Mann, 1982. Radioimmunoassay of factor V in human plasma and platelts. *Blood* 60:59–63.

18. Hubbell, J. A., and L. V. McIntire, 1986. Platelet Active Concentration Profiles Near Growing Thrombi. A Mathematical Consideration. *Biophys J* 50:937–945.

19. Olson, S. T., I. Björk, and J. D. Shore, 1993. [30] Kinetic characterization of heparin-catalyzed and uncatalyzed inhibition of blood coagulation proteinases by antithrombin. *In* Methods in Enzymology, Elsevier, volume 222, 525–559. https://linkinghub.elsevier.com/retrieve/pii/007668799322033C.

20. Butenas, S., and K. Mann, 1996. Kinetics of Human Factor VII activation. *Biochemistry* 35:1904–1910.

21. Limentani, S. A., B. C. Furie, and B. Furie, 1994. The Biochemistry of Factor IX. *In* R. Colman, J. Hirsh, V. Marder, and E. Salzman, editors, Hemostasis and Thrombosis: Basic Principles and Clinical Practice, J.B. Lippincott Company, Philadelphia, PA, 94–108. 3d edition.

22. Nemerson, Y., 1992. The Tissue Factor Pathway of Blood Coagulation. *Semin Hematol* 29:170–176.

23. Monkovic, D. D., and P. B. Tracy, 1990. Functional Characterization of Human Platelet-released Factor V and its Activation by Factor Xa and Thrombin,. *J Biol Chem* 265:17132–40.

24. Hill-Eubanks, D., and P. Lollar, 1990. von Willibrand factor is a cofactor for thrombin-catalyzed cleavage of the Factor VIII light chain. *J Biol Chem* 265:17854–8.

25. Lollar, P., G. Knutson, and D. Fass, 1985. Activation of Porcine Factor VIII:C by Thrombin and Factor Xa. *Biochemistry* 24:8056–8064.

26. Gailani, D., and G. Broze Jr., 1991. Factor XI activation in a revised model of blood coagulation. *Science* 253:909–12.

27. Gailani, D., D. Ho, M. F. Sun, Q. Cheng, and P. N. Walsh, 2001. Model for a Factor IX activation complex on blood platelets: dimeric conformation of Factor XIa is essential. *Blood* 97:3117–22.

28. Sinha, D., M. Marcinkiewicz, D. Navaneetham, and P. Walsh, 2007. Macromolecular substrate-binding exosite on both the heavy and light chains of Factor XIa mediate the formation of the Michaelis Complex required for Factor IX activaiton. *Biochemistry* 46:9830–9.

29. Krishnaswamy, S., K. C. Jones, and K. G. Mann, 1988. Prothrombinase Complex Assembly. Kinetic Mechanism of Enzyme Assembly on Phospholipid Vesicles. *J Biol Chem* 263:3823–3834.

30. Mann, K. G., 1994. Prothrombin and thrombin. *In* R. Colman, J. Hirsh, V. Marder, and E. Salzman, editors, Hemostasis and Thrombosis: Basic Principles and Clinical Practice, J.B. Lippincott Company, Philadelphia, PA, 184–199. 3d edition.

31. Greengard, J., M. Heeb, E. Ersdal, P. Walsh, and J. Griffin, 1986. Binding of coagulation Factor XI to washed human platelets. *Biochemistry* 25:3884–90.

32. Monkovic, D., and P. Tracy, 1990. Activation of human Factor V by Factor Xa and thrombin. *Biochemistry* 29:1118.

33. Mann, K. G., 1987. The assembly of blood clotting complexes on membranes. *TIBS* 12:229–233.

34. Rawala-Sheikh, R., S. Ahmad, B. Ashby, and P. N. Walsh, 1990. Kinetics of coagulation Factor X activation by platelet-bound Factor IXa. *Biochemistry* 29:2606–11.

35. Nesheim, M. E., R. P. Tracy, P. B. Tracy, D. S. Boskovic, and K. G. Mann, 1992. Mathematical Simulation of Prothrominase. *Methods Enzymol.* 215:316–328.

36. Olson, S. T., R. Swanson, E. Raub-Segall, T. Bedsted, M. Sadri, M. Petitou, J.-P. Hérault, J.-M. Herbert, and I. Björk, 2004. Accelerating ability of synthetic oligosaccharides on antithrombin inhibition of proteinases of the clotting and fibrinolytic systems Comparison with heparin and low-molecular-weight heparin. *Thrombosis and haemostasis* 92:929–939.

37. Solymoss, S., M. Tucker, and P. Tracy, 1988. Kinetics of inactivation of membrane-bound Factor Va by activated protein C. Protein S modulates Factor Xa protection. *J Biol Chem* 263:14884–90.

38. Jesty, J., T.-C. Wun, and A. Lorenz, 1994. Kinetics of the Inhibition of Factor Xa and the Tissue Factor-Factor VIIa Complex by the Tissue Factor Pathway Inhibitor in the Presence and Absence of Heparin. *Biochemistry* 33:12686–12694. https://pubs.acs.org/doi/abs/10.1021/bi00208a020.

39. Maroney, S. A., and A. E. Mast, 2015. New insights into the biology of tissue factor pathway inhibitor. *J Thromb Haemost* 13:S200–S207. https://onlinelibrary.wiley.com/doi/10.1111/jth.12897.

40. Jesty, J., T. Wun, and A. Lorenz, 1994. Kinetics of the Inhibition of Factor Xa and the Tissue Factor-Factor VIIa Complex by the Tissue Factor Pathway Inhibitor in the Presence and Absence of Heparin. *Biochemistry* 33:12686–12694.

41. Griffin, J. H., 2001. Control of Coagulation Reactions. *In* E. Beutler, B. Coller, M. Lichtman, T. Kipps, and U. Seligsohn, editors, Williams Hematology, McGraw Hill, New York, 1435–1447.

42. Olson, S., I. Björk, R. Sheffer, P. Craig, J. Shore, and J. Choay, 1992. Role of the antithrombin-binding pentasaccharide in heparin acceleration of antithrombin-proteinase reactions. Resolution of the antithrombin conformational change contribution to heparin rate enhancement. *Journal of Biological Chemistry* 267:12528–12538. https://linkinghub.elsevier.com/retrieve/pii/S0021925818423095.

43. Broze, G., and J. Miletich, 1994. Biochemistry and Physiology of Protein C, Protein S, and Thrombomodulin. *In* R. W. Colman, J. Hirsh, V. J. Marder, and E. W. Salzman, editors, Hemostasis and Thrombosis: Basic Principles and Clinical Practice, Lippincott Company, Philadelpha, 259–276.

44. Turitto, V. T., and H. R. Baumgartner, 1979. Platelet Interaction with Subendothelium in Flowing Rabbit Blood: Effect of Blood Shear Rate. *Microvasc. Res.* 17:38–54.

45. Turitto, V. T., H. J. Weiss, and H. R. Baumgartner, 1980. The Effect of Shear Rate on Platelet Interaction with Subendothelium Exposed to Citrated Human Blood. *Microvasc. Res.* 19:352–365.

46. Kuharsky, A., and A. Fogelson, 2001. Surface-mediated Control of Blood Coagulation: The Role of Binding Site Densities and Platelet Deposition. *Biophys J* 80:1050–1074.

47. Gear, A. R. L., 1994. Platelet adhesion, shape change, and aggregation: rapid initiation and signal transduction events. *Can. J. Physiol. Pharmacol.* 72:285–94.

48. Byun, J.-H., I.-S. Jang, J. W. Kim, and E.-H. Koh, 2016. Establishing the heparin therapeutic range using aPTT and anti-Xa measurements for monitoring unfractionated heparin therapy. *Blood Research* 51:171. https://synapse.koreamed.org/DOIx.php?id=10.5045/br.2016.51.3.171.

49. Bangham, D., and M. V. Mussett, 1959. The Second International Standard for Heparin. *Bulletin of the World Health Organization* 20:1201.

50. Linhardt, R. J., 2016. Heparin and anticoagulation. *Frontiers in Bioscience* 21:1372–1392. https://imrpress.com/journal/FBL/21/7/10.2741/4462.

51. Hirsh, J., and M. Levine, 1992. Low molecular weight heparin. *Blood* 79:1–17. https://ashpublications.org/blood/article/79/1/1/173040/Low-molecular-weight-heparin.

52. Link, K. G., M. T. Stobb, J. Di Paola, K. B. Neeves, A. L. Fogelson, S. S. Sindi, and K. Leiderman, 2018. A local and global sensitivity analysis of a mathematical model of coagulation and platelet deposition under flow. *PLoS ONE* 13:e0200917. https://dx.plos.org/10.1371/journal.pone.0200917.
