# Supplementary material for: Inhibition of platelet-surface-bound proteins during coagulation under flow II: Antithrombin and heparin
Source: Biophys J. 2022 Nov 2;122(1):230–40. doi: 10.1016/j.bpj.2022.10.038 (PMC9822793; doi:10.1016/j.bpj.2022.10.038)
Supplement: Document S2. Article plus supporting material [file mmc2.pdf]

Biophysical Journal
Article

Biophysical Society

Inhibition of platelet-surface-bound proteins during coagulation under flow II: Antithrombin and heparin

Kenji Miyazawa,[1] Aaron L. Fogelson,[2,3] and Karin Leiderman[4,5,*]

[1]Quantitative Biosciences and Engineering, Colorado School of Mines, Golden, Colorado; [2]Department of Mathematics, University of Utah, Salt Lake City, Utah; [3]Department of Biomedical Engineering, University of Utah, Salt Lake City, Utah; [4]Mathematics Department, University of North Carolina at Chapel Hill, Chapel Hill, North Carolina; and [5]Computational Medicine Program, University of North Carolina at Chapel Hill, Chapel Hill, North Carolina

ABSTRACT Blood coagulation is a self-repair process regulated by activated platelet surfaces, clotting factors, and inhibitors. Antithrombin (AT) is one such inhibitor that impedes coagulation by targeting and inactivating several key coagulation enzymes. The effect of AT is greatly enhanced in the presence of heparin, a common anticoagulant drug. When heparin binds to AT, it either bridges with the target enzyme or induces allosteric changes in AT leading to more favorable binding with the target enzyme. AT inhibition of fluid-phase enzymes caused little suppression of thrombin generation in our previous mathematical models of blood coagulation under flow. This is because in that model, flow itself was a greater inhibitor of the fluid-phase enzymes than AT. From clinical observations, it is clear that AT and heparin should have strong inhibitory effects on thrombin generation, and thus we hypothesized that AT could be inhibiting enzymes bound to activated platelet surfaces that are not subject to being washed away by flow. We extended our mathematical model to include the relevant reactions of AT inhibition at the activated platelet surfaces as well as those for unfractionated heparin and a low molecular weight heparin. Our results show that AT alone is only an effective inhibitor at low tissue factor densities, but in the presence of heparin, it can greatly alter, and in some cases shut down, thrombin generation. Additionally, we studied each target enzyme separately and found that inactivation of no single enzyme could substantially suppress thrombin generation.

SIGNIFICANCE We have developed a novel mathematical model of flow-mediated coagulation that is sensitive to changes in antithrombin levels, especially at low tissue factor densities, and to antithrombin and heparin for all tissue factor densities examined. The sensitivity of the system is entirely due to inhibition reactions that occur on activated platelet surfaces. The model we present here can serve as tool to explore other combinations of target enzymes for optimal inhibition and anticoagulation strategies.

INTRODUCTION

Antithrombin (AT) is an inhibitor of blood coagulation that belongs to the serine protease inhibitor (serpin) superfamily (1) and is found in plasma (2,3). It is continually generated in the liver to maintain a normal level in plasma of approximately 2.4 μM (4). The anticoagulant activity of AT involves the irreversible inactivation (5) of four key serine proteases generated during coagulation: FIXa, FXa, FXIa, and thrombin. Inactivation of FIXa reduces tenase formation (6); inactivation of FXa reduces prothrombinase formation (4); inactivation of FXIa weakens its positive feedback (7); and inactivation of thrombin reduces positive feedback, platelet activation, and overall clot formation (4). A deficiency in AT can lead to excessive thrombin generation (8), which is associated with venous thromboembolism (9).

Although AT is thought to be an important inhibitor by itself, the range of its inactivation rates for coagulation enzymes is quite large, from 10,000 to 14,000/(M•s), which covers measurements at both 25°C and 37°C (10,11). Its anticoagulant activity is significantly enhanced in the presence of heparin, which is the oldest anticoagulant drug used in clinical medicine, discovered first by Mclean in

Submitted January 9, 2022, and accepted for publication October 26, 2022.

*Correspondence: karin.leiderman@unc.edu

Editor: Mark Alber.

https://doi.org/10.1016/j.bpj.2022.10.038

© 2022 Biophysical Society.

This is an open access article under the CC BY license (http://creativecommons.org/licenses/by/4.0/).

1916 (12,13). Heparin is a naturally occurring glycosaminoglycan that exerts its anticoagulant properties by forming a complex with AT and facilitating an enhanced inhibitory effect of AT on activated coagulation factors (14). Unfractionated heparin (UFH) is a minimally processed form of the natural heparin and thus was the first to be used in medicine. The structure and length of UFH were found to be quite heterogeneous, and this led to unwanted and unpredictable side effects and the need for continuous monitoring during its use as an anticoagulant drug. Further processing to shorten and standardize lengths was desirable to achieve more predictable outcomes (12). These low molecular weight heparins (LMWHs) are derived from UFH using different but controlled manufacturing processes that lead to mean molecular weights that are less than half that of UFH (15,16). Compared with UFH, LMWHs have longer circulating half-lives and higher bioavailability, more predictable outcomes, and less monitoring and are thus largely favored for clinical use (17–20).

The anticoagulant activity of heparin is its ability to accelerate the inactivation of activated coagulation factors via AT. This activity and the molecular weight of heparin have an interesting and complex relationship. It is now understood that there are two main mechanisms for heparin's anticoagulant activity. There is allosteric activation of AT by heparin, which alters the structure of AT and enhances its recognition by the various coagulation factors (21,22), and there is also the fact that heparin provides a template to which both AT and coagulation factors can bind and form a ternary bridging complex (23,24). It is thought that the longer the heparin, the more significant the bridging effect can be (11). The allosteric activation mechanism works to inactivate FIXa and FXa but not thrombin or FXIa (21,22). The bridging mechanism can potentially affect all four species, to varying degrees, but thrombin inhibition is solely dependent on this mechanism; thrombin inhibition is significantly enhanced by UFH when there is sufficient room on it for thrombin to bind but much less so by LMWHs (23,24). Enhanced inhibition of FXIa occurs mainly by the bridging mechanism but with a slight variation: FXIa has two binding sites for heparin, one noncatalytic site through which the bridging with AT can occur, and a catalytic site through which heparin can trigger allosteric modulation of FXIa functional activity (in contrast to the allosteric effects on AT described above) (7,25). To summarize, both LMWH and UFH can affect all four enzymes, but they do so to varying degrees, depending on their lengths. In this study, we used a mathematical model of flow-mediated coagulation to explore the effects of LMWH and UFH on inhibition of each individual enzyme and all of them together. We chose to use Nadroparin as the LMWH since there are literature values for its kinetic rate constants along with rates for UFH within the same experimental study (11).

In our companion study focused on tissue factor pathway inhibitor (TFPI), we showed that platelet surfaces played an important yet indirect role in the inhibitory mechanisms of TFPI (26). Platelet-surface-bound enzymes are necessary in coagulation since they help localize coagulation to the site of injury and are many orders of magnitude more efficient than their fluid-phase analogs. However, they are limited by the number of activated platelets and the corresponding binding sites on those platelets' surfaces. We hypothesized that direct binding of these platelet-bound enzymes by fluid-phase inhibitors would have a stronger overall inhibitory effect on coagulation compared with their binding of fluid-phase enzymes since the fluid-phase complexes are subject to being washed away by flow. Our simulation results confirmed our hypothesis in the case of TFPI, and this led us to hypothesize that AT (with and without heparin) may be working in a similar manner.

To investigate this, we extended the model presented in our companion paper (26) to include surface-dependent inactivation by AT. In our previous model, AT could directly bind and inactivate fluid-phase enzymes only, FIXa, FXa, and thrombin, but these inhibitory reactions showed little to no effect on thrombin generation (27) except under the near-stasis conditions of venous thrombosis (28). Here, we have included the inactivation of FXa, FIXa, and thrombin bound to the platelet surface and, additionally, the inactivation of FXIa in both fluid phase and bound to the platelet surface. Heparin (UFH and LMWH) was explicitly introduced into the model to examine its effects on anticoagulant activity via AT. Our results demonstrated that the inclusion of surface-dependent inactivation magnified the effects of AT and did so to a greater extent when heparin was present. AT in the new version of the model altered thrombin generation at a low, but not high, TF and almost entirely through its effect on surface-bound enzymes. In the presence of heparin, thrombin generation could be significantly delayed and reduced, and these behaviors, too, were completely dependent on direct inhibition of the surface-bound enzymes. In summary, we tested two targets for inhibition of thrombin generation under flow: fluid phase and platelet-bound enzymes. We identified direct binding to platelet-surface-bound enzymes by fluid-phase inhibitors to be the primary mechanism for effective overall inhibition of thrombin generation under flow.

MATERIALS AND METHODS

Mathematical model review

Here, we give a brief review of our previously developed mathematical model of flow-mediated coagulation (29–31) and the details of the extensions we have made to it. More details about this model and its sensitivity to parameters can be found elsewhere (27). The model simulates the coagulation reactions occurring in a small reaction zone (RZ) above an injury where TF in the

subendothelium (SE) is exposed (see model schematic in Fig. S1). Clotting factors and platelets are transported into and out of the RZ by a combination of flow and diffusion using a mass transfer coefficient whose value is a function of vessel and injury size, the flow's shear rate, and the species' diffusivity. Clotting factor concentrations in the RZ change due to their involvement in the coagulation reactions and by transport in and out of the zone. Similarly, platelet concentrations change as platelets adhere to the injured wall and become activated and as other platelets are transported in and out of the zone. As platelets build up in the RZ, the height and volume of the RZ increase, with the volume of plasma and concentration of platelets in it changing accordingly. The concentration of each species in the RZ is tracked with an ordinary differential equation; this choice relies on the assumption that each species is uniformly distributed (well mixed) within the RZ. An additional well-mixed endothelial zone (EZ) is located adjacent to the RZ in the direction perpendicular to the flow, with height equal to that of the RZ and the width dependent on the flow shear rate and protein diffusion coefficients. The EZ is where active protein C is produced by a complex formed by thrombomodulin in that zone and thrombin, which has diffused to the EZ from the RZ. This active protein C either diffuses into the RZ or is carried away by the flow.

There are three forms of platelets in the model: unactivated platelets that exist in the plasma phase, activated platelets that are directly attached to the SE, and activated platelets in the thrombus that are not directly attached to the SE. Activation of platelets is achieved by contact with the SE, by interaction with thrombin, or by exposure to already activated platelets (this is an indirect way to model release of agonists from platelet stores). Activated platelets provide the membrane surface necessary for coagulation factors to bind and react. Each activated platelet expresses specified types and numbers of binding sites to which coagulation proteins can selectively bind.

In our companion paper (26), we extended the model to include novel TFPI-mediated inhibition reactions that allow TFPI to act directly on activated platelet-surface-bound species. These reactions enabled enhanced inhibitory effects compared with our previous version(s) of the model. Here, we included an additional extension to incorporate the inactivation of coagulation enzymes by AT and the AT-heparin complex, specifically on the platelet surface.

Extension in the new model

The new AT/heparin-mediated inactivation reactions include the inactivation of FXa, FIXa, and thrombin bound to the platelet surface and inactivation of FXIa both in the fluid phase and bound to the platelet surface. We previously assumed that the AT-mediated inactivation occurred with a pseudo first-order reaction rate due to the high concentration of AT in the plasma. To track intermediate species more carefully in this study, we have explicitly introduced both AT and heparin as new species. We allow the interaction between AT and heparin to include the formation of complexes, which then have an accelerated inactivation rate that depends on the heparin type (UFH or LMWH). The standard concentration of AT is set to 2.4 μM, and the concentration of heparin depends on the type of heparin (refer to Section S3). The kinetic rates we used are based on the experimentally observed data (11). Additionally, we allow AT to diffuse into the endothelial region and further inhibit the FXa, FIXa, and thrombin that reside there.

All of the new reactions involving AT and heparin are sketched in Fig. 1, and the corresponding reactions are listed in Tables 1 and 2. In Eq. 1, we show the evolution equation for the concentration of one of the new species as an

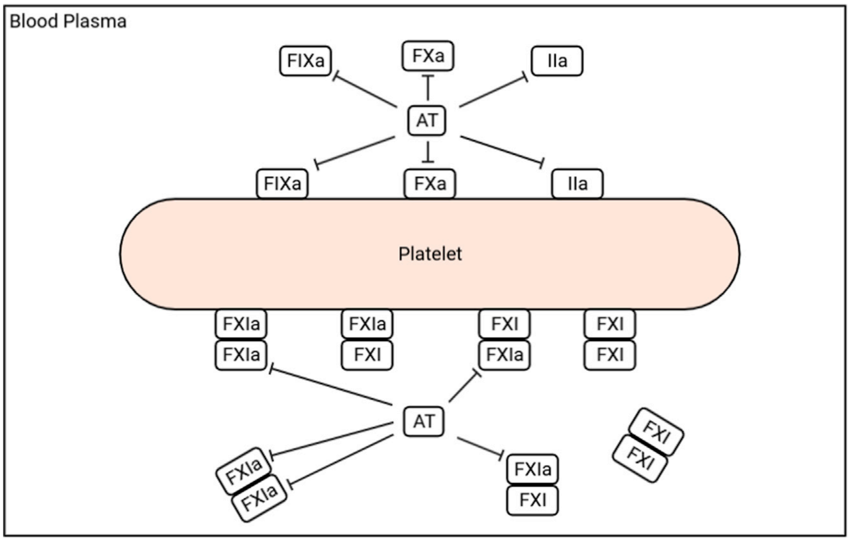

FIGURE 1 Newly added reactions involving AT, FIXa, FXa, thrombin (IIa), and FXIa. Inactivation reactions are indicated with T-shaped arrows. FXI/FXIa exists as a dimer form, and only the exposed end of FXIa can be inactivated by AT. To see this figure in color, go online.

TABLE 1 List of reactions added on top of the extension with newly added TFPI-meditaed reactions, and their kinetic rate constants, with literature references

Reaction list							
Reaction no.	Reactants	Products	k^+ ($M^{-1}s^{-1}$)	k^- (s^{-1})	k^+_{LMWH} ($M^{-1}s^{-1}$)	k^+_{UFH} ($M^{-1}s^{-1}$)	Note
1	AT+ E_9^m	AT: E_9^m	4.8 * 10^2	–	–	–	a
2	AT+ E_9^{m*}	AT: E_9^{m*}	4.8 * 10^2	–	–	–	b
3	AT+ E_{10}^m	AT: E_{10}^m	3.5 * 10^3	–	–	–	a
4	AT+ E_2^m	AT: E_2^m	1.4 * 10^4	–	–	–	a
5	AT + E_{11}:Z_{11}	AT:E_{11}:Z_{11}	2.4 * 10^2	–	–	–	a
6	AT + E_{11}:E_{11}	AT:E_{11}:E_{11}	2.4 * 10^2	–	–	–	a
7	AT + AT:E_{11}:E_{11}	AT:E_{11}:E_{11}:AT	2.4 * 10^2	–	–	–	a
8	AT + E_{11}: Z_{11}^m	AT:E_{11}: Z_{11}^m	2.4 * 10^2	–	–	–	a
9	AT + E_{11}: E_{11}^m	AT:E_{11}: E_{11}^m	2.4 * 10^2	–	–	–	a
10	AT + Hep	ATH	1	2.77 * 10^7	–	–	c
11	ATH+ E_9^m	ATH: E_9^m	–	–	5 * 10^5	6.2 * 10^6	d
12	ATH+ E_9^{m*}	ATH: E_9^{m*}	–	–	5 * 10^5	6.2 * 10^6	b
13	ATH+ E_{10}^m	ATH: E_{10}^m	–	–	1.3 * 10^6	6.6 * 10^6	d
14	ATH+ E_2^m	ATH: E_2^m	–	–	5.3 * 10^6	4.7 * 10^7	d
15	ATH + E_{11}:Z_{11}	ATH:E_{11}:Z_{11}	–	–	1 * 10^4	1.8 * 10^5	d
16	ATH + E_{11}:E_{11}	ATH:E_{11}:E_{11}	–	–	1 * 10^4	1.8 * 10^5	d
17	ATH + ATH:E_{11}:E_{11}	ATH:E_{11}:E_{11}:AT	–	–	1 * 10^4	1.8 * 10^5	d
18	ATH + E_{11}: Z_{11}^m	ATH:E_{11}: Z_{11}^m	–	–	1 * 10^4	1.8 * 10^5	d
19	ATH + E_{11}: E_{11}^m	ATH:E_{11}: E_{11}^m	–	–	1 * 10^4	1.8 * 10^5	d

k^+ shows forward reaction rate, and k^- shows backward reaction rate. Subscript LMWH and UFH indicates reaction rates when AT is bound to either LWMH or UFH, respectively.

[a]For inhibition of FIXa by AT, $k^+ = 4.8 * 10^2$. For inhibition of FXa by AT, $k^+ = 3.5 * 10^3$. For inhibition of thrombin by AT, $k^+ = 1.4 * 10^4$. And for inhibition of FXIa by AT, $k^+ = 2.4 * 10^2$, from Olson et al. (10).

[b]We assume the inhibition rate of FIXa and binding rate to platelet on specific binding site are the same as the normal binding site.

[c]Binding of heparin to antithrombin, $K_D = 36$ nM for LWMH, and $K_D = 9.7$ nM for UFH, from Olson et al. (21).

[d]Accelerated inhibition of FIXa by AT:UFH complex, $k^+ = 6.2 * 10^6$, and AT:LMWH complex, $k^+ = 5 * 10^5$. For FXa by AT:UFH complex, $k^+ = 6.6 * 10^6$, and AT:LMWH complex, $k^+ = 1.3 * 10^6$. For thrombin by AT:UFH complex, $k^+ = 4.7 * 10^7$, and AT:LMWH complex, $k^+ = 5.3 * 10^6$. And for FXIa by AT:UFH complex, $k^+ = 1.8 * 10^5$, and AT:LMWH complex, $k^+ = 1 * 10^4$, from Olson et al. (11).

example of the nature of the model's equations. A full listing of the model equations and parameter values is given in Section S2. Note that the ordinary differential equations labeled 1–104 comprise the model from our companion paper (26), and the remaining equations represent the new species in this paper.

The model uses the following notation: Z_i and E_i refer to a specific zymogen or procofactor species and the corresponding enzyme or cofactor species when they are in the plasma, Z_i^m and E_i^m refer to the surface-bound versions of these proteins (e.g., E_7^m refers to the TF:VIIa complex on the SE), and E_8 and E_8^m refer to factor VIIIa in the plasma and bound to a platelet surface, respectively. The concentrations of the proteins are denoted similarly but with lower case z and e. So, e_8^m is the concentration of platelet-bound factor VIIIa. The symbols TF, P_2, P_5, P_8, P_9, P_{10}, and P_{11} are used to denote TF and the platelet binding sites for prothrombin, FV/FVa, FVIII/FVIIIa, FIX/FIXa, FX/FXa, and FXI, respectively. For the platelet binding sites specific to thrombin, factor IXa, and factor XIa, we use the symbols P_2^*, P_9^*, and P_{11}^*. The concentrations of binding sites are indicated similarly but with lower case p. We denote the complex of Z_i and E_j by Z_i:E_j and its concentration by [Z_i:E_j]; so, for example, $AT : E_9^m$ denotes AT bound to platelet-bound Factor IXa, and $[AT : E_9^m]$ refers to its concentration.

Inhibition of FXa/FIXa/thrombin by AT

In our previous models (29–31), AT inactivated FXa, FIXa, and thrombin in the fluid phase only. Now, AT can additionally inactivate all of these enzymes when they are bound to the platelet surface (reactions 1–4 in Table 1). We assume the inactivation rates to be the same for both fluid-phase and platelet-bound enzymes and allow the inactivated AT-enzyme complex to bind/unbind from the platelet surface with kinetic rates that match those of the enzyme itself. We also assume that platelet-bound enzymes, which are also bound to AT, occupy their corresponding binding site. For example, one AT:FXa complex on the membrane, $AT : E_{10}^m$, takes up one FX/FXa binding site.

Inactivation of FXIa by AT

In our new model, we introduce the inactivation of FXIa by AT in both fluid- and platelet-bound phases (reactions 5–9 in Table 1). Since FXI and FXIa are dimers, we have included inactivation of only the FXIa part of the several dimeric forms that involve FXI and FXIa: FXI:FXI, FXI:FXIa, and FXIa:FXIa in the fluid. Each of these can also be bound to the platelet surface. We assume that when FXIa is bound

TABLE 2 List of platelet-binding reactions added on top of the extension with newly added TFPI-mediated reactions, and their kinetic rate constants, with literature references

Reaction list

Reaction no.	Reactants	Products	k^{on} ($M^{-1}s^{-1}$)	k^{off} (s^{-1})	Note
20	AT:E_9+P_9	AT: E_9^m	1 * 10^7	2.5 * 10^{-2}	a
21	AT:E_9+ P_9^*	AT: E_9^{m*}	1 * 10^7	2.5 * 10^{-2}	b
22	AT:E_{10} + P_{10}	AT: E_{10}^m	1 * 10^7	2.5 * 10^{-2}	c
23	AT:E_2+ P_2^*	AT: E_2^m	1 * 10^7	5.9	d
24	AT:E_{11}:Z_{11} + P_{11}	AT:E_{11}: Z_{11}^m	1 * 10^7	0.1	e
25	AT:E_{11}:E_{11}+ P_{11}^*	AT:E_{11}: E_{11}^m	1 * 10^7	0.017	f
26	ATH:E_9+P_9	ATH: E_9^m	1 * 10^7	2.5 * 10^{-2}	a
27	ATH:E_9+ P_9^*	ATH: E_9^{m*}	1 * 10^7	2.5 * 10^{-2}	b
28	ATH:E_{10} + P_{10}	ATH: E_{10}^m	1 * 10^7	2.5 * 10^{-2}	c
29	ATH:E_2+ P_2^*	ATH: E_2^m	1 * 10^7	5.9	d
30	ATH:E_{11}:Z_{11} + P_{11}	ATH:E_{11}: Z_{11}^m	1 * 10^7	0.1	e
31	ATH:E_{11}:E_{11}+ P_{11}^*	ATH:E_{11}: E_{11}^m	1 * 10^7	0.017	f

k^{on} shows binding rate and k^{off} shows unbinding reactions rate.

[a]Binding of FIXa to platelet surface, K_D = 2.5 * 10^{-9}, from Ahmad et al. (32).

[b]We assume the inhibition rate of FIXa and binding rate to platelet on specific binding site are the same as the normal binding site.

[c]Binding of FXa to platelet surface, K_D = 2.5 * 10^{-9}, from Walsh et al. (33).

[d]Binding of thrombin to platelet surface, K_D = 5.9 * 10^{-7}, from Mann et al. (31).

[e]Binding of FIX to platelet surface, K_D = 1 * 10^{-7}, from Greengard et al. (34).

[f]Binding of FIXa to platelet surface, K_D = 1.7 * 10^{-7}, from Miller et al. (35).

directly to a platelet binding site, it does not participate in any reactions and that AT can only inhibit the free FXIa end of the dimer that is not directly bound. For example, AT can bind and inhibit FXIa in the FXIa:FXIa complex in the fluid phase on either or both ends (reaction 6 in Table 1) but can only inhibit the FXIa that is exposed to the fluid when the other end is bound to the platelet (reactions 8 and 9 in Table 1, respectively). The detailed FXI/FXIa complexes and available inhibition sites are also shown in the reaction schematic in Fig. 1.

Introduction of heparin

Heparin is introduced as a new species with a fixed upstream concentration set to 253 and 759 nM for UFH and LMWH, respectively, based on the recommended therapeutic range 0.3–0.7 U/mL (36). The conversion of heparin potency to molar concentration can be found in Section S3. We also varied this concentration to understand how this affects heparin's impact.

Inactivation by AT:Heparin complex

We assume that AT can bind to heparin and form the complex we call *ATH* (reaction 10 in Table 1). The AT:Heparin complex inactivates FXa, FIXa, FXIa, and thrombin in either the fluid- or platelet-bound phase by direct binding (reactions 11–19 in Table 1). When AT is bound to LMWH, we simply change the kinetic rate constants to represent Nadroparin, which greatly accelerates the inactivation of FIXa (by 3 orders of magnitude), accelerates inactivation of FXa and thrombin to a lesser extent (by a factor of 370), and accelerates FXIa inactivation by a yet smaller amount (by a factor of 40). When AT is bound to UFH, we use the reaction kinetic reported from the same experiment, which indicates that it accelerates inactivation of all of the targeted enzymes (750–13,000 times increase) more potently than does LMWH. Platelet-bound enzymes that are inactivated by AT:heparin complexes can also bind/unbind from the platelet surface and, while bound, occupy the corresponding enzyme binding site on the platelet (reactions 20–31 in Table 2).

Example equation

Newly added reactions listed in Table 1 were expressed mathematically using the law of mass action. For example, the following equation describes the rate of change of the platelet-bound concentration of FXa bound to AT. It accounts for the binding and unbinding of the complex FXa:AT with the platelet surface and the direct binding of AT from the fluid to platelet-bound FXa:

$$\frac{d\left[E_{10}^{m}:AT\right]}{dt} = \underbrace{k_{10}^{\mathrm{on}}\left[E_{10}:AT\right]p_{10}^{\mathrm{avail}}}_{\text{FXa:AT binding to platelet}} - \underbrace{k_{10}^{\mathrm{off}}\left[E_{10}^{m}:AT\right]}_{\text{FXa:AT unbinding from platelet}} + \underbrace{k_{e_{10}^{m}:\mathrm{AT}}^{+}\cdot e_{10}^{m}\cdot AT}_{\cdot\text{Inactivation of platelet-bound FXa by AT}\cdot} \tag{1}$$

Here, p_{10}^{avail} is the concentration of FX/FXa binding sites on activated platelets that are not occupied by an FX- or FXa-containing species.

RESULTS

TF and shear rate dependence

Here, we examined how the variation in TF density and shear rate affected thrombin production with the new AT inhibitory mechanisms at the platelet surface and compared the outcomes with those without that surface inhibition. For various TF densities in the range 0–30 fmol/cm^2 and for shear rates 100, 500, and 1,500/s, we performed simulations with and without surface inhibition (denoted "SI" in the figure). We looked at two output metrics, the lag time, which we define as the time point at the thrombin concentration first reaches 1 nM, and the thrombin concentration at 10 min. Fig. 2 *A* shows the lag times, and Fig. 2 *B* shows the thrombin concentrations at 10 min.

With and without the surface inhibition, the lag time decreased as the TF density increased and/or the shear rate decreased. These behaviors occur because a higher TF density provides a larger initial stimulus and decreasing the shear rate slows the loss of essential enzymes from the RZ. Also, the thrombin concentrations at 10 min increased with the TF density, sharply at low TF densities and more gradually at high ones. In fact, the results indicated a threshold dependence on TF density in all cases examined. (We refer to curves of thrombin at 10 min versus TF density as threshold curves.) The thrombin concentration at 10 min, however, was only very slightly affected by the shear rate (Fig. 2 *B*). In summary, the effects of TF density and shear rate remain qualitatively the same in the presence of the new AT reactions without heparin-mediated acceleration.

Influence of AT level on thrombin generation

Next, we compared how thrombin generation was affected by various AT levels at low versus high TF density. We varied the AT level from 0% to 200% of its physiological concentration (2.4 μM) and used 4 and 15 fmol/cm^2 for the low and high TF densities, respectively. Fig. 3 shows plots of thrombin generation with (Fig. 3, *C* and *D*) and without (Fig. 3, *A* and *B*) surface inhibition by AT for low TF (Fig. 3, *A* and *C*) and high TF (Fig. 3, *B* and *D*). In the absence of the surface AT reactions, there is no to little change in the thrombin generation for all levels of AT (Fig. 3, *A* versus *B*). When the AT surface reactions are present, thrombin generation is significantly delayed at the low, but not the high, TF density (Fig. 3, *C* and *D*). Another observation is that the concentration of thrombin after 20 min of activity remains almost the same in all cases; similar to our TFPI study, the AT affects mainly the timing of the thrombin burst. In summary, surface-dependent inactivation by AT affects thrombin generation, but this is more prominent at a low TF density. Since measurements of plasma levels of thrombin-AT (TAT) are used clinically, we calculated the instantaneous rates of generation of TAT complexes (in the plasma and on the platelet surfaces) and their removal by flow for various levels of AT. We found that more TAT was generated than carried away by flow, with the majority staying bound to platelet surfaces, as shown in Fig. S8.

Influence of heparin level on thrombin generation

The effect of different heparin concentrations was also examined. The thrombin time courses shown in Fig. 4 were generated for different heparin concentrations (0.1%, 10%, 50%, and 100%) for both LWMH (where 100% = 253 nM) and UFH (for which 100% = 759 nM). The values for 100% were based on recommended dosages, and the conversions and references are in the supporting material. As the level of heparin was increased, the delay in thrombin generation was increased, and at the therapeutic concentration, both types of heparins prevented the thrombin concentration from reaching 1 nM. As expected, UFH had a higher overall inhibitory effect. Our new model exhibits the distinct and clinically observed effects on anticoagulant activity of LMWH and UFH.

Effects of surface-dependent AT inactivation on thrombin, FIXa, FXa, and FXIa

To study the individual enzymes and the effect of inactivation on their concentrations, we tracked the time courses of thrombin, FXa, FIXa, and FXIa. Fig. 5 shows plots of the sum of both the fluid-phase and platelet-bound species. We examined these concentrations in the absence and presence of the surface-dependent inactivation by AT, and under the condition of no heparin, with LWMH or with UFH at 25% of their therapeutic concentrations. For all species, the cases without surface-dependent inactivation by AT or ATH (antithrombin bound to heparin) showed almost indistinguishable curves, meaning that AT and ATH were not effective at reducing enzyme concentrations when

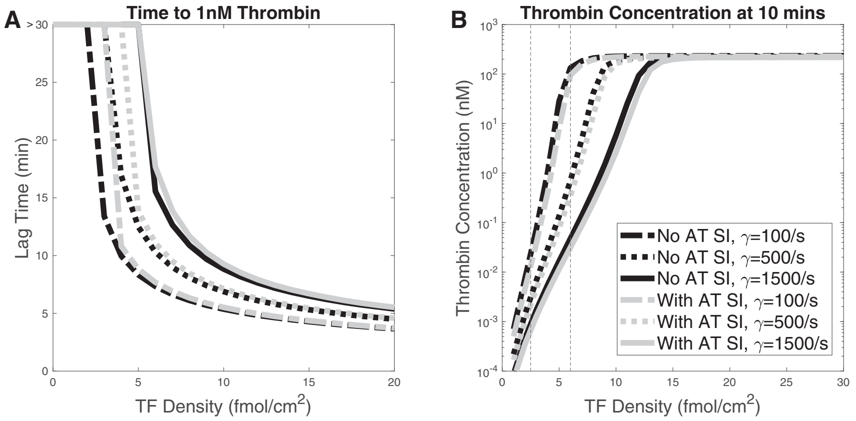

FIGURE 2 (*A*) Lag time and (*B*) thrombin concentration after 10 min of activity, with variations in TF density and shear rate. TF density was varied from 0 to 30 fmol/cm^2, and shear rate was either 100, 500, or 1,500/s. "SI" in the legend represents surface-dependent inactivation. Black lines represent results with AT-mediated inactivation of fluid-phase enzymes and noinactivation of surface-bound enzymes, and gray lines represent the results with inactivation of both fluid-phase and surface-bound enzymes by AT. Vertical dashed lines indicate TF densities of interest. Flat curves at >30 min indicate a lag time larger than 30 min in (*A*).

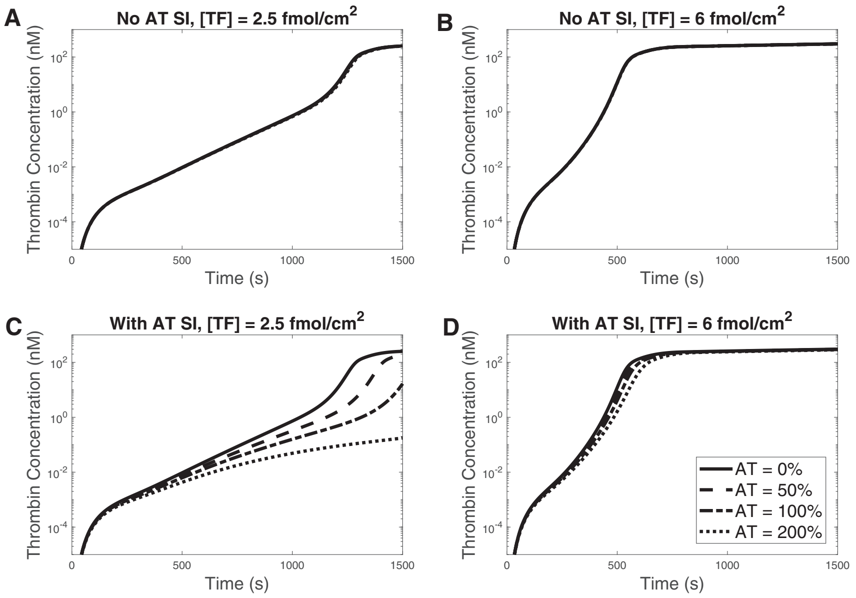

FIGURE 3 Thrombin time courses for various AT levels, with and without surface-dependent inactivation reactions. SI denotes surface-mediated inactivation. Shown in the top row of the figure are TF = (*A*) 2.5 and (*B*) 6 fmol/cm^2, each without SI. Shown in the bottom row of the figure are TF = (*C*) 2.5 and (*D*) 6 fmol/cm^2, each with SI. AT level was 0%, 50%, 100%, or 200% of 2.4 μM. Shear rate was set to 100/s for all simulations.

inactivating fluid-phase enzymes only. In the presence of surface-dependent inactivation by AT, but in the absence of heparin, the concentrations of all species look similar to the case with no surface-dependent inactivation. With surface-dependent inactivation, both the LMWH and UFH substantially reduced the concentrations of all enzymes, with the strongest effects from UFH. These results collectively show that the surface-dependent inhibition reactions are critical and necessary to induce observable effects of heparin on its target enzymes.

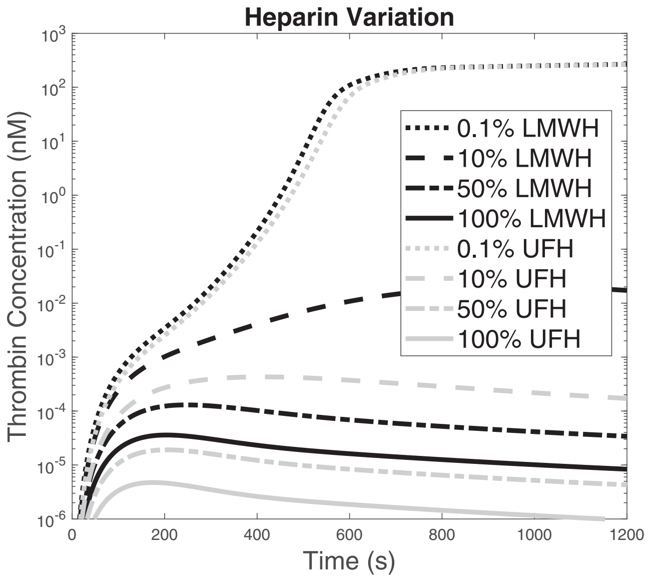

FIGURE 4 Thrombin generation with TF = 6 fmol/cm^2 and varied levels of LWMH and UFH. Thrombin generation in the presence of LMWH and UFH are shown in black and gray lines, respectively. The heparin levels were varied as 0.1%, 10%, 50%, or 100% of a standard therapeutic concentration.

Examination of the major inactivation reactions

To understand the effects of inactivating each enzyme alone, we separately kept individual inactivation reactions "turned on" while "turning off" the remaining reactions; turning off a reaction here means that we set the corresponding association rates to zero. For example, to focus on the effects of inactivating FIXa alone, we keep AT inactivation of FIXa turned on while setting AT association rates for FXa, FXIa, and thrombin to zero. For these studies, we used a TF density of 15 fmol/cm^2 and a shear rate of 100/s. Fig. 6 shows the resulting thrombin time courses under the influence of either LMWH (Fig. 6 *A*) or UFH (Fig. 6 *B*). Simulations with LMWH and UFH showed similar trends. In both cases, inactivating FXIa had a negligible effect. Inactivating either FIXa, FXa, or thrombin increased the lag time; with LMWH, the lag times were increased by about 200 s, and with UFH, they were increased by about 400 s for FIXa and thrombin inactivation and by about 800 s for FXa inactivation. The thrombin concentrations after 20 min were about the same for individual inactivation of FIXa, FXa, and FXIa, around 150 nM. For inactivation of thrombin, the thrombin concentration after 20 min is reduced to about 100 nM with LMWH and about 20 nM with UFH; this is due to a reduction in both positive feedback and the direct inactivation itself. When LMWH and UFH are working to their full potential, i.e., they are inactivating all four enzymes to their respective degrees, the thrombin generation is essentially shut off, with concentrations that never even reach 1 pM. In summary, our model

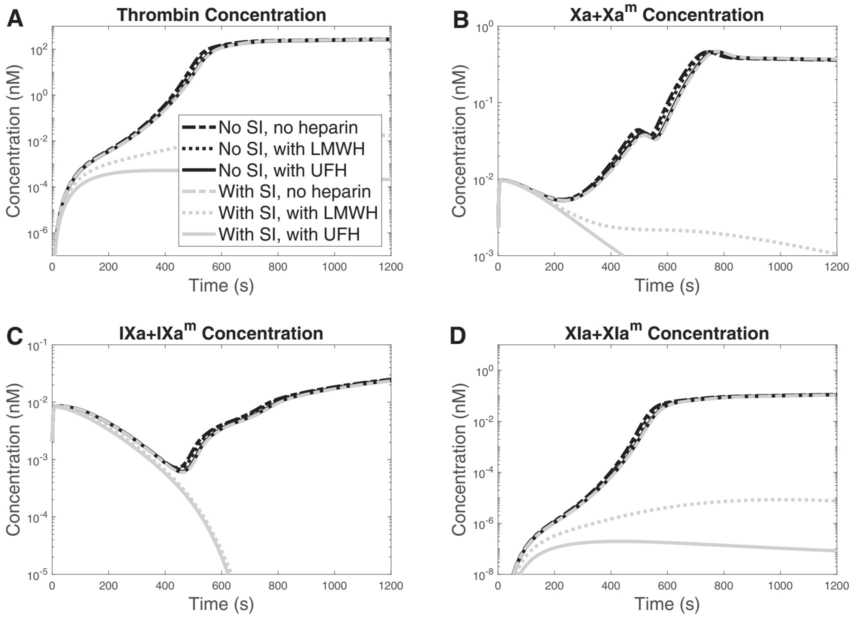

FIGURE 5 Concentration time courses of (*A*) thrombin, (*B*) FXa, fluid phase and surface bound, (*C*) FIXa, fluid phase and surface bound, and (*D*) FXIa, fluid phase and surface bound, each with a TF density of 15 fmol/cm^2 and a shear rate of 100/s. Simulations were performed with or without surface-dependent AT inactivation reactions (SI, surface-mediated inactivation), which are shown as gray and black curves, respectively, and for no heparin, LMWH, or UFH, shown by varying line styles.

shows that inactivation of any of the single target enzymes alone is not enough to prevent robust thrombin generation; inhibiting multiple targets together enables accumulative inhibition to completely extinguish thrombin generation.

DISCUSSION

In our previous mathematical models of flow-mediated coagulation (29–31), AT inactivated FIXa, FXa, and thrombin in the fluid phase only, and this had little effect on thrombin production since the fluid-phase species, whether inactivated or not, were subject to flow and could easily be washed away. Our extended model, which includes direct binding of fluid-phase AT to platelet-bound species, shows a new sensitivity of the model coagulation system to AT. In particular, we find that AT can dramatically increase the lag time of thrombin generation at low TF density through this surface-dependent inhibition mechanism. However, at high TF, even when AT was increased to 200% of its normal concentration, the changes in thrombin generation are slight. Addition of these new reactions does not significantly alter the TF density threshold or shear rate dependence. We found only small increases in the lag time and slight changes in thrombin concentration after 10 min of clotting activity, when compared with simulations run in the absence of the AT-mediated inactivation reactions, over a wide range of TF densities. As observed in our previous studies (29–31), increasing the flow shear rate increases the lag time and reduces the thrombin concentration after 10 min of clotting activity.

The presence of heparin greatly magnifies the sensitivity of the system to AT, and this sensitivity is due entirely to the direct binding of platelet-bound species by fluid-phase ATH. When the AT-mediated inactivation of platelet-surface-bound enzymes is turned off, neither type of heparin has any noticeable effect on the system. This highlights the

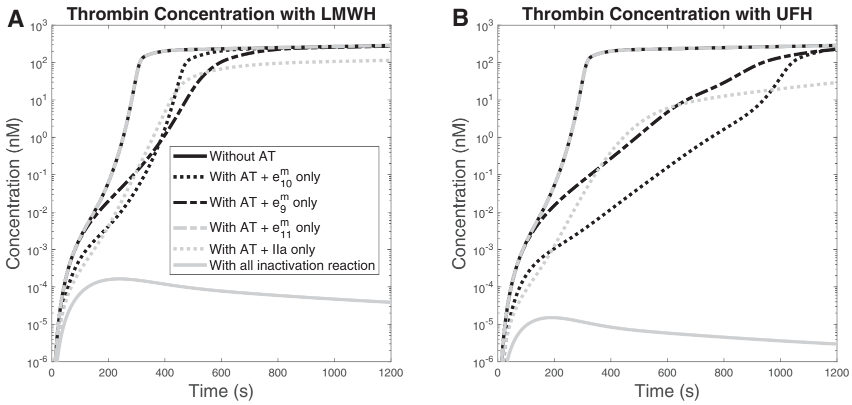

FIGURE 6 Thrombin generation in the presence of (*A*) LMWH or (*B*) UFH. The time course is obtained from simulations in which we turn off all AT-mediated inactivation reactions and then allow inhibition of FXa, FIXa, FXIa, and thrombin, individually and one by one. Each curve thus shows thrombin generation when there is either no or only one inactivation reaction that exists in the system. TF density was set to 15 fmol/cm^2, and shear rate was set to 100/s. Heparin concentration is fixed to 100% of the standard therapeutic concentration.

critical role that the platelet surface plays in this process. We find that UFH at 10% of a therapeutic level or LMWH at 100% of a therapeutic level can completely shut down thrombin generation, even at high TF densities. These results are generally in line with observed responses to heparin therapies in the sense that UFH is a stronger inhibitor than LMWH; this is because UFH affects the inactivation of FIXa, FXa, FXIa, and thrombin, whereas LMWH affects mainly the inactivation of FIXa and FXa and has less influence on the inactivation of thrombin and FXIa.

In our model we do not include direct effects of heparin on the rate of platelet deposition in the reactions. The only way that platelet deposition can be affected by heparin in this model is indirectly through its effect on thrombin generation and thrombin's activation of platelets. In particular, less thrombin from the heparin will reduce positive feedback and will also reduce the number of platelets activated by thrombin. Some plots of the platelet dynamics with varying types and concentrations of heparin are shown in the Fig. S6; heparin indeed reduces the platelet deposition.

It has been shown that the addition of heparin can also accelerate the inhibitory effect of TFPI, as shown in prothrombinase activity assays initiated with FXa, FV, prothrombin, and lipids, but such an effect was greatly diminished when FXa was preincubated with partially activated FV (37). These data are in line with several observations showing procoagulant effects of heparin when AT is not present in the system (37,38). Wood et al. further explored these ideas and showed that the negatively charged heparin molecule can block the interaction between TFPIα and partially activated FV (FV-h) (39). Therefore, addition of heparin can reduce the inhibitory effect of TFPIα toward prothrombinase that is made with FV-h but has no effect on prothrombinase made with fully activated FVa. Our model does not reflect the reduced binding interaction between TFPIα and FV-h by heparin treatment, but our model and results can still give some insight about what might happen under these conditions. The results in our companion TFPI study (26) showed that the coagulation response in the absence of binding between TFPIα and FV-h was enhanced. That scenario resembles the situation where heparin would block the binding of TFPIα to FV-h. We note that AT-mediated inactivation in that version of the model had no effect because it only acted on fluid-phase enzymes, thus we can consider it to be a case in which there is essentially no AT, isolating the procoagulant effects of heparin to be through TFPIα/FV-h interactions. Nevertheless, further exploration of procoagulant effects of heparin and the extension of heparin-TFPIα would be an interesting topic for future work.

The model allowed us to do simulations in which we could isolate the effect of inhibiting one coagulation enzyme at a time by setting the rate constants for other enzymes to zero. In doing this systematically, we found that no one single enzyme inactivation was enough to prevent substantial thrombin generation under flow for the TF densities examined. Inactivating FXIa alone had almost no effect. Inactivation of each FIXa, FXa, or thrombin individually by either LMWH or UFH led to increased lag time. The lag times were increased more for UFH than LMWH. Substantial thrombin was still produced by 20 min in these cases. The strength of heparin inhibition seems to be the simultaneous enzyme targets at multiple steps in the coagulation system.

There are other types of LMWH heparins and derivatives of UFH that have been developed for use as anticoagulant drugs (11,12,15) that we did not study in this work. There is a large body of clinical research to understand which heparins and their derivatives work best for various indications, for example treatment of deep vein thrombosis versus thromboprophylaxis after surgery. Complete details are beyond the scope of this work, but we point interested readers to a few published reviews (40,41). Researchers are still trying to identify individual risk factors associated with bleeding when using heparin as an anticoagulant treatment (42,43). Even though newer types of anticoagulants are being developed, advanced, and frequently used in place of heparins (44), both LMWH and UFH were effective in managing clotting complications of COVID-19 (45,46).

CONCLUSION

In this study, we explored the effects of surface-dependent inactivation by AT in a model coagulation system under flow. As we concluded in our companion (26) TFPI study, targeting the enzymes bound to activated platelet surfaces was required for efficient inhibition of thrombin generation. We showed that AT alone can delay thrombin generation when TF density is low but not when TF density is high. This inhibition was completely dependent on the platelet surface reactions. Our results show that AT in the presence of heparin can drastically inhibit thrombin generation. We developed a new version of our mathematical model that is sensitive to AT and heparin in ways that have been observed clinically, i.e., the magnitude of the effects of LMWH versus UFH. We found that inactivating single enzymes only was ineffective at suppressing thrombin generation. Combinations of two or three targets could be examined to aid in the design of new anticoagulant drugs. The model we developed in this study and in our companion TFPI study (26) will serve as a powerful tool for such use in future studies.

SUPPORTING MATERIAL

Supporting material can be found online at https://doi.org/10.1016/j.bpj.2022.10.038.

AUTHOR CONTRIBUTIONS

K.M. carried out all simulations. K.M., A.L.F., and K.L. designed the research, analyzed the data, and wrote the article.

ACKNOWLEDGMENTS

We would like to thank Dr. Dougald Monroe for useful discussions. This work was, in part, supported by the National Institutes of Health (R01 HL120728 and R01 HL151984) and the National Science Foundation CAREER (DMS-1848221).

DECLARATION OF INTERESTS

K.L. received research support from Novo Nordisk.

REFERENCES

1. Irving, J. A. 2000. Phylogeny of the serpin superfamily: implications of patterns of amino acid conservation for structure and function. *Genome Res.* 10:1845–1864. http://www.genome.org/cgi/doi/10.1101/gr.GR-1478R.
2. Bick, R. L., M. L. Dukes, …, L. F. Fekete. 1977. Antithrombin III (AT-III) as a diagnostic aid in disseminated intravascular coagulation. *Thromb. Res.* 10:721–729. https://linkinghub.elsevier.com/retrieve/pii/0049384877900548.
3. Rezaie, A. R., and H. Giri. 2020. Anticoagulant and signaling functions of antithrombin. *J. Thromb. Haemost.* 18:3142–3153. https://onlinelibrary.wiley.com/doi/10.1111/jth.15052.
4. Quinsey, N. S., A. L. Greedy, …, R. N. Pike. 2004. Antithrombin: in control of coagulation. *Int. J. Biochem. Cell Biol.* 36:386–389. https://linkinghub.elsevier.com/retrieve/pii/S1357272503002449.
5. Rosenberg, R. D., and P. S. Damus. 1973. The purification and mechanism of action of human antithrombin-heparin cofactor. *J. Biol. Chem.* 248:6490–6505. https://linkinghub.elsevier.com/retrieve/pii/S0021925819434728.
6. Westmark, P. R., P. Tanratana, and J. P. Sheehan. 2015. Selective disruption of heparin and antithrombin-mediated regulation of human factor IX. *J. Thromb. Haemost.* 13:1053–1063. https://onlinelibrary.wiley.com/doi/10.1111/jth.12960.
7. Yang, L., M.-f. Sun, …, A. R. Rezaie. 2009. Characterization of a heparin-binding site on the catalytic domain of factor XIa: mechanism of heparin acceleration of factor XIa inhibition by the serpins antithrombin and C1-inhibitor. *Biochemistry.* 48:1517–1524.
8. Mirta Hepner, V. K. 2013. Antithrombin. *Methods Mol. Biol.* 992:355–364.
9. Croles, F., J. Borjas-Howard, …, K. Meijer. 2018. Risk of venous thrombosis in antithrombin deficiency: a systematic review and Bayesian meta-analysis. *Semin. Thromb. Hemost.* 44:315–326. http://www.thieme-connect.de/DOI/DOI?10.1055/s-0038-1625983.
10. Olson, S. T., I. Björk, and J. D. Shore. 1993. [30] Kinetic characterization of heparin-catalyzed and uncatalyzed inhibition of blood coagulation proteinases by antithrombin. Methods in Enzymology222. Elsevier, pp. 525–559. https://linkinghub.elsevier.com/retrieve/pii/007668799322033C.
11. Olson, S. T., R. Swanson, …, I. Björk. 2004. Accelerating ability of synthetic oligosaccharides on antithrombin inhibition of proteinases of the clotting and fibrinolytic systems Comparison with heparin and low-molecular-weight heparin. *Thromb. Haemost.* 92:929–939. http://www.thieme-connect.de/DOI/DOI?10.1160/TH04-06-0384.
12. Oduah, E., R. Linhardt, and S. Sharfstein. 2016. Heparin: past, present, and future. *Pharmaceuticals.* 9:38. http://www.mdpi.com/1424-8247/9/3/38.
13. Mclean, J. 1959. The discovery of heparin. *Circulation.* 19:75–78. https://www.ahajournals.org/doi/10.1161/01.CIR.19.1.75.
14. Lindahl, U., G. Bäckström, …, A. Linker. 1979. Structure of the antithrombin-binding site in heparin. *Proc. Natl. Acad. Sci. USA.* 76:3198–3202.
15. Gray, E., B. Mulloy, and T. W. Barrowcliffe. 2008. Heparin and low-molecular-weight heparin. *Thromb. Haemost.* 99:807–818.
16. Hao, C., M. Sun, …, L. Zhang, W. Wang. 2019. Low molecular weight heparins and their clinical applications. *Prog. Mol. Biol. Transl. Sci.* 163:21–39.
17. Hirsh, J. 1998. Low-molecular-weight heparin: a review of the results of recent studies of the treatment of venous thromboembolism and unstable Angina. *Circulation.* 98:1575–1582. https://www.ahajournals.org/doi/10.1161/01.CIR.98.15.1575.
18. Bergqvist, S., U. Hedner, …, E. Holmer. 1983. Anticoagulant effects of two types of low molecular weight heparin administered subcutaneously. *Thromb. Res.* 15:381–391.
19. Fareed, J., J. M. Walenga, …, D. A. Hoppensteadt. 1985. Studies on the antithrombotic effects and pharmacokinetics of heparin fractions and fragments. *Semin. Thromb. Hemost.* 11:56–74. http://www.thieme-connect.de/DOI/DOI?10.1055/s-2007-1004360.
20. Harenberg, J., B. Würzner, …, G. Schettler. 1986. Bioavailability and antagonization of the low molecular weight heparin CY 216 in man. *Thromb. Res.* 44:549–554. https://linkinghub.elsevier.com/retrieve/pii/0049384886903336.
21. Olson, S. T., I. Björk, …, J. Choay. 1992. Role of the antithrombin-binding pentasaccharide in heparin acceleration of antithrombin-proteinase reactions. Resolution of the antithrombin conformational change contribution to heparin rate enhancement. *J. Biol. Chem.* 267:12528–12538. https://linkinghub.elsevier.com/retrieve/pii/S0021925818423095.
22. Izaguirre, G., R. Swanson, …, S. T. Olson. 2021. Paramount importance of core conformational changes for heparin allosteric activation of antithrombin. *Biochemistry.* 60:1201–1213.
23. Olson, S. 2002. Heparin activates antithrombin anticoagulant function by generating new interaction sites (exosites) for blood clotting proteinases. *Trends Cardiovasc. Med.* 12:331–338. https://linkinghub.elsevier.com/retrieve/pii/S1050173802001834.
24. Li, W., D. J. D. Johnson, …, J. A. Huntington. 2004. Structure of the antithrombin–thrombin–heparin ternary complex reveals the antithrombotic mechanism of heparin. *Nat. Struct. Mol. Biol.* 11:857–862.
25. Sinha, D., K. O. Badellino, …, P. N. Walsh. 2004. Allosteric modification of factor XIa functional activity upon binding to polyanions. *Biochemistry.* 43:7593–7600.
26. Miyazawa, K., A. L. Fogelson, and K. Leiderman. Inhibition of platelet-surface-bound proteins during coagulation under flow I: TFPI. Biophys J, In This Issue.
27. Link, K. G., M. T. Stobb, …, K. Leiderman. 2018. A local and global sensitivity analysis of a mathematical model of coagulation and platelet deposition under flow. *PLoS One.* 13, e0200917. https://dx.plos.org/10.1371/journal.pone.0200917.
28. Elizondo, P., and A. L. Fogelson. 2016. A mathematical model of venous thrombosis initiation. *Biophys. J.* 111:2722–2734.
29. Kuharsky, A. L., and A. L. Fogelson. 2001. Surface-mediated control of blood coagulation: the role of binding site densities and platelet deposition. *Biophys. J.* 80:1050–1074. https://linkinghub.elsevier.com/retrieve/pii/S0006349501760857.
30. Fogelson, A. L., and N. Tania. 2005. Coagulation under flow: the influence of flow-mediated transport on the initiation and inhibition of coagulation. *Pathophysiol. Haemost. Thromb.* 34:91–108. https://www.karger.com/Article/FullText/89930.
31. Fogelson, A. L., Y. H. Hussain, and K. Leiderman. 2012. Blood clot formation under flow: the importance of factor XI depends strongly on platelet count. *Biophys. J.* 102:10–18. https://linkinghub.elsevier.com/retrieve/pii/S0006349511013142.
32. Ahmad, S. S., R. Rawala-Sheikh, and P. N. Walsh. 1989. Comparative interactions of factor IX and factor IXa with human platelets. *J. Biol. Chem.* 264:3244–3251. https://linkinghub.elsevier.com/retrieve/pii/S0021925818940585.
33. Walsh, P. N. 1994. Platelet-coagulant protein interactions. *In* Hemostasis and Thrombosis: Basic Principles and Clinical Practices, Third

edition. R. W. Colman, J. Hirsh, V. J. Marder, and E. W. Salzman, eds J.B. Lippincott Company, pp. 629–651.

34. Greengard, J. S., M. J. Heeb, …, J. H. Griffin. 1986. Binding of coagulation factor XI to washed human paltelets. *Biochemistry.* 25:3884–3890.

35. Miller, T. N., D. Sinha, …, P. N. Walsh. 2007. A catalytic domain exosity (Cys^{527}–Cys^{542}) in factor FXIa mediates binding to a site on activated platelets. *Biochemistry.* 46:14450–14460. https://pubs.acs.org/doi/10.1021/bi701310x.

36. Byun, J.-H., I.-S. Jang, …, E.-H. Koh. 2016. Establishing the heparin therapeutic range using aPTT and anti-Xa measurements for monitoring unfractionated heparin therapy. *Blood Res.* 51:171–174. https://synapse.koreamed.org/DOIx.php?id=10.5045/br.2016.51.3.171.

37. Mast, A. E. 1996. Physiological concentrations of tissue factor pathway inhibitor do not inhibit prothrombinase. *Blood.* 87:1845–1850.

38. Smith, S. A., and J. H. Morrissey. 2008. Heparin is procoagulant in the absence of antithrombin. *Thromb. Haemost.* 100:160–162. http://www.thieme-connect.de/DOI/DOI?10.1160/TH08-05-0275.

39. Wood, J. P., M. W. Bunce, …, A. E. Mast. 2013. Tissue factor pathway inhibitor-alpha inhibits prothrombinase during the initiation of blood coagulation. *Proc. Natl. Acad. Sci. USA.* 110:17838–17843. https://pnas.org/doi/full/10.1073/pnas.1310444110.

40. Weitz, D. S., and J. I. Weitz. 2010. Update on heparin: what do we need to know? *J. Thromb. Thrombolysis.* 29:199–207.

41. Levy, J. H., and J. M. Connors. 2021. Heparin resistance—clinical perspectives and management strategies. *N. Engl. J. Med.* 385:826–832.

42. Nieuwenhuis, H. K., J. Albada, …, J. J. Sixma. 1991. Identification of risk factors for bleeding during treatment of acute venous thromboembolism with heparin or low molecular weight heparin. *Blood.* 78:2337–2343.

43. Cossette, B., M.-È. Pelletier, …, P. Farand. 2010. Evaluation of bleeding risk in patients exposed to therapeutic unfractionated or low-molecular weight heparin: a cohort study in the context of a quality improvement initiative. *Ann. Pharmacother.* 44:994–1002.

44. Weitz, J. I., and J. Harenberg. 2017. New developments in anticoagulants: past, present and future. *Thromb. Haemost.* 117:1283–1288.

45. Hippensteel, J. A., W. B. LaRiviere, …, E. P. Schmidt. 2020. Heparin as a therapy for COVID-19: current evidence and future possibilities. *Am. J. Physiol. Lung Cell Mol. Physiol.* 319:L211–L217.

46. Liu, J., J. Li, …, N. S. Key. 2020. Using heparin molecules to manage COVID-2019. *Res. Pract. Thromb. Haemost.* 4:518–523.

Biophysical Journal, Volume *122*

Supplemental information

Inhibition of platelet-surface-bound proteins during coagulation under flow II: Antithrombin and heparin

Kenji Miyazawa, Aaron L. Fogelson, and Karin Leiderman

Manuscript submitted to **Biophysical** *Journal*

Article

Supplementary Information for: Inhibition of platelet-surface-bound proteins during coagulation under flow

Kenji Miyazawa, Aaron L. Fogelson, Karin Leiderman

S1 MODEL SCHEMATIC

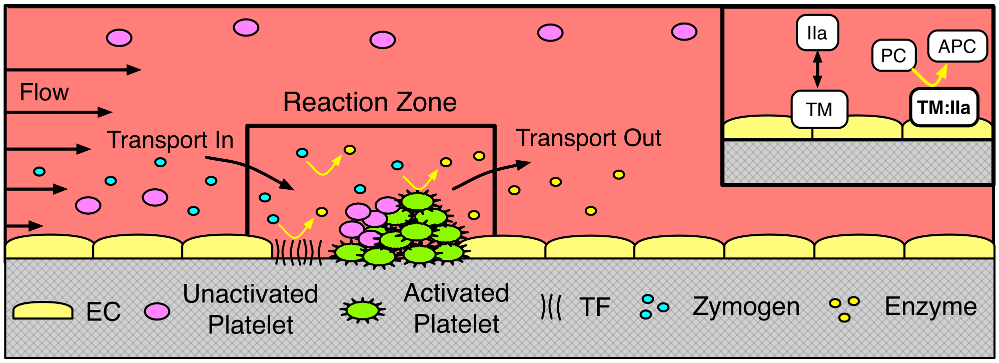

Figure S1: Schematic of the model reaction zone (main figure) and endothelial zone (inset).

S2 MODEL EQUATIONS

Below we have listed the full model equations for all species. The model detailed here includes extensions of our previous work (1–3). In total, the model consists of 130 species (and their corresponding ordinary differential equations) and 239 parameters including kinetic rates and initial/upstream concentrations. The solution of the model equations was carried out with our in-house fortran code that uses DLSODE for the numerical solution of the differential equations; each run of the model that simulates 40 minutes of clotting activity takes less than 10 seconds on a linux-based laptop. Simulations of this model (in the absence of heparin) can be performed with our online coagulation simulator: ClotSims. Below, blue text indicates the additional terms we added for the TFPI-mediated reactions, and purple text indicates the additional terms we added for the AT-mediated reactions. Strikeouts shows the terms we have removed from our previous version of the model.

$$\begin{aligned}
\frac{d}{dt}z_7 &= -k_7^{on} z_7 [TF]^{avail} + k_7^{off} z_7^m \\
&\quad -k^+_{z_7:e_2} z_7 e_2 + k^-_{z_7:e_2}[Z_7 : E_2] \\
&\quad -k^+_{z_7:e_{10}} z_7 e_{10} + k^-_{z_7:e_{10}}[Z_7 : E_{10}] \\
&\quad +k_{flow}(z_7^{up} - z_7) \\
&\quad -k^+_{z_7:e_9} z_7 e_9 + k^-_{z_7:e_9}[Z_7 : E_9]
\end{aligned} \tag{1}$$

$$\begin{aligned}
\frac{d}{dt}e_7 &= -k_7^{on} e_7 [TF]^{avail} + k_7^{off} e_7^m \\
&\quad +k^{cat}_{z_7:e_2}[Z_7 : E_2] + k^{cat}_{z_7:e_{10}}[Z_7 : E_{10}] \\
&\quad k_{flow}(e_7^{up} - e_7) + k^{cat}_{z_7:e_9}[Z_7 : E_9]
\end{aligned} \tag{2}$$

$$\begin{aligned}
\frac{d}{dt}z_{10} &= -k_{10}^{on} z_{10} p_{10}^{avail} + k_{10}^{off} z_{10}^m \\
&\quad -k^+_{z_{10}:e_7^m} e_7^m + k^-_{z_{10}:e_7^m}[Z_{10} : E_7^m] \\
&\quad k_{flow}(z_{10}^{up} - z_{10})
\end{aligned} \tag{3}$$

$$\begin{aligned}
\frac{d}{dt}e_{10} &= -k_{10}^{on} e_{10} p_{10}^{avail} + k_{10}^{off} e_{10}^m \\
&\quad +k^{cat}_{z_{10}:e_7^m}[Z_{10} : E_7^m] \\
&\quad +(k^{cat}_{z_7:e_{10}} + k^-_{z_7:e_{10}})[Z_7 : E_{10}] - k^+_{z_7:e_{10}} e_{10} z_7 \\
&\quad +(k^{cat}_{z_7^m:e_{10}} + k^-_{z_7^m:e_{10}})[Z_7^m : E_{10}] - k^+_{z_7^m:e_{10}} e_{10} z_7^m \\
&\quad -k^+_{TFPI:e_{10}} e_{10}[TFPI] + k^-_{TFPI:e_{10}^m}[TFPI : E_{10}] \\
&\quad k_{flow}(e_{10}^{up} - e_{10}) \\
&\quad \cancel{-k^{in}_{AT:e_{10}} e_{10}} \\
&\quad -k_{diff}(e_{10} - e_{10}^{ec}) \\
&\quad -k^+_{TFPI:e_5^h:e_{10}}[TFPI : E_5^h]e_{10} \\
&\quad -k^-_{TFPI:e_5^h:e_{10}}[E_{10} : TFPI : E_5^h] \\
&\quad -k^+_{TFPI:e_5^{hm}:e_{10}}[TFPI : E_5^{hm}]e_{10} \\
&\quad +k^-_{TFPI:e_5^{hm}:e_{10}}[E_{10} : TFPI : E_5^{hm}] \\
&\quad -k^{AT}_{e_{10}} e_{10}[AT] \\
&\quad +k^{ATH}_{e_{10}} e_{10}[AT : Hep]
\end{aligned} \tag{4}$$

$$
\begin{aligned}
\frac{d}{dt} z_{10}^m &= k_{10}^{on} z_{10} p_{10}^{avail} - k_{10}^{off} z_{10}^m & (5) \\
& k_{z_{10}^m:TEN}^{+} z_{10}^m [TEN] + k_{z_{10}^m:TEN}^{-} [Z_{10}^m : TEN] \\
& -k_{z_{10}^m:TEN}^{+} z_{10}^m [TEN^*] + k_{z_{10}^m:TEN}^{-} [Z_{10}^m : TEN^*]
\end{aligned}
$$

$$
\begin{aligned}
\frac{d}{dt} e_{10}^m &= k_{10}^{on} e_{10} p_{10}^{avail} - k_{10}^{off} e_{10}^m & (6) \\
& +k_{z_{10}^m:TEN}^{cat} [Z_{10}^m : TEN] + (k_{z_5^m:e_{10}^m}^{cat} + k_{z_5^m:e_{10}^m}^{-}) [Z_5^m : E_{10}^m] \\
& -k_{z_5^m:e_{10}^m}^{+} e_{10}^m z_5^m + (k_{z_8^m e_{10}^m}^{cat} + k_{z_8^m e_{10}^m}^{-}) [Z_8^m : E_{10^m}] \\
& -k_{z_8^m:e_{10}^m}^{+} e_{10}^m z_8^m \\
& +k_{e_5^m:e_{10}^m}^{-} [PRO] - k_{e_5^m:e_{10}^m}^{p} e_{10}^m e_5^m \\
& +k_{z_{10}^m:TEN}^{cat} [Z_{10}^m : TEN^*] \\
& -k_{e_5^{hm}:e_{10}^m}^{+} e_{10}^m e_5^{hm} + k_{e_5^{hm}:e_{10}^m}^{-} PRO^h \\
& -k_{TFPI:e_{10}^m}^{+} e_{10}^m TFPI + k_{TFPI:e_{10}^m}^{-} [TFPI : E_{10}^m] \\
& -k_{TFPI:e_5^{hm}:e_{10}^m}^{+} [TFPI : E_5^{hm}] e_{10}^m \\
& +k_{TFPI:e_5^{hm}:e_{10}^m}^{-} [E_{10}^m : TFPI : E_5^{hm}] \\
& -k_{TFPI:e_{10}:e_5^{hm}}^{+} [TFPI : E_5^{hm}] e_{10}^m \\
& +k_{TFPI:e_{10}:e_5^{hm}}^{-} [TFPI : PRO^h]^{v5} \\
& -k_{e_{10}^m}^{AT} e_{10}^m [AT] \\
& -k_{e_{10}^m}^{ATH} e_{10} [AT : Hep]
\end{aligned}
$$

$$
\begin{aligned}
\frac{d}{dt} z_5 &= -k_5^{on} z_5 p_5^{avail} + k_5^{off} z_5^m & (7) \\
& -k_{z_5:e_2}^{+} z_5 e_2 + k_{z_5:e_2}^{-} [Z_5 : E_2] \\
& k_{flow} (z_5^{up} - z_5) \\
& +n_5 (k_{adh}^{+} p_{PLAS}^{avail} + k_{plt}^{act} ([PL_a^v] + [PL_a^s]) + k_{e2}^{act} \frac{e_2}{e_2 + 0.001}) [PL]
\end{aligned}
$$

$$
\begin{aligned}
\frac{d}{dt} e_5 &= -k_5^{on} e_5 p_5^{avail} + k_5^{off} e_5^m & (8) \\
& +k_{z_5:e_2}^{cat} [Z_5 : E_2] \\
& +k_{flow} (e_5^{up} - e_5) \\
& k_{e_5:APC}^{-} [APC : E_5] - k_{e_5:APC}^{+} e_5 [APC] \\
& +k_{e_5^h:e_2}^{cat} [E_5^h : E_2]
\end{aligned}
$$

$$
\begin{aligned}
\frac{d}{dt} z_5^m &= k_5^{on} z_5 p_5^{avail} - k_5^{off} z_5^m & (9) \\
& -k_{z_5^m e_{10}^m}^{+} z_5^m e_{10}^m + k_{z_5^m:e_{10}^m}^{-} [Z_5^m : E_{10}^m] \\
& -k_{z_5^m:e_2^m}^{+} z_5^m e_2^m + k_{z_5^m e_2^m}^{-} [Z_5^m : E_2^m]
\end{aligned}
$$

$$
\begin{aligned}
\frac{d}{dt}e_5^m &= k_5^{on} e_5 p_5^{avail} - k_5^{off} e_5^m \\
&+k^{cat}_{z_5^m:e_2^m}[Z_5^m : E_2^m] \\
&+k^-_{e_5^m:APC}[APC : E_5^m] \\
&-k^+_{e_5^m:APC} e_5^m [APC] \\
&-k^+_{e_5^m:e_{10}^m} e_5^m e_{10}^m + k^-_{e_5^m:e_{10}^m}[PRO] \\
&+k^{cat}_{e_5^{hm}:e_2^m}[E_5^{hm} : E_2^m]
\end{aligned} \tag{10}
$$

$$
\begin{aligned}
\frac{d}{dt}z_8 &= -k_8^{on} z_8 p_8^{avail} + k_8^{off} z_8^m \\
&+k_{flow}(z_8^{up} - z_8) \\
&-k^+_{z_8:e_2} z_8 e_2 + k^-_{z_8:e_2}[Z_8 : E_2]
\end{aligned} \tag{11}
$$

$$
\begin{aligned}
\frac{d}{dt}e_8 &= -k_8^{on} e_8 p_8^{avail} + k_8^{off} e_8^m \\
&k_{flow}(e_8^{up} - e_8) \\
&+k^{cat}_{z_8:e_2} - 0.005 e_8 \\
&+k^-_{e_8:APC}[APC : E_8] - k^+_{e_8:APC} e_8 [APC]
\end{aligned} \tag{12}
$$

$$
\begin{aligned}
\frac{d}{dt}z_8^m &= k_8^{on} z_8 p_8^{avail} - k_8^{off} z_8^m \\
&-k^+_{z_8^m:e_{10}^m} z_8^m e_{10}^m + k^-_{z_8^m:e_{10}^m}[Z_8^m : E_{10}^m] \\
&-k^+_{z_8^m:e_2^m} z_8^m e_2^m \\
&+k^-_{z_8^m:e_2^m}[Z_8^m : E_2^m]
\end{aligned} \tag{13}
$$

$$
\begin{aligned}
\frac{d}{dt}e_8^m &= k_8^{on} e_8 p_8^{avail} - k_8^{off} e_8^m \\
&k^{cat}_{z_8^m:e_{10}^m}[Z_8^m : E_{10}^m] + k^{cat}_{z_8^m:e_2^m}[Z_8^m : E_2^m] \\
&-k^+_{e_8^m:APC} e_8^m [APC] + k^-_{e_8^m:APC}[APC : E_8^m] \\
&-k^+_{e_8^m:e_9^m} e_9^m e_8^m + k^-_{e_8^m:e_9^m}[TEN] - 0.005 e_8^m \\
&-k^+_{e_8^m:e_9^m} e_8^m e_9^m + k^-_{e_8^m:e_9^m}[TEN]
\end{aligned} \tag{14}
$$

$$
\begin{aligned}
\frac{d}{dt}z_9 &= k_{flow}(z_9^{up} - z_9) - k_9^{on} p_9^{avail} z_9 + k_9^{off} z_9^m \\
&-k^+_{z_9:e_7^m} z_9 e_7^m + k^-_{z_9:e_7^m}[Z_9 : E_7^m] \\
&-k^+_{z_9:e_{11}^h} e_{11}^h + k^-_{z_9:e_{11}^h}[Z_9 : E_{11}^h] \\
&-k^+_{z_9:e_{11}1} z_9 e_{11} + k^-_{z_9:e_{11}}[Z_9 : E_{11}]
\end{aligned} \tag{15}
$$

$$
\begin{aligned}
\frac{d}{dt}e_9 &= f_{flow}(e_9^{up} - e_9) \\
&\quad -k_9^{on} p_9^{avail} e_9 + k_9^{off} e_9^m \\
&\quad k_{z_9:e_7^m}^{cat} [Z_9 : E_7^m] \\
&\quad \cancel{-k_{AT:e_9}^{in} e_9} - k_{z_7:e_9}^{+} z_7 e_9 \\
&\quad +(k_{z_7:e_9}^{cat} + k_{z_7:e_9}^{-})[Z_7 : E_9] \\
&\quad +(k_{z_7^m:e_9}^{cat} + k_{z_7^m:e_9}^{-})[Z_7^m : E_9] - k_{z_7^m:e_9}^{+} z_7^m e_9 \\
&\quad -k_9^{on} p_9^{*,avail} e_9 + k_9^{off} e_9^{m*} \\
&\quad -k_{diff}(e_9 - e_9^{ec}) \\
&\quad +k_{z_9:e_{11}^h}^{cat} [Z_9 : E_{11}^h] + k_{z_9:e_{11}}^{cat} [Z_9 : E_{11}] \\
&\quad -k_{e_9}^{AT} e_9 [AT] \\
&\quad -k_{e_9}^{ATH} e_9 [AT : Hep]
\end{aligned} \tag{16}
$$

$$
\begin{aligned}
\frac{d}{dt}z_9^m &= k_9^{on} p_9^{avail} z_9 - k_9^{off} z_9^m \\
&\quad -k_{z_9^m:e_{11}^{h,m}}^{+} z_9^m e_{11}^{h,m} + k_{z_9^m e_{11}^{h,m}}^{-} [Z_9^m : E_{11}^{h,m}] \\
&\quad -k_{z_9^m:e_{11}^{m*}}^{+} z_9^m e_{11}^{m*} + k_{z_9^m e_{11}^{m*}}^{-} [Z_9^m : E_{11}^{m*}]
\end{aligned} \tag{17}
$$

$$
\begin{aligned}
\frac{d}{dt}e_9^m &= k_9^{on} p_9^{avail} e_9 - k_9^{off} e_9^m \\
&\quad -k_{e_8^m:e_9^m}^{+} e_8^m e_9^m + k_{e_8^m:e_9^m}^{-} [TEN] \\
&\quad +k_{z_9^m:e_{11}^{h,m}}^{cat} [Z_9^m : E_{11}^{h,m}] \\
&\quad +k_{z_9^m:e_{11}^{m*}}^{cat} [Z_9^m : E_{11}^{m*}] \\
&\quad -k_{e_9^m}^{AT} e_9^m [AT] \\
&\quad -k_{e_9^m}^{ATH} e_9^m [AT : Hep]
\end{aligned} \tag{18}
$$

$$
\frac{d}{dt}z_2 = -k_2^{on} p_2^{avail} z_2 + k_2^{off} z_2^m + k_{flow}(z_2^{up} - z_2) \tag{19}
$$

$$
\begin{aligned}
\frac{d}{dt}e_2 &= k_{flow}(e_2^{up} - e_2) \\
&\quad -k_{2*}^{on} p_2^{*,avail} e_2 + k_{2*}^{off} e_2^m + k_{z_2^m:PRO}^{cat} [Z_2^m : PRO] \\
&\quad \cancel{-k_{AT:e_2}^{in} e_2} - k_{z_5:e_2^p} z_5 e_2 + (k_{z_5:e_2}^{cat} + k_{z_5:e_2}^{-})[Z_5 : E_2] \\
&\quad -k_{z_8:e_2}^{+} z_8 e_2 + (k_{z_8:e_2}^{cat} + k_{z_8:e_2}^{-})[Z_8 : E_2] \\
&\quad -k_{z_7:e_2}^{+} z_7 e_2 + (k_{z_7^m:e_2}^{cat} + k_{z_7:e_2}^{-})[Z_7 : E_2] \\
&\quad -k_{z_7^m e_2}^{+} z_7^m e_2 + (k_{z_7^m:e_2}^{cat} + k_{z_7^m:e_2}^{-})[Z_7^m : E_2] \\
&\quad -k_{diff}(e_2 - e_2^{ec}) \\
&\quad -k_{z_{11}:e_2}^{+} z_{11} + (k_{z_{11}:e_2}^{-} + k_{z_{11}:e_2}^{cat})[Z_{11} : E_2] \\
&\quad -k_{e_{11}^h:e_2}^{+} e_{11}^h e_2 + (k_{e_{11}^h:e_2}^{-} + k_{e_{11}^h} : e_2)^{cat} [E_{11}^h : E_2] \\
&\quad +k_{z_2^m:PRO^h}^{cat} [Z_2^m : PRO^h] - k_{e_5^h:e_2}^{+} e_2 e_5^h \\
&\quad +k_{e_5^h:e_2}^{-} [E_5^h : E_2] + k_{e_5^h:e_2}^{cat} [E_5^h : E_2] \\
&\quad -k_{e_2}^{AT} e_2 [AT] \\
&\quad -k_{e_2}^{ATH} e_2 [AT : Hep]
\end{aligned} \tag{20}
$$

$$\frac{d}{dt}z_2^m = k_2^{on}p_2^{avail}z_2 - k_2^{off}z_2^m \tag{21}$$
$$-k^+_{z_2^m:PRO}z_2^m[PRO] + k^-_{z_2^m:PRO}[Z_2^m : PRO]$$
$$-k^+_{z_2^m:PRO^h}z_2^m PRO^h + k^-_{z_2^m:PRO^h}[Z_2^m : PROh]$$

$$\frac{d}{dt}e_2^m = k_{2*}^{on}p_2^{avail}e_2 - k_{2*}^{off}e_2^m \tag{22}$$
$$+(k^{cat}_{z_5^m:e_2^m} + k^-_{z_5^m:e_2^m})[Z_5^m : E_2^m]$$
$$-k^+_{z_5^m:e_2^m}z_5^m e_2^m$$
$$+(k^{cat}_{z_8^m:e_2^m} + k^-_{z_8^m:e_2^m})[Z_8^m : E_2^m] - k^+_{z_8^m:E_2^m}z_8^m e_2^m$$
$$-k^+_{z_{11}^m:e_2^m}z_{11}e_2^m$$
$$+(k^-_{z_{11}^m:e_2^m} + k^{cat}_{z_{11}^m:e_2^m})[Z_{11}^m : E_2^m]$$
$$-k^+_{e_{11}^{h,m*}e_2^m}e_{11}^{h,m*}e_2^m$$
$$+(k^-_{e_{11}^{h,m*}e_2^m} + k^{cat}_{e_{11}^{h,m*}:e_2^m})[E_{11}^{hms} : E_2^m]$$
$$-k^+_{e_5^{hm}:e_2^m}e_2^m e_5^{hm} + k^-_{e_5^{hm}:e_2^m}[E_5^{hm} : E_2^m]$$
$$+k^{cat}_{e_5^{hm}:e_2^m}[E_5^{hm} : E_2^m]$$
$$-k^+_{PRO^h:e_2^m}PRO^h e_2^m + k^-_{PRO^h:e_2^m}[PRO^h : E_2^m]$$
$$+k^{cat}_{PRO^h:e_2^m}[PRO^h : E_2^m]$$
$$-k^{AT}_{e_2^m}e_2^m[AT]$$
$$-k^{ATH}_{e_2^m}e_2^m[AT : Hep]$$

$$\frac{d}{dt}[TEN] = -k^-_{e_8^m:e_9^m}[TEN] + k_{e_8^m:e_9^m}e_8^m e_9^m \tag{23}$$
$$+(k^{cat}_{z_{10}^m:TEN} + k^-_{z_{10}^m:TEN})[Z_{10}^m : TEN] - k^+_{z_{10}^m:TEN}z_{10}^m[TEN]$$

$$\frac{d}{dt}[PRO] = -k^-_{e_5^m:e_{10}^m}[PRO] + k^+_{e_5^m:e_{10}^m}e_{10}^m e_5^m \tag{24}$$
$$+(k^{cat}_{z_2^m:PRO} + k^-_{z_2^m:PRO})[Z_2^m : PRO] - k^+_{z_2^m:PRO}z_2^m[PRO]$$
$$+k^{cat}_{PRO^h:e_2^m}[PRO^h : E_2^m]$$

$$\frac{d}{dt}[PL_a^s] = k^+_{adh}p^{avail}_{PLAS}[PL] - k^-_{adh}[PL_a^s] \tag{25}$$
$$+k^+_{adh}[PL_a^v] * p^{avail}_{PLAS}$$

$$\frac{d}{dt}[PL] = k^p_{flow}([PL]^{up} - [PL]) \tag{26}$$
$$-k^+_{adh}p^{avail}_{PLAS} + (k^{act}_{plt}([PL_a^v] + [PL_a^s]) + k^{act}_{e2}\frac{e_2}{e_2 + 0.001})[PL]$$

$$\frac{d}{dt}[PL_a^v] = k^-_{adh}[PL_a^s] - k^+_{adh}[PL_a^v]p^{avail}_{PLAS} \tag{27}$$
$$+(k^{act}_{plt}([PL_a^v] + [PL_a^s]) + k^{act}_{e2}\frac{e_2}{e_2 + 0.001})[PL]$$

$$\frac{d}{dt}z_7^m = k_7^{on}z_7[TF]^{avail} - k_7^{off}z_7^m \tag{28}$$
$$-k^+_{z_7^m:e_{10}}z_7^m e_{10} - k^+_{z_7^m:e_2}z_7^m e_2$$
$$+k^-_{z_7^m:e_{10}}[Z_7^m : E_{10}] + k^-_{z_7^m:e_2}[Z_7^m : E_2]$$
$$-k^+_{z_7^m:e_9}z_7^m e_9 + k^-_{z_7^m:e_9}[Z_7^m : E_9]$$
$$-z_7^m\frac{d}{dt}[PL_a^s]\frac{1}{p^{avail}_{PLAS}}$$

$$
\begin{aligned}
\frac{d}{dt}e_7^m = {} & k_7^{on}e_7[TF]^{avail} - k_7^{off}e_7^m \\
& k^+_{TFPI:e_{10}:e_7^m}e_7^m[TFPI:E_{10}] + k^-_{TFPI:e_{10}:e_7^m}[TFPI:E_7^m] \\
& +k^{cat}_{z_7^m:e_{10}}[Z_7^m:E_{10}] + k^{cat}_{z_7^m:e_2}[Z_7^m:E_2] \\
& +(k^{cat}_{z_{10}:e_7^m} + k^-_{z_{10}:e_7^m})[Z_{10}:E_7^m] \\
& -k^+_{z_{10}:e_7^m}e_7^m z_{10} - k^+_{z_9:e_7^m}e_7^m z_9 \\
& +(k^{cat}_{z_9:e_7^m} + k^-_{z_9:e_7^m})[Z_9:E_7^m] \\
& +k^{cat}_{z_7^m:e_9}[Z_7^m:E_9] - e_7^m\frac{d}{dt}[PL_a^s]\frac{1}{p^{avail}_{PLAS}}
\end{aligned} \tag{29}
$$

$$
\begin{aligned}
\frac{d}{dt}[TFPI] = {} & -k^+_{TFPI:e_{10}}e_{10}[TFPI] + k^-_{TFPI:e_{10}}[TFPI:E_{10}] \\
& k_{flow}([TFPI]^{up} - [TFPI]) \\
& -k^+_{TFPI:e_5^{hm}}e_5^{hm}[TFPI] + k^-_{TFPI:e_5^{hm}}[TFPI:E_5^{hm}] \\
& -k^+_{TFPI:e_5^h}e_5^h[TFPI] + k^-_{TFPI:e_5^h}[TFPI:E_5^h] \\
& -k^+_{TFPI:e_{10}^m}e_{10}^m[TFPI] + k^-_{TFPI:e_{10}^m}[TFPI:E_{10}^m] \\
& -k^+_{TFPI:PRO^h:v_{10}}PRO^h[TFPI] \\
& +k^-_{TFPI:PRO^h:v_{10}}[TFPI:PRO^h]^{v10} \\
& -k^+_{TFPI:PRO^h:v_5}PRO^h[TFPI] \\
& +k^-_{TFPI:PRO^h:v_5}[TFPI:PRO^h]^{v5}
\end{aligned} \tag{30}
$$

$$
\begin{aligned}
\frac{d}{dt}[TFPI:E_{10}] = {} & k^+_{TFPI:e_{10}}e_{10}[TFPI] - k^-_{TFPI:e_{10}}[TFPI:E_{10}] \\
& +k^-_{TFPI:e_{10}:e_7^m}[TFPI:E_{10}:E_7^m] \\
& -k^+_{TFPI:e_{10}:e_7^m}e_7^m[TFPI:E_{10}] + k_{flow}([TFPI:E_{10}]^{up} - [TFPI:E_{10}]) \\
& -k^+_{TFPI:e_{10}:e_5^h}[TFPI:E_{10}]e_5^h \\
& +k^-_{TFPI:e_{10}:e_5^h}[E_{10}:TFPI:E_5^h] \\
& -k^{on}_{10}[TFPI:E_{10}]p^{avail}_{10} + k^{off}_{10}[TFPI:E_{10}^m]
\end{aligned} \tag{31}
$$

$$
\begin{aligned}
\frac{d}{dt}[TFPI:E_{10}:E_7^m] = {} & -k^-_{TFPI:e_{10}:e_7^m}[TFPI:E_{10}:E_7^m] + k^+_{TFPI:e_{10}:e_7^m}e_7^m[TFPI:E_{10}] \\
& -[TFPI:E_{10}:E_7^m]\frac{d}{dt}[PL_a^s]\frac{1}{p^{avail}_{PLAS}}
\end{aligned} \tag{32}
$$

$$
\begin{aligned}
\frac{d}{dt}[APC] = {} & (k^{cat}_{e_5^m:APC} + k^-_{e_5^m:APC})[APC:E_5^m] - k^{cat}_{e_5^m:APC}e_5^m \\
& +(k^{cat}_{e_8^m:APC} + k^-_{e_8^m:APC})[APC:E_8^m] - k^+_{e_8^m:APC}e_8^m[APC] \\
& +k_{flow}([APC]^{up} - [APC]) - k_{diff}([APC] - [APC^{ec}]) \\
& +(k^{cat}_{e_5:APC} + k^-_{e_5:APC})[APC:E_5] - k^+_{e_5:APC}e_5[APC] \\
& +(k^{cat}_{e_8:APC} + k^-_{e_8:APC})[APC:E_8] - k^+_{e_8:APC}e_8[APC] \\
& -k^+_{e_5^{hm}:APC}e_5^{hm}APC + k^-_{e_5^{hm}:APC}[APC:E_5^{hm}] \\
& +k^{cat}_{e_5^{hm}:APC}[APC:E_5^{hm}] - k^+_{e_5^h:APC}e_5^h APC \\
& +k^-_{e_5^h:APC}[APC:E_5^h] + k^{cat}_{e_5^h:APC}[APC:E_5^h]
\end{aligned} \tag{33}
$$

$$\frac{d}{dt}[Z_7:E_2] = k_{flow}([Z_7:E_2]^{up} - [Z_7:E_2]) + k^+_{z_7:e_2}e_2z_7 - (k^{cat}_{z_7:e_2} + k^-_{z_7:e_2})[Z_7:E_2] \quad (34)$$

$$\frac{d}{dt}[Z_7:E_{10}] = k^+_{z_7:e_{10}}e_{10}z_7 - (k^{cat}_{z_7:e_{10}} + k^-_{z_7:e_{10}})[Z_7:E_{10}] + k_{flow}([Z_7:E_{10}]^{up} - [Z_7:E_{10}]) \quad (35)$$

$$\frac{d}{dt}[Z_7^m:E_{10}] = k^+_{z_7^m:e_{10}}e_{10}z_7^m - (k^{cat}_{z_7^m:e_{10}} + k^-_{z_7^m:e_{10}})[Z_7^m:E_{10}] - [Z_7^m:E_{10}]\frac{d}{dt}[PL_a^s]\frac{1}{p^{avail}_{PLAS}} \quad (36)$$

$$\frac{d}{dt}[Z_7^m:E_2] = k^+_{z_7^m:e_2}e_2z_7^m - (k^{cat}_{z_7^m:e_2} + k^-_{z_7^m:e_2})[Z_7^m:E_2] - [Z_7^m:E_2]\frac{d}{dt}[PL_a^s]\frac{1}{p^{avail}_{PLAS}} \quad (37)$$

$$\frac{d}{dt}[Z_{10}:E_7^m] = k^+_{z_{10}:e_7^m}e_7^mz_{10} - (k^{cat}_{z_{10}:e_7^m} + k^-_{z_{10}:e_7^m})[Z_{10}:E_7^m] - [Z_{10}:E_7^m]\frac{d}{dt}[PL_a^s]\frac{1}{p^{avail}_{PLAS}} \quad (38)$$

$$\frac{d}{dt}[Z_{10}^m:TEN] = k^+_{z_{10}^m:TEN}z_{10}^m[TEN] - (k^{cat}_{z_{10}^m:TEN} + k^-_{z_{10}^m:TEN})[Z_{10^m}:TEN] \quad (39)$$

$$\frac{d}{dt}[Z_5:E_2] = k^+_{z_5:e_2}e_2z_5 - (k^{cat}_{z_5:e_2} + k^-_{z_5:e_2})[Z_5:E_2] + k_{flow}([Z_5:E_2]^{up} - [Z_5:E_2]) \quad (40)$$

$$\frac{d}{dt}[Z_5^m:e_{10}^m] = k^+_{z_5^m:e_{10}^m}e_{10}^mz_5^m - (k^{cat}_{z_5^m:e_{10}} + k^-_{z_5^m:e_{10}^m})[Z_5^m:E_{10}^m] \quad (41)$$

$$\frac{d}{dt}[Z_5^m:E_2^m] = k^+_{z_5^m:e_2^m}e_2^mz_5^m - (k^{cat}_{z_5^m:e_2^m} + k^-_{z_5^m:e_2^m})[Z_5^m:E_2^m] \quad (42)$$

$$\frac{d}{dt}[Z_8^m:E_{10}^m] = k^+_{z_8^m:e_{10}^m}e_{10}^mz_8^m - (k^{cat}_{z_8^m:e_{10}^m} + k^-_{z_8^m:e_{10}^m})[Z_8^m:E_{10}^m] \quad (43)$$

$$\frac{d}{dt}[Z_8^m:E_2^m] = k^+_{z_8^m:e_2^m}e_2^mz_8^m - (k^{cat}_{z_8^m:e_2^m} + k^-_{z_8^m:e_2^m})[Z_8^m:E_2^m] \quad (44)$$

$$\frac{d}{dt}[Z_8:E_2] = k^+_{z_8:e_2}e_2z_8 - (k^{cat}_{z_8:e_2} + k^-_{z_8:e_2})[Z_8:E_2] \; k_{flow}([Z_8:E_2]^{up} - [Z_8:E_2]) \quad (45)$$

$$\frac{d}{dt}[APC:E_8^m] = k^+_{e_8^m:APC}e_8^m[APC] - (k^{cat}_{e_8^m:APC} + k^-_{e_8^m:APC})[APC:E_8^m] \quad (46)$$

$$\frac{d}{dt}[Z_9:E_7^m] = k^+_{z_9:e_7^m}e_7^mz_9 - (k^{cat}_{z_9:e_7^m} + k^-_{z_9:e_7^m})[Z_9:E_7^m] - [Z_9:E_7^m]\frac{d}{dt}[PL_a^s]\frac{1}{p^{avail}_{PLAS}} \quad (47)$$

$$\frac{d}{dt}[Z_2^m:PRO] = k^+_{z_2^m:PRO}z_2^m[PRO] - (k^{cat}_{z_2^m:PRO} + k^-_{z_2^m:PRO})[Z_2^m:PRO] \quad (48)$$

$$\frac{d}{dt}[APC:E_5^m] = k^+_{e_5^m:APC}e_5^m[APC] - (k^{cat}_{e_5^m:APC} + k^-_{e_5^m:APC})[APC:E_5^m] \quad (49)$$

$$\frac{d}{dt}[TF] = -[TF]\frac{d}{dt}[PL_a^s]\frac{1}{p^{avail}_{PLAS}} \quad (50)$$

$$\frac{d}{dt}[Z_7:E_9] = k^+_{z_7:e_9}e_9z_7 - (k^{cat}_{z_7:e_9} + k^-_{z_7:e_9})[Z_7:E_9] \quad (51)$$

$$\frac{d}{dt}[Z_7^m:E_9] = k^+_{z_7^m:e_9}e_9z_7^m - (k^{cat}_{z_7^m:e_9} + k^-_{z_7^m:e_9})[Z_7^m:E_9] - [Z_7^m:E_9]\frac{d}{dt}[PL_a^s]\frac{1}{p^{avail}_{PLAS}} \quad (52)$$

$$
\begin{aligned}
\frac{d}{dt}e_9^{m*} &= k_9^{on}p_9^{*,avail}e_9 - k_9^{off}e_9^{m*} + k^-_{e_8^m:e_9^m}[TEN^*] \\
&\quad -k^+_{e_8^m:e_9^m}e_8^m e_9^{m*} \\
&\quad -k^{AT}_{e_9^m}e_9^{m*}[AT] \\
&\quad -k^{ATH}_{E_9^m}e_9^{m*}[AT:Hep]
\end{aligned} \tag{53}
$$

$$
\begin{aligned}
\frac{d}{dt}[TEN^*] &= -k^-_{e_8^m:e_9^m}[TEN^*] + k^+_{e_8^m:e_9^m}e_8^m e_9^{m*} \\
&\quad +(k^{cat}_{z_{10}^m:TEN} + k^-_{z_{10}^m:TEN})[Z_{10}^m:TEN^*] \\
&\quad k^+_{z_{10}^m:TEN}[TEN^*]z_{10}^m
\end{aligned} \tag{54}
$$

$$
\frac{d}{dt}[Z_{10}^m:TEN^*] = k^+_{z_{10}^m:TEN}[TEN^*]z_{10}^m - (k^{cat}_{z_{10}^m:TEN} + k_{z_{10}^m}:TEN)^-[Z_{10}^m:TEN^*] \tag{55}
$$

$$
\begin{aligned}
\frac{d}{dt}e_2^{ec} &= k_{diff}(e_2 - e_2^{ec}) + k_{flow}(e_2^{ec,up} - e_2^{ec}) \\
&\quad \cancel{-k^{in}_{AT:e_2}e_2^{ec}} \\
&\quad -k^{on}_{TM}e_2^{ec}[TM]^{avail} + k^{off}_{TM}[TM:E_2^{ec}] - k^{AT}_{e_2^{ec}}e_2^{ec}[AT]
\end{aligned} \tag{56}
$$

$$
\begin{aligned}
\frac{d}{dt}[APC^{ec}] &= k_{flow}([APC]^{up} - [APC^{ec}]) + k_{diff}([APC] - [APC^{ec}]) \\
&\quad +k^{cat}_{PC:TM:e_2}[TM:E_2^{ec}:APC]
\end{aligned} \tag{57}
$$

$$
\begin{aligned}
\frac{d}{dt}[TM:E_2^{ec}] &= k^+_{TM}[E_2^{ec}](1 - [TM:E_2^{ec}] - [TM:E_2^{ec}:APC]) \\
&\quad -k^-_{TM}[TM:E_2^{ec}] - k^+_{PC:TM:e_2}[TM:E_2^{ec}] \\
&\quad +(k^-_{PC:TM:e_2} + k^{cat}_{PC:TM:e_2})[TM:E_2^{ec}:APC]
\end{aligned} \tag{58}
$$

$$
\begin{aligned}
\frac{d}{dt}[TM:E_2^{ec}:APC] &= k^+_{PC:TM:e_2}[TM:E_2^{ec}] \\
&\quad -(k^-_{PC:TM:e_2} + k^{cat}_{PC:TM:e_2})[TM:E_2^{ec}:APC]
\end{aligned} \tag{59}
$$

$$
\begin{aligned}
\frac{d}{dt}e_9^{ec} &= k_{diff}(e_9 - e_9^{ec}) + k_{flow}(e_9^{up} - e_9^{ec}) \\
&\quad \cancel{-k^{in}_{AT:e_9}e_9^{ec}} - k^{AT}_{e_9^{ec}}e_9^{ec}[AT]
\end{aligned} \tag{60}
$$

$$
\begin{aligned}
\frac{d}{dt}e_{10}^{ec} &= k_{diff}(e_{10} - e_{10}^{ec}) + k_{flow}(e_{10}^{up} - e_{10}^{ec}) \\
&\quad \cancel{-k^{in}_{AT:e_{10}}e_{10}^{ec}} - k^{AT}_{e_{10}^{ec}}e_{10}^{ec}[AT]
\end{aligned} \tag{61}
$$

$$
\frac{d}{dt}[APC:E_5] = -(k^{cat}_{e_5:APC} + k^-_{e_5:APC})[APC:E_5] + k^+_{e_5:APC}e_5[APC] \tag{62}
$$

$$
\frac{d}{dt}[APC:E_8] = -(k^{cat}_{e_8:APC} + k^-_{e_8:APC})[APC:E_8] + k^+_{e_8:APC}e_8[APC] \tag{63}
$$

$$
\begin{aligned}
\frac{d}{dt}z_{11} &= k_{flow}(z_{11}^{up} - z11) - k^{on}_{z_{11}}z_{11}p_{11}^{avail} \\
&\quad k^{off}_{z_{11}}z_{11}^m - k^+_{z_{11}:e_{11}^h}z_{11}e_{11}^h \\
&\quad k^-_{z_{11}:e_{11}^h}[Z_{11}:E_{11}^h] - k^+_{z_{11}:e_{11}}z_{11}e_{11} \\
&\quad k^-_{z_{11}:e_{11}}[Z_{11}:E_{11}] - k^+_{z_{11}:e_2}z_{11}e_2 \\
&\quad k^-_{z_{11}:e_2}[Z_{11}:E_2]
\end{aligned} \tag{64}
$$

$$
\begin{aligned}
\frac{d}{dt}e_{11} &= k_{flow}(e_{11}^{up} - e_{11}) && (65)\\
&-k_{e_{11}^{on,s}}e_{11}p_{111}^{avail} + k_{e_{11}^{off,s}}e_{11}^{m*}\\
&-k_{z_9:e_{11}}^{+}z_9e_{11} + (k_{z_9:e_{11}}^{-} + k_{z_9:e_{11}}^{cat})[Z_9 : E_{11}]\\
&-k_{z_{11}:e_{11}}^{+}z_{11}e_{11} + (k_{z_{11}:e_{11}}^{-} + k_{z_{11}:e_{11}}^{cat})[Z_{11} : E_{11}]\\
&+k_{e_{11}^h:e_{11}^h}^{cat}[E_{11}^h : E_{11}^h] - k_{e_{11}^h:e_{11}}^{+}e_{11}^h e_{11}\\
&+(k_{e_{11}^h:e_{11}}^{-} + 2 * k_{e_{11}^h:e_{11}}^{cat})[E_{11}^h : E_{11}]\\
&+k_{e_{11}^h:e_2}^{cat}[E_{11}^h : E_2]\\
&-k_{e_{11}}^{AT}e_{11}[AT]\\
&-k_{e_{11}}^{ATH}e_{11}[AT : Hep]\\
\frac{d}{dt}z_{11}^m &= k_{z_{11}}^{on}z_{11}p_{11}^{avail} - k_{z_{11}}^{off}z_{11}^m && (66)\\
&-k_{z_{11}^m:e_{11}^{h,m}}^{+}z_{11}^m e_{11}^{h,m} + k_{z_{11}^m:e_{11}^{h,m}}^{-}[Z_{11}^m : E_{11^{hm}}]\\
&-k_{z_{11}^m:e_{11}^{m*}}^{+}z_{11}^m e_{11}^{m*} + k_{z_{11}^m:e_{11}^{m*}}^{-}[Z_{11}^m : E_{11}^{m*}]\\
&-k_{z_{11}^m:E_2^m}^{+}z_{11}^m e_2^m + k_{z_{11}^m:e_2^m}^{-}[Z_{11}^m : E_2^m]\\
\frac{d}{dt}e_{11}^{m*} &= k_{e_{11}}^{on*}e_{11}p_{111}^{avail} - k_{e_{11}}^{off*}e_{11}^{m*} && (67)\\
&-k_{z_9^m:e_{11}^{m*}}^{+}e_{11}^{m*}z_9^m\\
&+(k_{z_9^m:e_{11}^{m*}}^{-} + k_{z_9^m:e_{11}^{m*}}^{cat})[Z_9^m : E_{11}^{m*}]\\
&-k_{z_{11}^m:e_{11}^{m*}}^{+}z_{11}^m e_{11}^{m*}\\
&+(k_{z_{11}^m:e_{11}^{m*}}^{-} + k_{z_{11}^m:e_{11}^{m*}}^{cat})[Z_{11}^m : E_{11}^{m*}]\\
&+k_{e_{11}^{h,m*}:e_{11}^{m*}}^{+}e_{11}^{h,m*}e_{11}^{m*}\\
&+(k_{e_{11}^{h,m*}:e_{11}^{m*}}^{-} + 2 * k_{e_{11}^{h,m*}:e_{11}^{m*}}^{cat})[E_{11}^{hms} : E_{11}^{h,m}]\\
&+k_{e_{11}^{h,m*}:e_2^m}^{cat}[E_{11}^{hms} : E_2^m]\\
&+k_{e_{11}}^{AT}e_{11}^{m*}[AT]\\
&-k_{e_{11}}^{ATH}e_{11}^{m*}[AT : Hep]\\
\frac{d}{dt}[Z_{11}^m : E_2^m] &= k_{z_{11}^m:e_2^m}^{+}z_{11}^m e_2^m - (k_{z_{11}^m:e_2^m}^{-} + k_{z_{11}^m:e_2^m}^{cat})[Z_{11}^m : E_2^m] && (68)\\
\frac{d}{dt}[Z_9^m : E_{11}^{m*}] &= k_{z_9^m:e_{11}^{m*}}^{+}z_9^m e_{11}^m s && (69)\\
&-(k_{z_9^m:e_{11}^{m*}}^{-} + k_{z_9^m:e_{11}^{m*}}^{cat})[Z_9^m : E_{11}^{m*}]\\
\frac{d}{dt}[Z_{11} : E_2] &= k_{flow}([Z_{11} : E_2]^{up} - [Z_{11} : E_2]) + k_{z_{11}:e_2}^{+}z_{11}e_2 && (70)\\
&-(k_{z_{11}:e_2}^{-} + k_{z_{11}:e_2}^{cat})[Z_{11} : E_2]\\
\frac{d}{dt}[Z_9 : E_{11}] &= k_{flow}([Z_9 : E_{11}]^{up} - [Z_9 : E_{11}]) + k_{z_9:e_{11}}^{+}z_9e_{11} && (71)\\
&-(k_{z_9:E_{11}}^{-} + k_{z_9:e_{11}}^{cat})[Z_9 : E_{11}]\\
\frac{d}{dt}[Z_{11} : E_{11}] &= k_{flow}([Z_{11} : E_{11}]^{up} - [Z_{11} : E_{11}]) + k_{z_{11}:e_{11}}^{+}z_{11}e_{11} && (72)\\
&-(k_{z_{11}:e_{11}}^{-} + k_{z_{11}:e_{11}}^{cat})[Z_{11} : E_{11}]
\end{aligned}
$$

$$
\begin{aligned}
\frac{d}{dt}e_{11}^{h} = \; & k_{e_{11}^{h}}^{on*}e_{11}^{h}p_{111}^{avail}+k_{e_{11}^{h}}^{off*}e_{11}^{h,m*} \\
& -k_{e_{11}^{h}}^{on}e_{11}^{h}p_{11}^{avail}+k_{e_{11}^{h}}^{off}e_{11}^{h,m} \\
& -k_{z_9:e_{11}^{h}}^{+}z_9e_{11}^{h}+(k_{z_9:e_{11}^{h,m}}^{-}+k_{z_9:e_{11}^{h}}^{cat})[Z_9:E_{11}^{h}] \\
& -k_{z_{11}:e_{11}^{hp}}z_{11}e_{11}^{h}+(k_{z_{11}:e_{11}^{h}m}^{-}+2*k_{z_{11}:e_{11}^{h}}^{cat})[Z_{11:E_{11}^{h}}] \\
& +k_{z_{11}:e_{11}}^{cat}[Z_{11}:E_{11}]+k_{z_{11}:e_2}^{cat}[Z_{11}:E_2] \\
& -2*k_{e_{11}^{h}:e_{11}^{h}}^{+}e_{11}^{h}e_{11}^{h} \\
& +(2*k_{e_{11}^{h}:e_{11}^{h}}^{-}+k_{e_{11}^{g}:e_{11}^{h}}^{cat})[E_{11}^{h}:E_{11}^{h}] \\
& -k_{e_{11}^{h}:e_{11}}^{+}e_{11}^{h}e_{11}+k_{e_{11}^{h}:e_{11}}^{-}[E_{11}^{h}:E_{11}] \\
& -k_{e_{11}^{h}:e_2}^{+}e_{11}^{h}e_2+k_{e_{11}^{h}:e_2}^{-}[E_{11}^{h}:E_2] \\
& +k_{flow}(e_{11}^{h,up}-e_{11}^{h}) \\
& -k_{e_{11}^{h}}^{AT}e_{11}^{h}[AT] \\
& -k_{e_{11}}^{ATH}e_{11}^{h}[AT:Hep]
\end{aligned}
\tag{73}
$$

$$
\begin{aligned}
\frac{d}{dt}e_{11}^{h,m} = \; & k_{e_{11}^{h}}^{on}e_{11}^{h}p_{11}^{avail}-k_{e_{11}^{h}}^{off}e_{11}^{h,m} \\
& -k_{z_9^m:e_{11}^{h,m}}^{+}z_9^{m}e_{11}^{h,m} \\
& +(k_{z_9^m:e_{11}^{h,m}}^{-}+k_{z_9^m:e_{11}^{h,m}}^{cat})[Z_9^m:E_{11}^{h,m}] \\
& -k_{z_{11}^m:e_{11}^{h,m}}^{+}z_{11}^{m}e_{11}^{h,m} \\
& +(k_{z_{11}^m:e_{11}^{h,m}}^{-}+2*k_{z_{11}^m:e_{11}^{h,m}}^{cat})[Z_{11}^m:E_{11}^{h,m}] \\
& +k_{z_{11}^m:e_{11}^{m*}}^{cat}[Z_{11}^m:E_{11}^{m*}]+k_{z_{11}^m:e_2^m}^{cat}[Z_{11}^m:E_2^m] \\
& -k_{e_{11}^{h,m*}:e_{11}^{h,m}}^{+}e_{11}^{h,m*}e_{11}^{h,m} \\
& +(k_{e_{11}^{h,m*}:e_{11}^{h,m}}^{-}+k_{e_{11}^{h,m*}:e_{11}^{h,m}}^{cat})[E_{11}^{hms}:E_{11}^{h,m}] \\
& -k_{e_{11}}^{AT}e_{11}^{hm}[AT] \\
& -k_{e_{11}}^{ATH}e_{11}^{hm}[AT:Hep]
\end{aligned}
\tag{74}
$$

$$
\begin{aligned}
\frac{d}{dt}e_{11}^{h,m*} = \; & k_{e_{11}^{h}}^{on*}e_{11}^{h}p_{111}^{avail}-k_{e_{11}^{h}}^{off*}e_{11}^{h,m*} \\
& -k_{e_{11}^{h,m*}:e_{11}^{h,m}}^{+}e_{11}^{h,m*}e_{11}^{h,m}+k_{e_{11}^{h,m*}:e_{11}^{h,m}}^{-}[E_{11}^{hms}:E_{11}^{h,m}] \\
& -k_{e_{11}^{h,m*}:e_{11}^{m*}}^{+}e_{11}^{h,m*}e_{11}^{m*}+k_{e_{11}^{h,m*}:e_{11}^{m}s}^{-}[E_{11}^{hms}:E_{11}^{m*}] \\
& -k_{e_{11}^{h,m*}:e_2^m}^{+}e_{11}^{h,m*}e_2^{m}+k_{e_{11}^{h,m*}:e_2^m}^{-}[E_{11}^{hms}:E_2^{m}]
\end{aligned}
\tag{75}
$$

$$
\begin{aligned}
\frac{d}{dt}[Z_9:E_{11}^{h}] = \; & k_{flow}([Z_9:E_{11}^{h}]^{up}-[Z_9:E_{11}^{h}]) \\
& +k_{z_9:e_{11}^{h}}^{+}z_9e_{11}^{h}-(k_{z_9:e_{11}^{h}}^{-}+k_{z_9:e_{11}^{h}}^{cat})[Z_9:E_{11}^{h}]
\end{aligned}
\tag{76}
$$

$$
\begin{aligned}
\frac{d}{dt}[Z_9^m:E_{11}^{h,m}] = \; & k_{z_9^m:e_{11}^{h,m}}^{+}z_9^{m}e_{11}^{h,m} \\
& -(k_{z_9^m:e_{11}^{h}m}^{-}+k_{z_9^m:e_{11}^{h}m}^{cat})[Z_9^m:E_{11}^{h,m}]
\end{aligned}
\tag{77}
$$

$$\begin{aligned} \frac{d}{dt}[Z_{11}:E_{11}^h] &= k_{flow}([Z_{11}:E_{11}^h]^{up} - [Z_{11}:E_{11}^h]) \\ &+k_{z_{11}:e_{11}^{hp}} z_{11} e_{11}^h \\ &-(k^-_{z_{11}:e_{11}^h} + k^{cat}_{z_{11}:e_{11}^h})[Z_{11}:E_{11}^h] \end{aligned} \tag{78}$$

$$\begin{aligned} \frac{d}{dt}[E_{11}^h:E_{11}^h] &= k_{flow}([E_{11}^h:E_{11}^h]^{up} - [E_{11}^h:E_{11}^h]) \\ &+k^+_{e_{11}^h:e_{11}^h} e_{11}^h e_{11}^h \\ &-(k^-_{e_{11}^h:e_{11}^h} + k^{cat}_{e_{11}^h:e_{11}^h})[E_{11}^h:E_{11}^h] \end{aligned} \tag{79}$$

$$\begin{aligned} \frac{d}{dt}[E_{11}^h:E_{11}] &= k_{flow}([E_{11}^h:E_{11}]^{up} - [E_{11}^h:E_{11}]) \\ &+k^+_{e_{11}^h:e_{11}} e_{11}^h - (k^-_{e_{11}^h:e_{11}} + k^{cat}_{e_{11}^h:e_{11}})[E_{11}^h:E_{11}] \end{aligned} \tag{80}$$

$$\begin{aligned} \frac{d}{dt}[E_{11}^h:E_2] &= k_{flow}([E_{11}^h:E_2]^{up} - [E_{11}^h:E_2]) \\ &+k^+_{e_{11}^h:e_2} e_{11}^h e_2 - (k^-_{e_{11}^h:e_2} + k^{cat}_{e_{11}^h:e_2})[E_{11}^h:E_2] \end{aligned} \tag{81}$$

$$\begin{aligned} \frac{d}{dt}[Z_{11}^m:E_{11}^{h,m}] &= k^+_{z_{11}^m:e_{11}^{h,m}} z_{11}^m e_{11}^{h,m} \\ &-(k^-_{z_{11}^m:e_{11}^{h,m}} + k^{cat}_{z_{11}^m:e_{11}^{h,m}})[Z_{11}^m:E_{11}^{h,m}] \end{aligned} \tag{82}$$

$$\begin{aligned} \frac{d}{dt}[Z_{11}^m:E_{11}^{m*}] &= k^+_{z_{11}^m:e_{11}^{m*}} z_{11}^m e_{11}^{m*} \\ &-(k^-_{z_{11}^m:e_{11}^{m*}} + k^{cat}_{z_{11}^m:e_{11}^m s})[Z_{11}^m:E_{11}^{m*}] \end{aligned} \tag{83}$$

$$\begin{aligned} \frac{d}{dt}[E_{11}^{hms}:E_{11}^{h,m}] &= k^+_{e_{11}^{h,m*}:e_{11}^{h,m}} e_{11}^{h,m} \\ &-(k^-_{e_{11}^{h,m*}:e_{11}^{h,m}} + k^{cat}_{e_{11}^{h,m*}:e_{11}^{h,m}})[E_{11}^{hms}:E_{11}^{h,m}] \end{aligned} \tag{84}$$

$$\begin{aligned} \frac{d}{dt}[E_{11}^{hms}:E_{11}^{m*}] &= k_{e_{11}^{h,m*}:e_{11}^{h,m*}} e_{11}^{m*} \\ &-(k^-_{e_{11}^{h,m*}:e_{11}^{m*}} + k^{cat}_{e_{11}^{h,m*}:e_{11}^{m*}})[E_{11}^{hms}:E_{11}^{m*}] \end{aligned} \tag{85}$$

$$\begin{aligned} \frac{d}{dt}[E_{11}^{hms}:E_2^m] &= k^+_{e_{11}^{h,m*}:e_2^m} e_{11}^{h,m*} e_2^m \\ &-(k^-_{e_{11}^{hms}:e_2^m} + k^{cat}_{e_{11}^{h,m*}:e_2^m}[E_{11}^{hms}:E_2^m]) \end{aligned} \tag{86}$$

$$\begin{aligned} \frac{d}{dt}e_5^{hm} &= +k^{cat}_{z_5^m:e_{10}^m}[Z_5^m:E_{10}^m] + k_5^{on} e_5^h p_5^{avail} \\ &-k_5^{off} e_5^{hm} - k^+_{e_5^{hm}:e_{10}^m} e_{10}^m e_5^{hm} + k^-_{e_5^{hm}:e_{10}^m} PRO^h \\ &-k^+_{e_5^{hm}:e_2^m} e_2^m e_5^{hm} + k^-_{e_5^{hm}:e_2^m}[E_5^{hm}:E_2^m] \\ &-k^+_{TFPI:e_5^{hm}} e_5^{hm} TFPI + k^-_{TFPI:e_5^{hm}}[TFPI:E_5^{hm}] \\ &-k^+_{e_5^{hm}:APC} e_5^{hm} APC + k^-_{e_5^{hm}:APC}[APC:E_5^{hm}] \\ &-k^+_{TFPI:e_{10}:e_5^{hm}}[TFPI:E_{10}^m] e_h^{hm} \\ &+k^-_{TFPI:e_{10}:e_5^{hm}}[E_{10}^m:TFPI:E_5^{hm}] \\ &-k^+_{TFPI:e_{10}:e_5^{hm}:e_{10}^m}[TFPI:E_{10}^m] e_5^{hm} \\ &+k^-_{TFPI:e_{10}:e_5^{hm}:e_{10}^m}[TFPI:PRO^h_{v10}] \end{aligned} \tag{87}$$

$$
\begin{aligned}
\frac{d}{dt}e_5^h &= -k_{5on}e_5^h p_5^{avail} + k_5^{off} e_5^{hm} \\
&+k_{flow}(e_5^{up} - e_5^h) + (1 - f_5)N_5 dpl \cdot p \\
&-k^+_{e_5^h:e_2} e_5^h + k^-_{e_5^h:e_2}[E_5^h : E_2] \\
&-k^+_{e_5^h:APC} APC \cdot e_5^h + k^-_{e_5^h:APC}[APC : E_5^h] \\
&-k^+_{TFPI:e_5^h} e_5^h TFPI \\
&+k^-_{TFPI:e_5^h}[TFPI : E_5^h] \\
&-k^+_{TFPI:e_{10}:e_5^h}[TFPI : E_{10}]e_5^h \\
&+k^-_{TFPI:e_{10}:e_5^h}[E_{10} : TFPI : E_5^h] \\
&-k^+_{TFPI:e_{10}^m:e_5^h}[TFPI : E_{10}^m]e_5^h \\
&+k^-_{TFPI:e_{10}^m:e_5^h}[E_{10}^m : TFPI : E_5^h]
\end{aligned} \tag{88}
$$

$$
\begin{aligned}
\frac{d}{dt}PRO^h &= +k^+_{e_5^{hm}:e_{10}^m} e_{10}^m e_5^{hm} - k^-_{e_5^{hm}:e_{10}^m} PRO^h \\
&-k^+_{z_2^m:PRO^h} PRO^h z_2^m + k^-_{z_2^m:PRO^h}[Z_2^m : PRO^h] \\
&+k^{cat}_{z_2^m:PRO^h}[Z_2^m : PRO^h] \\
&-k^+_{TFPI:PRO^h:v_{10}} PRO^h TFPI + k^-_{TFPI:PRO^h:v_{10}}[TFPI : PRO^h_{v10}] \\
&-k^+_{TFPI:PRO^h:v_5} PRO^h TFPI + k^-_{TFPI:PRO^h:v_5}[TFPI : PRO^h_{v5}] \\
&-k^+_{PRO^h:e_2^m} PRO^h e_2^m + k^-_{PRO^h:e_2^m}[PRO^h : E_2^m]
\end{aligned} \tag{89}
$$

$$
\begin{aligned}
\frac{d}{dt}[Z_2^m : PRO^h] &= +k^+_{z_2^m:PRO^h} PRO^h z_2^m - k^-_{z_2^m:PRO^h}[Z_2^m : PRO^h] \\
&-k^{cat}_{z_2^m:PRO^h}[Z_2^m PRO^h]
\end{aligned} \tag{90}
$$

$$
\begin{aligned}
\frac{d}{dt}[E_5^{hm} : E_2^m] &= +k^+_{e_5^{hm}:e_2^m} e_2^m e_5^{hm} - k^-_{e_5^{hm}:e_2^m}[E_5^{hm} : E_2^m] \\
&-k^{cat}_{e_5^{hm}:e_2^m}[E_5^{hm} : E_2^m]
\end{aligned} \tag{91}
$$

$$
\begin{aligned}
\frac{d}{dt}[E_5^h : E_2] &= +k^+_{e_5^h:e_2} e_2 e_5^h - k^-_{TFPI:e_5^h}[E_5^h : E_2] \\
&-k^{cat}_{e_5^h:e_2}[E_5^h : E_2] + k_{flow}([E_5^h : E_2]^{up} - [E_5^h : E_2])
\end{aligned} \tag{92}
$$

$$
\begin{aligned}
\frac{d}{dt}[TFPI : E_5^{hm}] &= +k^+_{TFPI:e_5^{hm}} e_5^{hm} TFPI - k^-_{TFPI:e_5^{hm}}[TFPI : E_5^{hm}] \\
&-k^+_{TFPI:e_5^{hm}:e_{10}m}[TFPI : E_5^{hm}]e_{10}^m \\
&+k^-_{TFPI:e_5^{hm}:e_{10}m}[E_{10}^m : TFPI : E_5^{hm}] \\
&+k_5^{on}[TFPI : E_5^h]p_5^{avail} - k_5^{off}[TFPI : E_5^{hm}] \\
&-k^+_{TFPI:e_{10}:e_5^{hm}}[TFPI : E_5^{hm}]e_{10}^m \\
&+k^-_{TFPI:e_{10}:e_5^{hm}}[TFPI : PRO^h_{v5}] \\
&-k^+_{TFPI:e_5^{hm}:e_{10}}[TFPI : E_5^{hm}]e_{10} \\
&+k^-_{TFPI:e_5^{hm}:e_{10}}[E_{10} : TFPI : E_5^{hm}]
\end{aligned} \tag{93}
$$

$$
\begin{aligned}
\frac{d}{dt}[APC : E_5^{hm}] &= +k^+_{e_5^{hm}:APC} E_5^{hm} APC - k^-_{e_5^{hm}:APC}[APC : E_5^{hm}] \\
&+k^{cat}_{e_5^{hm}:APC}[APC : E_5 hm]
\end{aligned} \tag{94}
$$

$$
\begin{aligned}
\frac{d}{dt}[APC:E_5^h] = {} & +k^{+}_{e_5^h:APC}e_5^h APC - k^{-}_{e_5^{hm}:APC}[APC:E_5^h] \\
& -k^{cat}_{e_5^h:APC}[APC:E_5^h] \\
& +k_{flow}([APC:E_5^h]^{up} - [APC:E_5^h])
\end{aligned} \tag{95}
$$

$$
\begin{aligned}
\frac{d}{dt}[TFPI:E_5^h] = {} & +k_{TFPI:e_5^h up}e_5^h TFPI - k^{-}_{kTFPI:e_5^h}[TFPI:E_5^h] \\
& +k_{flow}([TFPI:E_5^h]^{up} - [TFPI:E_5^h]) \\
& -k^{+}_{TFPI:e_5^h:e_{10}}[TFPI:E_5^h]e_{10} \\
& +k^{-}_{TFPI:e_5^h:e_{10}}[E_{10}:TFPI:E_5^h] \\
& +k_5^{on}[TFPI:E_5^h]p_5^{avail} + k_5^{off}[TFPI:E_5^{hm}]
\end{aligned} \tag{96}
$$

$$
\begin{aligned}
\frac{d}{dt}[TFPI:E_{10}^m] = {} & +k^{+}_{TFPI:e_{10}^m}e_{10}^m TFPI - k^{-}_{TFPI:e_{10}^m}[TFPI:E_{10}^m] \\
& -k^{+}_{TFPI:e_{10}:e_5^h m}[TFPI:E_{10}^m]e_5^{hm} \\
& +k^{-}_{TFPI:e_{10}:e_5^{hm}}[E_{10}^m:TFPI:E_5^{hm}] \\
& +k_{10}^{on}[TFPI:E_{10}]p_{10}^{avail} \\
& -k_{10}^{off}[TFPI:E_{10}^m] \\
& -k^{+}_{TFPI:e_{10}^m:e_5^{hm}}[TFPI:E_{10}^m]e_5^{hm} \\
& +k^{-}_{TFPI:e_{10}^m:e_5^{hm}}[TFPI:PRO^h]^{v10} \\
& -k^{+}_{TFPI:e_{10}^m:e_5^h}[TFPI:E_{10}^m]e_5^h \\
& +k^{-}_{TFPI:e_{10}^m:e_5^h}[E_{10}^m:TFPI:E_5^h]
\end{aligned} \tag{97}
$$

$$
\begin{aligned}
\frac{d}{dt}[TFPI:PRO^h]^{v10} = {} & +k^{+}_{TFPI:PRO^h:v_{10}}PRO^h TFPI \\
& -k^{-}_{TFPI:PRO^h:v_{10}}[TFPI:PRO^h]^{v10} \\
& +k^{+}_{TFPI:e_{10}^m:e_5^{hm}}[TFPI:E_{10}^p][TFPI:E_{10}^m]e_5^{hm} \\
& -k^{-}_{TFPI:e_{10}^m:e_5^{hm}}[TFPI:PRO^h]^{v10}
\end{aligned} \tag{98}
$$

$$
\begin{aligned}
\frac{d}{dt}[TFPI:PRO^h]^{v5} = {} & +k^{+}_{TFPI:PRO^h:v_5}PRO^h TFPI \\
& -k^{-}_{TFPI:PRO^h:v_5}[TFPI:PRO^h]^{v5} \\
& +k^{+}_{TFPI:e_{10}:e_5^{hm}}[TFPI:E_5^{hm}]e_{10}^m \\
& -k^{-}_{TFPI:e_{10}:e_5^{hm}}[TFPI:PRO^h]^{v5}
\end{aligned} \tag{99}
$$

$$
\begin{aligned}
\frac{d}{dt}[E_{10}^m:TFPI:E_5^{hm}] = {} & +k^{+}_{TFPI:e_{10}:e_5^h m}[TFPI:E_{10}^m]e_5^{hm} \\
& -k^{-}_{TFPI:e_{10}:e_5^h m}[E_{10}^m:TFPI:E_5^{hm}] \\
& +k^{+}_{TFPI:e_5^{hm}:e_{10}m}[TFPI:E_5^{hm}]e_{10}^m \\
& -k^{-}_{TFPI:e_5^{hm}:e_{10}m}[E_{10}^m:TFPI:E_5^{hm}] \\
& +k_{10}^{ont}p_{10}^{avail} - k_{10}^{offt}[E_{10}^m:TFPI:E_5^{hm}] \\
& +k_5^{ont}[E_{10}^m:TFPI:E_5^h]p_5^{avail} \\
& -k_5^{offt}[E_{10}^m:TFPI:E_5^{hm}]
\end{aligned} \tag{100}
$$

$$\frac{d}{dt}[E_{10}:TFPI:E_5^h] = +k_{flow}([E_{10}:TFPI:E_5^h]^{up} - [E_{10}:TFPI:E_5^h]) \tag{101}$$
$$+k^+_{TFPI:e_{10}:e_5^h}[TFPI:E_{10}]e_5^h$$
$$-k^-_{TFPI:e_{10}:e_5^h}[E_{10}:TFPI:E_5^h]$$
$$+k^+_{TFPI:e_5^h:e_{10}}[TFPI:E_5^h]e_{10}$$
$$-k^-_{TFPI:e_5^h:e_{10}}[E_{10}:TFPI:E_5^h]$$
$$-k_5^{ont}[E_{10}:TFPI:E_5^h]p_5^{avail}$$
$$+k_5^{offt}[E_{10}:TFPI:E_5^{hm}]$$
$$-k_{10}^{ont}[E_{10}:TFPI:E_5^h]p_{10}^{avail}$$
$$+K_{10}^{offt}[E_{10}^m:TFPI:E_5^h]$$

$$\frac{d}{dt}[E_{10}:TFPI:E_5^{hm}] = +k_5^{ont}[E_{10}:TFPI:E_5^h]p_5^{avail} \tag{102}$$
$$-k_5^{offt}[E_{10}:TFPI:E_5^{hm}]$$
$$-k_{10}^{ont}[E_{10}:TFPI:E_5^[hm]]p_{10}^{avail}$$
$$+k_{10}^{offt}[E_{10}^m:TFPI:E_5^{hm}]$$
$$+k^+_{TFPI:e_5^{hm}:e_{10}}[TFPI:E_5^{hm}]e_{10}$$
$$-k^-_{TFPI:e_5^{hm}:e_{10}}[E_{10}:TFPI:E_5^{hm}]$$

$$\frac{d}{dt}[E_{10}^m:TFPI:E_5^h] = +k_{10}^{ont}[E_{10}:TFPI:E_5^h]p_{10}^{avail} \tag{103}$$
$$-k_{10}^{offt}[E_{10}^m:TFPI:E_5^h]$$
$$-k_5^{ont}[E_{10}^m:TFPI:E_5^h]p_5^{avail}$$
$$+k_5^{offt}[E_{10}^m:TFPI:E_5^{hm}]$$
$$+k^+_{TFPI:e_{10}^m:e_5^h}[TFPI:E_{10}^m]e_5^h$$
$$-k^-_{TFPI:e_{10}^m:e_5^h}[E_{10}^m:TFPI:E_5^h]$$

$$\frac{d}{dt}[PRO^h:E_2^m] = +k^+_{PRO^h:e_2^m}PRO^hE_2^m - k^-_{PRO^h:e_2^m}[PRO^h:E_2^m] \tag{104}$$
$$-k^{cat}_{PRO^h:e_2^m}[PRO^h:E_2^m]$$

$$\frac{d}{dt}[E_9:AT] = -k_9^{on}p_9^{avail}[E_9:AT] \tag{105}$$
$$+k_9^{off}[E_9^m:AT] + k_{e_9}^{AT}e_9[AT]$$
$$+k_{flow}([E_9:AT]_{up} - [E_9:AT])$$
$$-k_9^{on}p_{91}^{avail}[E_9:AT] + k_9^{off}[E_9^{m*}:AT]$$

$$\frac{d}{dt}[E_9^m:AT] = +k_{e_9^m}^{AT}e_9^m[AT] \tag{106}$$
$$-k_9^{off}[E_9^m:AT] + k_9^{on}p_9^{avail}[E_9:AT]$$

$$\frac{d}{dt}[E_9^{m*}:AT] = +k_{e_9^m}^{AT}e_9^{m*}[AT] \tag{107}$$
$$-k_9^{off}[E_9^{m*}:AT] + k_9^{on}p_{91}^{avail}[E_9:AT]$$

$$\frac{d}{dt}[E_{10}:AT] = +k_{e_{10}}^{AT}e_{10}[AT] \tag{108}$$
$$+k_{flow}([E_{10}:AT]_{up} - [E_{10}:AT])$$
$$+k_{10}^{off}[E_{10}^m:AT] - k_{10}^{on}p_{10}^{avail}[E_{10}:AT]$$

$$\frac{d}{dt}[E_{10}^m : AT] = +k_{e_{10}^m}^{AT} e_{10}^m [AT] \quad (109)$$
$$-k_{10}^{off}[E_{10}^m : AT] + k_{10}^{on} p_{10}^{avail}[E_{10} : AT]$$
$$\frac{d}{dt}[E_2 : AT] = +k_{e_2}^{off}[E_2^m : AT] - k_{e_2}^{on} p_2^{avail}[E_2 : AT] \quad (110)$$
$$+k_{e_2}^{AT} e_2 + k_{flow}([E_2 : AT]_{up} - [E_2 : AT])$$
$$\frac{d}{dt}[E_2^m : AT] = +k_{e_2^m}^{AT} e_2^m [AT] \quad (111)$$
$$-k_{e_2}^{off}[E_2^m : AT] + k_{e_2}^{on} p_2^{avail}[E_2 : AT]$$
$$\frac{d}{dt}[E_{11} : AT] = +k_{e_{11}}^{AT} e_{11}[AT] \quad (112)$$
$$-k_{e_{11}}^{AT}[E_{11} : AT][AT]$$
$$+k_{11}^{off}[E_{11}^{m*} : AT] - k_{11}^{on} p_{111}^{avail}[E_{11} : AT]$$
$$+k_{e_{11}}^{ATH} e_{11}[AT : Hep] - k_{e_{11}}^{ATH}[E_{11} : ATH][AT : Hep]$$
$$+k_{flow}([E_{11} : ATH]_{up} - [E_{11} : ATH])$$
$$-k_{e_{11}}^{on} p_{111}^{avail}[E_{11} : ATH] + k_{e_{11}}^{off}[E_{11}^{m*} : ATH]$$
$$\frac{d}{dt}[AT : E_{11} : AT] = +k_{e_{11}}^{AT}[E_{11} : AT][AT] \quad (113)$$
$$\frac{d}{dt}[E_{11}^{m*} : AT] = +k_{e_{11}}^{AT} e_{11}^{m*}[AT] \quad (114)$$
$$-k_{11}^{off}[E_{11}^{m*} : AT] + k_{11}^{on} p_{111}^{avail}[E_{11} : AT]$$
$$\frac{d}{dt}[E_{11}^h : AT] = +k_{e_{11}}^{AT} e_{11}^h [AT] \quad (115)$$
$$+k_{11}^{off}[E_{11}^{hm} : AT] - k_{11}^{on} p_{11}^{avail}[E_{11}^h : AT]$$
$$\frac{d}{dt}[E_{11}^{hm} : AT] = +k_{e_{11}}^{AT} e_{11}^{hm}[AT] \quad (116)$$
$$-k_{11}^{off}[E_{11}^{hm} : AT] + k_{11}^{on} p_{11}^{avail}[E_{11}^h : AT]$$
$$\frac{d}{dt}[AT] = -k_{e_9}^{AT} e_9[AT] - k_{e_9^m}^{AT} e_9^m[AT] \quad (117)$$
$$+k_{e_9^m}^{AT} e_9^{m*}[AT]$$
$$-k_{e_{10}}^{AT} e_{10}[AT] + ke_{10}^{m\,AT} e_{10}^m[AT]$$
$$-k_{e_2}^{AT} e_2[AT] - k_{e_2^m}^{AT} e_2^m[AT]$$
$$-k_{e_{11}}^{AT} e_{11}[AT] - k_{e_{11}}^{AT}[E_{11} : AT][AT]$$
$$-k_{e_{11}}^{AT} e_{11}^{m*}[AT] - k_{e_{11}}^{AT} e_{11}^h[AT]$$
$$-k_{e_{11}}^{AT} e_{11}^h[AT]$$
$$+k_{flow}([AT]_{up} - [AT])$$
$$-k_{[AT:Hep]}^{+}[Hep][AT] + k_{[AT:Hep]}^{-}[AT : Hep]$$
$$\frac{d}{dt}[Hep] = -k_{[AT:Hep]}^{+}[Hep][AT] + k_{[AT:Hep]}^{-}[AT : Hep] \quad (118)$$

$$\begin{aligned}\frac{d}{dt}[AT:Hep] = {} & +k^{+}_{[AT:Hep]}[Hep][AT] - k^{-}_{[AT:Hep]}[AT:Hep] \\ & -k^{ATH}_{e_{10}} e_{10}[AT:Hep] - k^{ATH}_{e^m_{10}} e^m_{10}[AT:Hep] \\ & -k^{ATH}_{e_2} e_2[AT:Hep] - k^{ATH}_{e^m_2} e^m_2[AT:Hep] \\ & -k^{ATH}_{e_9} e_9[AT:Hep] - k^{ATH}_{e^m_9} e^m_9[AT:Hep] \\ & -k^{ATH}_{e^m_9} e^{m*}_9[AT:Hep] \\ & -k^{ATH}_{e_{11}} e_{11}[AT:Hep] - k^{ATH}_{e_{11}}[E_{11}:ATH][AT:Hep] \\ & -k^{ATH}_{e_{11}} e^{m*}_{11}[AT:Hep] - k^{ATH}_{e_{11}} e^h_{11}[AT:Hep] \\ & -k^{ATH}_{e_{11}} e^{hm}_{11}[AT:Hep]\end{aligned} \tag{119}$$

$$\begin{aligned}\frac{d}{dt}[E_{10}:ATH] = {} & +k^{ATH}_{e_{10}} e_{10}[AT:Hep] + k_{flow}([E_{10}:ATH]_{up} - [E_{10}:ATH]) \\ & -k^{on}_{10} p^{avail}_{10}[E_{10}:ATH] + k^{off}_{10}[E^m_{10}:ATH]\end{aligned} \tag{120}$$

$$\begin{aligned}\frac{d}{dt}[E^m_{10}:ATH] = {} & +k^{ATH}_{e^m_{10}} e^m_{10}[AT:Hep] \\ & +k^{on}_{10} p^{avail}_{10}[E_{10}:ATH] - k^{off}_{10}[E^m_{10}:ATH]\end{aligned} \tag{121}$$

$$\begin{aligned}\frac{d}{dt}[E_2:ATH] = {} & +k^{ATH}_{e_2} e_2[AT:Hep] + k_{flow}([E_2:ATH]_{up} - [E_2:ATH]) \\ & -k^{on}_{e_2} p^{avail}_2[E_2:ATH] + k^{off}_{e_2}[E^m_2:ATH]\end{aligned} \tag{122}$$

$$\begin{aligned}\frac{d}{dt}[E^m_2:ATH] = {} & +k^{ATH}_{e^m_2} e^m_2[AT:Hep] \\ & +k^{on}_2 p^{avail}_2[E_2:ATH] - k^{off}_{e_2}[E^m_2:ATH]\end{aligned} \tag{123}$$

$$\begin{aligned}\frac{d}{dt}[E_9:ATH] = {} & +k^{ATH}_{e_9} e_9[AT:Hep] + k_{flow}([E_9:ATH]_{up} - [E_9:ATH]) \\ & -k^{on}_{e_9} p^{avail}_9[E_9:ATH] + k^{off}_{e_9}[E^m_9:ATH] \\ & -k^{on}_{e_9} p^{avail}_{91}[E_9:ATH] + k^{off}_9[E^{m*}_9:ATH]\end{aligned} \tag{124}$$

$$\begin{aligned}\frac{d}{dt}[E^{m*}_9:ATH] = {} & +k^{ATH}_{e^m_9} e^m_9[AT:Hep] \\ & +k^{on}_{e_9} p^{avail}_9[E_9:ATH] - k^{off}_{e_9}[E^m_9:ATH]\end{aligned} \tag{125}$$

$$\begin{aligned}\frac{d}{dt}[E^{m*}_9:ATH] = {} & +k_{e^m_9:ATH} e^{m*}_9[AT:Hep] \\ & +k^{on}_{e_9} p^{avail}_{91}[E_9:ATH] - k^{off}_{e_9}[E^{m*}_9:ATH]\end{aligned} \tag{126}$$

$$\begin{aligned}\frac{d}{dt}[ATH:E_{11}:ATH] = {} & +k^{ATH}_{e_{11}}[AT:Hep][E_{11}:ATH] \\ & +k_{flow}([ATH:E_{11}:ATH]_{up} - [ATH:E_{11}:ATH])\end{aligned} \tag{127}$$

$$\begin{aligned}\frac{d}{dt}[E^{m*}_{11}:ATH] = {} & +k^{ATH}_{e_{11}} e^{m*}_{11}[AT:Hep]+k^{on}_{e_{11}} p^{avail}_{111}[E_{11}:ATH] \\ & -k^{off}_{e_{11}}[E^{m*}_{11}:ATH]\end{aligned} \tag{128}$$

$$\begin{aligned}\frac{d}{dt}[E^h_{11}:ATH] = {} & +k^{ATH}_{e_{11}} e^h_{11}[AT:Hep] \\ & +k_{flow}([E^h_{11}:ATH]_{up} - [E^h_{11}:ATH]) \\ & +k^{off}_{e_{11}}[E^{hm}_{11}:ATH] - k^{on}_{e_{11}} p^{avail}_{111}[E^h_{11}:ATH]\end{aligned} \tag{129}$$

$$\begin{aligned}\frac{d}{dt}[E^{hm}_{11}:ATH] = {} & +k^{ATH}_{e_{11}} e^{hm}_{11}[AT:Hep] \\ & -k^{off}_{11}[E^{hm}_{11}:ATH] \\ & +k^{on}_{11} p^{avail}_{111}[E^h_{11}:ATH]\end{aligned} \tag{130}$$

S1 Table. INITIAL PLASMA LEVELS. Descriptions, notation and labels for each parameter associated with initial plasma levels are listed. The value of each parameter is found in the corresponding table listed above.

Description	Notation	Label	Table
Prothrombin	z_2	Z_2	S8
Factor V	z_5	Z_5	S8
Factor VII	z_7	Z_7	S8
Factor VIII	z_8	Z_8	S8
Factor IX	z_9	Z_9	S8
Factor X	z_{10}	Z_{10}	S8
Factor XI	z_{11}	Z_{11}	S8
TFPI	[TFPI]	TFPI	S8
AT	[AT]	AT	S8
Hep	[Hep]	Hep	S8

S2 Table. PLATELET CHARACTERISTICS. Descriptions, notation and labels for each parameter associated with platelet characteristics are listed. The value of each parameter is found in the corresponding table listed above.

Description	Notation	Label	Table
Platelet count	PL^{up}	PLup	S8
Binding site number for II	N_2	N2	S8
Binding site number for IIa	N_2^*	N2*	S8
Binding site number for V/Vh/Va	N_5	N5	S8
Binding site number for VIII/VIIIa	N_8	N8	S8
Binding site number for IX	N_9	N9	S8
Binding site number for IXa	N_9^*	N9*	S8
Binding site number for X/Xa	N_{10}	N10	S8
Binding site number for XI	N_{11}	N11	S8
Binding site number for XIa	N_{11}^*	N11*	S8
Rate of unactivated platelets adhering to SE	k^{+}_{adh}	kadh	S14
Rate of activated platelets adhering to SE	$k^{+,*}_{adh}$	kadh1	S14
Rate of platelet activation by platelet in solution	k^{act}_{plt}	kact_{plt}	S14
Rate of platelet activation on SE	$k^{act,*}_{plt}$	$\mathrm{kact*}_{plt}$	S14
Rate of platelet activation by thrombin	$k^{act}_{e_2}$	kact_{e2}	S14

S3 Table. KINETIC RATE CONSTANTS. Descriptions, notation and labels for each parameter associated with kinetic rate constants are listed. The value of each parameter is found in the corresponding table listed above.

Description	Notation	Label	Table
Rates of activation of TF:VII by fX	K_M	KZ7mE10M	S9
	$k^{cat}_{z_7^m:e_{10}}$	KZ7mE10CAT	S9
	$k^{-}_{z_7^m:e_{10}}$	KZ7mE10MI	S9
Rates of activation of fX by TF:VIIa	K_M	KZ10E7mM	S9
	$k^{cat}_{z_{10}:e_7^m}$	KZ10E7mCAT	S9
	$k^{-}_{z_{10}:e_7^m}$	KZ10E7mMI	S9
Rates of activation of fIX by TF:VIIa	K_M	KZ9E7mM	S9
	$k^{cat}_{z_9:e_7^m}$	KZ9E7mCAT	S9
	$k^{-}_{z_9:e_7^m}$	KZ9E7mMI	S9
Rates of binding of fVII/fVIIa to TF	k_7^{on}	K7ON	S9
	k_7^{off}	K7OFF	S10
Rates of activation of TF:VII by fXa	K_M	KZ7E10M	S10
	$k^{cat}_{z_7:e_{10}^m}$	KZ7E10CAT	S10
	$k^{-}_{z_7:e_{10}^m}$	KZ7E10MI	S10
Rates of activation of TF:VII by fIIa	K_M	KZ7E2M	S10
	$k^{cat}_{z_7:e_2}$	KZ7E2CAT	S10
	$k^{-}_{z_7:e_2}$	KZ7E2MI	S10
Rates of activation of TF:VII by fIXa	K_M	KZ7E9M	S10
	$k^{cat}_{z_7:e_9}$	KZ7E9CAT	S10
	$k^{-}_{z_7:e_9}$	KZ7E9MI	S10
Rates of activation of fV by fIIa	K_M	KZ5E2M	S10
	$k^{cat}_{z_5:e_2}$	KZ5E2CAT	S10
	$k^{-}_{z_5:e_2}$	KZ5E2MI	S10
Rates of activation of fVIII by fIIa	K_M	KZ8E2M	S10
	$k^{cat}_{z_8:e_2}$	KZ8E2CAT	S10
	$k^{-}_{z_8:e_2}$	KZ8E2MI	S10
Rates of activation of fIX by fXIa-fXIa	$k^{+}_{z_9:e_{11}}$	KZ9E11P	S10
	$k^{cat}_{z_9:e_{11}}$	KZ9E11CAT	S10
	$k^{-}_{z_9:e_{11}}$	KZ9E11MI	S10
Rates of activation of fIX by fXIa-fXI	$k^{+}_{z_9:e_{11}^h}$	KZ9E11P	S10
	$k^{cat}_{z_9:e_{11}^h}$	KZ9E11CAT	S10
	$k^{-}_{z_9:e_{11}^h}$	KZ9E11MI	S10
Rates of activation of fXI by fIIa	$k^{+}_{z_{11}:e_2}$	KZ11E2P	S10
	$k^{cat}_{z_{11}:e_2}$	KZ11E2CAT	S10
	$k^{-}_{z_{11}:e_2}$	KZ11E2MI	S11
Rates of binding of fX/fXa to plt. surface	k_{10}^{on}	K10ON	S11
	k_{10}^{off}	K10OFF	S11
Rates of binding of fV/fVa to plt. surface	k_5^{on}	K5ON	S11
	k_5^{off}	K5OFF	S1
Rates of binding of fVIII/fVIIIa to plt. surface	k_8^{on}	K8ON	S1
	k_8^{off}	K8OFF	S1
Rates of binding of fIX/fIXa to plt. surface	k_9^{on}	K9ON	S1
	k_9^{off}	K9OFF	S11
Rates of binding of fII/fIIa to plt. surface	k_2^{on}, $k_2^{on,*}$	K2ON, K2SON	S1
	k_2^{off}, $k_2^{off,*}$	K2OFF, K2SOFF	S11
Rates of binding of fXI/fXIa to plt. surface	k_{11}^{on}, $k_{11}^{on,*}$	K11ON, K11SON	S11
	k_{11}^{off}, $k_{11}^{off,*}$	K11OFF, K11SOFF	S12

S4 Table. KINETIC RATE CONSTANTS. Descriptions, notation and labels for each parameter associated with kinetic rate constants are listed. The value of each parameter is found in the corresponding table listed above.

Description	Notation	Label	Table
Rates of activation of fV by fXa on plt. surface	K_M	KZ5mE10mM	S12
	$k^{cat}_{z_5^m:e_{10}^m}$	KZ5mE10mCAT	S12
	$k^{-}_{z_5^m:e_{10}^m}$	KZ5mE10mMI	S12
Rates of activation of fV by fIIa on plt. surface	K_M	KZ5mE2mM	S12
	$k^{cat}_{z_5:e_2^m}$	KZ5mE2mCAT	S12
	$k^{-}_{z_5:e_2^m}$	KZ5mE2mMI	S12
Rates of activation of fVIII by fXa on plt. surface	K_M	KZ8ME10MM	S12
	$k^{cat}_{z_8:e_{10}^m}$	KZ8ME10MCAT	S12
	$k^{-}_{z_8:e_{10}^m}$	KZ8ME10MMI	S12
Rates of activation of fVIII by fIIa on plt. surface	K_M	KZ8ME2MM	S12
	$k^{cat}_{z_8:e_2^m}$	KZ8mE2mCAT	S12
	$k^{-}_{z_8:e_2^m}$	KZ8mE2mMI	S12
Rates of activation of fX by TEN on plt. surface	K_M	KZ10mTENM	S12
	$k^{cat}_{z_{10}^m:TEN}$	KZ10mTENCAT	S12
Rates of activation of fII by PRO on plt. surface	K_M	KZ2mPROM	S12
	$k^{cat}_{z_2^m:PRO}$	KZ2mPROCAT	S12
Rates of activation of fXI by fIIa on plt. surfaces	$k^{+}_{z_{11}^m:e_2^m}$	KZ11mE2mP	S12
	$k^{cat}_{z_{11}^m:e_2^m}$	KZ11mE2mCAT	S12
	$k^{-}_{z_{11}^m:e_2^m}$	KZ11mE2mMI	S12
Rates of activation of fIX by fXIa-fXIa on plt. surface	K_M	KZ9mE11mP	S12
	$k^{cat}_{z_9^m:e_{11}^m}$	KZ9mE11mCAT	S12
	$k^{-}_{z_9^m:e_2^m}$	KZ9mE11mMI	S12
Rates of formation of TEN on plt. surface	$k^{+}_{e_8^m:e_9^m}$	KE8mE9mP	S12
	$k^{-}_{e_8^m:e_9^m}$	KE8mE9mMI	S12
Rates of formation of PRO on plt. surface	$k^{+}_{e_5^m:e_{10}^m}$	KE5mE10mP	S12
	$k^{-}_{e_5^m:e_{10}^m}$	KE5mE10mMI	S12
Rates of inhibition of fXa by TFPI	$k^{+}_{tfpia:e_{10}}$	KTFPI_E10_P	S13
	$k^{-}_{tfpia:e_{10}}$	KTFPI_E10_M	S13
Rates of inhibition of TF:VIIa by TFPIa	$k^{+}_{tfpia:e_7^m}$	KTFPIa_E7m_P	S13
	$k^{-}_{tfpia:e_7^m}$	KTFPIa_E7m_M	S13
Rates of inhibition of fVa by APC on plt. surface	K_M	KE5mAPCM	S13
	$k^{cat}_{e_5^m:APC}$	KE5mAPCCAT	S13
	$k^{-}_{e_5^m:APC}$	KE5mAPCMI	S13
Rates of inhibition of fVIIIa by APC on plt. surface	K_M	KE8mAPCM	S13
	$k^{cat}_{e_8^m:APC}$	KE8mAPCCAT	S13
	$k^{-}_{e_8^m:APC}$	KE8mAPCMI	S13
Rates of inhibition of fIIa by TM on plt. surface	k^{on}_{TM}	KTMP	S13
	k^{off}_{TM}	KTMM	S14

S5 Table. NEW KINETIC RATE CONSTANTS ADDED IN TFPI EXTENSION. Descriptions, notation and labels for each parameter associated with kinetic rate constants are listed. The value of each parameter is found in the corresponding table listed above.

Description	Notation	Label	Table
Rates of binding of fV-h by fXa on plt. surface	$k^{+}_{e5hme10m}$	KE5HME10MP	S10
	$k^{-}_{e_5^m:e_{10}^m}$	KE5HME10MMI	S12
Rates of activation of II by PROh on plt. surface	K_M	KZ2MPROHM	S10
	$k^{cat}_{z_2^m:PRO^h}$	KZ2MPROHCAT	S12
	$k^{-}_{z_2^m:PRO^h}$	KZ2MPROHMI	S12
Rates of activation of fV-h by IIa on plt. surface	K_M	KE5HME2MM	S10
	$k^{cat}_{e_5^{hm}:e_2^m}$	KE5HME2MCAT	S12
	$k^{-}_{e_5^{hm}:e_2^m}$	KE5HME2MMI	S12
Rates of activation of fV-h by IIa in fluid	K_M	KE5HE2M	S10
	$k^{cat}_{e_5^h:e_2}$	KE5HE2CAT	S12
	$k^{-}_{e_5^h:e_2}$	KE5HE2MI	S12
Rates of binding of fV-h by TFPI on plt. surface	$k^{+}_{TFPI:e_5^{hm}}$	KTFPIE5HMP	S10
	$k^{-}_{TFPI:e_5^{hm}}$	KTFPIE5HMMI	S13
Rates of binding of fV-h by TFPI in fluid	$k^{+}_{TFPI:e_5^h}$	KTFPIE5HP	S10
	$k^{-}_{TFPI:e_5^h}$	KTFPIE5HMI	S13
Rates of binding of fXa by TFPI on plt. surface	$k^{+}_{TFPI:e_{10}^m}$	KTFPI_E10M_P	S10
	$k^{-}_{TFPI:e_{10}^m}$	KTFPI_E10M_MI	S13
Rates of binding of PROh by TFPI on plt. surface by binding fXa	$k^{+}_{TFPI:PRO^h:v_{10}}$	KTFPIPROHV10P	S10
	$k^{-}_{TFPI:PRO^h:v_{10}}$	KTFPIPROHV10MI	S13
Rates of binding of PROh by TFPI on plt. surface by binding fV-h	$k^{+}_{TFPI:PRO^h:v_5}$	KTFPIPROHV5P	S10
	$k^{-}_{TFPI:PRO^h:v_5}$	KTFPIPROHV5MI	S13
Rates of inactivation of fV-h by APC on plt. surface	K_M	KE5HMAPCM	S10
	$k^{cat}_{e_5^{hm}}$	KE5HMAPCCAT	S13
	$k^{-}_{e_5^{hm}:APC}$	KE5HMAPCMI	S13
Rates of inactivation of fV-h by APC in fluid	K_M	KE5HAPCM	S10
	$k^{cat}_{e_5^h}$	KE5HAPCCAT	S13
	$k^{-}_{e_5^h:APC}$	KE5HAPCMI	S13

S6 Table. NEW KINETIC RATE CONSTANTS ADDED IN AT EXTENSION. Descriptions, notation and labels for each parameter associated with kinetic rate constants are listed. The value of each parameter is found in the corresponding table listed above.

Description	Notation	Label	Table
Rates of inactivation of fIXa by AT on plt. surface	$k_{e_9^m}^{AT}$	KE9MATIII	S13
Rates of inactivation of fXa by AT on plt. surface	$k_{e_{10}^m}^{AT}$	KE10MATIII	S13
Rates of inactivation of IIa by AT on plt. surface	$k_{e_2^m}^{AT}$	KE2MATIII	S13
Rates of inactivation of fXIa by AT on plt. surface	$k_{e_{11}^m}^{AT}$	KE11MATIII	S13
Rates of inactivation of fIXa by AT in fluid	$k_{e_9}^{AT}$	KE9ATIII	S13
Rates of inactivation of fXa by AT in fluid	$k_{e_{10}}^{AT}$	KE10ATIII	S13
Rates of inactivation of IIa by AT in fluid	$k_{e_2{}^{AT}}$	KE2ATIII	S13
Rates of inactivation of fXIa by AT in fluid	$k_{e_{11}}^{AT}$	KE11ATIII	S13
Rates of binding of AT by Heparin on plt. surface	$k_{AT:Hep}^{+}$	KATBHEPMI	S13
	$k_{AT:Hep}^{-}$	KATBHEPMI	S13
Rates of inactivation of fIXa by ATH on plt. surface	$k_{e_9^m}^{ATH}$	KE9MATH	S13
Rates of inactivation of fXa by ATH on plt. surface	$k_{e_{10}^m}^{ATH}$	KE10MATH	S13
Rates of inactivation of IIa by ATH on plt. surface	$k_{e_2^m}^{ATH}$	KE2MATH	S13
Rates of inactivation of fXIa by AT on plt. surface	$k_{e_{11}^m}^{ATH}$	KE11MATH	S13
Rates of inactivation of fIXa by ATH in fluid	$k_{e_9}^{ATH}$	KE9ATH	S13
Rates of inactivation of fXa by ATH in fluid	$k_{e_{10}}^{ATH}$	KE10ATH	S13
Rates of inactivation of IIa by ATH in fluid	$k_{e_2}^{ATH}$	KE2ATH	S13
Rates of inactivation of fXIa by AT in fluid	$k_{e_{11}}^{ATH}$	KE11ATH	S13

Kinetic and Physical Parameter Values:

S7 Table. DIFFUSION COEFFICIENTS FOR PLATELETS AND FLUID-PHASE CHEMICAL SPECIES (a) From (4). (b) From (5).

Platelets	2.5×10^{-7} cm^2/s	a
Proteins	5×10^{-7} cm^2/s	b

S8 Table. NORMAL CONCENTRATIONS AND SURFACE BINDING SITE NUMBERS (a) From (6). (b) From (7). (c) (8) suggests that normal plasma concentration of fVIIa is about 1% of the normal fVII concentration. (d) From (9). (e) (f) From (10). (g) Estimated as described in the text of the Supplementary Information. (h) From (11). (i) From (12). (j) From (13). (k) From (14). (l) From (15, 16). (m) Number of fV molecules released per activated platelet (17). (n) Maximum concentration of platelets in a 2 μm high reaction zone assuming that 20 platelets can cover a 10μm-by-10μm injured surface (18). (o) From (19). (p) Refer to heparin dosage calculation in later section of supplemental material.

Prothrombin	1.4 μM	a
Factor V	0.01 μM	b
Factor VII	0.01 μM	a
Factor VIIa	0.1 nM	c
Factor VIII	1.0 nM	a
Factor IX	0.09 μM	a
Factor X	0.17 μM	a
Factor XI	30.0 nM	a
TFPI	0.5 nM	d
Protein C	65 nM	e
Platelet count	$2.5(10)^5/\mu$l	f
N_2	1000/plt	g
N_2^*	1000/plt	g
N_5	3000/plt	h
N_8	450/plt	i
N_9	250/plt	j
N_9^*	250/plt	j
N_{10}	2700/plt	k
N_{11}	1500/plt	l
N_{11}^*	250/plt	l
n_5	3000/plt	m
p_{PLAS}	0.167 nM	n
AT	2.4 nM	o
$LMWH$	253 nM	p
UFH	759 nM	p

S9 Table. REACTIONS ON SUBENDOTHELIUM (a) $k^{cat}_{z_7^m:e_{10}} = 5.0$ sec^{-1} and $K_M = 1.2 \cdot 10^{-6}$ M (20). (b) $k^{cat}_{z_7^m:e_2} = 6.1 \cdot 10^{-2}$ sec^{-1} and $K_M = 2.7 \cdot 10^{-6}$ M (20). (d) $k^{cat}_{z_{10}:e_7^m} = 1.15$ sec^{-1} and $K_M = 4.5 \cdot 10^{-7}$ M (6). (d) $k^{cat}_{z_9:e_7^m} = 1.15$ sec^{-1} and $K_M = 2.4 \cdot 10^{-7}$ M (21). (e) $K_d = 1.0 \cdot 10^{-10}$ M (22).

Activation (of -, by -)							
(TF:VII,fXa)	E_{10}, Z_7^m	$Z_7^m : E_{10}$	E_7^m	$k^+_{z_7^m:e_{10}}$=5.0 · 10^6	$k^-_{z_7^m:e_{10}}$=1.0	$k^{cat}_{z_7^m:e_{10}}$=5.0	a
(TF:VII, fIIa)	E_2, Z_7^m	$Z_7^m : E_2$	E_7^m	$k^+_{z_7^m:e_2}$=3.92 · 10^5	$k^-_{z_7^m:e_2}$=1.0	$k^{cat}_{z_7^m:e_2}$=6.1 · 10^{-2}	b
(fX, TF:VIIa)	E_7^m, Z_{10}	$Z_{10} : E_7^m$	E_{10}	$k^+_{z_{10}:e_7^m}$=5.0 · 10^6	$k^-_{z_{10}:e_7^m}$=1.0	$k^{cat}_{z_{10}:e_7^m}$=1.15	c
(fIX, TF:VIIa)	E_7^m, Z_9	$Z_9 : E_7^m$	E_9	$k^+_{z_9:e_7^m}$=9.4 · 10^6	$k^-_{z_9:e_7^m}$=1.0	$k^{cat}_{z_9:e_7^m}$=1.15	d
Binding (of -, with -)							
(fVII, TF)	Z_7, TF		Z_7^m	k_7^{on}=5.0 · 10^7	k_7^{off}=5.0 · 10^{-3}		e
(fVIIa, TF)	E_7, TF		E_7^m	k_7^{on}=5.0 · 10^7	k_7^{off}=5.0 · 10^{-3}		e

S10 Table. REACTIONS IN THE PLASMA (a) $k^{\text{cat}}_{z_7:e_{10}} = 5.0\,\text{sec}^{-1}$ and $K_M = 1.2 \cdot 10^{-6}$ M (20). (b) $k^{\text{cat}}_{z_7:e_2} = 6.1 \cdot 10^{-2}\,\text{sec}^{-1}$ and $K_M = 2.7 \cdot 10^{-6}$ M (20) (c) $k^{\text{cat}}_{z_5:e_2} = 0.23\,\text{sec}^{-1}$ and $K_M = 7.17 \cdot 10^{-8}$ M (23). (d) $k^{\text{cat}}_{z_8:e_2} = 0.9\,\text{sec}^{-1}$ (24) and $K_M = 2 \cdot 10^{-7}$ M (25). (e) $k^{\text{cat}}_{z_{11}:e_2} = 1.3 \cdot 10^{-4}$, $K_M = 50$nM (26). Rate constants apply also for thrombin-activation of XIa-XI. (f) $k^{\text{cat}}_{z_9:e^h_{11}} = 0.21$, $K_M = 0.2\mu$M (27, 28). Rate constants apply also for activation of IX by XIa-XIa.

Reaction	Reactants	Complex	Product	$M^{-1}\text{sec}^{-1}$	sec^{-1}	sec^{-1}	Note
Activation (of -, by -)							
(fVII, fXa)	Z_7, E_{10}	$Z_7 : E_{10}$	E_7	$k^+_{z_7:e_{10}}$=5 $\cdot 10^6$	$k^-_{z_7:e_{10}}$=1.0	$k^{\text{cat}}_{z_7:e_{10}}$=5.0	a
(fVII, fIIa)	Z_7, E_2	$Z_7 : E_2$	E_7	$k^+_{z_7:e_2}$=3.92 $\cdot 10^5$	$k^-_{z_7:e_2}$=1.0	$k^{\text{cat}}_{z_7:e_2}$=6.1 $\cdot 10^{-2}$	b
(fV, fIIa)	Z_5, E_2	$Z_5 : E_2$	E_5	$k^+_{z_5:e_2}$= 1.73 $\cdot 10^7$	$k^-_{z_5:e_2}$=1.0	$k^{\text{cat}}_{z_5:e_2}$=0.23	c
(fVIII, fIIa)	Z_8, E_2	$Z_8 : E_2$	E_8	$k^+_{z_8:e_2}$=2.64 $\cdot 10^7$	$k^-_{z_8:e_2}$=1.0	$k^{\text{cat}}_{z_8:e_2}$=0.9	d
(fXI-fXI, fIIa)	Z_{11}, E_2	$Z_{11} : E_2$	E^h_{11}	$k^+_{z_{11}:e_2}$ = 2.0 $\cdot 10^7$	$k^-_{z_{11}:e_2}$ = 1.0	$k^{\text{cat}}_{z_{11}:e_2}$ = 1.3 $\cdot 10^{-4}$	e
(fIX, fXIa)	Z_9, E^h_{11}	$Z_9 : E^h_{11}$	E_9	$k^+_{z_9:e^h_{11}}$ = 0.6 $\cdot (10)^7$	$k^-_{z_9:e^h_{11}}$ = 1.0	$k^{\text{cat}}_{z_9:e^h_{11}}$ = 0.21	f

S11 Table. BINDING TO PLATELET SURFACES (a) For fIX binding to platelets, $K_d = 2.5 \cdot 10^{-9}$ M (13), and for fX binding to platelets, K_d has approximately the same value (11). For fX binding to PCPS vesicles, the on-rate is about 10^7 $M^{-1}sec^{-1}$ and the off-rate is about 1.0 sec^{-1} (29) giving a dissociation constant of about 10^{-7} M. To estimate on- and off-rates for the higher-affinity binding of fX to platelets, we keep the on-rate the same as for vesicles and adjust the off-rate to give the correct dissociation constant. The rates for fIX binding with platelets are taken to be the same as for fX binding. (b) We assume binding constants for fIXa binding to the specific fIXa binding sites are the same as for shared sites. (c) fV binds with high-affinity to phospholipids (PCPS) (29) and we use the same rate constants reported there to describe fV binding to platelets. (d) The K_d for fVIII binding with platelets is taken from (12). We set the off-rate k_8^{off} for fVIII binding to platelets equal to that for fV binding to platelets, and calculate the on-rate k_8^{on}. (e) For prothrombin interactions with platelets, K_d is reported to be $5.9 \cdot 10^{-7}$ M (30). We choose k_2^{off} and set $k_2^{\text{on}} = k_2^{\text{off}}/K_d$. (f) Estimated as described in the text of the Supplementary Information. (g) $K_d = 10$ nM (31). (h) $K_d = 1.7$ nM (16).

Reaction	Reactants	Products	$M^{-1}sec^{-1}$	sec^{-1}	Note
Factor IX	Z_9, P_9	Z_9^m	k_9^{on}=$1.0 \cdot 10^7$	k_9^{off}=$2.5 \cdot 10^{-2}$	a
Factor IXa	E_9, P_9	E_9^m	k_9^{on}=$1.0 \cdot 10^7$	k_9^{off}=$2.5 \cdot 10^{-2}$	a
Factor IXa	E_9, P_9^*	$E_9^{m,*}$	k_9^{on}=$1.0 \cdot 10^7$	k_9^{off}=$2.5 \cdot 10^{-2}$	b
Factor X	Z_{10}, P_{10}	Z_{10}^m	k_{10}^{on}=$1.0 \cdot 10^7$	k_{10}^{off}=$2.5 \cdot 10^{-2}$	a
Factor Xa	E_{10}, P_{10}	E_{10}^m	k_{10}^{on}=$1.0 \cdot 10^7$	k_{10}^{off}=$2.5 \cdot 10^{-2}$	a
Factor V	Z_5, P_5	Z_5^m	k_5^{on}=$5.7 \cdot 10^7$	k_5^{off}=0.17	c
Factor Vh	E_5^h, P_5	E_5^{hm}	k_5^{on}=$5.7 \cdot 10^7$	k_5^{off}=0.17	c
Factor Va	E_5, P_5	E_5^m	$k_5^{\text{on}} = 5.7 \cdot 10^7$	k_5^{off}=0.17	c
Factor VIII	Z_8, P_8	Z_8^m	$k_8^{\text{on}} = 5.0 \cdot 10^7$	k_8^{off}=0.17	d
Factor VIIIa	E_8, P_8	E_8^m	$k_8^{\text{on}} = 5.0 \cdot 10^7$	k_8^{off}=0.17	d
Factor II	Z_2, P_2	Z_2^m	$k_2^{\text{on}} = 1.0 \cdot 10^7$	k_2^{off}=5.9	e
Factor IIa	E_2, P_2	E_2^m	$k_2^{*,\text{on}} = 1.0 \cdot 10^7$	$k_2^{*,\text{off}} = 0.2$	f
Factor XI	Z_{11}, P_{11}	Z_{11}^m	$k_{z_{11}}^{\text{on}} = 1.0 \cdot 10^7$	$k_{z_{11}}^{\text{off}}$=0.1	g
Factor XIa	E_{11}, P_{11}^*	E_{11}^m	$k_{e_{11}}^{\text{on}} = 1.0 \cdot 10^7$	$k_{e_{11}}^{\text{off}}$=0.017	h

S12 Table. REACTIONS ON PLATELET SURFACES (a) $k^{\text{cat}}_{z_5^m:e_{10}^m} = 0.046$ sec^{-1} and $K_M = 10.4 \cdot 10^{-9}$ M (32). (b) The rate constants for thrombin activation of fV on platelets are assumed to be the same as in plasma. (c) $k^{\text{cat}}_{z_8^m:e_{10}^m} = 0.023$ sec^{-1} and $K_M = 2.0 \cdot 10^{-8}$ M (25). (d) The rate constants for thrombin activation of fVIII on platelets are assumed to be the same as in plasma. (e) The formation of the tenase and prothrombinase complexes is assumed to be very fast with $K_d = 1.0 \cdot 10^{-10}$ M (33). (f) $k^{\text{cat}}_{z_{10}^m:ten} = 20$ sec^{-1} and $K_M = 1.6 \cdot 10^{-7}$ M (34). (g) $k^{\text{cat}}_{z_2^m:pro} = 30$ sec^{-1} and $K_M = 3.0 \cdot 10^{-7}$ M (35). (h) $k^{\text{cat}}_{z_{11}^m:e_2^m} = 1.3 \cdot 10^{-4}$, $K_M = 50$ nM (26). Rate constants apply also for thrombin-activation of Plt-XIa-XI. (i) $k^{\text{cat}}_{z_9^m:e_{11}^{h,m}} = 0.21$, $K_M = 0.2\mu$M (27, 28). Rate constants apply also for activation of platelet-bound IX by Plt-XIa-XIa.

Reaction	Reactants	Complex	Product	M^{-1}sec^{-1}	sec^{-1}	sec^{-1}	Note
Activation (of -, by -)							
(V, Xa)	Z_5^m, E_{10}^m	$Z_5^m : E_{10}^m$	E_5^{hm}	$k^+_{z_5^m:e_{10}^m}$=1.0 · 10^8	$k^-_{z_5^m:e_{10}^m}$=1.0	$k^{\text{cat}}_{z_5^m:e_{10}^m}$=4.6 · 10^{-2}	a
(V, IIa)	Z_5^m, E_2^m	$Z_5^m : E_2^m$	E_5^m	$k^+_{z_5^m:e_2^m}$=1.73 · 10^7	$k^-_{z_5^m:e_2^m}$=1.0	$k^{\text{cat}}_{z_5^m:e_2^m}$= 0.23	b
(Vh, IIa)	E_5^{hm}, E_2^m	$E_5^{hm} : E_2^m$	E_5^m	$k^+_{z_5^m:e_2^m}$=1.73 · 10^7	$k^-_{z_5^m:e_2^m}$=1.0	$k^{\text{cat}}_{z_5^m:e_2^m}$= 0.23	b
(VIII, Xa)	Z_8^m, E_{10}^m	$Z_8^m : E_{10}^m$	E_8^m	$k^+_{z_8^m:e_{10}^m}$=5.1 · 10^7	$k^-_{z_8^m:e_{10}^m}$=1.0	$k^{\text{cat}}_{z_8^m:e_{10}^m}$=2.3 · 10^{-2}	c
(VIII, IIa)	Z_8^m, E_2^m	$Z_8^m : E_2^m$	E_8^m	$k^+_{z_8^m:e_2^m}$=2.64 · 10^7	$k^-_{z_8^m:e_2^m}$=1.0	$k^{\text{cat}}_{z_8^m:e_2^m}$= 0.9	d
(X, VIIIa:IXa)	Z_{10}^m, TEN	$Z_{10}^m : TEN$	E_{10}^m	$k^+_{z_{10}^m:ten}$=1.31 · 10^8	$k^-_{z_{10}^m:ten}$=1.0	$k^{\text{cat}}_{z_{10}^m:ten}$=20.0	f
(X, VIIIa:IXa*)	Z_{10}^m, TEN^*	$Z_{10}^m : TEN^*$	E_{10}^m	$k^+_{z_{10}^m:ten}$=1.31 · 10^8	$k^-_{z_{10}^m:ten}$=1.0	$k^{\text{cat}}_{z_{10}^m:ten}$=20.0	f
(II, Vh:Xa)	$Z_2^m, PROh$	$Z_2^m : PROh$	E_2^m	$k^+_{z_2^m:pro}$= 1.03 · 10^8	$k^-_{z_2^m:pro}$=1.0	$k^{\text{cat}}_{z_2^m:pro}$=30.0	g
(II, Va:Xa)	Z_2^m, PRO	$Z_2^m : PRO$	E_2^m	$k^+_{z_2^m:pro}$= 1.03 · 10^8	$k^-_{z_2^m:pro}$=1.0	$k^{\text{cat}}_{z_2^m:pro}$=30.0	g
(XI-XI, IIa)	Z_{11}^m, E_2^m	$Z_{11}^m : E_2^m$	E_{11}^{hm}	$k^+_{z_{11}^m:e_2^m}$ = 2.0 · 10^7	$k^-_{z_{11}^m:e_2^m}$ = 1.0	$k^{\text{cat}}_{z_{11}^m:e_2^m}$ = 1.3 · 10^{-4}	h
(IX, XIa)	Z_9^m, E_{11}^{hm}	$Z_9^m : E_{11}^{hm}$	E_9	$k^+_{z_9^m:e_{11}^m}$ = 0.6 · 10^7	$k^-_{z_9^m:e_{11}^m}$ = 1.0	$k^{\text{cat}}_{z_9^m:e_{11}^m}$ = 0.21	i
Binding (of -, with -)							
(VIIIa, IXa)	E_8^m, E_9^m		TEN	k^+_{ten}=1.0 · 10^8	k^-_{ten}=0.01		e
(VIIIa, IXa*)	$E_8^m, E_9^{m,*}$		TEN^*	k^+_{ten}=1.0 · 10^8	k^-_{ten}=0.01		e
(Vh, Xa)	E_5^{hm}, E_{10}^m		$PROh$	k^+_{pro}=1.0 · 10^8	k^-_{pro}=0.01		e
(Va, Xa)	E_5^m, E_{10}^m		PRO	k^+_{pro}=1.0 · 10^8	k^-_{pro}=0.01		e

S13 Table. INHIBITION REACTIONS (a) From (19). (b) From (36). (c) For inhibition of fVa by APC, $k^{\text{cat}}_{e_5^m:APC} = 0.5$ sec^{-1} and $K_M = 12.5 \cdot 10^{-9}$ (37). We assume the same reaction rates for the inhibition of fVIIIa by APC. (d) From (38). (e)From (39). (f) From (40). (g) $K_d = 0.5$ nM and $[PC] = 65$ nM (41). (h)From (42). (i) $k_{PC:TM:e_2^{ec}} = 0.167$ sec^{-1} , $K_M = 0.7 \cdot 10^{-6}$ M (43).

Reaction	Reactants	Product	$M^{-1}sec^{-1}$	sec^{-1}	sec^{-1}	Note
Inactivation (of -, by -)						
(IXa, AT-III)	E_9, AT	$E_9 : AT$		$k_{e_9}^{AT}$=4.8 · 10^2		a
(Xa, AT-III)	E_{10}, AT	$E_{10} : AT$		$k_{e_{10}}^{AT}$=3.5 · 10^3		a
(IIa, AT-III)	E_2, AT	$E_2 : AT$		$k_{e_2}^{AT}$=1.4 · 10^4		a
(XIa, AT-III)	E_{11}, AT	$E_{11} : AT$		$k_{e_{11}}^{AT}$=2.4 · 10^2		a
(XIa:AT, AT-III)	$E_{11} : AT$, AT	$AT : E_{11} : AT$		$k_{e_{11}}^{AT}$=2.4 · 10^2		a
(IXa, ATH)	E_9, ATH	$E_9 : ATH$		$k_{e_9}^{AT}$=5 · 10^5		b
(Xa, ATH)	E_{10}, ATH	$E_{10} : ATH$		$k_{e_{10}}^{AT}$=1.3 · 10^6		b
(IIa, ATH)	E_2, ATH	$E_2 : ATH$		$k_{e_2}^{AT}$=5.3 · 10^6		b
(XIa, ATH)	E_{11}, ATH	$E_{11} : ATH$		$k_{e_{11}}^{AT}$=1 · 10^4		b
(XIa:AT, ATH)	$E_{11} : AT$, ATH	$ATH : E_{11} : ATH$		$k_{AT:e_{11}}^{in}$=1 · 10^4		b
(APC, Va)	$APC,\ E_5^m$	none	$k_{e_5^m:APC}^{+} = 1.2 \cdot 10^8$	$k_{e_5^m:APC}^{-} = 1.0$	$k_{e_5^m:APC}^{\text{cat}} = 0.5$	c
(APC, VIIIa)	$APC,\ E_8^m$	none	$k_{e_8^m:APC}^{+} = 1.2 \cdot 10^8$	$k_{e_8^m:APC}^{-} = 1.0$	$k_{e_8^m:APC}^{\text{cat}} = 0.5$	c
Binding (of -, with -)						
(TFPI, Xa)	$TFPI$,E_{10}	$TFPIa$	$k_{tfpia:e_{10}}^{+}$=1.6 · 10^7	$k_{tfpia:e_{10}}^{-}$=3.3 · 10^{-4}		d
(TFPI, Vh)	$TFPI,\ E_5^h$	$TFPI : E_5^h$	$k_{tfpi:e5h}^{+} = 0.05 \cdot 10^9$	$k_{tfpi:e5h}^{-} = 0.0045$		e
(TFPI:Xa, Vh)	$TFPIa,\ E_5^h$	$E_5^h : TFPI : E_{10}$	$k_{tfpi:e5h}^{+} = 0.05 \cdot 10^9$	$k_{tfpi:e5h}^{-} = 0.0045$		e
(TFPI:Vh, Xa)	$TFPI : E_5^h, E_{10}$	$E_5^h : TFPI : E_{10}$	$k_{tfpia:e_{10}}^{+}$=1.6 · 10^7	$k_{tfpia:e_{10}}^{-}$=3.3 · 10^{-4}		d
(Xa:Vh, TFPI)	$E_{10} : E_5^h, TFPIa$	$E_{10} : E_5^h : TFPI$	$k_{tfpibprohv5}^{+} = 0.05 \cdot 10^9$	$k_{tfpibprohv5}^{-} = 0.0045$		e
(Xa:Vh, TFPI)	$E_{10} : E_5^h, TFPIa$	$TFPI : E_{10} : E_5^h$	$k_{tfpibprohv10}^{+} = 1.6 \cdot 10^7$	$k_{tfpibprohv10}^{-} = 3.3 \cdot 10^-4$		d
(TFPIa, TF:VIIa)	$TFPIa$,E_7^m	$TFPIa : E_7^m$	$k_{tfpia:e_{10}}^{+}$=1.6 · 10^7	$k_{tfpia:e_{10}}^{-}$=3.3 · 10^{-4}		f
(TM, Thrombin)	$TM,\ E_2^{ec}$	$TM : E_2^{ec}$	$k_{TM}^{\text{on}} = 1.0 \cdot 10^8$	$k_{TM}^{\text{off}} = 5.0 \cdot 10^{-2}$		g
(AT-III, Heparin)	AT, Hep	ATH	$k_{ATH}^{+} = 1.0$	$k_{ATH}^{-} = 2.77 \cdot 10^7$		h
Activation (of -, by -)						
(PC, TM:E_2^{ec})	$TM : E_2^{ec}$	APC	$k_{PC:TM:e_2^{ec}}^{+} = 1.7 \cdot 10^6$	$k_{PC:TM:e_2^{ec}}^{-} = 1.0$	$k_{PC:TM:e_2^{ec}}^{\text{cat}} = 0.16$	i

S14 Table. PLATELET TRANSITIONS (a) Estimated from data in (44, 45) as described in (46). (b) Estimated from data in (47) as described in (46). SE=subendothelium.

Reactants	Reactants	Products	$M^{-1}sec^{-1}$	sec^{-1}	Note
Unactivated platelet adhering to SE	PL, SE	PL_a^s	k_{adh}^{+}=2 $\cdot 10^{10}$	k_{adh}^{-}=0	a
Activated platelet adhering to SE	PL_a^v, SE	PL_a^v	k_{adh}^{+}=2 $\cdot 10^{10}$	k_{adh}^{-}=0	a
Platelet activation by platelet in solution	PL, PL_a^v	$2PL_a^v$	$k_{plt}^{\text{act}} = 3 \cdot 10^8$		b
Platelet activation on SE	PL, PL_a^s	PL_a^v, PL_a^s	$k_{plt}^{\text{act}} = 3 \cdot 10^8$		b
Platelet activation by thrombin	PL, E_2	PL_a^v		$k_{e_2}^{\text{act}}$=0.50	b

S3 CONVERSION OF HEPARIN POTENCY TO MOLAR CONCENTRATION

Based on the recommended dosage of heparin treatment (0.3-0.7 U/ml) (48), we use the value of 0.5 U/ml. Based on the information from second international standard for heparin by WHO, the conversion factor will be 130 U/mg (49). By using the mean molecular weight of heparin as 15kDa (50), we can get:
0.5 U/ml * 1/130 mg/U = 0.0038 mg/ml
$0.0038 g/L * 1/15000 mol/g = 2.53 * 10^{-}7M = 253 nM$
For the LMWH, since the molecular weight of LMWH varies based on different product (51), we use 5kDa as its molecular weight. Therefore, the concentration of heparin at 100% is determined by:
$0.0038 g/L * 1/5000 mol/g = 2.53 * 10^{-}7M = 759 nM$
Both concentrations were set as 100% baseline dosage concentration. For example, when we use 50% concentration of UFH and LMWH, we are using 126.5 nM and 379.5 nM respectively.

S4 LOCAL SENSITIVITY ANALYSIS - METHOD

As in our previous work (52), we again focus on the sensitivity of three special thrombin metrics:

1. Lag time: A measure of how fast the system is turned on, defined as the amount of time required for thrombin to reach 1 nM.
2. Maximum relative rate: A measure of how fast thrombin is produced once the system is turned on, defined as

$$\max_{t>t_{1nM}} \left(\frac{d[\text{thrombin}]}{dt}/[\text{thrombin}]\right).$$

3. Final concentration: The thrombin concentration after 20 minutes of clotting activity.

We examine the sensitivity of these metrics to two types of parameter variations: (i) the plasma levels of seven zymogens and two inhibitors, and (ii) the values of 24 new kinetic parameters that are related to TFPI reactions. We used a derivative-based approach to quantify the sensitivity of each metric with respect to centered difference in the parameters in a range of values (50%, 75%, 100%, 125% and 150% for the plasma level parameters, and 90%, 95%, 100%, 105% and 110% for kinetic parameters). The standard values for each plasma level parameters were set to the initial conditions. Similar to our previous SA results (52), we found that each of the metrics behaved monotonically with respect to varying each plasma level from 50% to 150% of the standard values, as shown in Fig. (S2A-C) and the kinetic parameters from 90% to 110% as shown in Fig. (S3A-C). The min/max values of these monotonic curves shows the change in the thrombin metric due to the factor change. Clotting factor variations had a significant effect on all three thrombin metrics but the largest change in the thrombin metrics due to variations in the kinetic parameters was less than 0.2% and therefore, we did not characterize the sensitivity of these parameters further. For the clotting factors and inhibitors, we quantified their sensitivity by the absolute difference they produced in each metric when considering their extremal values (i.e., 50% and 150%). For each metric, we ranked the parameters by considering their relative absolute difference. We define $x = (x_1, x_2, ..., x_p)$ to be the standard model parameter values and $m_i(x_{j,y\%})$ to be the values of the i-th metric when parameter j is chosen to be y% of its standard value and all other parameters are chosen to be at their standard value. The local sensitivity of the i-th metric to the j-th parameter is then:

$$LS_j^i = \frac{|m_i(x_{j,150\%} - m_i(x_{j,50\%}))|}{\max_k(|m_i(x_{k,150\%} - m_i(x_{k,50\%}))|)}$$

Each sensitivity score, LS, is then a number between 0 and 1 and we use these values to rank the input sensitivities. In our results, we denote LS scores higher than 0.75 with solid black triangles, LS scores from 0.25 to 0.75 as gray triangles, and for LS scores lower than 0.25 we use open triangles. In addition, because the response of the system outputs was monotonic throughout the entire range, we show separately the change in each metric for the 50% increase with the triangle upward and a 50% decrease with a triangle facing downward. Then the y-value of the triangle corresponds to its result on the output.

The local SA results in Fig. (S2D-I) reveal the most influential clotting factors and inhibitors, when perturbed one at a time for each of the three thrombin metrics. Fig. (S2D) shows that FVIII and FX have the greatest effect on the lag time, where an increase in either FVIII or FX levels by 50% leads to an approximately 10% decrease in the lag time from baseline. This is seen with the solid black (LS scores above 0.75), upward-facing (increase in factor level) triangles, with y-value near -10% showing the decrease in lag time. Comparing with sesitivity results from our old model((52)), we see an increased sensitivity to TFPI, where a decrease/increase by 50% leads to about a 8% decrease/5% increase in the lag time from baseline, respectively, although the TFPI LS score still does not reach 0.75. Fig. (S2E) shows that variations in FVIII, FIX, and FX have the largest effect on the maximum relative rate of thrombin generation, and this metric still has low sensitivity to TFPI. These findings are the same as in our previous results and make sense since these factors influence the rate of formation of the tenase complex on platelets, which affects the amplification stage of coagulation, and the inhibitory effect by TFPI does not alter such amplification process. It also indicates that new TFPI inhibitory reactions does not have significant influence towards the rate at which thrombin is being made. Fig. (S2F) shows that the final concentration metric is sensitive only to prothrombin (FII) as was found previously((52)). The corresponding LS scores are shown in Fig. (S2H-I).

Fig.(S3) demonstrates the local SA results for each of kinetic parameters that are related to TFPI reactions. Forward and reverse rate for each of the reaction are varied by 10% and change in lag time, maximum relative rate and final concentrations were observed. The results indicate that slight perturbation in reaction kinetics has minimal effect towards these three thrombin metrics, where none of the kinetic parameter caused more than 1% change from baseline in each cases. Such insensitiveness of the kinetic parameters, however, indicates the tolerance of the model towards the possible error in the kinetic parameters retrieved from experimental design.

S5 OTHER FIGURES

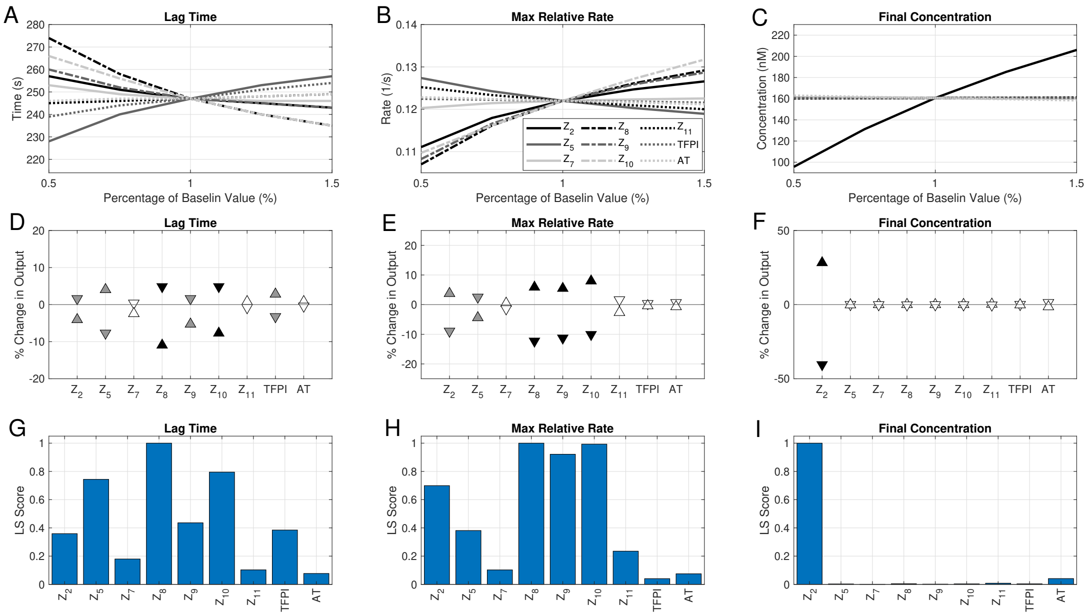

Figure S2: Local sensitivity analysis of clotting factor levels on thrombin metrics. The initial conditions of clotting factor and inhibitor levels were varied between 50% and 150% of their baseline values. Shown are (A,B,C) the amplitude change in lag time, maximum relative rate, and final thrombin concentration, (D,E,F) the percentage change in each of the metrics, and (G,H,I) the LS scores for each metric and for each species. Solid black triangles represent the species with LS score higher than 0.75, gray triangles for LS scores from 0.25 to 0.75, and open triangles for LS lower than 0.25. The arrow direction indicates if the variable was increased or decreased.

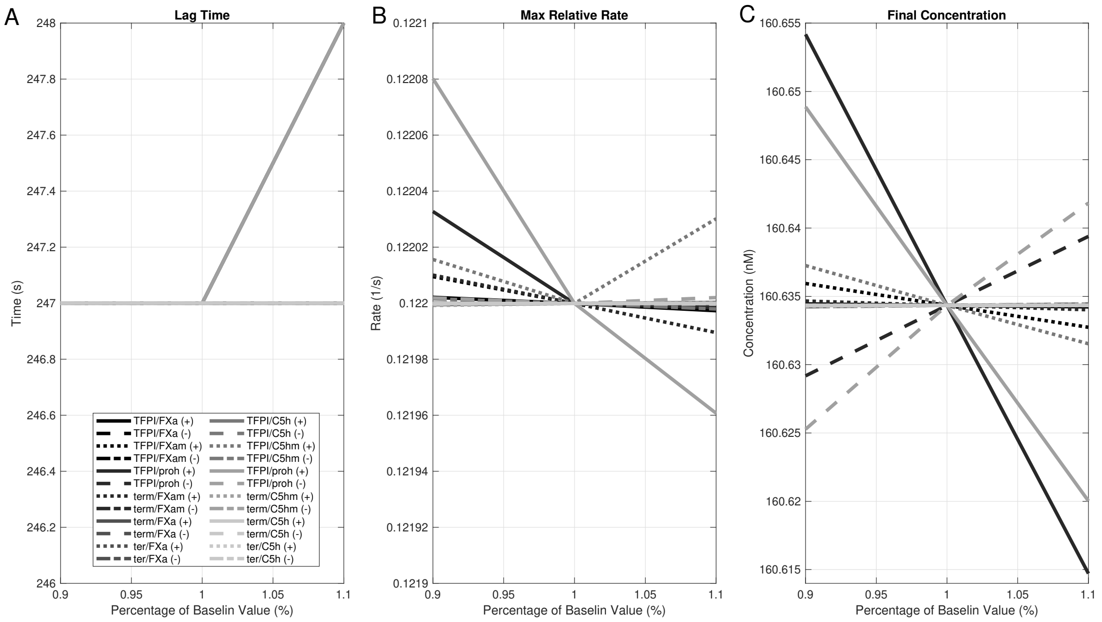

Figure S3: Local sensitivity analysis of TFPI-related kinetic rates on thrombin metrics. The new kinetic parameters were varied between 90% and 110% of their baseline values. Shown are (A,B,C) the amplitude of the changes in the lag time, maximum relative rate and final thrombin concentration due to the kinetic parameter variations. The plus/minus sign indicates the association/dissociation rate, respectively. Lower case *m* represents the components that are bound to platelet surface. The forward slash shows which two components are interacting each other, while the "ter" and "term" indicates interactions that involve a ternary complex and whether the species is in plasma or bound to the platelet surface, respectively. For example: term/FXa (+) indicates the rate of association between the platelet-bound TFPI:FV-h complex and the fluid phase FXa to form the ternary complex FXa:TFPI:FV-h.

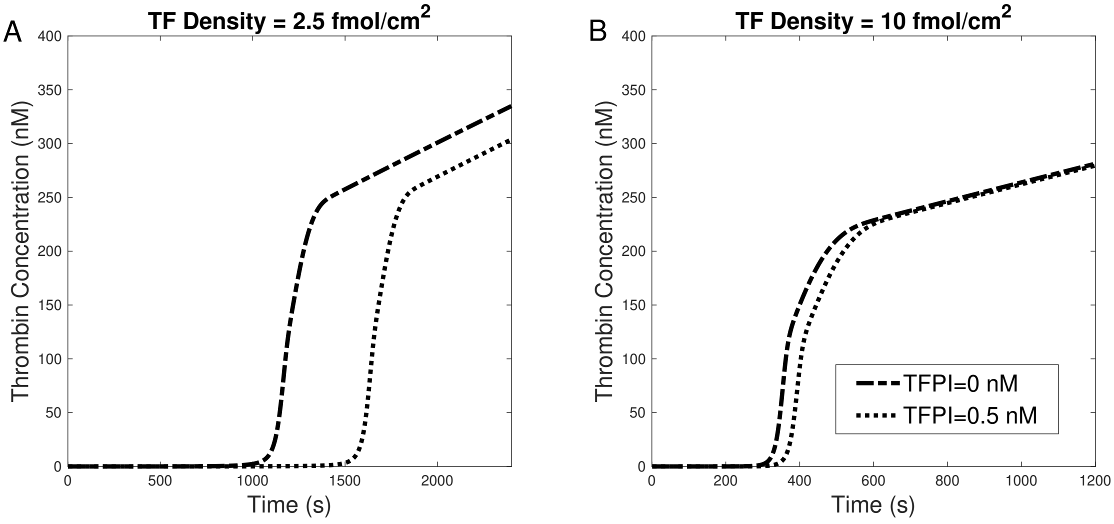

Figure S4: Thrombin generation time courses under different TFPI levels (0 nM and 0.5 nM) plotted in linear scale. TF level is varied by 2.5 fmol/cm^2 (A) and 10 fmol/cm^2 (B). Shear rate is fixed at 100/s.

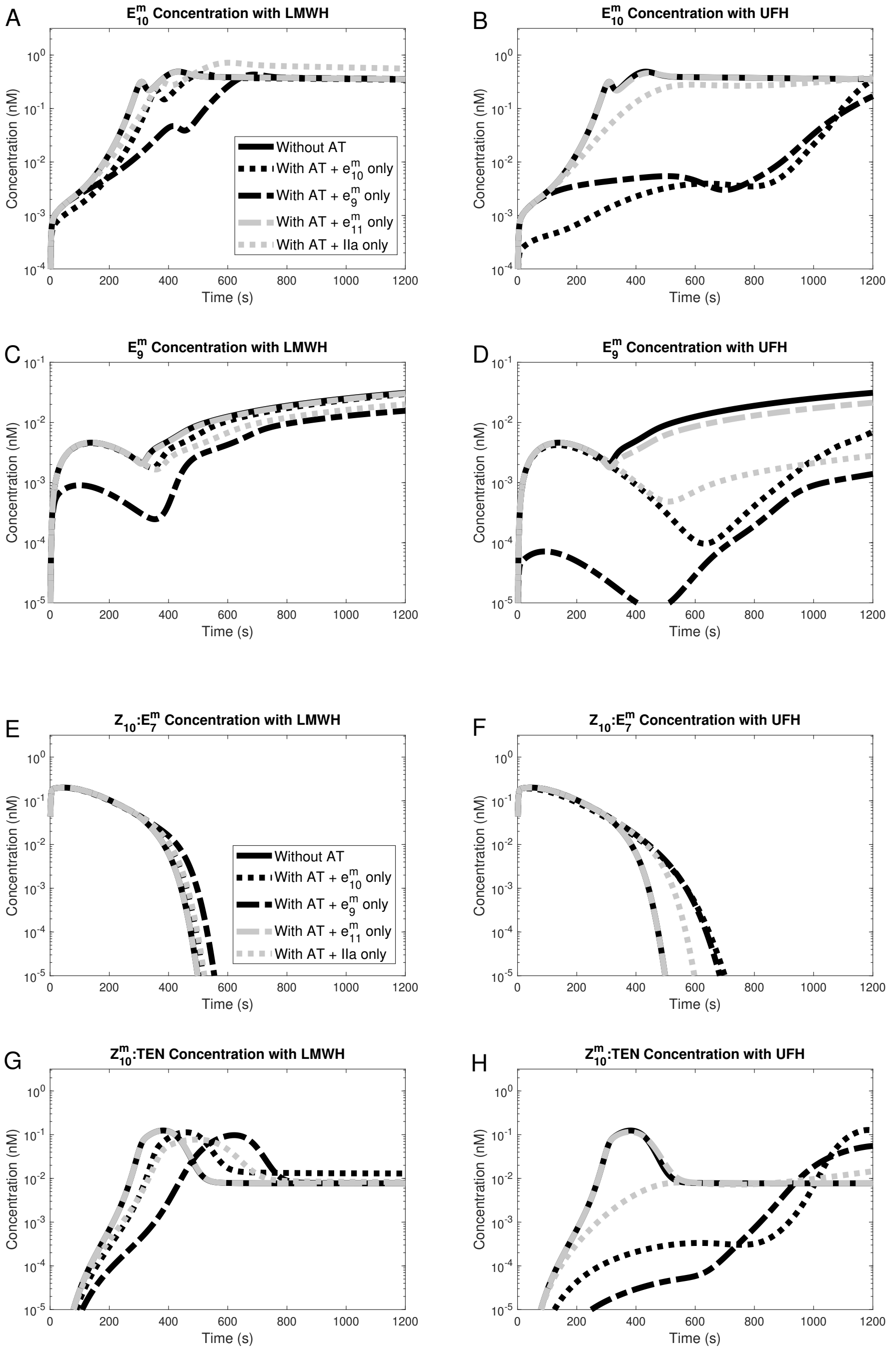

Figure S5: FXa concentration in the presence of LMWH (A) or UFH (B), FIXa concentration in the presence of LMWH (C) or UFH (D), FX:TF:VIIa concentration in the presence of LMWH (E) or UFH (F), and FX:tenase concentration in the presence of LMWH (G) or UFH (H). The time course is obtained from simulations in which we turn off all the AT-mediated inactivation reactions and then allow inhibition of FXa, FIXa, FXIa and thrombin, individually and one by one. Each curve thus shows thrombin/tenase generation when there is either no or only one inactivation reaction that exists in the system. TF density was set to 15 fmol/cm^2 and shear rate was set to 100/s. Heparin concentration is fixed to 100% of the standard therapeutic concentration.

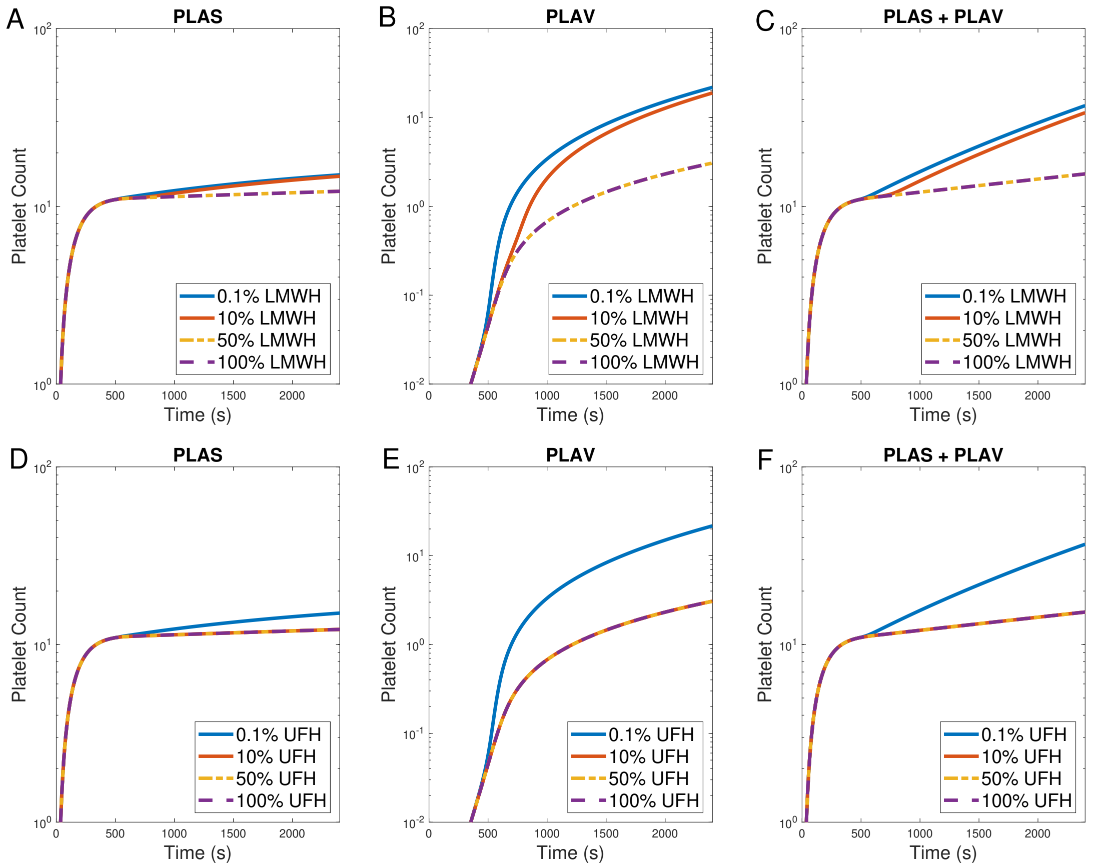

Figure S6: Subendothelium-attacehed platelet count (PLAS) and paltelet-attached-activated platelet count (PLAV) time course with varied LMWH treatmemnt (A-C) or UFH treatment (D-F). TF level is fixed to 6 $fmol/cm^2$. We examined how heparin in the system might affect platelet deposition. We specifically looked at two types of platelets: those that are activated and bound to subendothelium (PLAS), and those that are activated and bound to deposited platelets (PLAV), and their sum. The platelets accumulate on the subendothelium (SE) and PLAV eventually plateaus due to the limited space at the SE, whereas platelets above the injury site will continue to grow. Increasing the heparin concentrations led to decreases in both platelet species through time.This is because by increasing amount of heparin, it can greatly reduce the thrombin in the reaction zone, which leads to reduced amount of platelet to be activated by thrombin. Such a reduction can cause a shift from platelet-bound platelet to subendothelium-bound platelet. The increase in subendothelium-bound platelet will physically cover up the surface, which can negatively influence the initiation phase of coagulation.

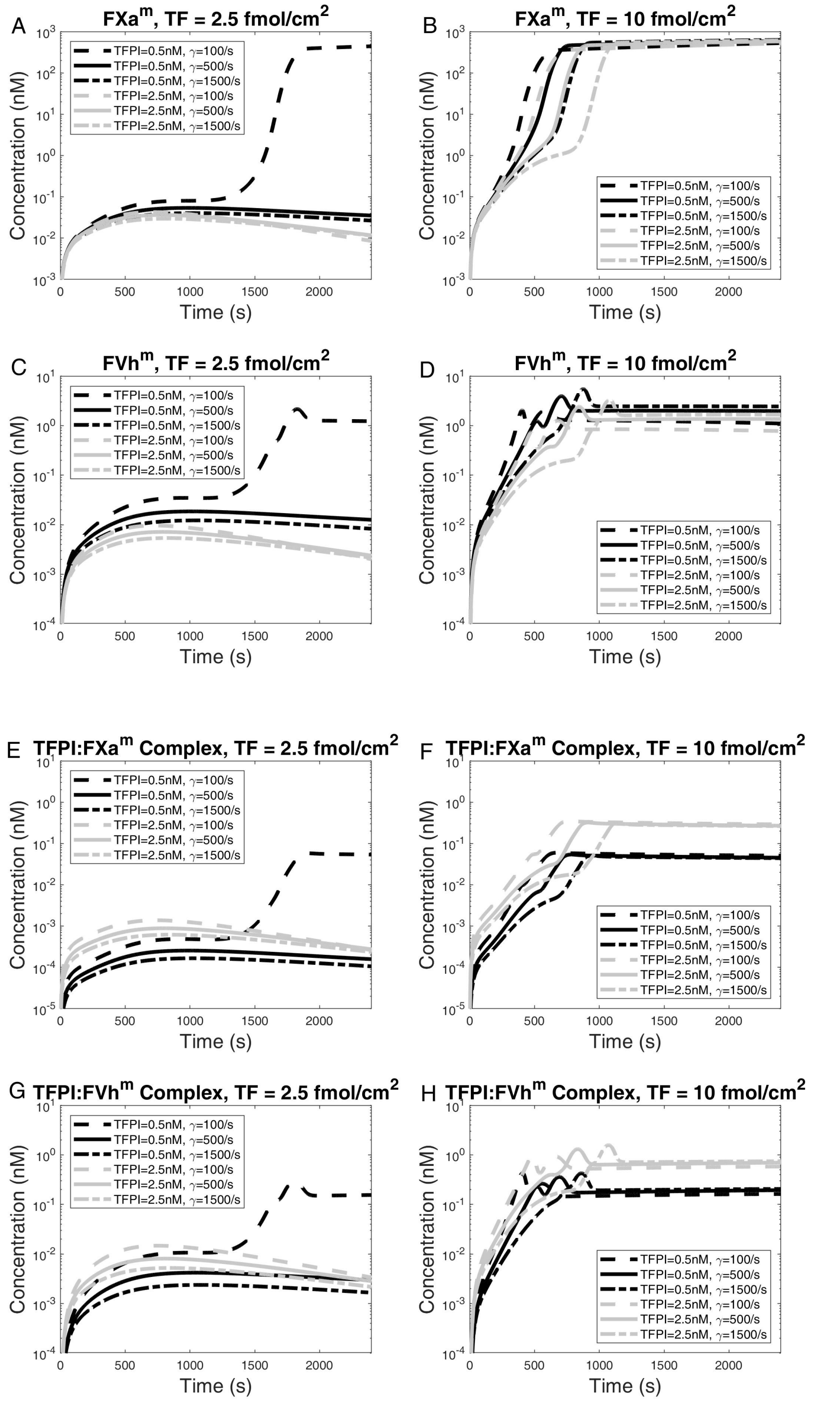

Figure S7: Concentration time course of platelet surface bound FXa, FV-h, and their complexes with TFPI. TF level is varied by 2.5 fmolcm% (A,C,E,G) and 10 fmol/cm^2 (B,D,F,H). Under each TF level, TFPI level is varied by 0.5 nM and 2.5 nM, and shear rate is varied by 100/s, 500/s and 1500/s.

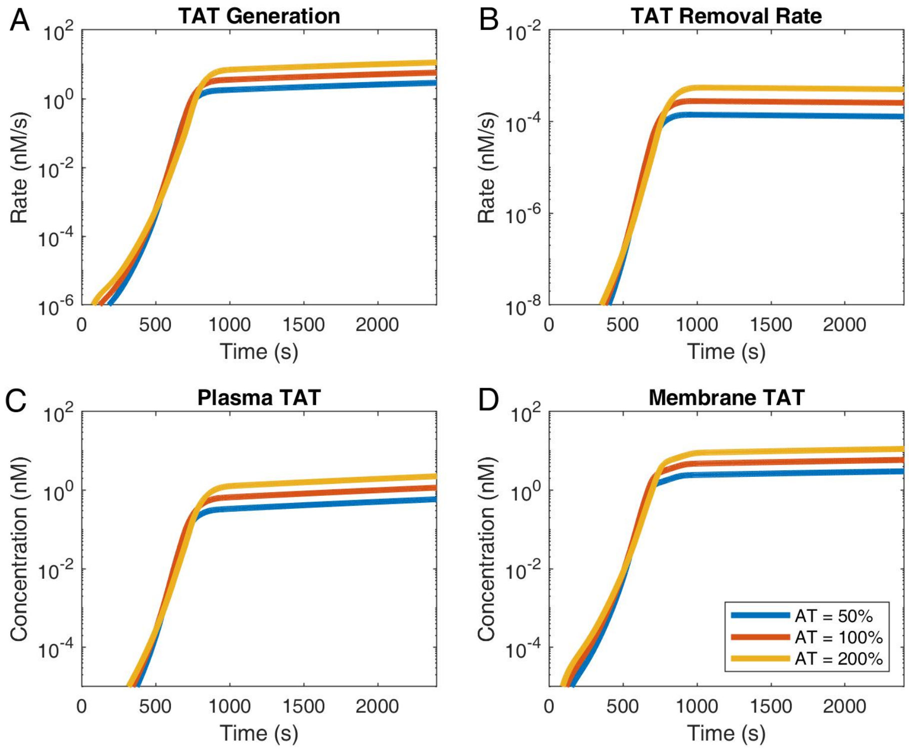

Figure S8: Instantaneous generation and removal of TAT (A,B), and accumulative concentration of TAT in plasma (C) and on the platelet membrane (D). TF level is fixed to 5 fmol/cm^2, and shear rate is fixed to 100/s.

REFERENCES

1. Kuharsky, A. L., and A. L. Fogelson, 2001. Surface-Mediated Control of Blood Coagulation: The Role of Binding Site Densities and Platelet Deposition. *Biophysical Journal* 80:1050–1074. https://linkinghub.elsevier.com/retrieve/pii/S0006349501760857.

2. Fogelson, A. L., and N. Tania, 2005. Coagulation under Flow: The Influence of Flow-Mediated Transport on the Initiation and Inhibition of Coagulation. *Pathophysiol Haemos Thromb* 34:91–108. https://www.karger.com/Article/FullText/89930.

3. Fogelson, A., Y. Hussain, and K. Leiderman, 2012. Blood Clot Formation under Flow: The Importance of Factor XI Depends Strongly on Platelet Count. *Biophysical Journal* 102:10–18. https://linkinghub.elsevier.com/retrieve/pii/S0006349511013142.

4. Turitto, V. T., and E. F. Leonard, 1972. Platelet adhesion to a spinning surface. *Trans. Amer. Soc. Artif. Int. Organs* 18:348–54.

5. Young, M., P. Carroad, and R. Bell, 1980. Estimation of diffusion coefficients of proteins. *Biotech and Bioeng* 22:947–955.

6. Mann, K., M. Nesheim, W. Church, P. Haley, and S. Krishnaswamy, 1990. Surface-dependent Reactions of the Vitamin K-dependent enzyme complexes. *Blood* 76:1–16.

7. Mann, K., E. Bovill, and S. Krishnaswamy, 1991. Surface-dependent reactions in the propagation phase of blood coagulation. *Ann. N. Y. Acad. Sci.* 614:63–75.

8. Morrissey, J. H., 1995. Tissue Factor Modulation of Factor VIIa Activity: Use in Measuring Trace Levels of Factor VIIa in Plasma,. *Thromb. Haemost.* 74:185–188.

9. Novotny, W., S. Brown, J. Miletich, D. Rader, and G. Broze, 1991. Plasma antigen levels of the lipoprotein-associated coagulation inhibitor in patient samples. *Blood* 78:387–93.

10. Weiss, H. J., 1975. Platelet Physiology and Abnormalities of Platelet Function (Part 1). *New Engl. J. Med.* 293:531–541.

11. Walsh, P. N., 1994. Platelet-Coagulant Protein Interactions. *In* R. W. Colman, J. Hirsh, V. J. Marder, and E. W. Salzman, editors, Hemostasis and Thrombosis: Basic Principles and Clinical Practice, J.B. Lippincott Company, Philadelphia, PA, 629–651. 3d edition.

12. Nesheim, M. E., D. D. Pittman, J. H. Wang, D. Slonosky, A. R. Giles, and R. J. Kaufman, 1988. The binding of S-labeled recombinant Factor VIII to activated and unactivated human platelets. *J Biol Chem* 263:16467.

13. Ahmad, S., R. Rawala-Sheikh, and P. Walsh, 1989. Comparative interactions of Factor IX and Factor IXa with human platelets. *Journal of Biological Chemistry* 264:3244–3251.

14. Mann, K., S. Krishnaswamy, and J. Lawson, 1992. Surface-dependent Hemostasis. *Semin Hematol* 29:213–26.

15. Baglia, F., B. Jameson, and P. Walsh, 1995. Identification and Characterization of a Binding Site for Platelets in the Apple 3 domain of coagulation Factor XI. *J Biol Chem* 270:6734–40.

16. Miller, T., D. Sinha, T. Baird, and P. Walsh, 2007. A Catalytic Domain Exosite (Cys^{527}-Cys^{542}) in Factor XIa mediates binding to a site on activated platelets. *Biochemistry* 46:14450–60.

17. Tracy, P., L. L. Eide, E. J. Bowie, and K. G. Mann, 1982. Radioimmunoassay of factor V in human plasma and platelts. *Blood* 60:59–63.

18. Hubbell, J. A., and L. V. McIntire, 1986. Platelet Active Concentration Profiles Near Growing Thrombi. A Mathematical Consideration. *Biophys J* 50:937–945.

19. Olson, S. T., I. Björk, and J. D. Shore, 1993. [30] Kinetic characterization of heparin-catalyzed and uncatalyzed inhibition of blood coagulation proteinases by antithrombin. *In* Methods in Enzymology, Elsevier, volume 222, 525–559. https://linkinghub.elsevier.com/retrieve/pii/007668799322033C.

20. Butenas, S., and K. Mann, 1996. Kinetics of Human Factor VII activation. *Biochemistry* 35:1904–1910.

21. Limentani, S. A., B. C. Furie, and B. Furie, 1994. The Biochemistry of Factor IX. *In* R. Colman, J. Hirsh, V. Marder, and E. Salzman, editors, Hemostasis and Thrombosis: Basic Principles and Clinical Practice, J.B. Lippincott Company, Philadelphia, PA, 94–108. 3d edition.

22. Nemerson, Y., 1992. The Tissue Factor Pathway of Blood Coagulation. *Semin Hematol* 29:170–176.

23. Monkovic, D. D., and P. B. Tracy, 1990. Functional Characterization of Human Platelet-released Factor V and its Activation by Factor Xa and Thrombin,. *J Biol Chem* 265:17132–40.

24. Hill-Eubanks, D., and P. Lollar, 1990. von Willibrand factor is a cofactor for thrombin-catalyzed cleavage of the Factor VIII light chain. *J Biol Chem* 265:17854–8.

25. Lollar, P., G. Knutson, and D. Fass, 1985. Activation of Porcine Factor VIII:C by Thrombin and Factor Xa. *Biochemistry* 24:8056–8064.

26. Gailani, D., and G. Broze Jr., 1991. Factor XI activation in a revised model of blood coagulation. *Science* 253:909–12.

27. Gailani, D., D. Ho, M. F. Sun, Q. Cheng, and P. N. Walsh, 2001. Model for a Factor IX activation complex on blood platelets: dimeric conformation of Factor XIa is essential. *Blood* 97:3117–22.

28. Sinha, D., M. Marcinkiewicz, D. Navaneetham, and P. Walsh, 2007. Macromolecular substrate-binding exosite on both the heavy and light chains of Factor XIa mediate the formation of the Michaelis Complex required for Factor IX activaiton. *Biochemistry* 46:9830–9.

29. Krishnaswamy, S., K. C. Jones, and K. G. Mann, 1988. Prothrombinase Complex Assembly. Kinetic Mechanism of Enzyme Assembly on Phospholipid Vesicles. *J Biol Chem* 263:3823–3834.

30. Mann, K. G., 1994. Prothrombin and thrombin. *In* R. Colman, J. Hirsh, V. Marder, and E. Salzman, editors, Hemostasis and Thrombosis: Basic Principles and Clinical Practice, J.B. Lippincott Company, Philadelphia, PA, 184–199. 3d edition.

31. Greengard, J., M. Heeb, E. Ersdal, P. Walsh, and J. Griffin, 1986. Binding of coagulation Factor XI to washed human platelets. *Biochemistry* 25:3884–90.

32. Monkovic, D., and P. Tracy, 1990. Activation of human Factor V by Factor Xa and thrombin. *Biochemistry* 29:1118.

33. Mann, K. G., 1987. The assembly of blood clotting complexes on membranes. *TIBS* 12:229–233.

34. Rawala-Sheikh, R., S. Ahmad, B. Ashby, and P. N. Walsh, 1990. Kinetics of coagulation Factor X activation by platelet-bound Factor IXa. *Biochemistry* 29:2606–11.

35. Nesheim, M. E., R. P. Tracy, P. B. Tracy, D. S. Boskovic, and K. G. Mann, 1992. Mathematical Simulation of Prothrominase. *Methods Enzymol.* 215:316–328.

36. Olson, S. T., R. Swanson, E. Raub-Segall, T. Bedsted, M. Sadri, M. Petitou, J.-P. Hérault, J.-M. Herbert, and I. Björk, 2004. Accelerating ability of synthetic oligosaccharides on antithrombin inhibition of proteinases of the clotting and fibrinolytic systems Comparison with heparin and low-molecular-weight heparin. *Thrombosis and haemostasis* 92:929–939.

37. Solymoss, S., M. Tucker, and P. Tracy, 1988. Kinetics of inactivation of membrane-bound Factor Va by activated protein C. Protein S modulates Factor Xa protection. *J Biol Chem* 263:14884–90.

38. Jesty, J., T.-C. Wun, and A. Lorenz, 1994. Kinetics of the Inhibition of Factor Xa and the Tissue Factor-Factor VIIa Complex by the Tissue Factor Pathway Inhibitor in the Presence and Absence of Heparin. *Biochemistry* 33:12686–12694. https://pubs.acs.org/doi/abs/10.1021/bi00208a020.

39. Maroney, S. A., and A. E. Mast, 2015. New insights into the biology of tissue factor pathway inhibitor. *J Thromb Haemost* 13:S200–S207. https://onlinelibrary.wiley.com/doi/10.1111/jth.12897.

40. Jesty, J., T. Wun, and A. Lorenz, 1994. Kinetics of the Inhibition of Factor Xa and the Tissue Factor-Factor VIIa Complex by the Tissue Factor Pathway Inhibitor in the Presence and Absence of Heparin. *Biochemistry* 33:12686–12694.

41. Griffin, J. H., 2001. Control of Coagulation Reactions. *In* E. Beutler, B. Coller, M. Lichtman, T. Kipps, and U. Seligsohn, editors, Williams Hematology, McGraw Hill, New York, 1435–1447.

42. Olson, S., I. Björk, R. Sheffer, P. Craig, J. Shore, and J. Choay, 1992. Role of the antithrombin-binding pentasaccharide in heparin acceleration of antithrombin-proteinase reactions. Resolution of the antithrombin conformational change contribution to heparin rate enhancement. *Journal of Biological Chemistry* 267:12528–12538. https://linkinghub.elsevier.com/retrieve/pii/S0021925818423095.

43. Broze, G., and J. Miletich, 1994. Biochemistry and Physiology of Protein C, Protein S, and Thrombomodulin. *In* R. W. Colman, J. Hirsh, V. J. Marder, and E. W. Salzman, editors, Hemostasis and Thrombosis: Basic Principles and Clinical Practice, Lippincott Company, Philadelpha, 259–276.

44. Turitto, V. T., and H. R. Baumgartner, 1979. Platelet Interaction with Subendothelium in Flowing Rabbit Blood: Effect of Blood Shear Rate. *Microvasc. Res.* 17:38–54.

45. Turitto, V. T., H. J. Weiss, and H. R. Baumgartner, 1980. The Effect of Shear Rate on Platelet Interaction with Subendothelium Exposed to Citrated Human Blood. *Microvasc. Res.* 19:352–365.

46. Kuharsky, A., and A. Fogelson, 2001. Surface-mediated Control of Blood Coagulation: The Role of Binding Site Densities and Platelet Deposition. *Biophys J* 80:1050–1074.

47. Gear, A. R. L., 1994. Platelet adhesion, shape change, and aggregation: rapid initiation and signal transduction events. *Can. J. Physiol. Pharmacol.* 72:285–94.

48. Byun, J.-H., I.-S. Jang, J. W. Kim, and E.-H. Koh, 2016. Establishing the heparin therapeutic range using aPTT and anti-Xa measurements for monitoring unfractionated heparin therapy. *Blood Research* 51:171. https://synapse.koreamed.org/DOIx.php?id=10.5045/br.2016.51.3.171.

49. Bangham, D., and M. V. Mussett, 1959. The Second International Standard for Heparin. *Bulletin of the World Health Organization* 20:1201.

50. Linhardt, R. J., 2016. Heparin and anticoagulation. *Frontiers in Bioscience* 21:1372–1392. https://imrpress.com/journal/FBL/21/7/10.2741/4462.

51. Hirsh, J., and M. Levine, 1992. Low molecular weight heparin. *Blood* 79:1–17. https://ashpublications.org/blood/article/79/1/1/173040/Low-molecular-weight-heparin.

52. Link, K. G., M. T. Stobb, J. Di Paola, K. B. Neeves, A. L. Fogelson, S. S. Sindi, and K. Leiderman, 2018. A local and global sensitivity analysis of a mathematical model of coagulation and platelet deposition under flow. *PLoS ONE* 13:e0200917. https://dx.plos.org/10.1371/journal.pone.0200917.
